# Periscope

**Published:** 1832-10-01

**Authors:** 


### Periscope;

OR,

### CIRCUMSPECTIVE REVIEW.

"Ore trahit quodcunque potest, atque addit acervo."

### Various Selections from the French Journals.

I.

### Remarks on the Fetid Abscesses, which frequently form in the Neighbourhood of Mucous Membranes.

Many of the laws, which were supposed formerly to regulate and to be peculiar to the actions of living bodies, have been of late years disputed; and physiology has called in the aid of physical science to explain a number of pathological phenomena. The course of the fluid in the different tissues has been found, in many respects, to obey the ordinary rules of chemical and mechanical agency, as it will be illustrated by the transmission both of liquids and of gases, through the medium of imbibition, from one structure to another, without the intervention of the circulatory or absorbent systems. The formation of many abscesses is intimately connected, according to M. Velpeau, with such a process. His attention was first excited, by observing that many collections of matter, situated in the cellular or muscular structures, and apparently the result of active phlegmonous inflammation, are filled with an offensive and fetid pus, although there is no communication between them and any carious bones, or internal viscera. Such abscesses are observed around the mouth, at the sides or in front of the larynx or trachea, at the margin of the anus, and still more often in the abdominal parietes; in short, along the track of all organs which are covered with a mucous coat, and whose walls are more or less soft and more or less extensible. Every surgeon must have remarked the stench of many collections of matter formed in the gums, or round the mouth and anus. A few examples, however, will confirm the truth of this statement.

*Case* 1. A man had a large diffused swelling on the side of the face; it pointed between the cheek and arch of the upper jaw, and had existed only for six days. On opening it with a lancet, about half a wine-glassful of blackish-yellow pus, excessively putrid, escaped. One of the teeth was slightly carious; the abscess was quite healed in the course of five days.

*Case* 2. A woman, aged 50, had an immense abscess, which occupied all the side of the left cheek and jaw; it broke, and so abominable was the stench of the matter, that few could approach her. The matter continued to flow, and shreds of dead, cellular substance were extracted; in a month she was well.

In this case there was no caries of the teeth, nor any disease of the maxillæ, and although there was no communication with the cavity of the mouth, the fetid smell was perceived immediately after it burst, and became less and less offensive when the abscess was thereby exposed to the external air.

*Case* 3. A man, aged 30, had a fluctuating swelling in front, and extending a little to the left side of the larynx. On opening it, the surgeon's attention was struck by the extreme fetor of the contents, and inferred that the os hyoides or laryngeal cartilages were diseased; or else that some communication existed between the abscess and the gastro-pulmonary passages. A careful examination, however, proved quite convincingly that it was a simple, uncomplicated collection of matter, reaching from the parotid gland to the middle of the neck, and was neither connected with any caries or ulceration of the hard parts, nor had any opening into the larynx or œsophagus. The patient died of another disease, and the accuracy of the above account was confirmed by dissection.

*Case* 4. A man, aged 35 years, had suffered much from pain, &c. around the anus for 10 days before he applied for advice. An abscess existed close to the sphincter: it was freely opened, and much blackish, stinking pus, mixed with lumps of putrefied substance, and smelling strongly of fæces, was evacuated. The probe did not enter the gut, and the surgeon concluded that the abscess, in spite of its fetid contents, was simply phlegmonous, and might heal without any operation. The patient was discharged, cured, in 15 days.

*Case* 5. A woman, aged 57, thin, but healthy, was suddenly seized with severe colic in the middle of December, 1831. Two days after, a swelling appeared in the lower and right side of the abdomen; it was painful on pressure, gradually became more circumscribed, and soon attained the size of two fists. It burst in January, and discharged a large quantity of blackish-grey, grumous pus, smelling very strongly of the intestinal contents; some gas also escaped, and a few pellets of dead cellular substance. A careful examination with the probe could detect no communication with the cavity of the peritoneum, or any part of the gut; and the speedy cure made it certain that the matter had been formed in the muscular parietes.

Le Dran has recorded the following case.

*Case* 6. A man, aged 24, was, by his own account, seized with symptoms of enteritis. A tumor appeared in the right groin, and extended to the umbilicus; it was opened by a bistoury, and discharged much fetid pus. Le Dran thought that this abscess had its seat between the omentum and the muscular parietes; but the successful result sufficiently proves that it was not connected with the abdominal cavity.

*Case* 7. A young woman had a phlegmonous swelling in the hypogastric region. On puncturing it, a milky fluid and an offensive pus were evacuated. The patient died, and the abscess was an enormous sac, situated between the peritoneum and the muscles.

*Observations on the preceding Cases.*—Abscesses in the soft, spongy textures of the gums are very common; the fœtor of the pus has usually been attributed to the co-existence of disease of the teeth, or of the maxillary bones; but this explanation is not satisfactory in all cases, for such disease often does not exist. M. Velpeau rather ascribes it to air having entered by imbibition, and the matter becoming thereby vitiated from the resulting chemical changes; for in what other manner should abscesses, whose symptoms, progress, and maturation, differ in no respect from ordinary phlegmonous inflammations, be filled with a highly offensive pus, before the possible introduction of air from without or within through any opening, and which are not accompanied with any diseased bones. It is very interesting to remark that these abscesses are observed in such regions as are lying close to mucous canals, and separated from these only by very thin walls. In the neck we find them between the fascia cervicalis and the thyro-hyoid membrane, or the pharynx, the œsophagus, or the trachea; under the jaw, between the supra-hyoid aponeurosis, and the inferior wall of the cavity of the mouth; in the face, imbedded deep in the flesh of the cheeks—so that the air very probably permeates the texture of the mucous linings, and thus blends with the contents of the sac. The phenomena alluded to are still better observed, when abscesses form round the margin of the anus. Many surgeons deem the stercoraceous smell, emitted from such abscesses, as unequivocally denoting the existence of genuine fistulæ in ano. M.

Velpeau assures us that this idea is frequently quite erroneous, and reports a case in confirmation. A man had a very extensive abscess, situated close to the sphincter, and, when opened, every one supposed, from the stench of the discharge, that it must have communicated with the gut. The suppuration, however, speedily dried up, and the sore was cicatrized in a few days. Many analogous cases are narrated. M. Velpeau has, after a careful examination of these, come to the conclusion that a large proportion of true fistulæ ani are originally mere abscesses on the outside of the gut, and that the fistulous aperture into the bowel is a subsequent occurrence. Fortunately, purulent matter has always a tendency to the exterior of the body; else the thin walls of the gut would, in every case, be inevitably diseased.

When a fetid abscess forms in the abdominal parietes, M. Velpeau thinks that they are developed originally in, or at least take their start from, the fascia propria, or cellular tissue which unites the peritoneum to the abdominal muscles. The purulent matter being thus deeply lodged, is supposed to receive, by transudation from the bowels, an admixture of gas or of liquids, or perhaps only of odorous effluvia. What induces M. Velpeau to believe that such a transudation does really take place, is, that he has observed 'that the smell of the pus varies with the situation in the abdominal parietes where the abscess is formed. Thus, in one case where an abscess existed in the right groin, a strong and distinct stercoraceous odour was emitted from the pus; whereas in another, where the abscess was situated in the epigastric region, the smell was sourish, and not unlike that of imperfectly digested food. Besides, the colour of the discharge is found to vary much in different instances, and may be often observed to be somewhat indicative of the substances which may be contained in the bowel, lying next to the posterior wall of the abscess.

The fetid abscesses of the abdominal parietes are of tedious formation, and are preceded by dull, deep-seated pain, by a tumefaction more diffuse than circumscribed, and by symptoms of general feverish irritation. When the skin becomes prominent, still the fluctuation is often very indistinct; because, in consequence of the thickness and toughness of the integuments, the matter burrows deep, separating the peritoneum from the muscular layers. Sometimes, though very rarely, the contents are discharged into the cavity of the abdomen. Le Dran reports such a case. The rarity of the occurrence is to be attributed to the tendency of suppuration towards the surface, and also to the uniform and equable pressure made by the contained viscera upon the posterior wall of the cyst. The agency of this second cause has hitherto been overlooked; but probably it is the more efficacious of the two. In all abscesses, formed in the abdominal parietes, it is necessary to make a free and large incision. M. Velpeau says that the more extensive the opening, the less danger there is from any re-action of the atmosphere, either on the walls of the cavity or on its contents; and also from the occurrence of the fever of "absorption," and of those symptoms of prostration which are not unfrequent in such cases. As to the dressing of the sore, it is to be conducted in the usual manner.*—*Journal Univers. et Hebdom.*

---

### II.

### Case of Aphonia, treated by Nitras Argenti.

A young woman had gradually lost her voice, from repeated attacks of catarrh; her general health was quite good. During deglutition, she experienced an uneasiness in the larynx. When she did not make any very strong effort to speak out, her voice resembled a low whisper; but if animated, and anxious to exert her speech, a noise, like a succession of shrill whistles, or of the mewings of a kitten, was produced. M. Trousseau treated the case

* We are not to be understood as implicitly agreeing with M. Velpeau in these views.—*Ed.*

by the local application of a strong solution of lunar caustic, introduced by means of a sponge affixed to a bougie, and gently pressed over the opening of the larynx. The operation was followed by retchings, vomiting, and a cough which nothing could allay. These distressing symptoms abated in about a quarter of an hour, and then the patient assured M. T. that she felt little pain when the caustic was applied; and she expressed her astonishment that already her voice was much stronger, and that she could speak louder than she had been able to do for eight months. She was ordered to gargle her mouth and throat with a solution of alum. The retching and vomiting recurred at intervals till the following day, and the pain of the operation continued for a day or two longer. The patient was, however, able to drink, and to swallow soup, &c. What is strange is, that although, almost immediately after the application of the caustic, the voice was so much improved, at the end of 10 days the aphonia was as bad as ever. The remedy was re-applied, but in a still stronger form. A sponge, dipped in a saturated solution of the caustic, was introduced as far as the opening of the larynx, and about 10 drops allowed to distil into it. Immediately a violent convulsive cough arose, by which most of it was expelled in the Doctor's face! The pharynx, tongue, and lining membrane of the cheeks were rendered as white as milk. Vomiting and a severe cough came on; fortunately there was but little pain, no fever or constitutional disturbance. Her voice did not receive the speedy amendment, as after the first operation. The cough was very troublesome for 12 days; but the voice appeared to have obtained strength, although it was very hoarse. After the 13th day, she was able to speak for some minutes in succession, and the aphonia never afterwards returned. The voice, at first hoarse and occasionally squeaking, became more and more clear; however, after a long walk, or exposure to cold, or too much talking, the hoarseness still returns, but not the aphonia.—*Journ. Univers. et Hebdom.*

### III.

### CASES OF RHEUMATISM, TREATED BY THE LOCAL USE OF THE ACETATE OF MORPHIA.

A WOMAN, aged 28, had a smart attack of rheumatism in both knees, the shoulders, and wrists; the pains were constant, and the affected parts were considerably swollen and red. She was freely bled from the arm, without relief—blood sizey. A blister on each knee ordered, and the raw surfaces to be dressed with half a grain of acetate of morphia; for the three subsequent days she was quite free of all pain—the quantity of the morphia increased to three quarters of a grain. In two days more she had completely recovered.

*Case* 2. A man, aged 27, was seized with violent pains in both knees on the 15th March. On the 22d, the joints were blistered, and the morphia applied in the dose of a quarter of a grain. In two days the pains were much relieved. On the 28th, he was pronounced cured.

*Case* 3. A young man, aged 16, suffered unremitting pains in the knees, wrists, and elbow-joints, which were swollen, red, and very tender on pressure, and incapable of being moved. For three nights he could not sleep, from the severe suffering; pulse full, skin burning hot: he was bled and starved. Next day the pulse was more favourable, but the pains rather worse: a blister to be applied to each knee, and the surfaces to be dressed with a quarter of a grain of the acetate of morphia. This treatment was continued for four days, when the patient made no complaints but from the blistered surfaces.—*Journal Hebdom.*

---

### IV.

### CASE OF SEVERE SCIATICA, CURED BY BLOODLETTING.

A. R. aged 39, had suffered for a month from excruciating pains in the upper and back part of the right thigh; they shot down on the outer side of the limb to the ankle and foot, and generally ceased with a distressing shaking or trembling of the extremity. These paroxysms returned almost every hour, and were so agonizing as to force the patient to scream out. While they lasted, the whole limb appeared to be affected with a tetanic spasm, the gastrocnemii muscles being drawn into hard knotty swellings. During the intermissions, there was a constant, slightly-hot pain along the course of the sciatic nerve. He was largely bled from the arm, and 30 leeches ordered over the sciatic notch, to be followed by opiate poultices. On the following day, the number of paroxysms was reduced by one-half, and the character of the pain changed from cramp and the feeling of burning, which shot like a rocket from the hip to the toes, to that of cold water gliding rapidly under the skin in the same direction ; leeches to be re-applied. In two days the pains had quite disappeared ; and in order to confirm the cure, the patient was ordered to rub in 20 drops of croton oil on the outside of the thigh ; a copious eruption of pustules was produced, and, in six days more, the patient was discharged cured.—*Journ. Hebdom.*

---

V.

### Paralysis of the Portio Dura, cured by Moxas.

A. M. aged 43. The mouth was much drawn to the right side, and the right commissure pulled considerably upwards, while the left was directed downwards and inwards, leaving the front teeth exposed; the left cheek was much stretched, and closely applied to the alveolar arches ; the left eyelids closed imperfectly, and thus part of the cornea was unprotected ; the cause of this was chiefly attributable to the upper lid being drawn up by its levator muscle. Considerable conjunctivitis had ensued, but the vision did not seem affected. The food was always involuntarily carried to the right side of the mouth ; the saliva and any drink trickled from the left angle of the mouth down his chin ; the speech, at first, was not affected, but afterwards he could not pronounce certain words ; the sensibility of the parts, and the functions of taste and smell, were perfect. The symptoms had existed for seven days, and no assignable cause could be stated. A moxa was applied over the condyle of the lower maxilla, and good effects were apparent, on the second day, in those parts which are supplied by the middle or transverse branch of the facial nerve ; a second moxa was, therefore, ordered over the angle of the jaw, and speedily the speech became more distinct, the deformity of the mouth less, the food was more easily and steadily retained between the teeth in mastication, and liquids could be better retained. When the patient attempted to pronounce the letter "O," the hiatus of the mouth was decidedly less on the affected side than hitherto ; still the motions of the left eyelids were very imperfect, and, therefore, two days after the last moxa, a third was ordered over the course of the ascending twig of the nerve. The effects were most gratifying ; for on the same evening, the patient could close the lids quite accurately, the ophthalmia disappeared, and in 17 days the patient was discharged well.

*Reflections.* This case shews satisfactorily that such affections of the facial nerve are frequently local, and not at all connected with cerebral disease. The occurrence of the paralysis successively in the different branches of the nerve, and the effects of the treatment, first on one of these branches, and then on the other two, are very interesting, and confirm the opinion just stated. The propulsion of the food between the teeth and cheek of the right, or healthy side, causing it to be distended, and requiring the finger to bring the mouthful between the teeth, is rather contradictory to what is usually observed.—*Journ. Univers. et Hebdom.*

---

VI.

EPILEPSY CONNECTED WITH SCROFULOUS DISEASE OF THE CEREBELLUM.

R. S. aged 36, was carried to the Hôtel Dieu on the 7th October, 1831, while under an epileptic fit—he had been subject to such attacks for a long time, and they returned usually every month. When he coughed, a sharp pain was felt at the back part of the head. There was a swelling at the lower part of the dorsal region, caused by the projection of the lowest dorsal and upper lumbar vertebræ; this had existed for six or seven years. This patient had a great reluctance or an inability to stand for any length of time, or move forward; yet he could walk if he chose to compel himself. He died very suddenly, and the dissection revealed the following appearances. The swelling on the back arose from the bunching out of the spinous processes of the vertebræ; the bodies of which were softened and wasted—the dura mater of the medulla at the part was diseased, and the canal filled with purulent matter. In the upper lobe of the left lung, was found a tuberculous mass, passing into a chalky state; and quite at the summit of this lobe there was an empty pouch or cavity, lined by an apparently serous membrane.

The substance of the cerebrum was much firmer than usual, as if it had been steeped in an astringent fluid. The lateral ventricles were enormously distended by a lemon-coloured serum, amounting to 4 or 5 oz. in quantity. The cerebellum was also much firmer in consistence, and on dividing it vertically a tuberculous mass in the state of infiltration into the medullary tissue and about an inch in extent, was found to rest on the posterior wall of the 4th ventricle, which had thus been considerably contracted in size.

*Reflections.* It is well known that many physiologists have placed the seat of physical love in the median, or central part of the cerebellum; now this was exactly the part which was found diseased in the present case; and yet the man retained all sexual desires; which were neither encreased, impaired, or perverted.

The opinion of M. Magendie that the median lobe of the cerebellum is, as it were, the regulator of the voluntary movements of the body may be supposed to be neither confirmed, nor confuted by the particulars observed in this patient, in consequence of the co-existence of spinal disease.

An interesting case however of tubercular disease in the median part of the cerebellum, without any complication, is detailed in the Bulletin de la Société Anatomique. A soldier aged 20 suffered from excruciating head-aches, general weakness, tottering of the limbs, and great reluctance to be moved; he voided his urine and fæces in bed; but whether this proceeded from a paralysis of the sphincters, or from sheer unwillingness to be disturbed in bed, could not be ascertained—appetite considerable—mental faculties impaired. On dissection, the lateral ventricles were found distended with water; and on the upper part of the cerebellum close to its median line, a tubercular deposit of a yellowish colour, about three inches in circumference was observed. The substance of the cerebellum was in a state of ramollissement for the space of two lines round the tumour, with which however it was not incorporated. This case therefore corroborates the opinion that cerebellar disease generally causes a great diminution in the muscular powers of the lower extremities.

Another reflection arising from the study of the case which we have related is; in what manner are we to explain the periodicity of the epileptic seizures? they occurred in the present instance usually once every month. Are we to regard the tuberculous disease of the cerebellum as the "fons mali"? Our answer will probably be in the negative, and the reason we assign is, that the morbid structure could not be changed in its form or extent, at stated intervals. Perhaps, therefore, we should take into our consideration the action of the cephalo-spinal fluid, to which Magendie has drawn the attention of medical men. The lateral ventricles being much distended with water; and the diseased mass compressing the fourth ventricle, and consequently obstructing the opening of communication, discovered by M. Magendie, between the fourth ventricle, and the sub-arachnoid cellular tissue of the medulla, all the effused fluid was necessarily collected in the cerebral ventricles, and to such an extent at certain intervals, as to overcome the pathological reaction of the brain, and thus to induce the irregular movements of epileptic convulsions.—*Journ. Hebdom.*

*Remarks.* The language of this last sentence is not very easily understood.—Ed.

VII.

Epidemic Disease among Poultry.

While the cholera is raging in Paris, at Choisy-le-Roi, which has been spared from this scourge, a very fatal epizootic plague has appeared. This coincidence has not unfrequently been noticed in other epidemic diseases; sometimes horses, cows, sheep, &c. have been the victims; but more rarely has any description of bird suffered. The cholera had scarcely broke out in Paris, before a dreadful mortality among the poultry of the country round, was announced. At first this was attributed to poisoning; but soon this idea was abandoned; and the cholera itself was denounced as the destroyer; and now every one affirmed that the hens were vomited and purged, were cramped in their wings and legs, and became quite blue before death!! Be this as it may, it is very certain that the germ of cholera did not remain in the corpse, as many of the fowls, which had been victims, were eaten, without causing any bad effects!! The disease broke out on the 3rd of April; no cause could be assigned for its appearance; and it was decidedly not contagious. Ducks and geese escaped, though feeding in the same yard, and only three turkeys went to Orcus! The mode of seizure was as follows. The bird appeared to be feeble and unable to move, resting squat upon its tail; the crop was found distended with food—the breathing became short and hurried; the pulsations of the heart much quickened; diarrhœa of whitish liquid matter supervened; the throat and beak were choked up with a glairy mucus; the comb at first was of a livid hue, and became more and more purplish as death approached—convulsions frequently preceded the death. Almost every bird that was seized of the disease died. The intestines, when examined, appeared to be inflamed at various points; the liver was gorged with blood, and the gall-bladder distended with very thick green bile.—*Journ. Hebdom.*

VIII.

Diabetes Mellitus.

A soldier had suffered dreadfully for five months from a thirst which he could not quench; from great dryness and heat of the surface, &c. The quantity of urine per diem exceeded fifteen litres. He was put on a diet of strong animal food; rubbed embrocations on the skin, used sulphureous baths, and had moxas, and cupping glasses occasionally applied over the kidneys. The quantity of urine was reduced to six litres in three weeks; and he found himself so much better that he refused to remain longer in the hospital. On analyzing the urine, much saccharine matter was readily detected, in the proportion of an ounce and a half of a thick honey-like fluid, in every litre of urine. Neither urea, nor uric acid could be discovered; and the sulphates and phosphates were much diminished in quantity.—*Annales de la Medécine Physiol.*

IX.

Scrofulous Disease of the Bones.

*Case* 1. A young girl had long laboured under scrofulous disease of the shoulder joint; it was much swelled, and covered with numerous ash-coloured ulcers, from which flowed a profuse discharge—there were several fistulæ running towards the head of the humerus; but the bone could not be detected bare, by the probe. The joint was anchylosed; and the limb quite wasted—diarrhœa and hectic fever had brought the patient to death's door. The constitutional symptoms were first corrected; and then the use of iodine commenced, externally in the form of baths thrice a week; and the shoulder was daily rubbed with an ointment of ioduret of lead. As the patient bore the use of the iodine well, its inward use was now employed, and two ounces of the mineral iodated water given, the dose being gradually encreased to 8 and 12 ounces. The fistulæ were injected with a solution of iodine. The beneficial effects were speedily obvious; the joint was much reduced in volume, and the suppuration diminished in quantity. After the iodine had been taken for nearly two months, its use was suspended for three weeks, and diluents and aperients administered, with the view of counteracting any pernicious effects which the drug might have on the stomach, or on the general health; but again resumed and continued for six to eight weeks. In this way the medicine may be exhibited for a great length of time without any danger, especially if the preparation given is the solution of the hydriodate of ioduretted potass (a grain and a half of iodine, and three grains of hydriodate of potass in the course of the day.)

This patient obtained the cure of all the sores, and the state of the joint, though necessarily remaining anchylosed, was vastly improved.

*Case* 2. A child aged seven presented the left shoulder much puffed and enlarged; numerous fistulæ opened over the head of the bone, which was certainly much enlarged in size; the limb was quite atrophied—the motions of the joint were almost entirely lost—he had cough, diarrhæa, and evening fever. Mild nourishing food was ordered; the mineral iodated water given inwardly, and the shoulder rubbed with the ointment of ioduret of lead. In a fortnight the child was much improved; and to the above treatment was now conjoined frequent bathing in a river. The fistulæ were injected with a lixivial wash. At the end of three months, the movements of the joint had become much more free; the shoulder was very little swelled, and the limb had acquired flesh and strength. The iodine had been omitted at two periods, during which diluents and an occasional aperient were administered. The good effects were well marked in this case; the disease had not existed sufficiently long to expect a spontaneous cure; many anti-scrophulous remedies had been previously tried, and had all failed; but the iodine treatment had not been continued above a week or two, till the little patient shewed signs of improvement. These two cases are illustrative of strumous enlargement of the osseous texture. We shall now say a few words on the subject of caries. This morbid state most commonly attacks the small bones, as the phalanges of the toes and fingers, the metacarpal and metatarsal bones, and those of the tarsus and carpus. It is not rare in the vertebræ, or even in the sternum, ribs, and other long bones. Sometimes, but not always, it is preceded by swelling of the periosteum, or of the bony structure itself. A dull deep-seated pain, without any appreciable cause, or it may be, supervening after a lapse of time to a contusion, is the earliest symptom; to this is soon added a general tumefaction, and agglutination of the surrounding soft parts. An abscess at length is formed and discharges a thin greyish matter mixed with albuminous flocculi, and sometimes with portions of bone. The probe will often detect the roughness of the exposed bone; and in a few cases will penetrate into the cavity of a joint; the existence of such a fistula may be also inferred from the admixture of synovial fluid with the discharge. M. Baudelocque, the author of this memoir, states that he has exhibited iodine in upwards of 30 cases of scrophulous caries of the bones; but seldom with any decided success, amounting to a cure. Its action howevever was very obviously as beneficial to a certain extent in these, as in any other examples of strumous disease; but since morbid changes in bones are more slowly formed, and more slowly advance, than in softer textures, so the cure must necessarily be more tedious and protracted. A period of six months, and upwards, will be generally found necessary; and it is seldom that we can have patients, especially in public institutions of charity, submitted to treatment for such a length of time. The following four cases will illustrate the principles recommended.

*Case* 1. M. L. aged 9, was presented at the hospital on the 1st April, 1831. In addition to scrophulous disease of the eyes and lids, there were numerous fistulous openings in the skin over the sternum, which was found bare and rough by the probe. Two other fistulæ existed on the outer side of the mamma, just below the anterior fold of the axilla; and the rib at this part was also carious; a fluid injected through these last-named openings escaped by those situated over the sternum. The child laboured under the effects of mercurialism; profuse diarrhœa and hacking cough. The constitutional irritation having been first subdued, recourse was had to iodine. In the beginning of June the iodated water was given in the dose at first of two ounces, and gradually encreased to 10 ounces in the course of of the day—three iodated baths were ordered every week. This treatment was continued for five weeks, and then suspended for a fortnight or more. In addition to the former treatment, the fistulæ were now injected with a concentrated solution of iodine. By this time the ophthalmic disease was quite cured; and the discharge from the abscesses much diminished and improved. In a few months, the caries of the sternum and ribs was conquered, the fistulæ healed, and the patient discharged well.

*Case* 2. S. D. aged seven years, was very similarly affected to the last patient; obstinate disease of the eyes and lids, and caries of the two first phalanges of the left fore-finger, and of the fifth metatarsal bone of the same side. The iodine was administered in the form of drink, baths, and injections, and the cure was ultimately complete.

*Case* 3. T. S. aged 12. The left elbow-joint much swelled; the skin over it of a violet-red colour, and disfigured by numerous scars, and fistulous apertures, from which there flowed a profuse discharge. The bones found bare on the introduction of a probe. The fore-arm bent on the arm at a right angle, and could not be either bent, or extended—no motion between the radius and ulna. The general health was well attended to, and iodine administered both externally and internally. In four months all the fistulæ were cicatrised, and the motions of the joint much more considerable.

*Case* 4. M. L. aged 8. Almost complete anchylosis of the right elbow-joint, which is much swollen and puffed; there are five scrofulous ulcers, two of which penetrate to the humerus and ulna, which are found bare and rough. General health good, with the exception of an obstinate diarrhœa. At first sulphureous baths were employed; also local alkaline baths to the joint, and alcalis were given inwardly; but no success ensuing, cinchona was ordered, and an iodated bath thrice a week:—the ulcers to be dressed with an ointment containing ioduret of lead. In the course of a month, the mineral iodated water was given internally in the dose of from 4 to 6 ounces. With an occasional intermission this plan of treatment was continued for about three months, by which time the ulcers had healed; the swelling disappeared, and the anchylosis much diminished. We cannot fail to attribute the rapidity of the cure to the means employed; no spontaneous cure is ever effected so speedily; the patient had been in the hospital nearly 12 months, before the iodine was employed; and in that period very little progress had been made; whereas in three months afterwards the disease was subdued.—*Revue Médicale.*

---

### X.

### Puerperal Peritonitis, followed by Ascites and Spontaneous Perforation of the Abdominal Parietes.

F. G. æt. 30, was delivered April, 1830, of her 7th child. On the second day after her confinement, she was much agitated; and the consequence was, that severe pains in the abdomen came on, the lochia were suppressed, and the mammæ became flaccid. The symptoms were alternately relieved and aggravated for eight days; and at this period Dr. Puntons was called in. He found the patient much dispirited and alarmed; belly distended, sonorous, and painful on pressure; distinct fluctuation; suppressed lochia; urine much diminished in quantity, obstinate constipation, severe headach, tongue clean, much thirst, pulse irregular, somewhat hard and very frequent; œdema of the feet and ankles.

*Treatment.* Emollient fomentations, mercurial frictions on the inside of the thighs, calomel and nitre inwardly. Diet, chicken broth. This plan of treatment was persevered in for a week; the abdominal tenderness had much abated; but the distension had become greater; pulse less hard—a blister ordered to be applied to each thigh. On the 18th day from the commencement of the attack, an opening or slit occurred in the abdominal parietes, about an inch below the umbilicus; the part had been smooth and glistening for a few days before. About a pint of yellowish, inodorous fluid escaped. Two days afterwards a similar vent took place in the upper part of the left hypogastric region, and an ounce or two of a citron coloured serum flowed out. Simple dressings applied to the wounds, and firm compresses to the abdomen, in order to promote the expulsion of the fluid. The woman gradually recovered.

*Observations.* An analogous case is mentioned by Frank. The effused fluid was visible through the navel. The author of the Nosographie Philosophique narrates an example of puerperal peritonitis, in which a large abscess formed in the left labium; on the 14th day, it broke, and the patient was speedily cured. Van Swieten and Chomel allude to many cases in which the abscesses had found their way into the intestines and caused a purulent diarrhœa.—*Revue Médicale.*

### XI.

### ON SOME OBSCURE FORMS OF THE PHLEGMASIÆ.

AN inflammation, when its symptoms are uncomplicated and unobscured may be always very easily detected, and very certainly cured; cases however frequently occur, in which the characters of the disease are modified, and curtailed of their accustomed distinctness; and in the management of which unusual attention is demanded of the physician, as the attack is generally insidious, and the progress irregular and ill defined. The danger of such cases is ten fold greater than of others, in which the symptoms may be much more violent and formidable; as the activity of the remedies may be easily proportioned to the severity of the malady.

*Case* 1. A man, aged 55, had long been afflicted with strictures of the urethra. On the 4th February, 1831, he was admitted into the hospital. A constant muco-purulent discharge from the canal—bougies were employed, and the strictures dilated—still the discharge remained. The Venice turpentine was given inwardly, and produced very beneficial effects on this vesical catarrh. On the 24th of April he complained of heat in his throat, slight difficulty of swallowing and other characters of angina. The symptoms became alarming—great dyspnœa; pain in the larynx, and perfect inability to swallow. By very active depletion the inflammation was subdued; but a relapse most unexpectedly took place, in the course of two or three days; and so rapid was the onset, that on the evening of the attack, his life was despaired of, the respiration being performed with extreme difficulty and accompanied with a slight tracheal râle; the sputa viscid and purulent, and expectorated with great distress; no pleuritic pain. Nevertheless auscultation and the ensemble of the symptoms proclaimed the existence of a serious pneumonia. Patient bled from both arms; and 60 leeches to be applied to the side—died an hour after (! !) Several hours before his decease, the patient had experienced rheumatic pains in the right knee, which was tender on pressure.

*Dissection.* Slight traces of inflammation in the pharynx and larynx. The left pleuræ adhere by cellular bands. The lung is congested as in asphyxia. The right pleuræ present some recent albuminous patches, and, in their cavity, a purulent effusion to the amount of seven or eight ounces. The lung, towards its base, crepitates; but, in other parts, is gorged, and in a state of congestion.

*Reflections.* It is interesting to trace the events of the above case in their sequency. First a vesical catarrh—this is stopped; then symptoms of general fever supervene—angina and laryngitis ensue, and these give way to a fatal inflammation of the chest. What occasioned this succession of phlegmasiæ, in a constitution by no means plethoric? We can only answer, some inexplicable disorder of the constitution; but in what consisting, or on what depending, we cannot tell. This obscure and indistinct form of pulmonic inflammation is not very uncommon after severe wounds or injuries, when complicated with extensive suppuration. The attack is so insidious, that it has often made alarming progress before the existence of the disease is suspected. It is generally fatal in its issue; and the morbid appearances found on dissection differ considerably from the pathology of ordinary pneumonia and pleuritis. We do not recognize the different structural changes successively developed in, and denoting the progress of, the different stages of the disease; but, instead of this regularity, we find all these changes occupying confusedly, here and there, a lobe of the lungs, which thus acquire a sort of mosaic appearance, from the apposition of party-coloured spots or patches.

*Case* 2. A coachman, by a fall, received a severe contused wound of the leg. After suppuration had been established for some time, the discharge ceased for two days. The patient felt himself indisposed, but had no cough, nor expectoration, nor pain; nothing, in short, to lead any one to suspect serious pulmonary disease. In a day or so, copious sputa announced the presence of pneumonia in its third stage; but, in spite of the remedies used, the patient died, and dissection displayed one of the lungs in the morbid state described above, throughout its entire extent. There was also an abscess in the liver.

*Case* 3. A. L. aged 37, pale and emaciated; can lie only upon her back; pulse small, contracted, and frequent; constant cough, with expectoration of sputa, such as are observed during the resolution of an attack of pneumonia; diarrhœa, and distressing sleeplessness. The symptoms, after a time, became suddenly much aggravated, and threatened immediate dissolution: the respiration was panting and frequent—pulse rapid and full—skin hot—expectoration more abundant, and streaked with blood — diarrhœea increased; death soon followed. The patient had been ill of an obscure pectoral complaint, for upwards of a month before the history of the case begins.

*Dissection.* A large effusion of a purulent serum in the left cavity of the pleura. The lung of this side was much wasted, and adhered to the pleura costalis by strong false membranes. The superior lobe of the right lung exhibited numerous tubercles; the middle one was hepatized in different parts, and the remaining portion, although crepitating when compressed, was infiltrated with a frothy reddish serum. A well-formed pus oozed from some of the veins of the lungs, especially in the hepatized patches; and in other parts, the veins were found choked up with coagulable lymph. It is an interesting subject for reflection, whether the inflammation of the veins, which had given rise to the deposition of pus and fibrine in their canals, was caused primarily by the absorption of the purulent effusion in the pleuræ; if so, the present case is approximated, in a most important feature, to those already narrated in illustration of the form of pneumonic inflammation supervening during the process of suppuration.

### XII.

### FEEBLE AND INJUDICIOUS TREATMENT OF TYPHUS FEVER, BY THE FRENCH PHYSICIANS.

It is very generally admitted that the French are most excellent pathologists and physiologists, but inert and unsuccessful practitioners, especially in medical cases. We do not pass this censure from the details of a single case, as this would be unjust and uncandid, but from a review of their general practice. The following example will illustrate the truth of our remark.

B. C. aged 17, was seized suddenly and severely with headach and febrile irritation, on the 24th December, 1831. She felt as if beaten all over, had no appetite, but much thirst, general restlessness, and tendency to delirium. The symptoms between the above date and the 11th of January, 1832, are not narrated; at this latter period, her face had a pale and earthy aspect—she lay constantly on her back—did not answer any questions put to her—skin warm and dry—pulse small, but not frequent—respiration short—tongue red at the edges, and covered with a grey coating in the middle—teeth covered with a dark slime—urine scanty and high-coloured—pain in the right ilium when firmly pressed upon.

*Treatment.* Low diet and sulphuric lemonade!!!

On the following day, it was found that the patient had passed a restless night, being frequently in a high state of delirium; the face twisted to the left side, which made the Doctor suspect hemiplegia; but he found (simple man!) that the grin disappeared if he turned his patient a little round in bed. When her arm was pinched, she withdrew it, and grumbled some inarticulate sounds of suffering. This last circumstance (we are told!) led to the important discovery that, when questions were addressed to her, they were unanswered, though she appeared to understand them, especially if bawled sufficiently loudly, and if the brain, at the same time, was worked up to a sufficient pitch by pinching her arm!! The stupor had increased, the pupils were dilated, the head warm, skin dry, pulse soft and small, pain in the iliac region worse, urine and fæces passed involuntarily.

*Treatment.* The sulphuric lemonade, a blister to each thigh, and ice applied to the head!!

On the morrow she was considered somewhat better—tongue less dry—stupor not so great—the pulse had risen, and the skin was perspiring—pain of the side gone—occasional convulsive twitches of the muscles of the face.

*Treatment.* Limonad. sulphuric. lav. camphré.

Next day the report was, that she had been delirious all the previous night, and now there was great prostration of strength, insensibility, &c.

A blister to the nape of the neck, and ice to the head.

In the afternoon the breathing became so embarrassed as to threaten suffocation. She was bled from the jugular, and died immediately after!!

*Dissection.* No morbid appearance discernible in any of the abdominal viscera, beyond venous congestion of the liver and spleen. The lungs were, throughout almost their whole extent, lienised, or converted into a structure like that of the spleen; when cut into, much black blood flowed out. The venæ cavæ and pulmonary veins gorged with an inky, fluid blood. The veins of the brain were similarly congested.

Our readers will doubtless agree with us, in condemning the imbecile plan of treatment pursued in this case. The patient ought to have been bled at least locally; have taken mercury inwardly at first, and, towards the termination, wine and nourishment.—*Journal Hebdom.*

---

### XIII.

### ON THE USE OF LARGE FLAT LIGATURES IN CASES OF ANEURISM, &c.

It appears that M. Roux, one of the most successful operators in Paris, adheres to the old practice of employing flat, tape-like ligatures, and usually interposes a roll of plaster between the vessel and ligature. In upwards of 40

cases under his care, secondary hæmorrhage occurred only in four of them; and of these four, it was permanently arrested in two by the application of another similar ligature. Greater success than this has not, we think, been obtained by surgeons who employ the small round ligatures.—*Journ. Heb.*

---

XIV.

Resistance of the Official Mandate to disclose the Names and Abodes of Patients, who had received Wounds in the late Disturbances in Paris, and who were under Treatment, by the Surgeons who attended them.

The following sensible letter was addressed to the Academy of Medicine by M. Desportes; we shall transfer it to our pages, as a record of the public spirit of our French brethren, and as a worthy pattern to be copied on similar occasions.

Gentlemen,—The Government has more than once consulted you on questions which concern the practice of medicine, and you have always displayed a readiness and zeal in explaining the relations between the laws which regulate the profession of the healing art, and the general spirit of our national legislation, and, consequently, the connexion, in certain points, which subsists between the legislation, and the customs and manners of the people; for surely all government, to be good and permanent, must be in accordance with the ideas, feelings, and interests of those whom it rules. It belongs to you, at present, to declare the painful regret you experience, on the subject of the edict lately issued by one of the magistrates of the Department of the Seine, an edict upon which I shall not comment, as it can be viewed only as the offspring of a strange error, which will no doubt be expunged whenever it is exposed by you. It is not my intention to examine whether this disgraceful mandate is conformable to the principles of the existing legislation, and to the decision pronounced in last session by the Chamber of Deputies, wherein it is enacted, that non-disclosure shall no longer be considered as an offence or crime; or whether, in point of justice, the ordinance of 1666 and that of 1806 can be put in force, when the article 378 of the penal code subsists, and is later in date than these; or, lastly, whether it is well calculated to obtain any great political advantages. What I shall chiefly insist upon, is the flagitious immorality of the action required of professional men, and that, too, under pain of a penalty. As all political discussion is, and ought to be, interdicted within your walls, so our every action and every word must strictly accord with the rules of a pure morality, if we hope to benefit either science or humanity, our fellow-citizens or our individual characters. Oh! shameful outrage to the medical profession, to call upon us to reveal secrets as sacredly binding as any confessed to the minister of religion. Now a priest, if he ever abuses the confidence reposed in him, is for ever branded with infamy; and where is the difference between the duties of a priest and those of a physician? both have a public, official, and moral character to sustain, and without which the most imposing and useful attributes of their profession are often nullified. Both the one and the other are considered as "exceptional professions," in the article 378 of the penal code. By this article, medical men are received in the same light with the parents, brothers, and sisters of their patients; and thus it not only relieves them from all obligation of disclosing, but denounces punishment if they lose sight of those sacred ties which bind them to all such as are entrusted to their professional care. I, therefore, propose that a commission should be appointed, to examine into all the questions which may spring from, and are connected with, the above very important topic; and to decide whether the respect due to public morality, and the fulfilment of our duties towards our fellow-citizens, are compatible with the edict which has demanded so unworthy a compliance.—*Journ. Hebdom.*

---

XV.

### ON AN EPIDEMIC MILIARY SWEATING FEVER, WHICH RAGED IN THE DEPARTMENT of the OISE.

THE following is the account detailed by Dr. Menière.

"On the 9th of May, the Minister of Commerce and of Public Works was informed by the authorities of the department of the Oise, that a number of the districts were afflicted with an epidemic disease; and they requested that physicians might be sent to investigate its nature. The intelligence and petition were transmitted the same day to the Dean of the Faculty of Medicine. On the 10th Drs. Orfila, Pinel-Granchamp, Hourmann, and Menière set out for Beauvais, where the physicians and magistrates gave the requisite information. The disease proved to be the Miliary, or Picardy sweating fever, which had been observed in these localities several times before, and remarkably so in 1821. It broke out in the last week of April, or first of May, and was very mild, as, out of 84 patients, none had hitherto died. Its progress was noticed to be much arrested by cold weather. In some places its invasion was quite sudden after a storm, during which the thermometer rose considerably; and more than one half of the inhabitants of a village were thus seized in the course of one night. Females were more disposed to it than males. The disease commences with headache, sweating, and dispnœa; the skin then becomes red and very hot, and there is a most troublesome pricking sensation in the surface. No local pain; pulse full and soft. The dyspnœa appears to depend on a congestion of blood in the lungs and heart, and not on any weakness of any of the exterior respiratory organs: the plethora of the heart and large vessels is indicated by the ventricular pulsations being diffused, heavy, and slow, with little sound on auscultation. There is a distressing feeling of a choking weight in the præcordial region, and sometimes also in the epigastrium; the pulsations of the cœliac artery are so strong, as to heave up the abdominal parietes, and cause most obstinate gastralgia. The sweating is often prodigiously great, patients being obliged to change their shirts 20 or 30 times in the course of one night, and this "flux" continuing to the like extent for two or three days. Its odour was that of rotten straw, or of a weak solution of chlorine, or of the evacuations of the cholera morbus. The watery halitus from the skin is kept rarefied by the heat of the bed-clothes; when these are removed it is condensed, and forms a thick cloud, which speedily resolves itself into a sort of rain. The bowels are usually constipated, and the urine is scanty. The sweating lasts sometimes for four, five and six days, and it ceases gradually without the substitution of any other critical evacuation, or the occurrence of other symptoms; but in the majority of cases, a vesicular eruption on the chest, neck, back, and successively over all the body, appears on the second, third, or fourth day. It varies exceedingly in different patients: the vesicles are usually of the size of millet seeds—here and there a few larger ones are scattered. In the early stage the rash appears papular; when it dies away, the part is found covered with furfuraceous scales. The average duration of the fever, from its onset to the recovery of the patient, is from 8 to 14 days. The symptoms demanding most attention are those of congestion in the head and chest, threatening, in the one case, delirium and apoplexy—in the other, hæmoptysis and fatal dyspnœa.

The miliary epidemic of this year differs, in several important respects, from former eruptions of the disease; and it seems to have received a certain stamp from its more formidable brother, the cholera morbus, which exists at the same time in the same villages and districts. In the stead of the thoracic and cerebral symptoms, diarrhœa, vomiting, and gastro-enteritic irritation have frequently taken their place, and have carried off many of those affected with them. At Noailles, the chief feature of the epidemic was the cerebral congestion; while, at Cauvigny, only one league distant, it was congestion of the lungs, causing death sometimes in the space of a few hours.

There was no obvious difference in the topography of these two places to explain this diversity of character. In 1821, the disease raged severely at Cauvigny; there were 23 deaths, most of which were caused by the slow development of gastro-enteritic disease: in the present epidemic, death takes place at the very onset of the disease, or at the time of the miliary eruption, the patients suffering dreadful anxiety and distress at the præcordial region—racking pains and spasmodic stiffness of the back: sometimes they vomit, or rather hawk up, much frothy blood. At Chateau Rouge, many cases of what the French denominate "la suette exquise," that is, the sweating fever without any miliary eruption, were seen. In several patients, who were in a state of convalescence from the epidemic, symptoms of typhoid fever, such as stupor, emaciation, red patches on the belly, pulse frequent and soft, tongue red, and rough in the middle and towards the base, breath fetid, &c. supervened. From comparing the reports from 24 different districts in Picardy, it appears that, in the space of two or three days, not fewer than 5000 persons were seized with this epidemic disease. Many opinions have been offered to account for the frequent appearance of the miliary sweating fever in ancient Picardy; the miasmata of marshes and bad nourishment have been chiefly insisted upon, but we think incorrectly, as most of the patients were living in healthy dry localities, well housed, fed, and clothed, and frequently in affluence and ease. With regard to the occasional or exciting cause, there is little discrepancy of opinion: the elevation of temperature, and an electrical state of the atmosphere, have generally preceded the appearance of the malady. The miliary epidemic is fully as capricious in its course and career as the cholera; places being spared in the very line of its advance. It is certainly not contagious, as announced by the older authors.

*Treatment.* Formerly, when the disease was considered to proceed from a leaven, or poison, contaminating the blood, the efforts of the physician were directed to favour the expulsion of the peccant matter by sudorifics, cordials, warm ptisans and heavy bedclothes. Boyer, Tessier and others about the year 1773, introduced quite an opposite plan of cure. Venesection at the onset of the malady, mild tepid drinks, small doses of hypnotic medicines, and the very gentlest revulsive applications to the feet and hands, such as bathing them with mustard water are most proper; in short, whatever encourages the breaking out of the rash, which generally is accompanied with relief. Mild aperients are always useful. When symptoms of congestion in the lungs, or head occur, more active depletion and counter-irritation must be immediately adopted. We must be on our guard however not to lower our patient too much, as there is a strong tendency in this disease to nervous collapse. Whenever the eruption appears, the treatment ought to be limited to the mere regulation of the diet, &c. Many patients who have been bled at this period for dyspnœa, have rapidly sunk. The greatest care is necessary during the convalescence to prevent a relapse; every excitant, or irregularity must be studiously avoided.—*Archives Générales de Médecine.*

---

### XVI.

### Anatomy and Physiology of the Optic Nerves.

M. Ozanam of Lyons has lately directed his attention zealously to this subject, and the following is the result of his labours.

The optic nerves arise from the centre of the *nates*, by a minute white band, consisting of numerous fine fibrillæ, which wind round the anterior border of theoptic thalami, and join together at the corpus geniculatum externum. They seem to be blended, and incorporated at the point of junction, but we shall find that they are kept distinct from each other by a very thin membrane, which is a production of the dura mater; this forms a sort of pouch or bag full of a medullary substance of the consistence of cream. This arrangement may be most easily seen by extending a portion of the optic nerves on a tablet of polished dark wood, and then cautiously separating the junction with a blunt blade.

In order to arrive at a greater exactness, M. Ozanam placed between two plates of glass in a solar microscope, the optic nerves of a person who had just died of encephalitis; the nerves were detached three lines before their junction, and six lines beyond this point. The image thus formed was so magnified, as to appear six feet long, and three feet broad, thus occupying an area of 18 square feet; each nerve seemed to be eight inches round, and the membrane, or interposed pouch two feet across. If these observations be correct, it follows that the optic nerves do not decussate each other where they meet; we may suppose however that there is at their origin a mediate communication between them by the cerebral pulp, and by the nutritious bloodvessels. A similar connexion may exist at the point of junction; for it is highly probable that the pouch and contained fluid may serve such a purpose. —*Archiv. Gener.*

---

### XVII.

### Official Report on Cholera, by the Royal Academy of Medicine of France.

The Royal Academy of Medicine has been called upon a second time by government to report their opinion on this frightful scourge. The first report was occupied with a summary of the documents which had been transmitted to them from foreign countries where the disease was raging; but since that time, it has reached our own shores, and entered our own habitations; and the details which are now presented to the public are drawn from direct experience. It was between the 22nd and 26th of March, that the cholera broke out at Paris. Before this period indeed some isolated occurrences, and doubtful cases had been noticed in the metropolis; but neither the towns, nor villages situated on the frontiers of countries then infected, had afforded any unequivocal cases of the epidemic. The disease suddenly encreased to an alarming extent in a quarter of Paris which had less communication with strangers than with any other; and situated at a distance from the post-houses, inns, or even the streets, where strangers, goods, and provisions arrived from such countries, as were at the time suffering from it. At first those classes which were ill clothed, fed and housed, and addicted to debauchery and excesses, were chiefly the victims; and it was observed at that time to attack the different inmates of a dwelling sometimes simultaneously, at other times, one after the other. As a general fact, however, it may be stated that in the greater number of instances, at least among the more respectable society, one only of a family was seized. The members of the medical profession, though much more exposed to its influence than any other individuals, have certainly not suffered in a greater proportion than the rest of the population. The same observation is applicable to those who immediately attended on the patients, as nurses, servants of hospitals, relations, and clergymen. A statistical account will be published at a future period under the authority of government. The disease, on its first appearance, displayed its full intensity and virulence; but soon various forms and modifications were observed. Its attack was sometimes instantaneous; at other times preceded by premonitory symptoms.

A large proportion of the inhabitants have experienced more, or less, what may be termed the influence of the epidemic, characterized by weariness of the limbs, sleeplessness, heaviness in the head, lowness of spirits, loss of appetite, constipation, and scantiness of urine.

The disease was ushered in, sometimes with headach, or cramps of the extremities; at other times with vomiting; but most frequently the diarrhœa was the earliest warning; and if the attack was not suddenly alarming, these symptoms continued for several hours, or even days. Then followed the prostration of strength, feeling of weight, anguish and heat in the epigastrium, extending from the precordial region to the throat; nausea, borborygmi, dryness of the mouth, purging, and great feebleness of the pulse. The alvine evacuations were sometimes bloody; of a yellow, green, or even brown colour, and mixed with a white mucous discharge, but generally they had all the appearance of thickish rice-water, and were squirted out as if by the jet of a syringe. The blood drawn was black, coagulated, and pitchy; when allowed to rest, very little serum separated; and it was only rarely that slight traces of any buffiness were observed on the clot. The form of the disease now described has been by some very improperly designated cholerina; it must be regarded as cholera of the mildest degree, and occurs only under favorable circumstances. When more concentrated, and malignant, the disease exhibits the two formidable phases, viz. the cold stage; and the stage of reaction. When death took place in the cold stage, it was not unfrequent to observe the cessation of all vomiting and purging, and the patients to state that they felt better, when they had only a few moments to live. The transition from the cold stage to that of reaction, was too frequently chequered with repeated alternations of heat and of coldness, quickly succeeding to each other. In a few rare cases the cholera commenced with all the symptoms of the stage of reaction. The severity of the one stage appeared to have no influence upon or connexion with that of the other; sometimes the cases of most appalling iciness recovered without any subsequent febrile symptoms; and many patients died during the stage of reaction, although the cold stage had been short and imperfect. During convalescence, the slightest error of diet, trifling fatigue, exposure to cold, or damp, and mental emotions, often induced a fatal relapse. One attack of the disease did not necessarily secure the person from all danger of a second. In the course of the epidemic, several well authenticated instances of "cholera recidiva" have been reported. It even appears that by having once suffered from the agony of the epidemic, the system is more susceptible of its attacks, and more liable to relapses.

With respect to the pathology of cholera, it ought particularly to be noted in each case of dissection, what had been the character of the individual attack, and the duration of the disease, as upon these the extent and magnitude of the morbid changes are intimately dependent. In many of the formidably rapid examples, in which death had taken place, within three or four hours, scarcely any appreciable lesions were to be discovered. The corpses were generally of a purplish colour; the subcutaneous muscles formed prominent lines along the surface, the face and hands were much shrunk, and the fingers were strongly contracted. The pharynx was almost always normal; œsophagus generally so; but sometimes slightly red, and studded with enlarged mucous follicles. The stomach has in many cases been found not sensibly altered; commonly however, its mucous surface was highly red, either in patches or throughout its extent, and this appearance was accompanied occasionally with ramollissement of its texture. In the intestines a turbid white fluid with flocculi was found; and the mucous surface of the bowels was coated with a creamy substance. This coat was often highly inflamed and injected with red vessels, or patches were infiltrated with actual blood. In a large proportion of cases the glandulæ Brunneri, et Peyeri were magnified and very conspicuous, especially in the first convolution of the small gut, and also in the lower ones, near the termination of the ilium in the colon. The urinary bladder was invariably contracted and empty; its mucous surface was generally encrusted with a white softish layer, similar to that found in the intestines; and this appearance was sometimes traced in the ureters, and even in the pelvis of the kidney. The heart and large vessels gorged with black, half coagulated blood, which had much the aspect of currant jelly; and which had manifestly been deprived of much of its watery portion. All the serous membranes were unusually dry; most of the viscera were more or less injected with blood, and were of a blueish or black colour. Gall-bladder large and distended with thick bile. No morbid traces were discovered in the nerves, either of animal, or of organic life. The bones, in some cases, were found to be highly injected and had acquired the colour of the blood, as if the patient had died of ostitis.

The specific and essential cause of cholera is quite unknown; but numerous and well ascertained are the causes which favour its attack, and promote its virulence. Whatever enervates the body, as excesses of all kinds, insufficient, or unwholesome nutriment, vicissitudes of climate, and depressing mental emotions strongly predispose individuals to its influence. The disease successively invaded the different quarters of the metropolis; but what guided its march, and directed its onset, baffled all enquiries. It is not quite easy to explain how a regular, sober, and active mode of life proved so good a preservative; but so it was; for very few cases were seen in the numerous colleges, schools, religious and charitable establishments dispersed over the city.

*Treatment.* Hitherto no exclusive, or specific mode of treatment has been discovered. In the very commencement of the disease, at least in the young and plethoric, bleeding by the lancet and by leeches were of immense service; aromatic mucilaginous drinks, aerated, or acid water, lemonade, &c. were given; sometimes tepid baths, frictions on the surface, and mustard poultices according to the circumstances of the case. Emetics of ipecacuan were occasionally very useful in changing the character of the vomitings and purgings from the whitish and flocculent fluid, to true bilious evacuations, or causing them to stop altogether; in promoting perspiration and resuscitating the animal energies. In the cold stage, our efforts must be directed to restore the warmth and circulation, by hot baths, &c. Ice has been very successfully given at this period by some; others have preferred iced punch, strong wines, coffee, and tea. Blisters, sinapisms and embrocations have occasionally been of use, and the following mode of irritating and cauterising the skin has been recommended. Take a long piece of woollen stuff, of the length of the back, and six inches wide; soak it in a mixture of 8 parts of spirit of turpentine, and one part of liquor ammoniæ, and apply it along the spine; over this lay a linen cloth wrung out of hot water; and then pass along this a heated iron, which is to be made sufficiently hot to cause the fluids to be volatilised, and the cloths to be nearly dried. This operation must be repeated every hour, till a decided improvement in the patient's state has taken place. Venesection and bleeding by leeches in the extreme cold stage have been praised by some. Emetics have been also tried with some success.

In the third stage, or that of re-action, symptoms of typhus fever usually occurred; but varied in their character from high irritation and excitement, to great depression and debility. Cerebral, pulmonic, and abdominal congestions were not unfrequent, and had to be combated with depletion and counter-irritants. Ice kept applied to the head for six or eight hours, and warm simple opiate or stimulating poultices to the lower limbs were of obvious utility. During the convalescence from an attack of cholera, a tendency to diarrhœa often continued obstinately: to counteract this, leeches to the anus, rice drinks, decoction of rhatany root, and small doses of opium were best adapted. Enemata, consisting of the rhatany decoction, with, or without opium, were also employed with much advantage. Powdered vegetable charcoal in doses of ʒss every hour had good effects in restraining the diarrhœa, and in restoring it to a healthy bilious condition. To quiet the cardialgia and vomiting, nothing has been found so useful as ice, and cutaneous revulsive applications. The cramps were treated by bleeding, warm baths, opium, bismuth, anodyne frictions, a circular ligature of the limbs, &c.—*Archiv. Général.*

*Remarks on the preceding Report.* We have presented to our readers the protocol of the learned commission appointed by the Academy of Sciences, not because we consider it an important or valuable document, but because it ought to have been so, and we expected that it should be so. Surely the duty of a scientific body of men is something beyond the mere bulletinising and chronicling every varying and unsteady feature of a disease, describing every symptom, already so well known; detailing every mode of treatment however whimsical and inefficacious, registering every pathological appearance, and too often also inserting shallow conjectures and unsubstantial theories. Not one fact is added to our knowledge; nor one novel conclusion submitted to our reason. If it be answered that the report was drawn up for the guidance and instruction of the public in general, we hesitate not to say that in this point of view, it is yet more faulty; and not at all calculated to serve this end. No unprofessional person can possibly thread his way through a pamphlet of 20 closely printed pages, in which he finds himself bewildered among the numerous distinctions of the disease itself, puzzled with post-mortem appearances in facts about which he knows nothing in a state of health, and made disconsolate by being informed that no treatment is of almost any avail; in short if he comes to any conclusion, it must be that doctors everlastingly differ among themselves, and make experiments on their patients, rather from curiosity than from any rational, or well-grounded hopes of cure.

We have patiently, and with much fatigue examined almost every contribution on the subject of cholera in the French periodical press, in order that we might be able to communicate to our readers, all useful information on so interesting a subject; but hitherto we have laboured to little purpose; and nothing but the discovery of thrice-told tales, and of endless theories has been our reward.

It is gratifying to reflect that our own countrymen better deserve our praise and approval; their labours have certainly been more fruitful in the vineyard of enquiry, and may lead to still more satisfactory and agreeable results.

---

### XVIII.

### Memoir of the Last Illness and Death of Baron Cuvier.

Obs. The following account was drawn up by Dr. Rousseau who attended the deceased.

Cuvier was a man who had an astonishing aptitude for mental labour and exertion; his recreation indeed consisted in being busily employed; yet he was readily alarmed about any trifling indisposition, and although he put little trust in medical science, he used to consult every doctor he knew on the state of his health.

On Monday the 7th of May be experienced a little irregularity in his bowels, and was ordered to take an additional enema with 6 drops of laudanum in it. Next day he felt himself quite well; and delivered a lecture in the College of France, displaying unwonted energy and animation; and after lecture he walked home, a thing he seldom did. He dined as usual, and was present in the evening at the meeting of the professors of the museum. On the Wednesday he felt a pain in the right shoulder and arm; and some difficulty in closing the right hand; he attended however a state council, and on his return, was urgent for his dinner; but scarcely had he eaten some broth, before he experienced such a difficulty of swallowing any solid food, that he was obliged to make the attempt several times. His family became alarmed and sent for his physician Dr. Allard, who prescribed 20 leeches to the anus. On the following morning he could not move his right arm, and was obliged to rest it in a sling. The pulse was 80 to

85, the deglutition still difficult, and there was some headach. He was freely bled; had a mustard bath to the feet, and a blister to the nape of the neck; he felt very weak, and his voice became sensibly changed; he was restless during the night; and as the pulse again rose in fullness and strength, a second bleeding from the arm was performed. From this time the muscular energies were much impaired; but the heat of the body, nervous sensibility, and intellectual powers were remarkably entire. On the Friday an emetic was given, as the patient had not apparently derived benefit from the venesection: but it did not produce vomiting, but slightly purged the bowels. At two o'clock M. Dupuytren introduced by means of an œsophageal tube and syringe 24 grains of ipecacuan mixed in a little warm water; still no vomiting followed. At five o'clock 48 grains were injected into the stomach, without causing even any nausea. At seven o'clock a saline enema was administered, which brought on an extreme purging of dark-coloured bilious stools. The right arm was quite powerless; deglutition impossible. Two blisters were applied to the side of the neck in the evening, over the site of the cervical plexuses. The night was passed in an agitated and restless state. On Saturday morning, there was observed a palsy of the movements of the left leg, in addition to the previously mentioned symptoms; the hands became very cold, and the nails acquired a blue colour; the pulse was smaller and weaker than hitherto. Twenty leeches were applied over the mastoid process; in the night, the patient suffered great distress from not being able to move himself at all in bed—and on the Sunday, his appearance seemed as if he had become ten years older; his voice was more changed; still he was calm and complained but little. Cupping glasses were applied below the kidneys, and some orangeade was injected into the stomach; at eight o'cloek in the evening he conversed with Dr. Allard; he was again cupped, and was extremely fatigued by the operation. At nine o'clock he said that the faculties of his mind were leaving him, and a quarter before ten he died sitting upright in his arm-chair. The head was measured, and round ths greatest circumference was 22 inches, 4 lines. From the occipital fossa, along the vertex to the roof of the nose, it was 13 inches and 4 lines; from one meatus auditorius to the other, 15 inches. The facial angle was very large. The spinal canal was most cautiously opened, and the marrow and its membranes examined; but no trace of disease found. The cranium was of very ample dimensions. The cerebral convolutions were very numerous, and the furrows between them very deep; on many of the convolutions were observed mamillary protuberances, which formed an integral part of their substance. Each portion was most attentively inspected, and layer after layer examined; but all was found healthy. The lateral ventricles were very large, but contained little serum. The left choroid plexus presented two small cysts full of a transparent fluid, and very like in appearance to two granules of a white currant. The mass of the cerebrum weighed 3 pounds, 11 ounces, 4 drachms and a half; the cerebellum, when detached from this mass weighed 6 ounces and 1 drachm. The pharynx and œsophagus were perfectly healthy; the brachial nerves equally so. The processus dentatus of the second cervical vertebra was very large; and the third cervical, as well as several of the dorsal vertebræ had osseous excrescences on their anterior aspects, where one bone was united to another. This occurrence explains the great difficulty which Cuvier experienced in stooping forward to collect any things lying on the ground. The thoracic viscera were healthy; much fat was found in the mediastinum. In short, no obvious morbid change could be discovered in any part of the body; hence some have attributed the death of Cuvier to the epidemic influence of the cholera; but this is only a gratuitous conjecture, and not confirmed by a single symptom during life.

The character of this great man has been thus portrayed by an eminent writer. Cuvier was gifted with a mighty and capacious mind, rather than with any striking genius. His intellectual powers, were rather of a critical than of a didactic character; and as is the case with most encyclopediacal heads, he had a leaning to scepticism. His mind was clear, rapid, rigorously exact, but not sufficiently bold; and perhaps we may account for this deficiency, by calling to mind that the requisite mental hardihood is generally intimately associated with a resolute firmness of character, which Cuvier to a certain extent did not possess. In his lectures, when conveying instruction, and elucidating any subject, there was a spirit of modest circumspection, and a fondness for demonstrative proof, which belonged as much to the original stamp of his character, as to the philosophical genius of the present age in France. An unwearied, and most keen observer, gifted with a prodigious memory, and a capacity for mental labour, rarely possessed. He has left behind him numerous important works. Of universal knowledge, Cuvier knew every subject sufficiently well, to render his description or illustration of individual examples more perfect and complete. As a writer, he has little grandeur, or originality; but his works bear the impress of great thought:—his style is perspicuous, elegant, and correct. He describes things, not as a painter, but as an anatomist; he argues with ability, but his reasonings are stiffly and coldly expounded; he narrates, as a chronicler, rather than as a philosophical historian. His mind wanted the aid and influence of a mighty inward impulse, to inspire him with that passionate enthusiasm for the study of Nature, which in other language, is merely the thirst after Truth; it was his exquisite taste, and a natural aptitude for the sciences which he especially cultivated, which led him on to that proud distinction above all his cotemporaries.

Cuvier was born on the 25th of August, 1769, at Montbeliard, and consequently at his death was 63 years old.—*Archiv. Génér.*

---

### XIX.

Remarkable Emphysema of the Lung. Examination of Mr. M——y, July 3, 1832.

Present Mr. Thomas, Dr. James Johnson, Mr. Henry Johnson, and another Medical Gentleman.

Upper extremities and upper part of trunk attenuated—subcutaneous cellular membrane of lower part of trunk and of lower extremities anasarcous.

*Thorax.* On removing the sternum and cartilages of the ribs the right lung appeared extremely prominent, and did not collapse in the slightest degree. The left lung, though not so distended as the right, shewed no tendency to collapse when the chest was opened. On examination the lungs presented a very extraordinary specimen of emphysema, which was much more developed in the right than in the left. The whole lung was of great dimensions, nodulated as it were, and irregular, but light and in many parts elastic. The emphysematous appearance was chiefly noticed at the free margins of the lobes, which were rounded off, turgid, and globular in shape, from the extravasation of air into the cellular texture of the organ. The appearance presented was not a mere string of air vesicles, which are not unfrequently met with in the examination of dead bodies, but a continuous emphysema of extent and magnitude. Attached to the lower lobe was a sort of distinct cyst, much larger than an egg, perfectly pellucid, and containing air. The wall of the cyst appeared to be formed of the pulmonary pleura. The whole substance of the lung was evidently more or less emphysematous, except in parts where induration was felt, occasioned probably by hepatization. The left lung presented similar appearances, but in a less marked degree. There was some serous fluid in the right side of the chest. As the lung was removed by Mr. Thomas, in order that it might be preserved in the Museum of the College of Surgeons, no section of it was made, for the purpose of examining its structure more minutely.

The heart was much enlarged. The right auricle was dilated and somewhat hypertrophied—the right ventricle dilated, without much hypertrophy—the ostium venosum of large dimensions. The left auricle was dilated—the left ventricle dilated and hypertrophied. The semilunar valves of the aorta presented some ossification at their attached margins, but the alteration was not such as to prevent them from executing their proper functions. Perhaps they might have offered some little obstruction to the exit of the blood from the ventricle. The aorta was generally flabby, and somewhat dilated.

*Abdomen.* The liver was large, hard, dark-coloured; it descended low in the abdomen and presented a very decided margin. No other appearance of consequence presented itself.

*Cranium.* Not examined.

*Remarkable state of Lung.* The patient, whose dissection is appended, was a stationer in Tottenham-court-road, who had laboured for twenty years and more under what is usually termed asthma. He did not come under the observation of Dr. Johnson till within a few months of his death, when he was dropsical, in trunk, abdomen, and extremities. The breathing was so laborious that he could not lie down at all, and he was harassed with constant cough. On examination the heart was found to be greatly enlarged. It was difficult to say what was the actual condition of the lungs. Sy some brisk doses of elaterium, with extract of colocynth and the pilula hydrargyri, the dropsical swellings disappeared—the breathing became free—and he was able to go out to Hampstead and other places daily for air. But the state of the heart continued the same. After many fluctuations, the dropsical effusions returned—the medicines lost their power, and he died in a slow and gradual manner, leaving an express wish that his body should be examined by Mr. Thomas and Dr. Johnson.

---

### XX.

### ON THE STRUCTURE OF THE HUMAN PLACENTA, AND ITS CONNEXION WITH THE UTERUS. By DR. ROBERT LEE.

[*From the Philosophical Transactions.*]

THIS indefatigable and able physician pursues his physiological and practical labours with assiduity and success. The structure and connexion of the placenta have puzzled most of our anatomists, up to the present hour. So far back as 1780, John Hunter presented a paper to the Royal Society, claiming the discovery of ascertaining the true structure of the placenta. The following is the history of the appearances which he observed in the dissection of a woman who had died undelivered near the full term of utero-gestation, and on which his conclusions respecting the structure of the organ were founded.

"The veins and arteries of the uterus having been injected, an incision was made through the parietes, at the anterior part where the placenta adhered to the internal surface. Between the uterus and placenta lay an irregular mass of injected matter, and from this mass regular pieces of the wax passed obliquely between it and the uterus, which broke off, leaving part attached to that mass; and on attentively examining the portions towards the uterus, they plainly appeared to be a continuation of the veins passing from it to this substance, which proved to be the placenta. Other vessels, about the size of a crow-quill, were seen passing in the same manner, although not so obliquely. These also broke on separating the placenta and uterus, leaving a small portion on the surface of the placenta; and on examination they were discovered to be continuations of the arteries of the uterus. The veins were next traced into the substance of what appeared placenta; but these soon lost the regularity of vessels, by terminating at once upon the surface of the placenta, in a very fine spongy substance, the interstices of which were filled with yellow injected matter. He then examined the arteries; and tracing them in the same manner towards the placenta, found that, having made a twisted or close spiral turn upon themselves, they were lost on its surface.

On cutting into the placenta, he discovered in many places of its substance yellow injection, and in others red, and in many others these two colours mixed. The substance of the placenta, now filled with injection, had nothing of the vascular appearance nor that of extravasation, but had a regularity in its form which showed it to be naturally of a cellular structure, fitted to be a reservoir for blood.

From these appearances Mr. Hunter infers, 'that the arteries which are not immediately employed in conveying nourishment to the uterus go on towards the placenta, and proceeding obliquely between it and the uterus, pass through the decidua without ramifying. Just before entering the placenta, after making two or three close spiral turns upon themselves, they open at once into its spongy substance, without any diminution of size and without passing behind the surface as above described.

'The veins of the uterus appropriated to bring back the blood from the placenta, commence from this spongy substance by such wide beginnings, as are more than equal to the size of the veins themselves. These veins pass obliquely through the decidua to the uterus, enter its substance obliquely, and immediately communicate with the proper veins of the uterus. This structure of parts points at once to the nature of the blood's motion in the placenta. The blood detached from the common circulation of the mother moves through the placenta of the fœtus, and is then returned back into the course of the circulation of the mother to pass on to the heart.'

Dr. W. Hunter's description of the vascular connexion between the uterus and placenta coincides with that of his brother.

'For it seems incontestable (he observes) that the human placenta, like that of the quadruped, is composed of two distinct parts, though blended together; viz. an umbilical which may be considered as a part of the fœtus, and an uterine which belongs to the mother; that each of these parts has its peculiar system of arteries and veins, and its peculiar circulation, receiving blood by its arteries and returning it by its veins; that the circulation through these two parts of the placenta differs in the following manner:—in the umbilical portion the arteries terminate in the veins by a continuity of canal, whereas in the uterine portion there are intermediate cells, into which the veins begin.'

These two brothers came to a quarrel respecting the priority of this discovery! Their doctrines or facts have not been called in question for the last 40 years. Nevertheless our author proposes in this paper to describe certain appearances which he has observed during the examination of six gravid uteri; and many placenta expelled in natural labour, which seem to "demonstrate that a cellular structure does not exist in the placenta, and that there is no connexion between this organ and the uterus by great arteries and veins."

"If an incision be made through the parietes of the gravid uterus, where the placenta does not adhere, the membrana decidua will be observed lining the internal surface, and numerous minute blood-vessels and fibres passing from the inner membrane of the uterus to the decidua. At the circumference of the placenta, the membrana decidua separates from the chorion and amnion to pass between the uterus and placenta, and thus forms a complete membranous septum, which is interposed betwixt these organs. The chorion and amnion cover the fœtal surface of the placenta; and between these two membranes and the decidua lie the ramifications of the umbilical vein, and arteries subdivided to an almost indefinite extent, and connected together by white slender filaments running in various directions. The placenta thus consists solely of a congeries of the umbilical vessels, covered on the fœtal surface by the chorion and amnion, and on the uterine surface by the deciduous membrane, and inclosed between these membranes; it adheres to the fundus, or some part of the uterus by innumerable flocculent fibres and vessels.

On detaching the placenta carefully from the uterus, the deciduous membrane is found to adhere so closely to the umbilical vessels which it covers, that it is impossible to remove it without tearing these vessels. With the fibres uniting the placental decidua to the uterus are mingled numerous small blood-vessels, proceeding from the inner membrane of the uterus to the decidua; and these vessels, though more numerous at the connexion of the placenta with the uterus, exist universally throughout the whole extent of the membrane. There is no vestige of the passage of any great blood-vessel, either artery or vein, through the intervening decidua, from the uterus to the placenta; nor has the appearance of the orifice of a vessel been discovered, even with the help of a magnifier, on the uterine surface of the placenta. This surface of the placenta deprived of the deciduous membrane presents a mass of floating vessels, its texture being extremely soft and easily torn; and no cells are discernible in its structure, by the minutest examination.

At that part of the surface of the uterus to which the placenta has been adherent, there are observable a great number of openings leading obliquely through the inner membrane of the uterus, and large enough to admit the point of the little finger: their edges are perfectly smooth, and present not the slightest appearance of having been lacerated by the removal of the placenta. In some places they have a semilunar or elliptical form, and in others they resemble a double valvular aperture. Over these openings in the inner membrane of the uterus, the placenta, covered by deciduous membrane, is directly applied, and closes them in such a manner that the maternal blood, as it flows in the uterine sinuses, cannot possibly escape either into the cavity of the uterus, or into the substance of the placenta. The above appearances on the inner surface of the uterus have been accurately represented by RŒDERER; from whose work fig. 1. of Plate I. is taken.

When air is forcibly thrown either into the spermatic arteries or veins, the whole inner membrane of the uterus is raised by it; but none of the air passes across the deciduous membrane into the placenta, nor does it escape from the semilunar openings in the inner membrane of the uterus, until the attachment of the deciduous membrane to the uterus is destroyed. There are no openings in the deciduous membrane corresponding with these valvular apertures now described, in the internal membrane of the uterus. The uterine surface of the placenta is accurately represented in fig. 2. Plate I.

If a placenta be examined which has recently been separated from the uterus in natural labour, without any artificial force having been employed, its surface will be found uniformly smooth, and covered with the deciduous membrane; which could not be the case, did any large vessels connect it with the uterus. The placenta in a great majority of cases is also detached from the uterus after labour, with the least imaginable force; which would be impossible if a union by large blood-vessels, possessing the ordinary strength of arteries and veins, actually existed. Besides, a vascular connexion of such a kind would be likely to give rise, in every case, to dangerous hemorrhage subsequent to parturition, a circumstance not in accordance with daily experience."

The following observations are offered by our author as reasons why his predecessors have fallen into error in their anatomical researches.

"NOORTWYCH, RŒDERER, HALLER, Dr. W. and Mr. J. HUNTER, and Dr. DONALD MONRO, do not appear to have examined the gravid uterus and its contents in the natural state of the parts, but after fluids had been forcibly injected into the hypogastric and spermatic arteries. The laceration of the deciduous membrane covering the orifices of the uterine sinuses followed this artificial process, as well as the formation of deposits of injection in the vascular structure of the placenta, giving rise to the deceptive appearance of cells. That this took place in the examinations made by RŒDERER* and MONRO†, does

* Icones Uteri humani, Observationibus illustratæ. J. G. RŒDERER, 1759.

† Essays and Observations, Physical not admit of dispute; and the following facts render it more than probable that the HUNTERS were also misled, by the effects of artificial distension of the placenta, from the extravasation of the fluids forced into the uterine vessels.

In the course of last autumn, the preparations of the gravid uterus in the Hunterian Museum at Glasgow were examined at my request by Dr. NIMMO; and in none of them does it appear certain that any great blood-vessels pass from the uterus into cells in the placenta; but in many the deposits of injection, causing the appearance of cells, were observed evidently to be the result of extravasation. No preparation in the collection seems to have been expressly made for the purpose of proving or disproving the fact that the deciduous membrane passes over the uterine surface of the placenta; but in reference to preparation R. R. No. 139, it is observed by Dr. NIMMO that no vascular openings are visible in the membrane interposed between the uterus and placenta.

No. 178 'is a small section of the uterus with the veins injected green, and broken off where they were entering the placenta.' The surface of the injected matter is smooth; the edges of the openings defined and quite unlike ruptured vessels; their form in general elliptical, seeming as if they were holes cut in the side of a convolution.

No. 125. 'A portion of uterus and placenta, the latter injected from uterine vessels.' There is an opening which seems to be natural, corresponding to one of those in the uterus; but the majority of those whereby the injection has passed into the placenta seem to be mere lacerations.

No. 101. 'A section of uterus with veins injected black, and the injected matter protruding by irregular plugs into the cavity of the uterus.' The holes are semilunar and elliptical, with defined edges, and nothing resembling the continuation of vascular tubes to be seen.

R. R. 121, is described in the printed Catalogue as follows: 'A small portion of placenta and uterus, where the cells of the placenta have been injected from the veins of the uterus. The veins are seen very large, entering the substance of the placenta.'

Dr. NIMMO makes the following observations on this specimen: "This preparation seems to be most in point. I would describe it differently. The cellular substance of the placenta has certainly been filled from the uterine vessels. These, however, instead of passing directly into the placenta, are distinctly seen applying their open mouths to the membrane of the placenta, where the injection in some instances stops. The membrane is thinner here than where no vessels are applied, consisting, so to describe it, of one layer, while a second layer covers all other parts. Where the injection has passed into the substance of the placenta, it has evidently been forced to the side between the layers, and found some weak point, whereby it has entered into and been diffused throughout the cellular texture of the placenta.*'

From the foregoing facts, our author thinks he may safely conclude that the human placenta does not consist of two parts, maternal and fœtal—that no cells exist in its substance—and that there is no communication between the uterus and placenta by large arteries and veins. The whole blood sent to the uterus by the spermatic and hypogastric arteries, (except the small portion supplied to its parietes and to the membrana decidua by the inner membrane of the uterus) flows into the uterine veins or sinuses, and, after circulating through them, is returned into the general circulation of the mother by the spermatic and hypo-

---

and Literary, read before a Society in Edindurgh, 1754,. Vol. I.

* My friend SAMUEL BROUGHTON, Esq. F.R.S., during a recent visit to the Hunterian Museum at Glasgow, exexamined the preparations of the placenta and uterus at my request, and authorizes me to say that his observations fully confirm the accuracy of Dr. NIMMO's statements.

gastric veins, without entering the substance of the placenta.

"The deciduous membrane being interposed between the umbilical vessels and the uterus, whatever changes take place in the fœtal blood, must result from the indirect exposure of this fluid, as it circulates through the placenta, to the maternal blood flowing in the great uterine sinuses."

There is appended to the paper a valuable communication from Mr. Owen respecting the anatomy of the uterus and placenta, and the whole is illustrated by two expressive plates.

---

XXI.

Observations on various Diseases. By Robert J. Graves, M.D.

Under this head, Dr. G. has communicated, through the medium of our Dublin cotemporary (No. II.), some facts, elucidatory of the nature of diseases and the operation of remedies. The observations are practical, and not speculative.

1. *Ptyalism.* This was a case of profuse secretion of saliva, independent of mercury. The patient (a female) first had leucorrhœa, which suddenly ceased, and was succeeded by anasarca. This disappearing under proper remedies, was followed by gastric irritation; upon the disappearance of which a profuse ptyalism ensued, which resisted purgatives, tonics, gargles, and various other remedies.

"In twenty-four hours she spits more than a pint and a half of fluid, consisting of a whitish, viscid mucus, secreted by the mucous membrane of the fauces and back of the pharynx, from whence it is thrown into the mouth by a *hawking*, renewed every two or three minutes, with scarcely any interruption either during the night or day, and rendering the patient truly miserable from want of sleep. The throat and fauces are pale, and their soft parts extremely flabby and relaxed, although there is a constant irritation in the throat in consequence of the presence of an unnatural quantity of mucus, yet no soreness is felt, neither do the parts appear inflamed. The salivary glands are not concerned in the disease, and do not secrete more than the usual quantity of fluid. Her appetite is very bad, her skin dry, and she has a haggard, emaciated countenance.

The well-known good effects of opium in several diseases of increased secretion, diabetes, diarrhœa, and certain forms of dropsy, suggested to me the trial of this medicine in the apparently almost hopeless case I have related, and I accordingly ordered the patient one grain of opium every fourth hour. On the following day, she returned to inform me that she had slept during the whole night, and on awaking had no return of the spitting. Her joy was great, and she and her friends considered the effect of the pills, in thus suddenly stopping the spitting, as most extraordinary; and I must confess, that my surprise was almost equal to their's.

She then told me, that several medical students who lived in her house, and who had witnessed the previous violence and obstinacy of her complaint, had been so much struck by its sudden cessation under the influence of the pills, that she was commissioned by them to inquire what I ordered. I mention this circumstance, to shew how very remarkable was the benefit she received from the opium. The pills were continued for some days, when the quantity of opium was augmented on account of some recurrence of the spitting; unfortunately they induced constipation of bowels, and, consequently, she has been frequently obliged to leave them off, but she is, on the whole, much improved in health, and although she is still subject to the disease, its severity is comparatively trifling, and it uniformly disappears almost entirely when she recurs to the use of opium. She visited me this day, the 18th of March. In connexion with this subject I may observe, that a lady, a patient of mine, who took a large quantity of mercury many years ago, has been ever since subject to occasional returns of salivation, in *every* respect resembling that produced by mercury. During these fits, which are always brought on by exposure to cold, the mouth is sore; the gums red and swollen; the salivation copious, and the breath is strongly impregnated with the mercurial fetor. Several such cases have been recorded by others, and are extremely instructive, proving that the deleterious effects of mercury on the system may lie long dormant, and be afterwards suddenly called into action by various causes. In this way we may explain the attacks of periostitis, to which persons who have been mercurialized, are often liable for years, upon exposure to cold. Formerly such attacks were indiscriminately attributed to a remnant of the syphilitic taint, but I have witnessed the same occurrence in persons who had no syphilitic disease, and who had taken mercury for other complaints. One of the most violent attacks of periostitis I ever witnessed, followed salivation in a lady to whom mercury had been given several months before, for the cure of peritonitis. To conclude, I have seen many cases totally at variance with the assertion of Ballingall and others, that the secondary symptoms, attributed by recent authors to mercury, are owing to lues; not being observed, as they state, except in cases where that medium had been exhibited for the cure of the venereal disease. The venereal poison produces a peculiar train of primary and secondary symptoms. The mercurial another, and that not very dissimilar. Experience has proved, that in general the venereal symptoms yield to the action of mercury, but occasionally this is not the case, in consequence either of some constitutional peculiarity, or an injudicious use of this mineral, and thus the constitution labours under a modified disease, resulting from the combined effects of the two poisons. Attention to the effects of the remedy in the first instance, will generally prevent the occurrence of so disastrous a complication. My own experience has convinced me, that the majority of venereal cases may be cured by the non-mercurial plan of treatment; but whenever the disease, whether primary or secondary, proves more than usually obstinate, the practitioner should then have recourse to mercury; the previous treatment will render its curative influence at once safer and more energetic."

2. *Cold Affusion in Convulsions.* This remedy in the convulsions of children has not attracted much attention. Dr. Graves, having witnessed the good effects of the remedy, appears to have followed up the practice. The following case he offers as an example. A young gentleman, nine years of age, was convalescing from scarlatina, when anasarca, and afterwards convulsions, occurred. The fits were very violent, and the youth appeared moribund, his eyes being distorted, void of expression, and fixed—face cadaverous—extremities cold—pulse feeble, and 150.

"In addition, he appeared to be nearly destitute of muscular power, and in the interval between the fits was unable to speak, while a loud tracheal râle seemed to announce the near approach of death. As I have detailed the symptoms of the case with the most scrupulous fidelity, and without the least exaggeration, I need scarcely add, that our patient's case appeared utterly hopeless. Our first step was to place him in the arms of a strong nurse-tender, who maintained him as nearly as possible in the sitting posture; our object in this was to relieve the lungs, and diminish the cerebral congestion. Those who have watched over the dying, are aware that the final struggle may be often much protracted, by frequently changing the patient's position in bed, and particularly by avoiding the horizontal posture. This mode of proceeding, by preventing the gravitation of the blood to any one part of the lungs, and by counteracting the accumulation of mucus in any particular portion of the bronchial tubes, causes both to remain for a longer time pervious to the air, and favours the last efforts of the respiratory apparatus. We next proceeded to pour a small stream of cold water from a kettle on his head, the effects were extremely satisfactory, for in a short time the eyes assumed a more natural appearance, and lost the spasmodic fixedness, while the pulse became more and more distinct, and diminished in frequency; in short the violence of the fit soon subsided, he was able to expectorate the mucus which had clogged the larger air-passages, and had caused the *rattles,** and in the course of half an hour a very marked improvement was perceptible, the patient being then able to speak and swallow. The convulsions, however, returned several times during the ensuing day, but at each recurrence, their duration was lessened, and their violence diminished by the cold affusion. Sitting by the bed of this patient, I more than once was able to predict the immediate approach of the fit, by means of watching the pulsation of the carotids, which then became much more frequent and stronger. This observation, in connexion with the fact, that the pulse at the wrist became weaker and more indistinct at that very moment, suggests many interesting considerations concerning local determinations of blood.

It is almost unnecessary to remark, that the time we had so unexpectedly gained was not spent in inaction, and that we immediately had recourse to various other active remedies, such as leeching the neck, purgative injections, and mercurials, administered both internally and externally, with a view of affecting the mouth rapidly. In addition to the modes of applying mercury usually employed, I can recommend the application of the ointment to the armpits; this alone will frequently affect the mouth in a few days. The motions of the patient's arms here perform the office of friction, and this part of the skin seems to possess very active absorbing, as well as exhaling powers, and is likewise more protected from the contact of the clothes, &c., so that the ointment is less easily wiped off and wasted. With regard to local detraction of blood where there is determination to the head, experience has taught me, that in no case ought we to apply leeches to the temples. This is a very important observation, and applies to the treatment of various cerebral affections, such as occur in fever apoplexy, paralysis, hydrocephalus, &c. Leeching the temples in such cases, not unfrequently aggravates the cerebral symptoms, whereas, if the leeches are applied behind the ear, or what is still better, along one side of the neck, this untoward event will be avoided. I say along one side of the neck, because we are thus enabled to promote the flow of blood where the leeches fall off, with less annoyance to the patient than if leeches had been applied at both sides. A most instructive monograph might be written on the application of cold to the head in various diseases; at present, much mischief frequently arises from practitioners being unacquainted with the different degrees of cold suitable to different states of the cerebral organ, and the different methods of conducting its application, so as to produce relief. In one case of fever, I saw violent mania immediately follow the injudicious application of ice to the head; and in another, much difficulty was experienced in saving the life of a young person, in whom a collapse of the system, without relief of the local affection, had been induced by the too copious and continued application of cold water to the head. Where very violent pain in the head occurs in fever, the *cold dashing* with water from a height, as recommended by Dr. Smith in his excellent treatise, is often a most valuable remedy, but in convulsive diseases, like that now described, this application is too violent; in such cases, the stream of water should be small, not poured from a height, and should be discontinued the moment the fit ceases, to be again renewed on the approach of another paroxysm. I am informed by an eminent practitioner of this city, that he twice witnessed fatal convulsions follow the injudicious use of cold affusion in mania. The efficacy of cold affusion in delirium tremens, in asphyxia, in cases of over-

* "No bronchitis or pectoral affection was present, and consequently the *tracheal râle* (rattles) was of the most ominous import."

doses of prussic acid, &c., prove that it is too powerful an agent to be indiscriminately applied.

The young gentleman, whose case occasioned the foregoing remarks, recovered in the course of a few weeks, and is now perfectly well."

A case of hydrophobia, where strychnine was unsuccessful; and one of traumatic tetanus, where tobacco was ineffectually employed, are related.

3. *Neuralgia of the Mammæ.* This was the case of an unmarried lady, who was of sanguineous, robust habit. The disease had lasted two years, with various degrees of violence.

" During the paroxysms, which often lasted several days, and sometimes considerably longer, the mammæ, which in this lady were full and large, became extremely painful and tender, but were neither tumefied, hard, or red. The intervals between the paroxysms were marked not only by a total cessation, but by a gradual diminution of pain. At no period had there been any spinal tenderness. One breast was not more affected than the other, and the axillary glands were not swollen. She had consulted several practitioners, had taken much medicine. and made use of many topical applications, without relief. Leeches had been repeatedly applied, but their bites had invariably caused excruciating pain, and the bleeding they occasioned was not followed by the least relief. I at first tried stupes, narcotic liniments, and plasters, with warm salt-water baths, but these measures were unattended by the least improvement. The absence of complete intermissions, and of well-marked paroxysms, prevented me, during several weeks, from perceiving the true neuralgic nature of this pain ; at last this view of the subject occurred to me. I tried the carbonate of iron, with marked benefit. The disease has since frequently recurred, but its violence has always been lessened by the carbonate of iron. Sea bathing she likewise finds useful. I may here observe, that in those cases of neuralgia, in which carbonate of iron proves useful, I never find it necessary to raise the dose beyond one drachm, three times a day. Indeed a larger dose than half a drachm is seldom required. This statement of my experience, I consider necessary to counteract the impression made on the minds of students by a perusal of some of the London periodicals, where enormous doses of carbonate of iron are recommended, by no less an authority than the justly-celebrated Dr. Elliotson. I have examined this subject in a practical point of view with great attention, and think, that what is true concerning carbonate of iron, applies also to most tonic medicines. In fact we may consider it as a general rule, that tonics are rarely indicated, where moderate doses do not effect the desired purpose. This applies more particularly to the stronger tonics, such as the salts of iron, of arsenic, and quinine. I can scarcely conceive a case possible, in which a judicious physician will find it necessary for instance, to give more than ten grains of sulphate of quinine in a day, and yet much larger doses are not unusual here and elsewhere. Whenever the symptoms supposed to call for such a treatment, resist moderate doses of sulphate of quinine, we ought to pause, and reflect whether another plan of treatment ought to be adopted. There are two states of the system attended frequently with well-marked rigors, febrile paroxysms, and intermissions closely resembling ague ; I mean internal suppuration, and local inflammation without suppuration. Practical physicians are fully aware of this circumstance, *but there is another condition of the system in which symptoms simulating ague arise, totally unconnected with inflammation*, and of which I have seen two remarkable examples. They both occurred in females. One, a lady of a nervous temperament, in about a fortnight after her confinement, was affected with well-marked symptoms of quotidian ague, which grew worse and more violent during the exhibition of very large doses of sulphate of quinine, but she rapidly got rid of her complaint when, at my suggestion, camphor, aromatic spirit of ammonia, &c., were substituted in its place. In another lady, symptoms of tertian, and afterwards of double tertian, had continued for many weeks, and had reduced the patient extremely, sulphate of quinine, arsenic, and opium, had successively received a fair trial, but in vain. The disease, however, finally yielded to the exhibition of diffusible stimulants, used in combination with *antacids*."

We quite agree with Dr. Graves, in his observations on the doses of tonics. We rarely find it necessary to give more than six or eight grains of sulphate of quinine, or three or four scruples of carbonate of iron in the day.

4. *External Application of Narcotics.* A lady consulted our author, in June, 1831, for severe headache, coming on at uncertain periods, continuing one or several days, with intense agony. She passed sleepless nights occasionally; yet her general health was not much deteriorated.

"Usually the pain comes on at a certain hour in the evening, continues during the night, and diminishes about the same hour in the forenoon, but at times the pain continues for several days without any appreciable intermission. As she is of a bilious habit, I attempted the cure in the first instance by emetics, followed by purgatives, and finally by tonics, but without producing the least benefit. Carbonate of iron, sulphate of quinine and arsenic, were successively tried in vain. At last being sent for to see her in one of the violent paroxysms, I directed the scalp to be well steeped, and a narcotic plaster to be afterwards applied. I should have mentioned, that the hair had been frequently shaved for the purpose of trying tepid shower-baths, and that she never complained of tenderness in any part of the head, or even the feeling of external soreness, the sensation of pain being constantly referred to an internal headach. These circumstances were very unpromising, so far as regarded the probability of her receiving relief from the external application of narcotics; and to tell the truth, when I ordered the plaster, I myself did not expect much advantage from its use; and yet, strange to say, this method proved most effectual, as the pain immediately disappeared, and did not return for seven weeks.

She wore the plaster for a month, and when the pain returned, a second plaster again banished it. The following is the formula for this plaster:—

℞. Opii pulv. ℈ij.

Camphoræ, ʒss.

Picis Burgund.

Emplast. Lythargyr. āā q. s.

ut ft Emplastrum.

The quantity of narcotic ingredients given in this formula, is sufficient for the largest-sized plaster, for smaller they must be proportionally diminished; such plasters are of great service in rheumatic and neuralgic pains of the chest, back, loins, and occasionally they prove useful in sciatica; in the advanced stages of phthisis, much suffering is frequently produced by stitches, soreness, and pains in the sides and chest; in such cases, I always direct the part to be well stuped, and then rubbed with warm laudanum; this will very often procure immediate relief, but if it does not, we must apply a few leeches, and favour the flow of blood by the application of a cupping-glass, occasionally a very small venesection is necessary, and the application of a small blister to the painful part. Those who have not been engaged in practice will perhaps expect directions to enable them to distinguish which of these modes of treatment is suited to any particular pain. The pain of *pleurodyne*, they will say, is to be treated in one way, and that of pleurisy in another: now in the advanced stages of phthisis, it so happens, that the pleuritic affection occupies so small a space in most cases, that it cannot, *à priori*, be detected by the usual means of percussion and auscultation, and consequently we must try the remedies I have mentioned in succession; indeed I have seen laudanum and anodyne plasters succeed, where others believed that severer applications would have been necessary. In *crick of the neck*, diligent friction with laudanum affords immediate relief."

Dr. Graves promises to continue these contributions to medical science; and we shall always be happy to diffuse them as far as our Journal circulates.

---

### XXII.

### Case of diseased Heart, accompanied by Angina Pectoris, &c. By A. Newton, Esq.

[Dublin Journal, No. 2.]

The subject of this case was a man, 34 years of age, of sallow complexion, and who had enjoyed health till within these eight years, when he became affected with palpitations, head-ache, throbbing in his ears. He was then in the habit of sitting up very late, and drinking largely of green tea. On leaving off this beverage and adopting regular hours, most of the above symptoms abated; but after a lapse of three years the palpitations became troublesome, accompanied by pain and oppression at the epigastrium, lasting for a few minutes at a time. Two more years passed away before these symptoms became particularly distressing; and then the palpitations were occasionally violent, especially when suddenly excited by any circumstance.

"At a subsequent period, I had several opportunities of witnessing those attacks; on their approach he stood up and threw back his shoulders, his face was covered with perspiration, and expressive of great agony, neither the pulse nor the respirations were altered in character or frequency. He said that the pain, which was very intense, commenced at the epigastrium, whence it advanced upwards along the chest, and down the arms, till it reached the ends of his fingers, and then gradually subsided. These attacks were frequently terminated by eructation. He said that these paroxysms were much milder than those which he had at night."

The following was the result of an examination made in October, 1830.

"At this time his countenance was sallow; his lips livid; there was no emaciation. On exposing his neck and chest, the entire of the anterior wall of the thorax was seen elevated by each stroke of the heart; there was also a strong epigastric pulsation; the carotid and subclavian arteries throbbed violently. On applying the stethoscope over the region of the heart, the head was elevated by each cardiac impulse. The pulsations were irregular and intermitting. A very loud *bruit de soufflet* was heard, resembling the sound produced by a spinning wheel whilst in rapid motion; this sound accompanied both the auricular and the ventricular contractions, it was more marked at the left, than at the right side, but was distinctly audible over the entire chest; the respiratory murmur was every where natural, but feeble, and marked by the *bruit*. On examining the carotid, by means of the stethoscope, a loud and very distinct *bruit* was heard, differing however in character from that which existed at the heart, it was accompanied by a strong impulse; this phenomenon was observable, not only in the carotid and large arteries, but even in their minute ramifications, thus it was very apparent even down to the ends of the fingers. His pulse was full, hard, and intermitting, 88 in the minute. On comparing the pulse at the wrist, with the pulsations at the heart, the interval between them was much more marked than in health; the frequency of the pulse was not altered by change of posture; his appetite was bad; his bowels confined, and he was much troubled by flatulence."

In consultation with Dr. Marsh, it was determined to try the hydro-sulphuret of ammonia, in the dose of ten drops twice a day, gradually increasing it to 35 drops. Under this treatment the action of the heart greatly subsided, and the pulse fell to 48 in the minute, becoming much softer, and less irregular. His general health improved.

In the course of a fortnight the medicine nauseated the stomach, and was therefore temporarily omitted. But he continued to take it more or less for two months, during which the attacks were much less frequent than before. The medicine was discontinued, and he remained in tolerably good health for three months. He now was less attentive to regimen, and the paroxysms returned. The medicine was again resumed—and again benefit was experienced. Mr. Newton lost sight of him till July, 1831, when he was seized with cholera; but soon recovered. He was now earning a precarious existence as a waiter at different hotels, and the cardiac paroxysms were troublesome and frequent. An attack of bronchitis succeeded, and was relieved; but he suddenly expired one night in a paroxysm of dyspnœa.

*Dissection.* "On opening the skull, a considerable quantity of transparent colourless serum was found at the base of the brain, and in the ventricles, the brain and its membranes were healthy.

*Chest.*—About a pint of straw coloured fluid was found in each pleura, and about four ounces in the pericardium, the left lung was united to the ribs by some old bands of adhesion, both lungs were œdematous, but did not present any other lesion. On opening the pericardium, the heart was found enormously increased in size, this enlargement was principally at the left ventricle, the parietes of which were very much thickened, the cavity was not much larger than natural, the right ventricle was dilated, and its parietes slightly hypertrophied, the auricles were healthy, no valvular disease could be detected either in the heart or arteries, there was a very slight atheromatous deposit in the aorta immediately beyond the semilunar valves. The arteries of the neck, chest, and abdomen, presented no lesion of structure.

There was some serous effusion in the peritoneum, the abdominal viscera were healthy."

Mr. Newton makes many acute and sensible remarks on this case. He justly observes, the *bruit* is not a certain sign of valvular disease, since it has often been perceived where no organic change could be detected. Our own impression is that it is sometimes produced by mere strong action of the heart, and is the result of the mechanical friction of the current of blood through the orifices of the organ and along the arteries. How else can we account for the *bruit* in the carotid, or other arteries? Still we agree with our author that a permanent *bruit*, especially when combined with other anormal cardiac symptoms, is a pretty sure indication of organic disease. The following remarks are worthy of attention.

"Whilst upon the subject of diet, it may be mentioned, that every thing calculated to produce flatulence must be strictly prohibited, as this is a most troublesome and constant complication in cardiac disease.

Many objections apply to the treatment by digitalis; it is always a dangerous and often an uncertain remedy, and even in these cases in which it succeeds best, it soon loses its efficacy; its tendency to disorder the stomach is often such as to forbid its employment, it was therefore a great desideratum in medicine, to devise some plan of treatment which would lower the circulating system without producing permanent debility. Judging from the accounts which Cruickshanks and Rollo have given of the effects of hydrosulphuret of ammonia, it appeared to Dr. Marsh and me, that it might be advantageous in this case, particularly, as here, the angina seemed to depend upon organic disease of the heart; I tried it, and the event far surpassed our most sanguine expectations. Whilst the patient was upon meat diet and attending to his occupations, the action of the heart was lowered; the paroxysms of angina diminished in frequency, severity, and duration, and for a time were completely suspended; his general health also improved, and I may remark here, that his appetite instead of being destroyed, as is stated by Cruickshanks and Rollo to be the effect of this remedy, was, on the contrary, increased."

Mr. N. informs us that Dr. Marsh and himself have tried the hydrosulphuret of ammonia in various diseases, chiefly cardiac and cutaneous; in all of which it had a powerful effect in lowering the pulse, increasing the appetite, and promoting the secretion of urine. It is apt to be decomposed, and should be used fresh, and in drops rather than in draughts or mixtures.

### XXIII.

### Dr. Fergusson on Contagion.*

We regret that we are unable to give a detailed account of this very able paper, though that regret is much lessened by a knowledge of the wide circulation which Dr. Fergusson's paper has attained through the medium of our highly respected contemporary. We cannot, however, pass over the paper without paying our respects to a physician of great experience and accurate observation, who has dedicated much of his time and talents to the investigation of diseases—and especially of their causes, whether in human or in marsh emanations. The following extract will convey some idea of our ingenious and practical author's mode of reasoning.

"I assume, then, for reasons which I shall now farther illustrate, that endemic typhous fever is not essentially an infectious disease; that it may be approached at all times with impunity under ordinary circumstances of ventilation and personal purity; and that where those are observed, it cannot be carried or transported by any sick, however ill, so as to affect others in a different locality. To say that it has often spread to other inhabitants of the same locality or dwelling even, if it be incapable of transportation, does not constitute a contagion. It only amounts to a disease of locality, very frequently remittent or catarrhal fever, sublimed into *typhous* through neglect or improper treatment; and even should it infect visitors who choose to place themselves within its influence,—upon the same ground, that would be no proof of contagion, unless those visitors could also carry the infection so as to communicate it to others upon different ground at a distance; for to talk of contagion limited to one spot, is surely only saying that the spot of ground, and not the person of the patient, must be the source of the disease.

In this transportability resides the very touchstone and answer to the question of contagion. To suppose that a patient, infectious in his person, can only give out disease in a particular atmosphere and place, would be like what we have all read in the early lessons of our childhood, where an individual performs a prodigious leap in the island of Rhodes, but could not possibly be made to do it anywhere else. With equal justice may we assert that our contagious *exanthemata* can only become diffusible under similar circumstances, or that the infections of *syphilis* and *scabies* are influenced by the laws of atmosphere and locality. In regard to *variola*, the chief of those *exanthemata*, we may with more reason draw the direct contrary inference; for, previously to the discovery of the vaccine preventive, it used to be during the finest weather of the season that our babes, when carried out in arms, were struck and blighted by the disease, from passing even momentarily to leeward of an infected subject. Nor is it unreasonable to believe, that the very purity of the atmosphere actually contributed to the efficacy and surety of the infection; since then the contagion would be less adulterated and dilated with moisture, or other extraneous matter. No one would so dilute and obtund the actual variolous fluid (if a fluid) before proceeding to inoculation; and the contagious vapour can be nothing else than the same material under a gaseous form.

That other form of typhous fever which I may call factitious, (as being created by ourselves out of causes over which we ought to have exerted due control,) to distinguish it from the endemic, which we cannot prevent, must also be contagious under the same circumstances. Here, however, I believe, in like manner, that the person of the patient, independent of *fomites*, never gives out at any one time a sufficiency of the typhoid poison to affect another healthy person; that the poison can only be made effective through contamination of atmosphere, under long-

* Notes and Observations on the Contagion of Typhous Fever and Contagion generally. By W. Fergusson, M.D. (Corrected from the Edin. Med. and Surg. Journ.)

continued accumulation of morbific effluvia; and in fine, that the atmosphere of the patient is infectious, and not his person, which, if once cleaned and purified, and ventilation restored, may be approached, however ill he may be, with perfect impunity. In this belief I feel warranted, from the knowledge of several important facts, of a character so general as to warrant the greatest confidence in their application. 1*st*, The Bristol Hospital, for a great many years, has received typhous fevers into their well-disciplined wards, without having ever spread the disease even to the most contiguous beds. 2*d*, Several of the great hospitals in London have followed the same example with the same results. 3*d*, The most pestilentially dangerous fevers to approach when single, in the confined dwellings of the poor, have almost everywhere been found devoid of every infectious principle when collected together in numbers, and confined by the hundred within the walls of a well-regulated fever hospital. Upon this point the question of contagion must turn; for, if the evidence here given be not impugned, it will be impossible in human testimony to adduce anything more decisive and conclusive. Reasoning from single individual instances will generally deceive; but well-digested impartial observation upon masses of men can never lead to an erroneous conclusion.

While I thus, however, express my conviction of typhous fever never being a true essential contagion under any type, I must allow the question to be in some degree open to discussion, in regard to that (type) form which has an animal origin, and may justly be called an animal poison. The evidence of the great hospitals just alluded to would imply a negative; but it remains to be proved whether that belief may not have been founded upon the evidence of endemic typhus only, without experience of the other form of the disease. Thanks to our improved prison-discipline, such cases must now be rare. But when they existed in former times, the Black Assizes at Oxford, the Old Bailey Sessions, and other Sessions in Ireland and elsewhere, recorded in the different medical journals, would give fearful proof of its dreadful powers. I acknowledge, too, that the infection of hospital gangrene is a transportable and inoculable contagion, as applied to other ulcers, and that puerperal fever, another local modification of typhus, may be carried from house to house in any locality. With regard to the jail fever above alluded to, however, my own belief leads me to the conclusion, that the contagion resided in the woollen clothing of the prisoners in the dock, from whence it is stated that a stream of air from an open window blew directly upon the court; and that, had they been furnished with fresh clothing, and their bodies purified before being placed there, the closest proximity or actual contact would have produced no disease whatever.

This belief I ground on the fact, that woollen covering of any kind is as open and as prone to absorb, as it is tenacious to hold in accumulation, every species of animal exhalation; and the records of our press-gangs and prisons will show, that men would, in those days, be introduced into our fleet and armies, who for months together had never once had their clothing changed by night or by day; and this leads to another consideration of mighty import in the history and consideration of disease; for it would seem that these men had not themselves at the time the dreadful distemper, which they communicated with such fatal effect to the Court that tried them. That they had it not at the time I can fully credit; but that they were about to fall into it, and that, too, under its worst form, may be fairly presumed.

This proposition is not paradoxical; for it has been verified, and may be seen every autumn in all the aguish districts of our own country, that the actual inhabitant of the swamp itself, immersed night and day in a sea of malaria, is never so liable to fall under its influence as his neighbour on the dry rocky brow of the hill immediately above the marsh; thereby proving that the continuous application of a morbific power from without so deadens the nervous energy, that it becomes incapable of assuming the reaction and resistance which would be necessary to throw it off, under the form which nature has decreed of acute disease. The release from the deeply infected cells of a prison to the pure open air, would seem to restore this power to the prisoner, in the same way that the inhabitant of the hill, in a swampy country, from breathing alternately the wholesome air of Heaven, with the noxious vapours from below, always retains it, and leads the superficial observer to the fallacious conclusion of dry rocky ground being more pestiferous than the bed of the fen itself. But how different is the true state of the case. The dweller in the locality below may, it is true, exhibit no acute disease, but every organ is disordered ;—he has no health in any way, and is certainly destined to an early grave; while the other, although he may have imbibed the poison in its fullest dose, still possesses the power to throw it off, by an effort of his constitution, however dangerous and painful. He may, through such an ordeal, be restored to pristine health and vigour. With his benumbed neighbour, unless he removes from the marsh, it is impossible. He has been struck to the vitals, and must succumb to his fate. This is not imaginary, nor am I speaking of what I have not seen and examined. In many of the malarious districts of Spain, old age, among the resident inhabitants, was unknown. On the marshy banks of the great Guiana rivers, and in the central districts of Trinidad, presenting as concentrated a body of swamp as any in the world, our soldiers died by hundreds, blighted and withered, without ever showing a paroxysm of fever; and it is even recorded by Dr. Gregory of Edinburgh, in his lectures, that in a most unwholesome locality of the Southern United States of America, of which, at the distance of 40 years, I now forget the name, no native inhabitant constantly residing, ever attained the age of 21 years. I have been led into this digression from my desire to prove the possibility of the most intense existence of a cause, without a visible symbol of effect, as in the case of the prisoners of the Old Bailey, and the inhabitants of a deeply malarious county."

We recommend a perusal of the whole paper, in our respected cotemporary, to all our readers.

---

### XXVI.

### Ocular and Acoustic Illusions and Experiments.

It is a distinguishing and a gratifying feature of the extraordinary epoch in which we live, that men of great talent, great learning, and great reputation, are employed in writing for the instruction and entertainment of youth. In former ages great men wrote for the great, learned for the learned. Petrarch founded his hopes of immortality on his Latin compositions, yet those cherished works are mouldering in libraries, whilst his sonnets, composed in his despised native language, are the admiration of the world, and have obtained for him eternal renown. In those days, we say, the learned constituted a sort of separate fraternity, with masonic signs and a jargon of their own. They despised the mass of mankind, and esteemed it little short of desecration to labour for the enlightenment of the vulgar. Every period of existence, national and individual, has its peculiar wants. In the present state of civilization those wants are highly intellectual, the press has produced them, and the press only can satisfy them. In proportion as reading has become more general, it has also become more pure, and the class that in the year 1820 was delighted and contented with the trash of the Minerva Press, with ridiculous legends and supernatural romances, turns from them now with contempt and disgust, and seeks for productions fraught with more rational amusement and more wholesome information. When we look around us at the little world in our own island, and contemplate the progress of education, we cannot but feel how true was the exclamation that a little knowledge is a dangerous thing! A little knowledge is indeed dangerous, but it is infinitely better than none, because it *must* stimulate its possessor to acquire more. Children walk before they run, and the human mind has had its stages of infancy and debility precursory to vigour.

It is a maxim of commercial polity that the supply will be regulated by the demand, and the same holds with the traffic of the mind. In the æra of novels and romances the persons who ministered to the public appetite were mostly such as were unfit for the higher walks of literature. But as soon as a better taste was become diffused in the community, a few men of character and reputation felt that they would lose neither by embarking in the production of popular works. The example once given by a few has been followed by many, and the community having good books and bad placed before them, have shewn that they possess the sense of discriminating worth and of patronizing merit. This improvement has not been slow and gradual, it has almost been made by a bound, and probably there are few persons who would have ventured to predict even ten years ago that Sir Walter Scott would be writing history for his grandchildren—Sir David Brewster letters on natural magic for young people—or that a great society, composed of men distinguished by rank, and, what is better, of others renowned for their attainments, should be publishing sixpenny pamphlets for artisans, and establishing a penny magazine for all classes. The great experiment of educating every man is commenced in this country. What will be the result no one can say, but we do not doubt that it will be good. Knowledge is not only power, but in the gross it is virtue, enhances every good feeling, tends to moderate almost every bad. It is a wonderful sight to see the unwashed artificer speaking at public meetings, writing in periodical publications, acquainted with the policy of his own government, taking a deep interest in the situation of foreign nations, and discussing commercial and financial questions with as much good sense, in equal if not superior language, though not with quite so aristocratic a tone, as many a member of St. Stephen's chapel.

These remarks may seem misplaced in a medical journal, but we think that the connexion between the profession and the public is too close to admit of a separation of interest or destiny. If the intelligence of the age proceeds, the acquirements of medical men must correspond with it. Our profession has always been considered learned, but the standard measure of learning is undergoing material alterations, and the kind and quantity that sufficed in former times will not be adapted to the future. We do not hesitate to say, that the profession has sunk in public estimation, that it is sinking, and that it promises to sink yet farther. The *Times* newspaper is a good index of public opinion, it is, algebraically speaking, the exponent of its value. What did that newspaper observe in reference to the late lunatic inquisition on Miss Bagster? It stated that every trial in which medical men were interrogated as witnesses put the friends of the profession to the blush, and exposed the miserable system of education which could lead to such disgraceful exhibitions of inconsequential reasoning and shallow information. The *Times* is right. It is too melancholy to look on the profession, and see how it lags in the march of knowledge. The education of medical men in the mass is decidedly bad both in principle and in detail. It would be mischievous had it no sin but this, that it is a heterogeneous, patch-work thing, a congeries of pieces scarcely stitched together, and not a consistent whole. There is a mode of education for the apothecary, and a mode of education for the licentiate apothecary, and one for the surgeon, and one for the fellow of the College of Physicians, or rather the College of Fellows, and one for the licentiate of that College, and one—no, there is none for the physician-accoucheur. The sages who devised this precious compound of the exuviæ and excrementitious remains of the ancient and reputable corporation of barbers, and institution of that enlightened liberal, the eighth Henry, appear to have exercised the little wits they had in devising the minimum of knowledge necessary for apothecary, surgeon, or doctor to receive a license to practise their "mysterie and cunnynge"—and apothecary, surgeon, and doctor would seem to have seconded their laudable endeavour by ascertaining the minimum of study necessary to obtain that license. What with the corporations on one hand and human nature on the other, the medical profession is as we see it, distracted in itself, weak in its head, weak in its tail, with no unity of interest, no consent of action, flouted by lawyers, laughed at, pitied, and consulted by the public, despised by the press.

Let it not be imagined then that the great intellectual pursuits of the community, are rather matters of curiosity than of immediate importance to the members of the profession. We essentially belong to the community, grow with its growth, and decline with its decline; our honour is its opinion, our rank is its award. That honour and rank are not supported by prescriptive rights, nor heraldic blazonry; and when the world has begun to think meanly of us we are in fact degraded. The means of recovering the position which we certainly have lost should form the subject of serious consideration, of a cautious and deliberate attempt to accommodate our own internal constitution to the wants and wishes of the age, and to shake from us the incubus which the barbarous ideas of former generations have planted on our bosoms. We say that this should be the subject of anxious investigation, and we do not doubt that it will be so. Men's minds have of late been absorbed in the general contest between civilization and despotism, varied in its characters in different nations. In our own country the ideas of the age have obtained a signal triumph, and probably a new æra has dawned on our legislature. After such a mighty revolution achieved by the force of public opinion, it is idle to suppose that corporate bodies can successfully brave it. Sooner or later they will fall, consumed by the light of growing intelligence. If wisely disposed to suit themselves to circumstances, they may yet survive with an infusion of fresh and healthful vigour into their veins; but if not, they may be assured that we are no mendacious prophets when we foretel their inevitable destruction. The time is arriving when this discussion will be taken up, and when it is begun in earnest it will not be hastily abandoned.

These reflections were excited by a perusal of Sir David Brewster's Letters on Natural Magic, in the Family Library. We do not hesitate to say that every professional man should read the volume, and he will share in the advantages derived from its study by the rising generation, for which this Library has been professedly composed. We do not intend to give a formal account of the present work, but merely to select some portions containing curious facts or ingenious explanations in reference to what we may call the physiological department of magic.

### I. Ocular Illusions.

Sir David Brewster, after informing his readers that the part of the eye corresponding to the penetration of the sclerotica by the optic nerve is insensible to light, proceeds to describe another illusion of the eye that presented itself to him several years ago.

" When the eye is steadily occupied in viewing any particular object, or when it takes a fixed direction while the mind is occupied with any engrossing topic of speculation or of grief, it suddenly loses sight of, or becomes blind to, objects seen indirectly, or upon which it is not fully directed. This takes place whether we use one or both eyes, and the object which disappears will reappear without any change in the position of the eye, while other objects will vanish and revive in succession without any apparent cause. If a sportsman, for example, is watching with intense interest the motions of one of his dogs, his companion, though seen with perfect clearness by indirect vision, will vanish, and the light of the heath or of the sky will close in upon the spot which he occupied.

In order to witness this illusion, put a little bit of white paper on a green cloth, and within three or four inches of it, place a narrow strip of white paper. At the distance of twelve or eighteen inches, fix one eye steadily upon the little bit of white paper, and in a short time a part or even the whole of the strip of paper will vanish as if it had been removed from the green cloth. It will again reappear, and again vanish, the effect depending greatly on the steadiness with which the eye is kept fixed. This illusion takes place when both the eyes are open, though it is easier to observe it when one of them is closed. The same thing happens when the object is luminous. When a candle is thus seen by indirect vision, it never wholly disappears, but it spreads itself out into a cloudy mass, the centre of which is blue, encircled with a bright ring of yellow light.

This inability of the eye to preserve a sustained vision of objects seen obliquely, is curiously compensated by the greater sensibility of those parts of the eye that have this defect. The eye has the power of seeing objects with perfect distinctness, only when it is directed straight upon them; that is, all objects seen indirectly are seen indistinctly; but it is a curious circumstance, that when we wish to obtain a sight of a very faint star, such as one of the satellites of Saturn, we can see it most distinctly *by looking away from it,* and when the eye is turned full upon it, it immediately disappears."

Sir D. B. observes that the most efficacious illusion probably arises from the difficulty with which objects are seen, when illuminated by a small degree of light. The eye becomes fixed very steadily upon them, the exertion throws it into a state of painful agitation, the object swells and contracts and partly disappears, and will again become visible when the eye has recovered from the delirium into which it was thrown. Let time and place and circumstance be favorable, and it is not difficult to conceive that strong minded persons, much more the credulous and timid, may be deceived by this physical illusion into the belief that the distorted spectrum before them is actually some disembodied sprite, or unearthly messenger. As Sir David shrewdly hints, it is remarkable that spectres are mostly seen by the young and the ignorant at night, and that they are always *white,* the only colour which, under such cicumstances, could be seen. The observations of Sir David Brewster on what he terms the phosphorescence of the retina are also deserving of attention.

"Another class of ocular deceptions have their origin in a property of the eye which has been very imperfectly examined. The fine nervous fabric which constitutes the retina, and which extends to the brain, has the singular property of being *phosphorescent by pressure.* When we press the eye-ball outwards by applying the point of the finger between it and the nose, a circle of light will be seen, which Sir Isaac Newton describes as 'a circle of colours like those in the feather of a peacock's tail.' He adds, that 'if the eye and the finger remain quiet, these colours vanish in a second of time, but if the finger be moved with a quavering motion they appear again.' In the numerous observations which I have made on these luminous circles, I have never been able to observe any colour but white, with the exception of a general red tinge which is seen when the eyelids are closed, and which is produced by the light which passes through them. The luminous circles too always continue while the pressure is applied, and they may be produced as readily after the eye has been long in darkness as when it has been recently exposed to light. When the pressure is very gently applied, so as to compress the fine pulpy substance of the retina, light is immediately created when the eye is in total darkness; and when in this state light is allowed to fall upon it, the part compressed is more sensible to light than any other part, and consequently appears more luminous. If we increase the pressure, the eye-ball, being filled with incompressible fluids, will protrude all round the point of pressure, and consequently the retina at the protruded part will be *compressed* by the outward pressure of the contained fluid, while the retina on each side, namely, under the point of pressure and beyond the protruded part, will be drawn towards the protruded part or *dilated.* Hence the part under the finger which was originally compressed is now *dilated,* the adjacent parts *compressed,* and the more remote parts immediately without this *dilated* also. Now we have observed, that when the eye is, under these circumstances, exposed to light, there is a bright luminous circle shading off externally and internally into total darkness. We are led therefore to the important conclusions, that when the retina is compressed in total darkness it gives out light; that when it is compressed when exposed to light, its sensibility to light is increased; and that when it is *dilated under exposure to light, it becomes absolutely blind, or insensible to all luminous impressions*."

Whether this conclusion of Sir David's be positively true or not we do not feel qualified to decide, not having made sufficient experiments relative to the point in question. In headache attended with derangement of the stomach this phosphorescence of the retina assumes a new shape. When the eye is in total darkness, a faint blue light floats before it, varying in its shape, and passing away at one side. This blue light increases in intensity, becomes green and then yellow and sometimes rises to red; all these colours being frequently seen at once, or the mass of light shades off into darkness.

To the phenomena called ocular spectra, or accidental colours, we need not advert, as many of our readers are no doubt acquainted with their general characters. But these accidental spectra are occasionally very disagreable. Mr. Boyle, in his book of colours, mentions an individual who continued for years to see the spectre of the sun when he looked upon bright objects. Mr. Locke being struck with the fact applied to Sir Isaac Newton for an explanation of it, which elicited the following curious narrative of a similar circumstance that happened to himself.

"The observation you mention in Mr. Boyle's book of colours, I once made upon myself with the hazard of my eyes. The manner was this: I looked a very little while upon the sun in the looking-glass with my right eye, and then turned my eyes into a dark corner of my chamber, and winked, to observe the impression made, and the circles of colours which encompassed it, and how they decayed by degrees, and at last vanished. This I repeated a second and a third time. At the third time, when the phantasm of light and colours about it were almost vanished, intending my fancy upon them to see their last appearance, I found, to my amazement, that they began to return, and by little and little to become as lively and vivid as when I had newly looked upon the sun. But when I ceased to intend my fancy upon them they vanished again. After this, I found, that, as often as I went into the dark, and intended my mind upon them, as when a man looks earnestly to see any thing which is difficult to be seen, I could make the phantasm return without looking any more upon the sun; and the oftener I made it return, the more easily I could make it return again. And at length, by repeating this without looking any more upon the sun, I made such an impression on my eye, that, if I looked upon the clouds, or a book, or any bright object, I saw upon it a round bright spot of light like the sun, and, which is still stranger, though I looked upon the sun with my right eye only, and not with my left, yet my fancy began to make an impression upon my left eye as well as upon my right. For if I shut my right eye, and looked upon a book or the clouds with my left eye, I could see the spectrum of the sun almost as plain as with my right eye, if I did but intend my fancy a little while upon it; for at first, if I shut my right eye, and looked with my left, the spectrum of the sun did not appear till I intended my fancy upon it; but by repeating, this appeared every time more easily. And now in a few hours time I had brought my eyes to such a pass, that I could look upon no bright object with either eye but I saw the sun before me, so that I durst neither write nor read; but to recover the use of my eyes, shut myself up in my chamber made dark, for three days together, and used all means in my power to direct my imagination from the sun. For if I thought upon him, I presently saw his picture, though I was in the dark. But by keeping in the dark, and employing my mind about other things, I began in three or four days to have more use of my eyes again; and by forbearing to look upon bright objects, recovered them pretty well; though not so well but that, for some months after, the spectrums of the sun began to return as often as I began to meditate upon the phenomena, even though I lay in bed at midnight with my curtains drawn. But now I have been very well for many years, though I am apt to think, if I durst venture my eyes, I could still make the phantasm return by the power of my fancy. This story I tell you, to let you understand, that in the observation related by Mr. Boyle, the man's fancy probably concurred with the impression made by the sun's light to produce that phantasm of the sun which he constantly saw in bright objects."

Passing over some other matters we may pause for a minute to consider the occasional insensibility of the eye to particular colours, unaccompanied by other imperfection of vision, or derangement of health. The case of Harris the shoemaker at Maryport, related by Mr. Huddart, is familiar to most medical readers; he was capable of recognizing only the two opposite colours of *black* and *white*. Mr. Scott in describing his own case in the Philosophical Transactions stated that he did not know any *green*—that a *pink* and a *pale blue* were alike to him—that he often thought a *full red* and a *full green* a good match—that he sometimes could not distinguish a *full purple* from a *deep blue*, but that he knew light, dark, and middle *yellows*, and all degrees of *blue* except *sky blue*. This gentleman was angry, because on the occasion of his daughter's marriage, the bridegroom, " a gentleman of the law, in a fine rich claret-coloured dress," appeared to him to come in black. The lady, however, had better vision, and liking the colour, which she said " was very genteel," the ceremony was not interrupted in consequence. Mr. Scott's father, his maternal uncle, one of his sisters, and her two sons, had all the same imperfection. Dr. Nicholl recorded a case where a naval officer purchased a *blue* uniform coat and waistcoat with *red* breeches to match the blue, and Mr. Harvey describes the case of a tailor at Plymouth, who on one occasion repaired an article of dress with *crimson* in place of *black* silk, and on another patched the elbow of a *blue* coat with a piece of *crimson* cloth.

" It deserves to be remarked that our celebrated countrymen the late Mr. Dugald Stewart, Mr. Dalton, and Mr. Troughton, have a similar difficulty in distinguishing colours. Mr. Stewart discovered this defect when one of his family was admiring the beauty of the Siberian crab-apple, which he could not distinguish from the leaves but by its form and size. Mr. Dalton cannot distinguish *blue* from pink, and the solar spectrum consists only of two colours, *yellow* and *blue*. Mr. Troughton regards *red*, *ruddy pinks* and brilliant *oranges*, as *yellows*, and *greens* as *blues*, so that he is capable only of appreciating *blue* and *yellow* colours.

In all those cases which have been carefully studied, at least in three of them in which I have had the advantage of making personal observations, namely, those of Mr. Troughton, Mr. Dalton, and Mr. Liston, the eye is capable of seeing the whole of the prismatic spectrum, the red space appearing to be yellow. If the red space consisted of homogeneous or simple red rays, we should be led to infer that the eyes in question were not insensible to red light, but were merely incapable of discriminating between the impressions of red and yellow light. I have lately shown, however, that the prismatic spectrum consists of three equal and coincident spectra of *red*, *yellow*, and *blue* light, and consequently, that much yellow and a small portion of blue light exist in the red space;—and hence it follows, that those eyes which see only two colours, viz. *yellow* and *blue*, in the spectrum, are rarely insensible to red light of the spectrum, and see only the yellow with the small portion of blue with which the red is mixed. The faintness of the yellow light which is thus seen in the the red space, confirms the opinion that the retina has not appreciated the influence of the simple red rays."

Sir David relates a very curious accident which happened to himself, and might possibly happen in some degree to others.

" In the course of writing the preceding observations, an ocular illusion occurred to myself of so extraordinary nature, that I am convinced it never was seen before, and I think it far from probable that it will ever be seen again. Upon directing my eyes to the candles that were standing before me, I was surprised to observe, apparently among my hair, and nearly straight above my head, and far without the range of vision, a distinct image of one of the candles inclined about 45° to the horizon, as shown at A in Fig. 2. The image was as distinct and perfect as if it had been formed by reflexion from a piece of mirror glass, though of course much less brilliant, and the position of the image proved that it must be formed by reflexion from a perfectly flat and highly polished surface. But where such a surface could be placed, and how, even if it were fixed, it could reflect the image of the candle up through my head, were difficulties not a little perplexing. Thinking that it might be something lodged in the eyebrow, I covered it up from the light, but the image still retained its place. I then examined the eyelashes with as little success, and was driven to the extreme supposition that a crystallization was taking place in some part of the aqueous humour of the eye, and that the image was formed by the reflexion of the light of the candle from one of the crystalline faces. In this state of uncertainty, and, I may add, of anxiety, for this last supposition was by no means an agreeable one, I set myself down to examine the phenomenon experimentally. I found that the image varied its place by the motion of the head and of the eyeball, which proved that it was either attached to the eyeball or occupied a place where it was affected by that motion. Upon inclining the candle at different angles the image suffered corresponding variations of position. In order to determine the exact place of the reflecting substance, I now took an opaque circular body and held it between the eye and the candle till it eclipsed the mysterious image. By bringing the body nearer and nearer the eyeball till its shadow became sufficiently distinct to be seen, it was easy to determine the locality of the reflector, because the shadow of the opaque body must fall upon it whenever the image of the candle was eclipsed. In this way I ascertained that the reflecting body was in the upper eyelash, and I found, that, in consequence of being disturbed, it had twice changed its inclination, so as to represent a vertical candle in the horizontal position B, and afterwards in the inverted position C. Still, however, I sought for it in vain, and even with the aid of a magnifier I could not discover it. At last, however, Mrs. B. who possesses the perfect vision of short-sighted persons, discovered, after repeated examinations, between two eyelashes, a minute speck, which, upon being removed with great difficulty, turned out to be a chip of red wax not above the hundredth part of an inch in diameter, and having its surface so perfectly flat and so highly polished that I could see in it the same image of the candle, by placing it extremely near the eye. This chip of wax had no doubt received its flatness and its polish from the surface of a seal, and had started into my eye when breaking the seal of a letter."

The third letter is occupied with the consideration of those spectral illusions, or apparitions, which, as in Nicolai's celebrated case, may be occasioned by derangement of the health, or, as in the thousand and one *authenticated* ghost-stories, by the influence of imagination, fear, remorse, or superstition. Sir David communicates to the world the case of Mrs. A. resembling in some respects that of Nicolai. In Mrs. A. however, there appears to exist a more excitable temperament, and a susceptibility of nervous system which did not exist in the bookseller of Berlin, circumstances which detract in some measure from the surprise which the singular illusions would otherwise create in us. Mrs. A. too had been studying Dr. Hibbert's Philosophy of Apparitions, in which Nicolai's case is related: and Sir Walter Scott's Letters on Demonology. As this is a course of reading, not usually followed by ladies, it appears to us to prove two things:—first that Mrs. A. must have had something like an original disposition towards the supernatural; and secondly that disposition would in all probability have been increased by the perusal of works like those of Dr. Hibbert and Sir Walter Scott. All these circumstances should be looked to in endeavouring to account for the illusions to which Mrs. A. became and still is subject, and with such a state of mind we need not be surprised that corporeal derangement has not been so manifest in her as in Nicolai. As a specimen of the spectra which presented themselves to Mrs. A. we subjoin the following.

"On the 17th of March, Mrs. A. was preparing for bed. She had dismissed her maid, and was sitting with her feet in hot water. Having an excellent memory, she had been thinking upon and repeating to herself a striking passage in the Edinburgh Review, when, on raising her eyes, she saw seated in a large easy chair before her the figure of a deceased friend, the sister of Mr. A. The figure was dressed, as had been usual with her, with great neatness, but in a gown of a peculiar kind, such as Mrs. A. had never seen her wear, but exactly such as had been described to her by a common friend as having been worn by Mr. A.'s sister during her last visit to England. Mrs. A. paid particular attention to the dress, air, and appearance of the figure, which sat in an easy attitude in the chair, holding a handkerchief in one hand. Mrs. A. tried to speak to it, but experienced a difficulty in doing so, and in about three minutes the figure disappeared. About a minute afterwards, Mr. A. came into the room, and found Mrs. A. slightly nervous, but fully aware of the delusive nature of the apparition. She described it as having all the vivid colouring and apparent reality of life; and for some hours preceding this and other visions, she experienced a peculiar sensation in her eyes, which seemed to be relieved when the vision had ceased."

It appears from a consideration of the whole of this interesting case that Mrs. A. is a person, who, if uneducated and placed under favourable circumstances, would not only have seen but believed in apparitions. Having studied the philosophy of the subject, her mind is sufficiently strengthened to perceive that the whole are but 'airy nothings', whilst her temperament continues sufficiently excitable to conjure them up from the regions of imagination, and to convert the forms abiding in her memory into illusive objects of the sense.

The following observations of Sir David Brewster are sufficiently interesting to merit extraction, being insusceptible of useful abbreviation.

"In his admirable work on this subject, Dr. Hibbert has shown that spectral apparitions are nothing more than ideas or the recollected images of the mind, which in certain states of bodily indisposition have been rendered more vivid than actual impressions, or, to use other words, that the pictures in the 'mind's eye' are more vivid than the pictures in the body's eye. This principle has been placed by Dr. Hibbert beyond the reach of doubt; but I propose to go much farther, and to show that the 'mind's eye' is actually the body's eye, and that the retina is the common tablet on which both classes of impressions are painted, and by means of which they receive their visual existence according to the same optical laws. Nor is this true merely in the case of spectral illusions: It holds good of all ideas recalled by the memory or created by the imagination, and may be regarded as a fundamental law in the science of pneumatology.

It would be out of place in a work like this to adduce the experimental evidence on which it rests, or even to explain the manner in which the experiments themselves must be conducted; but I may state in general, that the spectres conjured up by the memory or the fancy have always a 'local habitation,' and that they appear in front of the eye, and partake in its movements exactly like the impressions of luminous objects after the objects themselves are withdrawn.

In the healthy state of the mind and body, the relative intensity of these two classes of impressions on the retina are nicely adjusted. The mental pictures are transient and comparatively feeble, and in ordinary temperaments are never capable of disturbing or effacing the direct images of visible objects. The affairs of life could not be carried on if the memory were to intrude bright representations of the past into the domestic scene, or scatter them over the external landscape. The two opposite impressions, indeed, could not co-exist: The same nervous fibre which is carrying from the brain to the retina the figures of memory, could not at the same instant be carrying back the impressions of external objects from the retina to the brain. The mind cannot perform two different functions at the same instant, and the direction of its attention to one of the two classes of impressions necessarily produce the extinction of the other: But so rapid is the exercise of mental power, that the alternate appearance and disappearance of the two contending impressions is no more recognized than the successive observations of external objects during the twinkling of the eyelids. If we look, for example, at the Façade of St. Paul's, and, without changing our position, call to mind the celebrated view of Mont Blanc from Lyons, the picture of the cathedral, though actually impressed upon the retina, is momentarily lost sight of by the mind, exactly like an object seen by indirect vision; and during the instant the recollected image of the mountain, towering over the subjacent range, is distinctly seen, but in a tone of subdued colouring, and indistinct outline. When the purpose of its recal is answered, it quickly disappears, and the picture of the cathedral again resumes the ascendancy.

In darkness and solitude, when external objects no longer interfere with the pictures of the mind, they become more vivid and distinct; and in the state between waking and sleeping, the intensity of the impressions approaches to that of visible objects. With persons of studious habits, who are much occupied with the operations of their own minds, the mental pictures are much more distinct than in ordinary persons; and in the midst of abstract thought, external objects even cease to make any impression on the retina. A philosopher absorbed in his contemplations experiences a temporary privation of the use of his senses. His children or his servants will enter the room directly before his eyes without being seen. They will speak to him without being heard; and they will even try to rouse him from his reverie without being felt; although his eyes, his ears, and his nerves, actually receive the impressions of light, sound, and touch. In such cases, however, the philosopher is voluntarily pursuing a train of thought on which his mind is deeply interested; but even ordinary men, not much addicted to speculations of any kind, often perceive in their mind's eye the pictures of deceased or absent friends, or even ludicrous creations of fancy, which have no connexion whatever with the train of their thoughts. Like spectral apparitions they are entirely involuntary, and though they may have sprung from a regular series of associations, yet it is frequently impossible to discover a single link in the chain.

If it be true, then, that the pictures of the mind and spectral illusions are equally impressions upon the retina, the latter will differ in no respect from the former, but in the degree of vividness with which they are seen; and those frightful apparitions become nothing more than our ordinary ideas, rendered more brilliant by some accidental and temporary derangement of the vital fuuctions. Their very vividness too, which is their only characteristic, is capable of explanation. I have already shown that the retina is rendered more sensible to light by voluntary local pressure, as well as by the involuntary pressure of the bloodvessels behind it; and if, by looking at the sun, we impress upon the retina a coloured image of that luminary, which is seen even when the eye is shut, we may by pressure alter the colour of that image, in consequence of having increased the sensibility of that part of the retina on which it is impressed. Hence we may readily understand how the vividness of the mental pictures must be increased by analogous causes.

In the case both of Nicolai and Mrs. A. the immediate cause of the spectres was a deranged action of the stomach When such a derangement is induced by poison, or by substances which act as poisons, the retina is peculiarly affected, and the phenomena of vision singularly changed. Dr. Patouillet has described the case of a family of *nine* persons who were all driven mad by eating the root of the *Hyoscyamus niger* or black Henbane. One of them leapt into a pond. Another exclaimed that his neighbour would lose a cow in a month, and a third vociferated that the crown piece of sixty pence would in a short time rise to five livres. On the following day they had all recovered their senses, but recollected nothing of what had happened. On the same day they all saw objects double, and, what is still more remarkable, on the third day *every object appeared to them as red as scarlet.* Now this red light was probably nothing more than the red phosphorescence produced by the pressure of the blood-vessels on the retina, and analogous to the masses of *blue, green, yellow,* and *red* light, which have been already mentioned as produced by a similar pressure in headaches, arising from a disordered state of the digestive organs.

Were we to analyse the various phenomena of spectral illusions, we should discover many circumstances favourable to these views. In those seen by Nicolai the individual figures were always somewhat paler than natural objects. They sometimes grew more and more indistinct, and became perfectly white; and, to use his own words, 'he could always distinguish with the greatest precision, phantasms from phenomena.' Nicolai sometimes saw the spectres when his eyes were shut, and sometimes they were thus made to disappear,—effects perfectly identical with those which arise from the impressions of very luminous objects. Sometimes the figures vanished entirely, and at other times only pieces of them disappeared, exactly conformable to what takes place with objects seen by indirect vision, which most of those figures must necessarily have been.

Among the peculiarities of spectral illusions there is one which merits particular attention, namely, that they seem to cover or conceal objects immediately beyond them. It is this circumstance more than any other which gives them the character of reality, and at first sight it seems difficult of explanation. The distinctness of any impression on the retina is entirely independent of the accommodation of the eye to the distinct vision of external objects. When the eye is at rest, and is not accommodated to objects at any particular distance, it is in a state for seeing distant objects most perfectly. When a distinct spectral impression, therefore, is before it, all other objects in its vicinity will be seen indistinctly, for while the eye is engrossed with the vision, it is not likely to accommodate itself to any other object in the same direction. It is quite common, too, for the eye to see only one of two objects actually presented to it. A sportsman who has been in the practice of shooting with both his eyes open actually sees a double image of the muzzle of his fowling-piece, though it is only with one of these images that he covers his game, having no perception whatever of the other. But there is still another principle upon which only one of two objects may be seen at a time. If we look very steadily and continuously at a double pattern, such as those on a carpet composed of two single patterns of different colours, suppose *red* and *yellow;* and if we direct the mind particularly to the contemplation of the red one, the green pattern will sometimes vanish entirely, leaving the red one alone visible, and by the same process the *red* one may be made to disappear. In this case, however, the two patterns, like the two images, may be seen together; but if the very same portion of the retina is excited by the direct rays of an external object, when it is excited by a mental impression, it can no more see them both at the same time than a vibrating string can give out two different fundamental sounds. It is quite possible, however, that the brightest parts of a spectral figure may be distinctly seen along with the brightest parts of an object immediately behind it, but then the bright parts of each object will fall upon different parts of the retina.

These views are illustrated by a case mentioned by Dr. Abercrombie. A gentleman, who was a patient of his, of an irritable habit, and liable to a variety of uneasy sensations in his head, was sitting alone in his dining-room in the twilight, when the door of the room was a little open. He saw distinctly a female figure enter, wrapped in a mantle, with the face concealed by a large black bonnet. She seemed to advance a few steps towards him, and then stop. He had a full conviction that the figure was an illusion of vision, and he amused himself for some time by watching it; at the same time observing that he could see through the figure so as to perceive the lock of the door, and other objects behind it.*

If these views be correct the phenomena of spectral apparitions are stripped of all their terror, whether we view them in their supernatural character or as indications of bodily indisposition. Nicolai, even, in whose case they were accompanied with alarming symptoms, derived pleasure from the contemplation of them, and he not only recovered from the complaint in which they originated, but survived them for many years.—Mrs. A., too, who sees them only at distant intervals, and with whom they have but a fleeting existence, will, we trust, soon lose her exclusive privilege, when the slight indisposition which gives them birth has subsided."

As Sir David has not advanced all his proofs and experiments in confirmation of his theory, that the "mind's eye" is really the "body's eye," we can neither be satisfied of its truth, nor fairly attempt to disprove its accuracy. Yet we venture to make one remark. Memory is the preservation of the impressions which have formerly been transmitted to the mind by the senses from without, or from the organic functions and sympathies within; such, at least, would be our definition of it. Memory is, therefore, a composite thing, the result of different impressions. Our remembrance of a friend implies our recollection of his external form, his voice, his character, and so on; our recollection of a place, our native village for example, is the recollection of the pleasant meadows which we *saw*, the bubbling rivulet which we *heard*, the hawthorn and the wild rose which we *smelt*, the balmy freshness of the air, so grateful to our sense of *feeling*, and the thousand things which appeal more particularly to the heart, and tell it of other and happier days.

Let us apply these facts to the explanation of spectral illusions. If a person sees before him the image of an absent or departed friend, we suppose that it is a capricious and fitful exercise of the memory. If the person does no more than *see* the spectre, it is manifest that this exercise of memory is but partial, a recal of only the impression formerly received through the sense of vision. Now Sir David believes that this is absolutely seated in the organic retina. But suppose the spectre a more comprehensive one, that we not merely see its form, but appear to hear its voice and listen to its admonition—or in place of imagining, as Sir David has beautifully done, the panorama of Mont Blanc gliding in, for a moment, between us and St. Paul's, let us venture to mount to the summit of that cathedral—let us glance at the mighty city at our feet, and, whilst musing on its future destiny, transplant ourselves in fancy to Rome—see the city of Romulus spread before us, its triumphs, its glories, and its crimes—pass in rapid review its rise, its zenith, and its fall—go to battle with Romulus, to the groves with Numa—hear Cicero in the Senate, and denounce his proscription—feel as true Romans felt, when tyrant after tyrant assumed the purple to commit crimes on an imperial scale—shudder at the march of Attila, and grieve at the sacking of the Bourbon—let us do all this, and we shall but have indulged in a reverie, in which many with classical recollections have indulged. Now the simple recollection of a striking landscape differs from this historical effort of memory and fancy only in ex-

* "Inquiries concerning the Intellectual Powers, and the Investigation of Truth. Edinburgh, 1830."

tent—and the same may be said of the mere recal of the form of an absent person, or of this with the addition of other attributes, requiring the presence of other senses besides vision for their perception.

Now shall it be said, in this wider view of the subject, that the perception of what we do ideally perceive is not in the brain, but that the organic sense is the immediate and efficient agent? In recalling the image of the country, is the perception of the sounds which we seem to hear, of the fragrance which we seem to smell, seated in the organs of hearing and smelling? We apprehend not. It must be borne in mind, that the sense is not actually existent in the organic apparatus by which it is obtained. The eye is the organ of vision, but the actual faculty is, in all probability, seated in the brain. The eye is the apparatus by which visual phenomena make their impression on the brain; that impression having been made, the memory of it remains, and surely in the brain. The objection urged by Sir David Brewster, that spectra are affected by the motions of the eyes, in fact, by what modifies actual vision, is specious, but not conclusive. It must be recollected that impressions received through the medium of the eye, are impressions which have been produced and regulated by the laws of light. If the memory of them be accurate, it is the memory of impressions which, having been obedient to those laws, must continue to be so; if not, the memory is false, the spectre conjured up is monstrous, and, as it would sin against our experience, its very irregularity would convict it. Again, the spectre of a visual object, if raised by the mind, must be presented to the eye; if not, it could not possibly be the representation of what we had formerly seen, nor would it evince the agency of memory. We throw out these hints, without pretending to have paid great attention to the subject; and as neither ourselves nor our readers are particularly partial to metaphysical reasonings, in the pages of a practical journal, we pass to something else.

There is an optical illusion which may readily occur, which has attracted some attention, and of which it is not amiss for medical men to be aware; it is that by which depressions are conceived to be elevations, and elevations depressions, or by which intaglios are converted into cameos, and cameos into intaglios.

"This curious fact seems to have been first observed at one of the early meetings of the Royal Society of London, when one of the members, in looking at a guinea through a compound microscope of new construction, was surprised to see the head upon the coin depressed, while other members could only see it embossed as it really was.

While using telescopes and compound microscopes, Dr. Gmelin, of Wurtemburg, observed the same fact. The protuberant parts of objects appeared to him depressed, and the depressed parts protuberant; but what perplexed him extremely, this illusion took place at some times and not at others, in some experiments and not in others, and appeared to some eyes and not to others.

After making a great number of experiments, Dr. Gmelin is said to have constantly observed the following effects: Whenever he viewed any object rising upon a plane of any colour whatever, provided it was neither white nor shining, and provided the eye and the optical tube were directly opposite to it, the elevated parts appeared depressed, and the depressed parts elevated. This happened when he was viewing a seal, and as often as he held the tube of the telescope perpendicularly, and applied it in such a manner that its whole surface almost covered the last glass of the tube. The same effect was produced when a compound microscope was used. When the object hung perpendicularly from a plane, and the tube was supported horizontally and directly opposite to it, the illusion also took place, and the appearance was not altered when the object hung obliquely and even horizontally. Dr. Gmelin is said to have at last discovered a method of preventing this illusion, which was, by looking not towards the centre of the convexity, but at first to the edges of it only, and then gradually taking in the whole. 'But why these things should so happen, he did not pretend to determine.'

The best method of observing this deception, is to view the engraved seal of a watch with the eye-piece of an achromatic telescope, or with a compound microscope, or any combination of lenses, which inverts the objects that are viewed through it.* The depression in the seal will immediately appear an elevation, like the wax impression which is taken from it; and though we know it to be hollow, and feel its concavity with the point of our finger, the illusion is so strong that it continues to appear a protuberance. The cause of this will be understood from Fig. 14, where S is the window of the apartment, or the light which illuminates the *hollow* seal L R, whose shaded side is of course on the same side L with the light. If we now invert the seal with one or more lenses, so that it may look in the opposite direction, it will appear to the eye as in Fig. 15, with the shaded side L farthest from the window. But as we know that the window is still on our left hand, and that the light falls in the direction R L, and as every body with its shaded side farthest from the light must necessarily be convex or protuberant, we immediately believe that the hollow seal is now a cameo or bas-relief. The proof which the eye thus receives of the seal being raised, overcomes the evidence of its being hollow derived from our actual knowledge, and from the sense of touch. In this experiment the deception takes place from our knowing the real direction of the light which falls upon the seal; for if the place of the window, with respect to the seal, had been inverted as well as the seal itself, the illusion could not have taken place."†

* "A single convex lens will answer the purpose, provided we hold the eye six or eight inches behind the image of the seal formed in its conjugate focus."

† Two woodcuts illustrate the explanations, but they can be understood sufficiently without them, although it is necessary to leave the referential letters. —*Ed.*

### II. Acoustic Illusions.

The doctrines of sound have been long and successfully applied to the purposes of delusion, and its phenomena have contributed in a very great degree to establish the influence of superstitious feelings, and to enable the weak to deceive themselves and others. Within a certain angle sounds are imperfectly discriminated, and we judge with much uncertainty of their direction and distance; an uncertainty which is the foundation of the art of ventriloquism.

"If we place ten men in a row at such a distance from us that they are included in the angle within which we cannot judge of the direction of sound, and if in a calm day each of them speaks in succession, we shall not be able with closed eyes to determine from which of the ten men any of the sounds proceeds, and we shall be incapable of perceiving that there is any difference in the direction of the sounds emitted by the two outermost; If a man and a child are placed within the same angle, and if the man speaks with the accent of a child without any corresponding motion in his mouth or face, we shall necessarily believe that the voice comes from the child: Nay, if the child is so distant from the man that the voice actually appears to us to come from the man, we will still continue in the belief that the child is the speaker; and this conviction would acquire additional strength if the child favoured the deception by accommodating its features and gestures to the words spoken by the man. So powerful, indeed, is the influence of this deception, that if a jack-ass placed near the man were to open its mouth, and shake its head responsive to the words uttered by his neighbour, we would rather believe that the ass spoke than that the sounds proceeded from a person whose mouth was shut, and the muscles of whose face were in perfect repose. If our imagination were even directed to a marble statue or a lump of inanimate matter, as the source from which we were to expect the sounds to issue, we would still be deceived, and would refer the sounds even to these lifeless objects. The illusion would be greatly promoted if the voice were totally different in its tone and character from that of the man from whom it really comes; and if he occasionally speak in his own full and measured voice, the belief will be irresistible that the assumed voice proceeds from the quadruped or from the inanimate object.

When the sounds which are required to proceed from any given object are such as they are actually calculated to yield, the process of deception is extremely easy, and it may be successfully executed even if the angle between the real and the supposed directions of the sound is much greater than the angle of uncertainty. Mr. Dugald Stewart has stated some cases in which deceptions of this kind were very perfect. He mentions his having seen a person who, by counterfeiting the gesticulations of a performer on the violin, while he imitated the music by his voice, rivetted the eyes of his audience on the instrument, though every sound they heard proceeded from his own mouth. The late Saville Carey, who imitated the whistling of the wind through a narrow chink, told Mr. Stewart that he had frequently practised this deception in the corner of a coffee-house, and that he seldom failed to see some of the company rise to examine the tightness of the windows, while others, more intent on their newspapers, contented themselves with putting on their hats and buttoning their coats."

The actual condition of the vocal organs in the exercise of ventriloquism is certainly ill understood. Let us hear what Sir David has to say.

"Ventriloquism loses its distinctive character if its imitations are not performed by a voice from the belly. The voice, indeed, does not actually come from that region, but when the ventriloquist utters sounds from the larynx without moving the muscles of his face, he gives them strength by a powerful action of the abdominal muscles. Hence he speaks by means of his belly, although the throat is the real source from which the sounds proceed. Mr. Dugald Stewart has doubted the fact, that ventriloquists possess the power of fetching a voice from within; he cannot conceive what aid could be derived from such an extraordinary power; and he considers that the imagination, when seconded by such powers of imitation as some mimics possess, is quite sufficient to account for all the phenomena of ventriloquism which he has heard. This opinion, however, is strongly opposed by the remark made to Mr. Stewart himself by a ventriloquist, 'that his art would be perfect if it were possible only to speak distinctly without any movement of the lips at all.' But, independent of this admission, it is a matter of absolute certainty, that this internal power is exercised by the true ventriloquist. In the account which the Abbé Chapelle has given of the performances of M. St. Gille and Louis Brabant, he distinctly states that M. St. Gille appeared to be absolutely mute while he was exercising his art, and that no change in his countenance could be discovered.* He affirms also that the countenance of Louis Brabant exhibited no change, and that his lips were close and inactive. M. Richerand, who attentively watched the performances of M. Fitz-James, assures us that during his exhibition there was a distention in the epigastric region, and that he could not long continue the exertion without fatigue."

There is an interesting chapter on the various mechanical attempts which have been made to imitate the voices of animals and of man. M. Vaucanson's flute-player and tabor-player were extremely ingenious automata, but a talking one, if perfected, would be infinitely more astonishing. Sir David, indeed, has no doubt that "before another century a *talking* and a *singing machine* will be numbered among the conquests of science." M. Recupelen actually constructed an automaton which could pronounce words, and even sentences, as *opera*, *astronomy*, *venez avec moi à Paris*, *&c*. It is curious to observe the effects of great elevations and the re-

* "Edinburgh Journal of Science, No. xviii. p. 254."

verse on sound, and in consequence on the human voice.

"In the region of common life, and even at the stillest hour of night, the ear seldom rests from its toils. When the voice of man and the bustle of his labours have ceased, the sounds of insect life are redoubled, the night-breeze awakens among the rustling leaves, and the swell of the distant ocean, and the sounds of the falling cataract or of the murmuring brook, fill the air with their pure and solemn music. The sublimity of deep silence is not to be found even in the steppes of the Volga, or in the forests of the Orinoco. It can be felt only in those lofty regions,

Where the tops of the Andes
Shoot soaringly forth.

As the traveller rises above the limit of life and motion, and enters the region of habitual solitude, the death-like silence which prevails around him is rendered still more striking by the diminished density of the air which he breathes. The voice of his fellow-traveller ceases to be heard even at a moderate distance, and sounds which would stun the ear at a lower level make a feeble impression. The report of a pistol on the top of Mont Blanc is no louder than that of an Indian cracker. But while the thinness of the air thus subdues the loudest sounds, the voice itself undergoes a singular change: the muscular energy by which we speak experiences a great diminution, and our powers of utterance, as well as our power of hearing, are thus singularly modified."

Dr. Colladon made the reverse experiment in descending in the diving-bell at Howth in 1820.

"We descended," says he "so slowly that we did not notice the motion of the bell; but as soon as the bell was immersed in water, we felt about the ears and the forehead a sense of pressure, which continued increasing during some minutes. I did not, however, experience any pain in the ears; but my companion suffered so much that we were obliged to stop our descent for a short time. To remedy that inconvenience, the workmen instructed us, after having closed our nostrils and mouth, to endeavour to swallow, and to restrain our respiration for some moments, in order that, by this exertion, the internal air might act on the Eustachian tube. My companion, however, having tried it, found himself very little relieved by this remedy. After some minutes, we resumed our descent. My friend suffered considerably; he was pale; his lips were totally discoloured; his appearance was that of a man on the point of fainting; he was in involuntary low spirits, owing, perhaps, to the violence of the pain, added to that kind of apprehension which our situation unavoidably inspired. This appeared to me the more remarkable, as my case was totally the reverse. I was in a state of excitement resembling the effect of some spirituous liquor. I suffered no pain; I experienced only a strong pressure round my head, as if an iron circle had been bound about it. I spoke with the workmen and had some difficulty in hearing them. This difficulty of hearing rose to such a height, that during three or four minutes I could not hear them speak. I could not, indeed, hear myself speak, though I spoke as loudly as possible; nor did even the great noise caused by the violence of the current against the sides of the bell reach my ears."

Sir David has given some curious details of remarkable feats of strength, which have been exhibited at different times by individuals of extraordinary physical powers. Of these the most astonishing was a man named Topham. Dr. Desaguliers, who minutely observed his feats, has calculated that the strength of the weakest men being 125lbs. and that of very strong men 400, the strength of Topham was 800; his weight was 200.

The following statements appear nearly incomprehensible to us, and although we casually heard the same assertions made in mixed company many months ago, we have never investigated their truth or fallacy.

"One of the most remarkable and inexplicable experiments relative to the strength of the human frame, which you have yourself seen and admired, is that in which a heavy man is raised with the greatest facility, when he is lifted up the instant that his own lungs and those of the persons who raise him are inflated with air. This experiment was, I believe, first shown in England a few years ago by Major H. who saw it performed in a large party at Venice under the direction of an officer of the American Navy. As Major H. performed it more than once in my presence, I shall describe as nearly as possible the method which he prescribed. The heaviest person in the party lies down upon two chairs, his legs being supported by the one and his back by the other. Four persons, one at each leg, and one at each shoulder, then try to raise him, and they find his dead weight to be very great, from the difficulty they experience in supporting him. When he is replaced in the chair, each of the four persons takes hold of the body as before, and the person to be lifted gives two signals by clapping his hands. At the first signal he himself and the four lifters begin to draw a long and full breath, and when the inhalation is completed, or the lungs filled, the second signal is given, for raising the person from the chair. To his own surprise and that of his bearers, he rises with the greatest facility, as if he were no heavier than a feather. On several occasions I have observed that when one of the bearers performs his part ill, by making the inhalation out of time, the part of the body which he tries to raise is left as it were behind. As you have repeatedly seen this experiment, and have performed the part both of the load and of the bearer, you can testify how remarkable the effects appear to all parties, and how complete is the conviction, either that the load has been lightened, or the bearer strengthened by the prescribed process.

At Venice the experiment was performed in a much more imposing manner. The heaviest man in the party was raised and sustained upon the points of the fore-fingers of six persons. Major H. declared that the experiment would not succeed if the person lifted were placed upon a board, and the strength of the individuals applied to the board. He conceived it necessary that the bearers should communicate directly with the body to be raised. I have not had an opportunity of making any experiments relative to these curious facts; but whether the general effect is an illusion, or the result of known or of new principles, the subject merits a careful investigation."

This notice, for it is neither review nor analysis, must draw to a close—indeed we have extracted all the more interesting parts which bear upon medical science. The following facts respecting the capability of resisting heat are worth transcribing.

"But even when the fluid requires a high temperature to boil, it may have other properties, which enable us to plunge our hands into it with impunity. This is the case with boiling tar, which boils at a temperature of 220°, even higher than that of water. Mr. Davenport informs us, that he saw one of the workmen in the King's Dock-yard at Chatham immerse his naked hand in tar of that temperature. He drew up his coat sleeves, dipped in his hand and wrist, bringing out fluid tar, and pouring it off from his hand as from a ladle. The tar remained in complete contact with his skin, and he wiped it off with tow. Convinced that there was no deception in this experiment, Mr. Davenport immersed the entire length of his fore-finger in the boiling cauldron, and moved it about a short time before the heat became inconvenient. Mr. Davenport ascribes this singular effect to the slowness with which the tar communicates its heat, which he conceives to arise from the abundant volatile vapour which is evolved 'carrying off rapidly the caloric in a latent state, and intervening between the tar and the skin, so as to prevent the more rapid communication of heat.' He conceives also, that when the hand is withdrawn, and the hot tar adhering to it, the rapidity with which this vapour is evolved from the surface exposed to the air cools it immediately. The workmen informed Mr. Davenport, that, if a person put his hand into the cauldron with his glove on, he would be dreadfully burnt, but this extraordinary result was not put to the test of observation.

But though the conjurors with fire may have availed themselves of these singular properties of individual bodies, yet the general secret of their art consisted in rendering the skin of the exposed parts callous and insensible to heat,—an effect which may be produced by continually compressing or singeing them till the skin acquires a horny consistence. A proof of this opinion is mentioned by Beckmann, who assures us that in September 1765, when he visited the copper works at Awestad, one of the workmen, bribed by a little money to drink, took some of the melted copper in his hand, and after shewing it to the company, threw it against a wall. He then squeezed the fingers of his horny hand close to each other, held it a few minutes under his arm-pit to make it perspire, as he said, and taking it again out, drew it over a ladle filled with melted copper, some of which he skimmed off, and moved his hand backwards and forwards very quickly by way of ostentation. During this performance, M. Beckmann noticed a smell like that of singed horn or leather, though the hand of the workman was not burned. This callosity of the skin may be effected by frequently moistening it with dilute sulphuric acid. Some allege that the juices of certain plants produce the same effect, while others recommend the frequent rubbing of the skin with oil. The receipt given by Albertus Magnus for this purpose was of a different nature. It consisted of a non-conducting calcareous paste, which was made to adhere to the skin by the sap of the marsh-mallow, the slimy seeds of the flea-bane, and the white of an egg."

It appears that Mr. Chantry, the eminent sculptor, has recently exposed himself to heated air at a temperature considerably exceeding that encountered by Sir Joseph Banks and Sir Charles Blagden in their celebrated experiments. The temperature of the air in the room which they entered was, at the highest, 260°. The furnace employed by Mr. Chantry for drying his moulds is about 14 feet long, 12 feet high, and 12 feet broad. When raised to its highest temperature, with the doors closed, the thermometer stands at 350°, and the iron floor is red hot. The workmen often enter it at a temperature of 340°, walking over the iron floor with wooden clogs, which are of course charred on the surface. On one occasion Mr. Chantry, accompanied by five or six of his friends, entered the furnace, and, after remaining two minutes, they brought out a thermometer which stood at 320°. Some of the party experienced sharp pains in the tips of their ears, and in the septum of the nose, whilst others felt a pain in their eyes.

Here we conclude. We think it unnecessary to add any formal recommendation of this little volume, which combines amusement with instruction, and is equally adapted for youthful intelligence, and for the leisure of those whose years are riper.

---

### XXV.

### Death from Injection of an Encysted Dropsy.

A girl had been tapped by M. Dupuy five times for encysted abdominal dropsy. On the sixth time he proceeded a step further, indeed a step too far. After having made a puncture he injected through a gum-elastic tube the vapour of heated wine. Inflammation succeeded, extended to the abdomen, and in thirteen days the girl died. No cadaveric examination was permitted, but on puncturing the abdomen a large quantity of fetid yellow matter escaped. —*Journal de Bourdeaux, Dublin Journ. July*, 1832.

---

### XXVI.

### Fungous Tumors of the Dura Mater and of the Bones of the Skull.

Our contemporary, the Dublin Journal, gives an abstract of a memoir on these tumors, presented to the Academy of Sciences in February 1832, by M. Chelius of Heidelberg. As the notice is brief, and insusceptible of further condensation, and as we have a remark or two to offer upon it, we shall quote it entire.

"M. Chelius lays it down, that the fungous tumours of the dura mater and those of the bones of the cranium are not the same disease; they differ in their seat and in their nature. He cites several cases to support his opinion: 1st. A soldier, aged 36 years, was attacked by slight pain in the arm, at the insertion of the deltoid. This pain gradually increasing, became at length intolerable, and yet the form of the limb was not altered: soon after, the movements of the arm became different, and the pain made him keep it constantly at rest. This state of things lasted six weeks, when by accident, the arm was luxated; the dislocation was reduced, and the shoulder covered by compresses steeped in oxycrate, &c. There then appeared at the tip of the shoulder a swelling which soon extended to the fingers. Some months afterwards, there appeared at the middle of the forehead, a circumscribed tumour of the size of a pea, affected by occasional lancinating pains. When this tumour had grown to the size of a pea, it was found to proceed through an opening in the skull, and to pulsate. From this time the two tumours continued to increase in size, and violent nasal hemorrhages frequently came on, after one of which the patient died, twenty months after the supervention of the first pain in the arm. The aorta having been previously injected, the temporal, frontal, superciliary and nasal arteries were found to be twice as large as they naturally are, and were more tortuous, and had more ramifications, all of which penetrated the tumour. This last was situated on the surface of the dura mater; below it the brain was healthy but depressed; the tumour had no adhesion to the bones of the skull, it was united to the dura mater and to the pericranium by numerous vessels, which, arising from these membranes, penetrated its tissue. It consisted of a pulpy mass, yellowish white, similar to marrow, in which existed a few small scattered vessels, and small, irregular, and dentilated spiculæ; at its centre there was a more consistent steatomatous mass, which contained still less vessels. The frontal bone presented an opening two inches in diameter externally, and four lines more internally, the ethmoid, unguis, the horizontal portion of the frontal, and the base of the orbital cavity, had been destroyed to the depth of a line and half. The tumour penetrated into the two orbits, completely enveloping the oblique nerves, and extending into the nasal cavity, where the septum had been partly destroyed, and finally arrived to within half an inch of the external opening of the nose. The tumour of the right arm was of the size of a child's head; formed in the same way, of a pulpy substance externally, and a hard steatomatous mass at the centre, with many small cavities filled with serous fluid. The humerus in its upper portion was destroyed; inferiorly where it corresponded to the tumour, it was totally deprived of periosteum, but he could not trace any organic connexion between it and the tumour. M. Chelius thinks, that in this case the disease commenced in the periosteum and dura mater, and that the bones continued free from any injury by the disease, and were destroyed only by the pressure made on them by the tumours. 2nd. A woman who always had been accustomed to labour in the fields, at the age of 50 adopted a sedentary mode of life. At the age of 54 she began to complain of continual somnolence and severe headach; these symptoms increased, and soon after a small tumour appeared on the forehead. This at first was not painful, but gradually enlarging, it produced frequent faintings and constant sneezing; it finally extended over the whole forehead, covered the right eye, and confounded itself with the parotid gland; it was hard in some places, and soft in others, with fluctuation in its centre. The woman finally died, with all the symptoms of compression of the brain. The opening of the body showed that the tumour was situated between the pericranium and bones of the skull. The pericranium was so dense and so adherent to the bone that they could not be separated without laceration. The skull was not perforated, but presented some small openings, scarcely capable of admitting a very fine probe. 3rd. A man aged 30 years, who had been scrofulous in his youth, and often attacked by venereal, had on the middle of his forehead a tumour, which rapidly grew as large as a hen's egg; it was firm, without any trace of pulsation or of osseous perforation around its base. In time the tumour grew until it occupied the left side of the forehead, the root of the nose, and the orbit, from which the eye was expelled. The patient suffered dreadful headaches, became weak, and finally constantly comatose. After death, it was found that the fungous tumour was connected on one side to the dura mater, and on the other to the pericranium; in some parts the corresponding bones of the skull had undergone sarcomatous degeneration; so that M. Chelius considers this case to belong to the sarcomatous affections of the bones.—*Archives de Medicine, Mars*, 1832."

Now we do not see that the foregoing cases prove the assertion of M. Chelius that the fungous tumours of the dura mater differ in their nature from those of the skull. To tell us that they differ in their seat is merely to tell us an obvious truism, but surely the other assertion to which we have alluded should be borne out by something like argument, of which we have none, or by obviously apposite facts, which are equally deficient. In order to prove the broad statement with which M. Chelius sets out, we should have cases of tumours of both descriptions placed side by side, compared, and shewn by rigorous analysis and comparison to differ. Is this done—is it even attempted? It is not.

So far as we have seen, and several instances of the kind have fallen under our observation, there is no essential difference between the fungous tumours of the skull and those of the dura mater. Fungous tumours on the head may spring from the pericranium, from the cranial diploë, from the dura mater, or from the brain and cerebellum. The tumours generally partake more of the character of fungus hæmatodes than of those of scirrhus, characters which we need not enumerate. Analogy is not proof, yet analogical reasonings are frequently calculated to elicit truth and defeat error. When we look at the composition of malignant tumours elsewhere, do we not find that fungus hæmatodes invades almost every tissue, that its organic structure suffers comparatively small alterations from the influence of tissue, and, to come more closely to the point in dispute, that the differences between fungus of bone and fungus of fibrous membrane are very unimportant indeed? From these considerations, and still more from actual observation of fungous tumours of the dura mater and of the cranium, we do not hesitate to express our opinion that, so far we can form a just one, there is no essential difference between them. At all events, we cannot allow that the assertions of M. Chelius are proofs, or that his proofs are calculated to confirm his assertions.

---

### XXVII.

### Composition of Cow's Milk before and after Parturition.*

M. Lassaigne has made some experiments on this subject; the results are contained in the following short summary.

Forty-two days before calving, the milk was yellowish-white, sp. gr. 1063, coagulated by heat, restored the colour of reddened litmus, and contained 78.4 per cent. of water.

One thousand parts contained, cream, 200; serum, 800.

There was much albumen and free soda, but no trace of caseum, of sugar of milk, or of lactic acid.

This state continues until about eleven days before calving, when the specific gravity was only 1040; the

* Annales de Chimie. Dublin Journ. July.

milk was much less coagulable, and had lost its alcaline re-action. The proportion of cream and serum remained unaltered. The albumen had nearly disappeared, and the caseum and sugar of milk, with lactic acid, were found to exist.

Four days after parturition, all trace of albumen had disappeared, sp. gr. 1035; otherwise same.

The only important alteration that afterwards takes place is the gradual diminution in the quantity of fatty matter.

On the sixth day after calving, there were in 1000 parts—

| | Serum. | Cream. |
|---|---|---|
| | 812 | 188 |
| 20th day........ .... | 922 | 78 |
| 30th day............ | 936 | 64 |

---

XXVIII.

### M. RODWEISS ON URIC ACID AND ITS DECOMPOSITION BY NITRIC ACID.

MANY analyses of uric acid have been made and they disagree, like most other analyses, among each other. M. Rodweiss has endeavoured to determine the most accurate, and obtained results different from all. Much as the uncertainty of medicine and disagreements of doctors have been the burden of the wit's jest and the philosophic sneer, we doubt if the uncertainties of chemistry are not equally great, though not equally palpable to the lettered and unlettered dunce.

The mean of M. Rodweiss' experiments gave for the composition of uric acid,

4 atoms azote. 3 atoms hydrogen.
10 —— carbon. 4 —— oxygen.

Atomic weight 153.1—capacity of saturation 5.298.

M. Rodweiss then formed and examined the purpuric acid of Prout, Vauquelin, Brugnatelli, and Quesnaville. His experiments prove that the acid of Prout, carefully prepared, is really a peculiar acid, having the property of producing a red solution with ammonia, and is not, as was advanced by Vauquelin, a combination of acid and colouring matter. The acid prepared by the process of Vauquelin is really a mixture of oxalic and purpuric acids. The erythric acid of Brugnatelli is, according to M. Rodweiss, a crystallizable combination of nitric acid and of purpuric acid, and this compound is procured also by the process of Quesnaville.—*Ibid.*

---

XXIX.

### VITREOUS HUMOUR REGENERATED.

A SERJEANT in the Garde Royale received an injury on the left eye which burst the globe and expelled the crystalline, vitreous, and aqueous humors. The palpebræ were ecchymosed; the conjunctiva red and bloated; and the lower part of the cornea was separated from its attachment to the sclerotic by an irregular cut, through which a portion of the iris protruded. The parts were washed, the iris carefully returned, the edges of the cornea approximated, the eyelids closed, and blood was drawn from the temporal artery. Ice to the head, sinapisms to the feet, constant darkness, and a vigorous diet were enjoined. During the night he was bled again, and next day cupping-glasses were applied between the shoulders. It was supposed that vision would be for ever lost, but *à notre très-grande et agréable surprise* the globe gradually refilled; on the 22d day the cornea began to assume a healing aspect, and in about six weeks it was perfectly cicatrized. The natural form, and almost the ordinary volume of the organ, were restored, and by the aid of a very convex glass, this soldier came to see objects distinctly, and continues to discharge all the duties of his office.—*Larrey's Clinique Chirurgicale—Glasg. Journ. Feb.* 1832.

---

XXX.

### CASES OF VESICO-VAGINAL AND RECTO-VAGINAL FISTULA CURED.

Two cases of this dreadful deformity, in which operations were crowned with success, have been lately published.

Such cases are always valuable, because they encourage surgeons to persevere in attempts at removing loathsome and almost unmanageable ills, when they might be utterly disheartened by failures by far too numerous.

*Case* 1.* Mrs. R. æt. 22, had laboured under vesico-vaginal fistula since her first confinement, which had been protracted and severe; the finger passed readily through the fistula into the bladder, and for eight months many plans had been tried without success. M. Malagodi operated in the following manner.

Having placed the patient as if for lithotomy, he introduced the fore-finger of the right hand, covered with the finger of a glove, into the fistulous opening, and bending it, drew down the left border of the fistula to the orifice of the vagina; and then, with the other hand, he pared off the edges with a straight bistoury. The hand was changed, and the same operation repeated on the right border of the fistula. The pared edges were then brought in contact. To do this M. Malagodi reintroduced the right index finger into the opening, and brought down its left border, and with the left hand passed a small curved needle, fastened with a ligature, through it, near the posterior angle of the wound. A second and third needle were passed in the same manner, at equal distances from each other, and the opposite border was transfixed in the same manner, when the ligatures were tied and the sides of the opening thus kept together. The patient was placed on her back in bed, a catheter kept in the bladder, and the urine allowed to flow constantly through it. Until the morning of the third day the water was passed through the catheter only, but then it began to escape by the wound into the vagina. On the fourth day it was found that the two posterior sutures remained firm; and on withdrawing them the union of the sides of the fistula was complete. The anterior ligature, on the other hand, had torn the left border of the wound, and nearly a third of the original fistula thus remained unhealed. The edges of this were regularly touched with the nitrate of silver, the catheter being kept constantly in the bladder. This plan was completely successful at the end of a few weeks.

*Case* 2.* Isabella Pigot, æt. 28, was admitted into the London Hospital, Dec. 2, 1830, with vesico-vaginal fistula. The opening between the vagina and neck of the bladder was sufficiently large to admit the point of a fore-finger, and seemed to involve a small portion of the commencement of the urethra; it was oval, its long diameter lying transversely and about 1¼ inch from the orifice of the meatus urinarius. The flow of urine was constant but the retentive power of the bladder was not wholly destroyed. The edges of the aperture were callous to only a slight extent. She stated that four months previously she had been delivered of a still-born child after a protracted labour, in which the bladder had not been relieved. Her recovery was speedy, but since her confinement she had been unable to retain her urine.

On the 29th Dec. Mr. Luke proceeded to perform the following operation. The patient being placed as for lithotomy, a female catheter was introduced into the bladder through the meatus urinarius. Weiss's two-branched dilator of the female urethra was then passed per vaginam, and fully opened, by which the fistulous orifice was exposed. Attempts were made to pass tenacula through the orifice but they were found too little curved, and two hooks were therefore employed. Their points were introduced about an eighth of an inch from the internal side of the opening, and brought out at the same distance on the external, when the sides were next pared by two semi-elliptical incisions made transversely in the vagina; to make the internal it was necessary

* Raccoglitor Medico. Glasgow Journal, Feb.

* Medical Gazette.

to use a scalpel, with one half the blade bent at a right angle with the other. There was now some difficulty experienced in passing the ligatures for closing the wound. Small curved needles held in a porte-aiguille would not answer. Mr. L. succeeded with larger ones less curved, passing one on each side of the urethra at a little distance from the edge of the wound; the ligatures when tightened perfectly closed the orifice. The dilator was withdrawn, and an elastic catheter secured in the urethra; the patient was then sent to bed, and ordered to lie on her face and allow the urine to drain on a cloth.

No water passed by the wound till the 1st January, when the ligatures were found to have ulcerated from one side, and the edges of the wound to be so separated as to allow the escape of the whole of the urine. On the 3d the opening was larger than before the operation; the ligatures were removed, the catheter allowed to remain. 6th. Elastic catheter being stopped up was withdrawn. Another ligature was passed in the centre of the opening including more substance than before; the silver flat catheter was fastened in and the patient placed on her face. On the 11th the ligatures having produced ulceration were removed, but the wound still adhered; a clean catheter was introduced. 15th. Catheter has become stopped; wound re-opened and nearly of former size.

*March* 19*th*. Mr. L. pared the edges of the opening by carrying a single hook through the sides and excising them with a common scalpel; a single ligature kept them in contact. The patient was placed on her face and a catheter introduced as before. On the 26th, the ligature having produced ulceration was removed; the sides of the opening were adherent, and all the urine flowed by the catheter, which was allowed to remain. On the 9th April the catheter was removed; the fistulous opening was perfectly closed. A stillicidium urinæ remained for a short time, but as the parts regained their tone it subsided. On the 25th she was discharged cured.

*Case* 3.* An Irish woman, æt. 30, was admitted into the London Hospital on the 24th May, under the care of Mr. Scott. A fistulous communication was found to exist between the rectum and vagina, and the fæces escaped through it into the latter. Her first labour had occurred about ten weeks previously, and during a severe pain whilst the child's head was in the vagina, the attending midwife passed the hand into the vagina, at which time the patient felt something give way.

The bowels having previously been freely opened Mr. Scott operated on the 13th June. He commenced by pulling down the vagina with a double hook, then pared off the cartilaginous edges with a probe-pointed bistoury, brought the raw edges together, and retained them in contact by means of three sutures. She was strictly confined to slops that the parts might not be disturbed by the occurrence of motions. During the night she passed her urine thrice and rested well. On the following night she was very unwell and fainted twice. On the 17th she was ordered some castor oil by the dresser, which was followed by a motion and the passage of a part of the fæces by the vagina. On the 26th Mr. Scott made an examination of the parts. The edges of the opening had united throughout nearly their whole extent, a small ununited portion only being left through which some feculent matter passes when she has a motion. This is the last report, and consequently the result of the operation is not yet determined.

There is one circumstance to be recollected in perusing such cases as the foregoing. The recto-vaginal, or vesico-vaginal fistula is usually the consequence of labour, and although a temporary cure be obtained by operations, the patients are liable to future pregnancies. We must not then consider the case as concluded when the female leaves the hospital cured. Future inquiries should determine whether that cure is permanent. Unfortunately such

* Lancet, July 21, 1832.

inquiries are too often neglected, or even when made, the issue, if unfortunate, is not published so eagerly as the former seeming success.

---

XXXI.

TIC DOULOUREUX OF A STUMP SAID TO BE CURED BY THE REMOVAL OF A PORTION OF THE MEDIAN NERVE.*

THERE is one disadvantage attending hospital reports as at present managed. The reporter, whoever he may be, is usually in such a hurry to open his budget, that he will not stop to learn the issue of a case before he gazettes it as a cure. The consequence is that patients have been dead or dying, at the very period when the success of the treatment employed has been going the rounds of the medical journals. If the result is afterwards made known it is done so in a parenthesis, and escapes many who read the former statement. We do not say that the case we are about to notice is an example of this fault, and yet we suspect that the narrator is scarcely justified in setting it down at present as an instance of cure from a particular operation. We had false facts enough in medicine, before the modern system of hospital reporting opened a fresh vein of the poisonous ore.

*Case.* Eliza Burkett, æt. 20, was admitted into the London Hospital, April 21, under the care of Mr. Luke.

"She stated that about three years and a half since, she was taken into St. Thomas's Hospital in consequence of severe injury to the left hand, occasioned by a fall. Every attempt to save it having been made, after two years amputation became necessary, on account of her general health suffering considerably from the excessive suppuration and painful sensations in the arm; the stump did not heal favourably; she suffered distressing agony, and her health again became affected. She was discharged from the hospital, and afterwards admitted into Cripplegate workhouse, under the care of Mr. Langstaff.* At that time the surface of the stump presented an unfavourable appearance; the skin covering the ends of the radius and ulna was very thin, excessively vascular, and the ends of these bones seemed likely to cause its absorption. There was also a constant state of convulsive action of the muscles of the stump, accompanied with agonizing pain. Poulticing, opiate lotions, belladonna, and gentle pressure by bandage, were employed to lessen her sufferings, but without any good effect.

Mr. Langstaff afterwards performed amputation above the elbow-joint by the flap operation; and previously to securing the arteries he drew out each nerve to the extent of half an inch from the surface of the stump with a tenaculum, and cut through them to prevent their interrupting the progress of cicatrization of the integumental parts. For some time she was relieved of all the painful sensations she had so long been distressed with; had no recurrence of hysteria or convulsion, and her health improved.

About two months after the last operation she applied at this hospital, complaining of severe pain in the stump, the extremity of which was inflamed and affected with spasmodic twitchings; she was constantly subject to headache, and was unable to obtain rest night or day; altogether her life was rendered so miserable that if relief could be procured, she was willing to undergo amputation at the shoulder-joint. The carbonate of iron, iodine, acetate of morphia, blisters in the axilla, leeches to the extremity of the stump, cupping at the nape of the neck, and various other remedies were employed, but without any beneficial effect, except the cup-

* Medical Gazette, July 13, 1832.

* "The early history of the case is partly taken from a paper by Mr. Langstaff, on the Healthy and Morbid condition of Stumps after Amputation, in the Medico-Chirurgical Transactions."

ping, which partly relieved the pain in the head. She continued her attendance as an out-patient for about four months, subsequently to which an exfoliation of bone took place, and she applied at St. Bartholomew's Hospital, where Mr. Earle cut down upon the extremity of the stump. She returned to this hospital without any diminution of her sufferings; and after attending a short time, Mr. Luke having ascertained that the pain proceeded from that part of the stump where the median nerve appeared to terminate, proposed to remove a portion of the nerve, to which operation she consented.

April 21st.—Mr. Luke made an incision in the course of the median nerve just below the axilla; and, after a little dissection, exposed and removed nearly half an inch. The woman thought that something was pulled, and complained of a sensation of numbness at the extremity of the stump: she immediately expressed herself entirely relieved from the pain, and could bear firm pressure upon it. In performing the operation, a small artery was divided, which required a ligature."

Some pyrexia succeeded and required saline medicines and venesection. Retention of urine succeeded, and she requires to have her water drawn off twice daily. The last report is dated June 12th, not two months from the performance of the operation, and it states that the wound is healed, that she is entirely free from pain, but that the retention of urine continues.

Now there are two considerations which induce us to think the reporter premature in giving this as a case of cure. In the first place, the period passed without pain, after the amputation performed by Mr. Langstaff, was longer than what has elapsed between the report and the operation on the median nerve, and yet we have seen that the former pause was deceptive, and that the disease returned. In the second place, it seems evident to us, from several circumstances, that the young woman is of an excitable, nervous, or hysteric habit, and we all know how difficult it is to treat such with success. We do not say that the above was a case of hysteria only, but we do say that there is much of that undefinable state of system mixed up with it, and that probably surgical operations will not put that out. However, be this the fact or be it not, we conceive the reporter hasty in publishing the case as one of cure.

In the same report which contains the preceding is a case of strangulated direct inguinal hernia. It was intestinal, had appeared suddenly three days previously after a strain; there was no fluid in the sac, and the stricture was very tight. We see no other features of great interest in the report. The man recovered, but an abscess formed just above the wound, and a considerable quantity of the superficial fascia sloughed.

In a case of stone impacted in the neck of the bladder, or rather in the prostatic part of the urethra, Weiss's instrument for the extraction of small calculi was ineffectually employed. An abscess formed in the perineum after its use. A staff was introduced into the urethra, and the stone being distinguished, by the finger in ano, to the right side of the perinæum and anterior to the prostate, it was cut down on and easily removed. The man sank about the sixth day after the operation. On dissection there was effusion of semi-purulent fluid into the cavity of the abdomen; a sloughy state of the fundus of the bladder; extensive suppuration of one kidney, with a calculus impacted in one of the calyces; softening of the other kidney; and dilatation of the ureters of both; the bladder thickened, and its mucous membrane inflamed and coated with lymph; and lastly, an abscess on the side of the prostate communicating with the urethra, and abscess in the perinæum. The patient stated, after the operation, that he had been operated on by Mr. Cline thirty years previously, when he was only eight years of age.

This case shews how necessary it is for the practitioner to make himself acquainted with the nature and causes of the alterations of the urine, in fact, with the connexion between them and the state of the urinary organs. In the present instance, the situation of the calculus and symptoms produced by it rendered it almost imperative on the surgeon to attempt its removal, yet that attempt was the immediate cause of death; and, under other circumstances, the performance or non-performance of an operation might make the difference of life or destruction to the patient. It is manifest that, with such structural alterations of the kidneys and bladder as existed in this case, there *must* necessarily be alterations in the character of their secretions, and in the working of their functions. Such alterations may or may not be apparent; that is not the question. It is certain that, if we carefully study the subject, we are less likely to be deceived, than if we give it the superficial examination it usually receives, and although at first the difficulties may seem great, they will be diminished, at all events, the further we proceed. We have already, on several occasions, recommended Dr. Prout's scientific work to the attention of the profession, and again seize the opportunity of repeating that recommendation.

---

### XXXII.

### Dr. Watson on Hæmaturia.

In one of the Lumleian lectures, which Dr. Watson has recently been delivering before the College of Physicians, there are some remarks on hæmaturia, which may be noticed.

Blood may proceed from any part of the secreting or excreting urinary apparatus—from the kidney, ureter, bladder, or even urethra, in consequence of stone—from the kidney or bladder, in consequence of malignant disease—from any part of the apparatus, in consequence of inflammation—from some determination to these organs, arising without obvious or definable cause, or occurring periodically or vicariously with the hæmorrhoidal flux, or in scurvy and purpura hæmorrhagica, or in some cases of dropsy, especially that occurring after scarlet fever, or when excessive venery has been indulged in. These, and probably some others, are causes of hæmaturia, and in determining which has operated in the individual case, consists the tact and sagacity of the practitioner.

Dr. Watson mentions the following instance of hæmaturia from one of the causes alluded to. A young man came to the Middlesex Hospital with hæmorrhage from the urethra, and said that he had lost a considerable quantity of blood in this manner in the course of a few hours. He stated that he had passed the previous night with a female in whom the catamenia had just returned, and he imagined himself inoculated with the flux. The bleeding was owing, no doubt, to rupture of a vessel or vessels in the excited urethra. We have seen an instance of the same kind and apparently owing to the same cause, but the patient had just recovered from severe gonorrhœa and inflammation of the mucous membrane of the bladder.

Blood in the urine communicates to it a red tinge, more or less deep, and by that tinge its presence is determined. But other substances and conditions of the system colour the urine. The prickly pear or Indian fig will do it. The Spaniards, on their discovery of America, eat largely of this fruit, and were alarmed by the consequence. Ellicot's people, in his travels for determining the boundary of the United States, ate plentifully of the same fruit, with the same result and the same alarm. Beet-root produces a simlar effect, and a curious instance of red-coloured urine, in a man accustomed to take beet-root for supper, is related by Dessault. Madder, some species of strawberries, and drinks of sorrel, are said to do the same. The simplest and most satisfactory criterion of blood in the urine, is afforded by raising the fluid to the boiling temperature; the blood will furnish a brown coagulum, while the rest will regain the natural colour of urine.

Let us glance at the diagnostic marks of hæmaturia, having its origin in the various parts on the conditions before mentioned.

Dr. Prout has said, that "blood derived from the kidney is generally equally diffused through the whole urine: derived from the bladder, it for the most part comes away, in greater or less quantity, at the termination only of the urinary discharge, the urine having previously flowed off nearly pure. With all due submission to Dr. P. we think this must depend in a great degree on circumstances; and, from what we have seen, we would not care to attach great importance to the distinction. There is one appearance, which, when present, is characteristic of hæmorrhage from the kidney, or commencement of the ureter—viz. the expulsion with the urine of small cylindrical portions of fibrinous coagulum, the moulds of the ureter; they are commonly whitish, and often look like slender maggots or small worms. Dr. Cullen says that he saw coagulum come away almost dry, like the half-burnt wick of a candle. The attendant symptoms of hæmorrhage from the kidney or commencement of the urethra are, probably, a sensation of heat, weight, or pain in the situation of the kidney, or one side of the body. If a calculus has previously descended from the kidney, or if the symptoms usually characterizing such a descent be present, the diagnosis is made still stronger. But if these kidney symptoms be absent, and those of stone, or inflammation of it, or diseased prostate be present, we may presume that the bladder is the seat of the hæmorrhage.

Dr. Prout thinks we may pronounce, with much confidence, on the existence of malignant disease, by a reddish sediment which the urine throws down, the characters of which are difficult of description, but strike the experienced eye.

The hæmorrhage occasionally accompanying strangury from turpentine or cantharides, is probably, from the character of the symptoms, the result of some inflammatory action. M. Renoult describes a troublesome hæmaturia which affected the French troops in Egypt, especially the cavalry, and even their horses. There was much pain in the situation of the bladder, extending to the glans penis—frequent and urgent desire to make water, the last drops being often pure blood, and passed with acute pain. On examining the bladder after death, M. Renoult found its mucous membrane inflamed.

A diseased bladder will give rise to hæmorrhage, as all practical surgeons know. A curious case happened to Mr. Heaviside. An old East Indian, subject to nephritic complaints, was suddenly siezed with what seemed to be retention of urine. A catheter was passed, no urine flowed, and it was thought the instrument had not entered the organ, in the seat of which was an evident tumor. The patient died next day. The bladder was found distended with a large coagulum of blood, which had come from the diseased mucous membrane; there was no blood in the kidneys or ureter.

When pure blood comes away, it is probably from the urethra.

In many cases of hæmaturia, the symptoms are very indecisive, the causes very obscure. In such, Dr. Watson thinks the hæmorrhage usually renal, and probably he is right. Calculi may exist in the kidney, as every body knows, without giving rise to obvious symptoms, and may there give rise to hæmorrhage.

The expulsion of blood in hæmaturia is sometimes attended with rigors, as the passage of a bougie, of a small stone, &c. often is. Dr. Elliotson had a case of intermittent hæmaturia occurring in a person who had formerly had a Walcheren fever; it always accompanied the cold fit of an ague, and was cured by quinine.

Sometimes the blood coagulates in the bladder, and gives rise to much inconvenience, or even forms the nucleus for stone. It is to be suspected when, after the recent passage of blood by the urethra, the patient continues to void brown or dark urine, depositing a chocolate-coloured sediment.

This is the substance of Dr. Watson's remarks on hæmaturia.

---

### XXXIII.

### Two Lectures on the Primary and Secondary Treatment of Burns.

By Henry Earle, F.R.S. Surgeon to St. Bartholomew's Hospital, &c. 8vo. Stitched, pp. 59. 1832.

It is undoubtedly of great consequence to arrive at a rational, consistent, appropriate, and successful treatment of burns and scalds. That we have not yet done so must be acknowledged, and the causes of our failure are partly to be discovered in the nature of the lesion which the application of excessive heat induces, partly in the unphilosophic manner in which the injury has been viewed and treated. It may be admitted as a general principle and truth that when opinions are greatly divided on the treatment of a malady or injury, no particular plan is greatly superior to another. There is comparatively little contention on the mode of treating itch or ague, because the powers of sulphur and bark are so superior to those of other remedies as to meet with the ready assent of men of common understandings. But in more intractable complaints, where particular modes of treatment are less decidedly and widely efficacious, disputes are constantly engaged in, and parties claim the palm of superiority for the plan which they severally prefer and employ. How many specifics for tetanus have been advocated by men of talent and erudition—how opinions have been divided on the cure for hydrophobia—how many more than a thousand and one successful remedies have been proposed for the epidemic cholera in the short period which has elapsed between its rise and present fatal prevalence, we need not weary our readers by relating. The last proposition is always defended with as much eagerness and blindness as the first, and like the ephemeris it flaunts its day, and is entombed in the mists of the evening. For projectors of these schemes history contains no lessons, their own experience reveals no truths. Bigots or knaves, they continue to impose upon themselves and endeavour to impose on others, so long as the world is content to let them. Look at the present saline treatment of cholera, and pronounce whether folly could be carried further, than by those who imagine that they have discovered in tumblers of salt and water the antidote for this cruel pest. Yet so it is that confident assertions will always impose on the credulous many, and the spectators that paid their money to see a man creep into a bottle, were and will be but the representatives of the three estates of all the kingdoms of the world from Nineveh to this.

We said that the differences observed in the treatment of burns must not be considered as merely evidence of blindness on the part of practitioners, or of preference of bad to much better methods. Without doubt a good surgeon will treat a burn much better and more successfully than a bad one, and without doubt also some of the remedies which have at various times been proposed are superior to others. But to look for a panacea, to expect the discovery of a mode of treatment applicable to all cases appears to us to be a wild chimera. When the texture of the skin has been destroyed by fire, no earthly power can prevent the subsequent stages of sloughing, suppuration, granulation. To imagine that these stages can be obviated by any art, by any empiricism, is to suppose that the laws of the animal economy can be altered by medicine. We need not demonstrate that they cannot be so. It is in burns of a slighter description, where the surface of the cutis only is injured, where it is not killed throughout, that one practitioner is more successful than another, that judicious management produces a speedy cure, bad practice a protracted and injurious one. These remarks are directed against the indulgence of any notion of a specific for burns, against the foolish expectation of setting aside the ordinary operations of nature in the reparation of injuries. We do not discountenance care and attention, we do not degrade all surgeons to one level, we do not imply that one man will not follow the indications of nature whilst another will neglect them, we do not defend the shocking deformities produced, or at least permitted, by bad management, ignorance, and carelessness, but we do assert that success is owing to judgment, acquaintance with surgery in general, practical skill and assiduous attention, not to a bare adoption of particular views or employment of particular practice.

After these preliminary observations we shall put our readers in possession of the sentiments and experience of Mr. Earle upon the subject. As a hospital surgeon he must have had opportunities of seeing and treating many cases, and his opinions will therefore be entitled to the weight which such opportunities must necessarily confer. The object of Mr. Earle is to establish the treatment of burns upon "rational principles." Certainly such a substratum is very necessary for any doctrine, but unfortunately each speculator considers his principles rational, whatever they may be. Mr. Earle mentions a curious instance of the low degree of power of resisting heat possessed under particular circumstances by particular individuals.

"A lady who had been long resident in India, where she had a large family, and who had suffered considerably during her voyage home, soon after her return had the misfortune to set fire to her sleeve, which, together with part of her gown, was destroyed by the flame. She was quite dressed at the time, and her stays were only very slightly scorched on the outer side; her chemise did not appear in the least degree injured. I was called to her the day following the accident, when I found that the whole depth of skin of the entire arm, shoulder, neck, and side, down to the lower margin of the ribs, was destroyed, and had lost its vitality, the slough being deepest and longest to separate over the serratus magnus muscle, which was eventually in part exposed. Now, a very considerable part of this dead surface had not been at all exposed to the flame, the stays being only slightly scorched, but quite entire; yet the heat conveyed through them had produced such destructive effects upon the integuments. This case is certainly very remarkable; but I have no doubt that similar cases have occurred in various degrees, and I can only conjecture that the state of her nervous powers was impaired, and had in some degree lost its conservative influence."

Mr. Earle supposes that the excessive action of heat was owing in this and similar instances to some impairment of the nervous influence. It is well known that the integuments of paralysed parts are easily vesicated by the application of a degree of heat which would not produce the same effect on other parts. Mr. Earle very properly points out the necessity of attending to the *kind* of burn. In the first degree of burn the skin is reddened, but not vesicated; vesications however may subsequently occur. In the second degree, vesications, more or less extensive, are immediately induced, and in parts the cuticle is removed, leaving the surface of the cutis exposed, sometimes red and moist, sometimes dry from the influence of the atmospheric air. It is especially in these cases that good management will effect rapid cures, and bad treatment lead to the production of severe and tedious ulcerations. In the last degree of burn, the parts are killed to a greater or less depth. The surface of the skin assumes a very peculiar appearance. In parts the cuticle is peeled off, in parts adherent, seldom are there vesications over a part which is actually killed; we have however seen the skin subsequently slough under a vesication. The skin has generally a light yellowish brown appearance, it is rather mottled, in some parts dark or black, in others, where the cuticle remains, of a peculiar lack-lustre dull white. But whatever may be the colour, the sensation communicated to the fingers on touching the part is characteristic. Instead of the supple elastic integument the surgeon touches a hard, inelastic surface; the skin feels brawny, and indeed it looks so. We never have this degree of burn without the second and third degree on its outskirts. It is where the texture of the integuments or deeper parts is disorganized that their separation by sloughing must inevitably ensue.

A burn is dangerous in several ways. One of the third degree is always to be looked on with dread, if extensive. But a burn of the second degree is frequently fatal, when a great extent of surface is implicated; and it is thus that scalds destroy. Patients may die from burns of the third or the second degree without reaction—or reaction may ensue and they may die within an early period of inflammations of the serous or mucous membranes, or, as children not infrequently do, with symptoms of effusion on the brain—finally, they may sink after a still more protracted interval from the debility consequent on extensive suppuration, or from mischief lit up in the lungs or intestinal mucous membrane. We have seen death take place in all these ways, some of which are alluded to by Mr. Earle, some not, and we have no hesitation in stating that the mode of death from burns has not been thoroughly or scientifically investigated. It is quite obvious, from what has been here stated, that no one plan of treatment can possibly be applicable to burns of all degrees, and this is admitted by Mr. Earle.

We proceed to the consideration of the method which he recommends. Two opposite plans have been employed in all ages, and continue to be adopted by opposing parties in the present. One is the application of cold—the other the application of heat, or stimulants of some description. Mr. Earle says somewhat oddly, that his father was one of the *warmest* advocates for *cold* water.

"When the hot fluid has been directly applied to any exposed surface, as the hands, the speedy employment of the antidote, cold, will often prevent vesication, and the case will terminate by resolution. It more commonly, however, happens, that the part affected is at the time enveloped in some article of clothing, as the legs and feet with stockings; in which case more or less vesication is likely to occur, from the clothes retaining the hot liquid in contact with the skin. It unfortunately happens too frequently under these circumstances, that the first thing that is done is to remove the stocking or clothes, which never fails to bring away with it large portions of the cuticle, leaving the highly inflamed cutis quite denuded. If, instead of this forcible removal of the clothes, such limbs were to be immediately immersed in the coldest water, this most serious result would generally be prevented. The same clothes which were the medium for conveying and retaining the heat, may be made the readiest means of abstracting it, and diminishing the inflammation; and should it become necessary, in consequence of the formation of large vesicles, to remove them, they should be carefully cut away, and the vesicles preserved unbroken; by which the serious consequences which always follow the exposure of the highly inflamed cutis will be prevented. If this plan were more commonly inculcated, I am persuaded that much mischief would be avoided; as in a large majority of cases it happens that the bane and antidote are very near at hand, as reservoirs of cold water will generally be found in kitchens, laboratories, &c. where these accidents most frequently occur."

Mr. Earle recommends the application of cold in cases of scalds and slighter burns, especially of the extremities, and where the skin is not broken. That cold applications are better than others in these cases we are confident, but we doubt the efficacy of ordinary cold water. The plan which we always adopt, and which we have found answer best in cases of burn of the first degree, and in the first instance for burns of the second degree, is copious and continued ablution with a lotion containing spirit and superacetate of lead, followed by the application of an ointment consisting of equal parts of the unguentum plumbi and unguentum zinci. We know of no applications so soothing at first, and which so moderate inflammations afterwards as these. To the employment of cold water in cases of extensive scald there are serious objections. We saw a boy of 13 or 14 years of age, die soon after being soaked with cold water. The cutis was not in any part destroyed, and the boy died without the recurrence of reaction. It is well known also that the application of cold occasions a greater liability to inflammation of the serous and mucous membranes.

The stimulating plan certainly appears injurious in burns of the first and second degree. If the patient be excessively cold and in a state of extreme prostration it is better in such cases to use warmth than stimulants externally. Bottles of warm water should be applied to the feet and warm cordials given internally. In such cases it has seemed to us that the carded cotton or eider down is at first the best application. It serves to retain the temperature excited by the stimulants and warmth, and serves the purpose, so essential in these cases, of excluding the atmospheric air from the injured parts. If the principles we have mentioned be kept in view, the means of applying them may vary,—the liniment of lime-water and linseed oil—superacetate or carbonate of lead and oil—the absorbent powders of calamine, oxyde of zinc, or flour, &c. The latter has been much lauded, but we think it a dirty, troublesome nostrum. It forms a paste with the accumulating moisture and discharge, and is not to be tolerated when zinc or calamine can be procured.

We now come to the stimulating, or Mr. Kentish's plan of treatment. Whilst judicious surgeons must always smile at the eccentric theory on which that gentleman's practice is based, they cannot but confess that the practice itself is most valuable in the set of cases adapted for its employment. Those cases are burns of the third degree, in which the texture of the parts is more or less destroyed, and a very depressing effect produced on the nervous system. In such cases we cannot do better than bathe the parts in warm spirits of turpentine, and apply a terebinthinate ointment. Mr. Earle criticizes the subsequent stages of Mr. Kentish's treatment. Our readers are probably aware that that gentleman gradually diminishes the strength of the stimulating application, until he reaches simple dressings. Mr. K. recommends the discontinuance of stimulants as soon as the patient is free from pain, whereas Mr. Earle leaves the dressings undisturbed until suppuration commences.

"The practice which I have been long in the habit of pursuing, with very happy results, has been to bathe the parts with warm spirits of turpentine; and, as speedily as possible, to envelop every part most carefully with soft lint, thickly spread with the liniment of turpentine and resin cerate. It is better entirely to surround the extremities when burnt, and to retain the dressings with bandages, accurately but not too tightly applied. This application appears very soothing; and the young patient will often cease to cry, and will even fall asleep as soon as dressed.

Having very accurately covered every part of the burnt surface with the dressing, the patient should be suffered to remain quiet, and the dressing should not be disturbed for many days—not, indeed, until suppuration is fully established. If, on removing the dressings, deep sloughs present themselves, there is no better application than warm emollient poultices. After the separation of the sloughs, the wounds must be dressed, and treated like common ulcers; but it is not my intention, at the present moment, to enter upon the subject of the secondary treatment of burns. The same rules with respect to the mode and accuracy of dressing burns should be adhered to, whether we employ Kentish's ointment, or any other application. The principle of excluding the air and not unnecessarily disturbing the patient should be steadily kept in view, whatever may be the remedial means resorted to."

We have only one objection to make, and it is this. We have had many cases of burn under our charge, and too many have, we are sorry to say, been severe. We never found that dressings could be continued in the manner advised by Mr. Earle. Children, who form the majority of the burnt, are restless and disturb the dressings in such a manner that it is not unusual to find the trunk or the limbs extensively exposed in the course of twelve or twenty-four hours after the dressing. It is indeed a distressing thing when the whole periphery of the trunk, or even of a lower limb is burnt, for with the utmost care and dexterity on the surgeon's part, portions of the surface soon become exposed. Nevertheless we freely acknowledge that Mr. Earle is right in point of principle: and "non-intervention," as far as is compatible with the proper contact of dressings, should be our motto, before the separation of sloughs, or occurrence of suppuration in less violent cases.

We also fully coincide with Mr. Earle on the subject of internal stimulants, and strongly recommend attention to the cautions he inculcates.

"It will generally be requisite, after the receipt of any considerable burn, to administer some cordial internally combined with opiates. Whenever the pulse is small and feeble, the extremities cold, and there exists a disposition to rigors, it will be right to administer some warm brandy, or wine and water, or ammonia, with from 5 to 60 minims of laudanum, according to the age of the patient. When the vital powers are not depressed, and the patient suffers much, the opium may be given without the spirit. Considerable judgment is required in administering cordials. The great advocate for this plan, Mr. Kentish, appears to have entertained some very visionary notions, which are mixed up with his really valuable and practical observations. He advises the giving powerful stimuli internally, on the principle of counter-irritation; and he advocates the perseverance in their use for several days, until secretion has taken place. It is true that he adduced some strong facts in illustration of this plan; but I could produce equally powerful arguments to prove that such a practice is most injurious and directly opposed to the dictates of common sense, and all the principles laid down for the treatment of inflammation. To adduce one memorable instance in which this stimulating plan was most injuriously persevered in, I will mention the cases of the firemen who suffered at the burning of Covent Garden Theatre. Several of these unfortunate men were admitted into this hospital, and died with every symptom of inflammation of the membranes of the brain and mucous linings of the lungs.

The stimulating plan was carried to too great an extent in these cases, which was fully proved by examination after death. If the vital powers be greatly depressed you may administer a cordial, and even repeat it until reaction has taken place; but when once that has occurred, it can rarely, if ever, be necessary to persevere in such a plan. When the first stage is passed, light and nutritious farinaceous food should be given, and the bowels gently regulated: opiates will often be required for some time, to allay the irritation and pain. Diarrhœa sometimes supervenes, and is occasionally beneficial when the discharge is very copious. From observing the occasional good effects resulting from spontaneous diarrhœa, when the discharge was profuse, in accelerating the healing process, Mr. Kentish strongly recommends the free use of purgatives under such circumstances, and I have known them very useful. The spontaneous diarrhœa, however, requires to be very carefully watched, as it may arise from a destructive inflammation of the mucous membrane of the bowels, which may require all our efforts to control."

We repeat, that the constitutional consequences of burns have not hitherto been investigated scientifically, although the inquiry is one of physiological as well as practical interest.

"In the second order of burns and scalds, when there is more or less of the cuticle destroyed, and the highly inflamed cutis exposed, it is right as soon as possible to exclude the air; and for this purpose, I believe you will find the lime-water and linseed oil a very efficacious remedy. When the vesications are very large, and cause pain from their distention, and when there is danger of their bursting from their bulk or situation, it is advisable to puncture them with a needle at several points, in the direction of the scales of the cuticle; this will allow of the escape of the serum without admitting the air to the cutis. But in all minor cases it is not necessary to do this, and whenever you can with safety leave the bladders untouched for one or two days, it is far better than puncturing them. By carefully pursuing this plan you will in most cases avoid that serious ulceration and sloughing, which so frequently follows the exposure of the inflamed cutis. The fluid will often be entirely absorbed, and the cuticle peel off, leaving the surface quite healed beneath it."

All this is true so far as it goes, but that is not far enough. When the cutis is exposed, cuticle may either be formed by a rapid process, or by a painful and tedious one of suppuration, perhaps ulceration. The difference of result depends in part on the actual injury which the excess of heat has inflicted on the structure of the cutis, in part on the length of time during which the latter has been exposed, and in a great degree on the surgeon himself. If he trusts to nature he will have a troublesome job, if he continues greasy applications he will be no better off, if he uses merely absorbent powders he will fail in many cases and be successful in a few, but if he does what we are about to mention he will succeed in many cases and fail but seldom. After adopting the plan which we have alluded to as applying to burns of the first and second degree, he must carefully watch when the surface of the cutis is beginning to secrete pus. It will then assume a peculiarly florid appearance, and the redder papillæ will give to it much of the aspect of a strawberry. Let him seize this moment of *washing* the surface with a solution of sulphate of zinc, or Bates's Red Wash, or of nitrate of silver, in the proportion of four grains or upwards to the ounce of water, according to the active or indolent appearance of the cutis. Having done this allow the surface to dry, or gently dry it with soft lint. Then sprinkle on the surface a mixture of calamine and oxyde of zinc powder, and treat it subsequently by these powders in the manner in which they are usually employed. It may be necessary to repeat the wash partially or generally, but the success of this method when judiciously used is certainly very considerable. All local treatment of superficial burns should be conjoined with appropriate constitutional treatment. Saline medicines and purging when there is excitement, and tonics when that excitement has passed away, will suggest themselves to all reflecting practitioners.

This concludes our notice of Mr. Earle's lecture on the primary treatment of burns and scalds. We think it judicious and calculated to do good. But we cannot in sincerity assert that it tells us any thing of which we were previously ignorant, or that it evinces the possession of principles or employment of practice superior to those of well-informed men. We are quite sure that if any person will step into one of our large hospitals, *not* St. Bartholomew's, he will see burns treated with views as sound, and with means equally well regulated and perhaps more varied, than what appear in our author's pages. But information is too frequently possessed in particular quarters which is not universally disseminated. This is the case with the treatment of burns. We have too often seen reason to deplore injudicious and mischievous applications employed, and young men appear to think that the management of these lesions is enveloped in a veil of mystery, impenetrable to the ordinary operations of sense. Such practical statements as those of Mr. Earle are calculated to disabuse them of this dangerous and vague illusion, and we seize all convenient opportunities of directing public professional attention to the subject.

### SECONDARY CONSEQUENCES OF BURNS.

Mr. Earle makes several good remarks on the management of ulcers after burns, but we have not space for their insertion, and indeed they are only calculated for young men who are not yet hospital dressers or house surgeons, or for older ignorami, who are no better for having been so. Mr. Earle directs the attention of students to the manner in which contractions at the flexures of joints, in the neck, &c. occur after burns, points out their distressing consequences, and the power of proper care, position, and permanent extension during the process of cure, and for some time afterwards, in preventing such unseemly and, to the surgeon, disgraceful occurrences. It is curious that, after the cicatrization of a burn, there is a great disposition in the cutis to become welted and thickened. Continual traction and irritation are, doubtless, the ordinary causes of this, but Mr. Earle remarks that they are not always so.

" Still, however, it must be admitted that, in some parts of the trunk, where no such contraction can operate, the cicatrices after burns form prominent ridges, and are morbidly hard; probably in consequence of extensive destruction of the subcutaneous cellular tissue.* This will at times amount to an increased morbid growth to a considerable extent, which will have, when cut into, all the characters of a true scirrhus. Such a case occurred to me, some years since, in the person of the young female, of whose neck I now present you a drawing, exhibiting prominent pendulous tumours, one of which I removed, and approximated the healthy integuments, in hopes of obtaining union by first intention. Erysipelas supervened, and the surface granulated; and after it had healed, the same diseased growth returned nearly to the same extent. This is the only instance of the kind which I have met with, and it appeared to depend on some peculiarity of the individual, as the whole original cicatrix was freely removed. When such a horny web or cicatrix, as I have described, has contracted any joint, or the neck, it would appear, to a superficial observer, that the whole evil depended on the contracted integuments, by a simple division of which the limb would be instantly set at liberty. So deceptive is this appearance, that I have known, more than once, that surgeons have indulged this vain hope of affording relief, until a painful and ineffectual operation has convinced them of their error. In recent cases, occurring in any of the extremities, the contraction may be confined to the integuments, by dividing which the deformity may for a time be removed; but the same cause continuing to operate, will produce the same effect, and the cicatrix will again contract after the wound is suffered to heal up. When the contraction has been of longer duration the muscles acquire a new sphere of action, and afford an additional and powerful opposition to the free exercise of the limb. Lastly, in some cases we find that even the bony fabric becomes moulded and altered by the powerful constriction exerted on it by this gradual but certain process. In such cases it is hardly necessary to add, that the most severe operations cannot afford a prospect of even temporary alleviation."

Reflecting on these circumstances, it is well known that Mr. Earle, in the year 1813, excised a cicatrix, and brought the integuments together, in a direction at right angles with that in which the contraction had taken place. He has operated on more than twenty cases with success, and has not observed that subsequent return of contraction which is said to have occurred in the practice of some other surgeons. Of course great attention must be paid to the maintenance of a proper position after the operation.

" Mr. James constructed a very ingenious apparatus for maintaining the chin elevated after operations for relieving contractions of the neck. This plan, with certain modifications, I have found most beneficial not only in curing, but also in preventing such contractions. In slighter cases the wearing, night and day, a stiff soldier's collar will be sufficient protection against contraction, provided the integument

* " The speculations of Hildanus as to the causes of this induration may be amusing to some of my readers. ' Præcipuæ causæ turpitudinis cicatricum post combustionum curationes sunt, primo, quod cutis, caro, venæ, &c. vi ignis, contrahuntur et indurantur; secundo, quod humidum radicale (cujus beneficio vulnera et ulcera omnia cicatrizantur) vi ignis exiccatum consumptum est, inde fit ut quemadmodum siccus et aridus ager, spinas tortuosas zizania et omnia imperfecta, ager vero pinguis et humidus omnia perfecta producit: ita etiam ex caloris innati et humidi radicalis defectu cicatrices fiunt turpissimæ.'—*De Combust.* cap. 14.

immediately below the chin be not burnt. It has been asked, At how distant a period from the receipt of the injury success may be obtained? This must, of course, depend upon the situation of the burn, and extent to which the muscles and bones are implicated in the mischief. I have, however, been truly astonished to find at how remote a period contractions of the fingers, hands, and arms may be restored. A young lady, aged 17, applied to me for an affection of her spine: during my attendance, I observed that her right hand was much deformed, in consequence of very firm cicatrices which bound down her thumb and three fingers, so as to greatly interfere with the use of the hand. She had been burnt when quite an infant, and the defect had existed for nearly 16 years, growing with her growth. From this history it was to be apprehended that the tendons and muscles would afford a serious obstacle to any improvement. I proposed, however, to make a trial with only one finger, and explained my views to the lady and her friends. She cheerfully acquiesced, as she was very desirous of being able to reach an octave on the piano-forte, being very fond of music. I succeeded so perfectly with the first finger that I proceeded with the others, and the thumb; and, eventually, restored the hand, and enabled my patient to attain the object of her ambition.

In performing operations for the removal of contractions, it will generally be better to excise the indurated cicatrix, although in some cases it may be divided on both sides, and dissected upwards or downwards, leaving it attached at one extremity; some portion will thus be retained, and the extent of wound will be less. In several cases, however, where this has been attempted, the detached cicatrix has sloughed, and no time has been gained. As the cicatrix is not a part of original formation it possesses less vitality, and is often very imperfectly nourished; when, therefore, only partially detached, it will generally perish. It often happens, that but little apparent good is effected at the time of the operation; but, by gradually extending the limb, in a few days the muscles and soft parts yield, and the contraction will be gradually removed. This occurs in a marked degree in contractions at the elbow, and of the wrist and fingers. Too forcible an extension should not be attempted at first; but, with the assistance of graduated splints, a little may be gained from day to day, until the limb is perfectly restored."

Mr. Hodgson has succeeded, it would appear, in removing contractions by mechanical contrivances, independent of any surgical operation. Mr. E. has twice adopted this practice. One patient, a girl, was affected with a contraction in front of the neck, and was greatly benefited by wearing a firm collar for a considerable time. The other patient had been burnt with lightning, and had the knee-joint contracted nearly to a right angle; the leg has been extended by means of a powerful double screw, but the whole cicatrix has ulcerated, and the tendon of the biceps can now be seen. The wound is as large as would have been made by the operation, and more time has been lost. When Mr. Earle published the first case in which he performed the operation, he was not aware that he had been anticipated by any other surgeon. He has since found that Hildanus has both recommended such an operation, and, to some degree, practised it; so true it is that there is nothing new, and so obvious it ought to be, that men should rather aim at being distinguished by sound judgment and reflection, than by a prurient desire of reputation for discoveries, which have probably been discovered and re-discovered a dozen times since the origin of civilization and learning.

Appended to the pamphlet are two lithographic plates, representing deformities after burns, and some instruments made by Mr. Ferguson, of Smithfield, for their prevention or removal. Before parting with the able and indefatigable author we beg to assure him that his pamphlet is calculated to be productive of benefit, by promoting the dissemination of good principles of treatment and eradicating bad.

---

### XXXIV.

Tabulæ Anatomiam Comparativam Illustrantes, quas exhibuit Carolus G. Carus, Med. et Philos. Doctor, &c. Pars III. pp. 22, folio. Lipsiæ, J. A. Barth, 1831.

Professor Carus, in a series of nine lithographic plates, has very beautifully illustrated a branch of physiology, which is alike most difficult and interestingly important; viz. the history of the evolution of the embryo and fœtus in the various natural classes of the animal world.

The subjects which he has selected for this purpose are, in general, well adapted to convey clear and intelligible ideas of the different modifications of the generative process; and in order to give as comprehensive a detail of particulars as is necessary for this elaborate theme, he has embodied numerous observations of other authors with his own original researches. Like most of his countrymen, he employs the synthetic method of description; not proceeding from things which are most, to those which are least known, and illustrating the process of generation from the higher to the lower animals, but rather addressing himself to the more advanced student of Nature, who already is acquainted with the varied divisions of zoological arrangement, and proceeding from the humble polypi to the organization of the mammalia.

The first plate is illustrative of the mode of propagation in that tribe of zoophytes which, on account of the less perfect substance proper to their different organs, and from their character, as representing the elementary stamina of the animal kingdom, he proposes to call "oozoa." The genera figured are "lacinularia," "plumatella," and "hydra," all appertaining to the tribe of naked fresh-water polypes. A new species of the first genus found by Professor Carus in the Elbe, and which he calls "L. fluviatilis," forms the subject of the first nine figures. These little animals are grouped together to the number of about 20, and are attached by a common stem to the surface of webs; in each of these, a single ovum appears to be developed in the parenchyma of the body by the side of the intestinal canal; the ova drop out among the parent stems; in time they grow, and become similarly affixed, thus supplying the place of the polypes of a previous generation.

Figures from 10 to 17 exhibit the evolution of the germs of plumatella; according to the author, this is effected in three different ways—first, by germination; secondly, by the formation of ova, which possess a firm corneous covering of a brown colour; and, lastly, by ova of a different kind, translucid, and whose whole circumference is beset with delicate oscillatory cilia. We, however, much doubt whether these remarkable differences in the ova do not rather indicate something more than a mere difference of species; we may also observe, that the last-mentioned is by far the most common structure of the ova of zoophytes. Mr. Ellis, Professor Grant, and others, have particularly described it, as it is exemplified in different genera; and it is by means of the peripheral cilia that the ova of the most fixed and motionless zoophytes, such as the sponge, execute, for a time, the locomotive faculty, and are carried to a greater or less distance from the parent stock, which otherwise might be injured by the accumulation of the ova around it, and be thus prematurely destroyed. Professor Carus ascribes another function to these oscillatory cilia, and supposes them to be somehow connected with respiration.

It is well known that the propagation of some zoophytes, such as the hydra viridis of Trembley, is effected by germination, a process which our author considers to be not essentially different from the formation of ova; for a germ or bud is equivalent to an egg, and *vice versâ*.

The second plate illustrates the evolution of the fœtus in several of the mollusca, such as the anodonta (a bivalva), the aplysia, lymnæus or fresh-water snail, the sepia or cuttle fish, and the argonanta, or paper nautilus. In the molluscs of the bivalve shells, the ova are found first in the substance of the foot, behind the liver, and around the alimentary canal, and afterwards pass into compartments in the laminæ

of the external gills;—in the space of eight days, the rudiments of the valves are perceptible, and the globule of Vitellus begins, as in the ova of the gasteropeda, to revolve regularly upon its axis; a motion which Carus attributes to the vortex, or eddy produced by the respiratory organs.

Among the figures which exhibit the generative process in the Gasteropodous Mollusk, as the aplysia and lymnæa, is a scheme of the singular revolving motions of their ova, which Professor Carus has observed to be more rapid in this, than in the preceding order (acephala.)

The remaining figures in this plate very satisfactorily shew the stages of evolution among some of the Cephalopodous Mollusca, viz. the Sepia Officinalis, and Loligo Sepiola; and to these is added the more than suspected figure of the embryo of the Ocythoë, or animal which inhabits the argonata argo, with the shell, published by Della Chiaja, in the continuation of the great work of Poli.

Table III. contains delineations of the various stages of the embryo in different tribes of the articulate series of animals, as of the intestinal worm (Spiroftera) from Nitzsch; of the medicinal leech, from Weber; of the spider, mole-cricket, and pine-moth.

The author considers certain motions which may be observed in the ova of the leech as being analogous to the above-described motions in the ova of the preceding classes, and as being essentially dependent on the respiratory actions. In the higher articulate animals a fatty substance (guttulum oleosum) is first to be perceived as a component part of the ovum, a part not found in the embryos of the mollusca, but which occurs in one form or another in those of all the higher classes.

Tables IV. and V. contain numerous figures of great interest, exhibiting the several stages of development observed by the author himself in a species of cyprinus, (c. dobulus, or dobel, a fish which is allied to the common tench, and carp.)

The details however are so minute, and elaborate, that we can afford only a very passing notice, and strongly recommend our readers to a studious perusal of the particulars.

The oily globule is observed here, as in the articulata, to be attached to the vitellus. On the 4th day after the detachment of the ovum, the rudiments of the embryo are visible; on the 6th its form can be plainly seen. The development of the intestinal canal from the membrane of the vitelline globule is illustrated by diagrams.

On the 7th day, the bodies of the vertebræ, the heart and brain simultaneously appear. On the 8th the lineaments of the organs of sense are manifest. On the 9th the natural divisions of the heart are seen; numerous branches from the vena cava constitute the rudiments of the liver; and thus prove the consentaneous origin of arteries and veins. On the 12th the bronchiæ are observed, and the arteries going to supply them. Six days after having quitted the ovum, the fœtus manifests the truly developed liver. The sacculus vitelli has passed entirely into the abdomen, and the swimming bladder is apparent near the spine.

Table VI. illustrates the generation of the salmon, shark, and pipe fish.

Table VII. shews the surprisingly beautiful metamorphoses of the tadpole to the perfect frog; and the evolution of the young of the salamander, of several serpents, lizards, and tortoises.

In Table VIII. we have numerous figures of the evolution of the chick, in our common fowl; and as it is expected that every medical man, however little taste he may have for comparative anatomy and natural history, should be acquainted with this part of the subject, we propose to revert to it in our next number, when we shall describe in detail the different figures of the eighth plate, and also the particulars of the ninth plate, which illustrates the process of generation in some of the mammalia.

In concluding the notice of this work by Carus we have the authority of a distinguished comparative anatomist, in designating it as one of high utility; and as a most correct compendium of what is known on the very important and mysterious subject of physiological research, that of generation. To those who are interested in the knowledge of the development of the fœtus, and we hope that every enlightened member of the medical profession is so, we strongly recommend an attentive examination of the plates.

---

XXXV.

### Mr. Arnott and the Medico-Chirurgical Reviewer.

In the last number of this Journal, p. 221, et seq., some remarks were made upon a pamphlet on Diseases of the Vascular System, published by Dr. A. T. Wise, in Bengal, and transmitted to the Editor of this Journal. Dr. Wise accused Mr. Arnott of plagiarizing his ideas and publishing his cases. In the number of the Medical Gazette which immediately followed the publication of the Journal containing the article in question, there appeared a letter from Mr. Arnott directed to Dr. James Johnson, and accusing him, as Editor of the Medico-Chirurgical Review, or writer of the article, of assurance and partiality. The gentleman who actually wrote the critique complained of, forwarded an answer to Mr. Arnott's letter to the Editor of the Medical Gazette. That Editor would not insert it. As it is consonant with the principles of justice and common sense, that Mr. Arnott should not be suffered to make accusations without an opportunity of defence being afforded to the accused, the letter in question is inserted here.

It is not required by law, nor justice, nor reason, that the Editor of a periodical should affix his name to it. The Edinburgh and Quarterly Review, have no avowed nor responsible editors. Neither is it required that the name of the writer should be appended to an article in a periodical, whether the Editor be avowed or not. Such is the custom and it is a good one. But if an article give offence, and the author criticised or person remarked on reply to that article, it may appear to some that the writer should then declare himself. Whether he should or should not, appears to us to depend on circumstances. If the writer of the article has stated on his personal authority facts which are denied by the accused, then, as the dispute is one which must be decided in a great degree by the weight of character, we might in justice call on the accuser to confront the accused. But when the dispute is on the nature of the article itself, on the arguments it contains and the expressions adopted in it, there is no such necessity for confronting the parties; the matter is to be decided on its own merits, and may be shewn to be false or true, absurd or reasonable, without any reference to the man who penned it.

Such principles are defensible by argument, and, what is better, they are founded on propriety and justice. They have always been acted on by the conductors of this Journal, and whatever may be the practice with other periodicals, they will always be the pole-star of this. We therefore, as we said before, insert the reply to Mr. Arnott, as it was transmitted to us by the Reviewer, because, if a gentleman is attacked for what he writes in a journal, he may expect to be allowed to defend himself in its pages. The conductors of this Journal entertain no ill-will towards Mr. Arnott, though they think he might have followed a better course than he has done. However, it matters little to them how violent personal attacks may be, when reason and justice are with them, for against such defences personalities are harmless, or recoil on him who hurls them. In order that the whole question may be laid before our readers, and that Mr. Arnott may not complain of partiality we insert his letter as well as the Reviewer's. Having done this we wash our hands of the controversy.

"*To the Editor of the London Medical Gazette.*

Mr. Arnott solicits the Editor of the Medical Gazette to do him the favour of giving insertion to the enclosed letter. When the conductor of a Quarterly Journal descends to personal attacks, either to gratify his own feelings or those of others, the weekly medical press is naturally looked to for aid in promptly repelling those calumnious charges.

"*To Dr. James Johnson, Editor of the Medico-Chirurgical Review.*

SIR,

An article in your journal for the present month has been pointed out to my notice, in which, assuming the representations of a writer in India to be correct, you think fit to accuse me of plagiarism, and of purloining the property of another. To your part in this transaction I shall advert subsequently; I first reply to the statements upon which you presume to make these charges.

From your account, it would appear that a Dr. Wise has recently published, at Calcutta, a pamphlet upon the Diseases of the Vascular System; and that, not in the printed essay, but in a letter accompanying a copy of it, to you or some one else, he takes the liberty of asserting that to him, while house-surgeon at St. Bartholomew's, I am indebted for my attention being first drawn to the subject of phlebitis—that I published as my own 'opinions,' 'cases' to which he first directed my attention—and that the three first cases which I have related were those alluded to. Without commenting on the length of time which has elapsed between the publication of my paper on the Secondary Effects of Inflammation of the Veins, and the advancement of these charges, I at once answer that they are unfounded. My attention was *not* called to the subject of phlebitis by Dr. Wise; I did *not* derive any cases, materials, facts, or opinions from him; nor do I know what were, or may be, his opinions thereon.

I was in the habit of attending Mr. Lawrence's visit at the hospital before I ever saw or heard of Dr. Wise. The three first cases published in my paper, which that gentleman is pleased to insinuate that I stole from him, occurred in the wards of that hospital, under the care of Mr. Lawrence. *The dissections were made by Mr. Lawrence; and from Mr. Lawrence's notes, which he had the kindness to lend me, my account was derived.* I was in attendance at the hospital when these cases happened; I witnessed their daily progress, and it was the extraordinary circumstances accompanying them which arrested my attention; *not the suggestion of any one.* I never saw a dissection by Dr. Wise of a patient who died from phlebitis, or any drawing of his on that subject. I am not aware of what is meant by 'the result of Dr. Wise's labours on the pathology of the vascular system:' the only opinions of his that came to my knowledge were some speculations analogous to those already promulgated by several French writers, upon a supposed affection of the veins in typhoid fevers and erysipelas, and certain changes which had been observed in the blood of the veins in persons who had died of malignant disease.

Thus much for the charges; and now, sir, let me acknowledge, as I ought, the sense I entertain of the manner in which you have brought them forward. You set out by proclaiming that you are going to do 'an act of justice;' you expatiate on the cruelty of a man's having any credit or emolument wrested from him by the grasp of another; you mention my name in connexion with plagiarism, and with purloining the property of another; and, after all this, you have the assurance or simplicity to pretend that you are perfectly impartial—that you espouse neither side, and are no party in a quarrel of which I now hear for the first time. I will answer for it that there is not another editor of a medical journal who would have published portions of a letter, containing such allegations against any individual, without making some inquiries as to their veracity—more especially if conscious that three months must intervene before any answer thereto could appear in his pages. For you, sir, there is no excuse in giving currency to acccusations, the truth or falsehood of which you had the means of ascertaining. You noticed my paper at some length, and therefore ought to have known that the three cases which I am accused of having assumed as my own, I did *not* cite as such, but as belonging to the distinguished surgeon in whose practice they occurred, and to whom, in relating them, I acknowledged my obligation.

I am, sir,

Your obedient servant,

JAMES M. ARNOTT.

2, *New Burlington-st.*

*July* 5, 1832."

*To the Editor of the Medical Gazette.*

Sir,—In the Number of the Medical Gazette published on Saturday, July 13th, I observe a communication from Mr. Arnott, complaining, in no measured terms, of an article which appeared in the Medico-Chirurgical Review for the present month. As the writer of that article I shall reply to Mr. Arnott, and although insinuations which might well have been spared are directed by that gentleman against Dr. James Johnson, I trust that I shall display more command of temper, and adopt a more gentlemanly tone than Mr. Arnott has done. Dr. Johnson was not the author of the article in question, nor was he aware of its existence till after its publication. I agree with those who consider the editor of a periodical work responsible, both legally and morally, for what may appear upon its pages. But I protest against the practice of attributing to an editor every sentiment as his own, and of impugning his motives on every occasion, when an author or gentleman feels himself aggrieved by a critique or a remark. It is a species of personality, masked it may be, but not the less reprehensible.

But Mr. Arnott's personality is not masked; it is open, undisguised, and gross. He accuses Dr. James Johnson of "the assurance or simplicity to pretend that he is perfectly impartial," language which I shall not stoop to denounce—a mode of argument to which Dr. Johnson would not condescend to reply. And this abuse is heaped upon Dr. Johnson, because Mr. Arnott has chosen to assume that Dr. Johnson wrote an article which, in point of fact, he scarcely read, until this violent attack must have called his attention to it.

I will now, Sir, proceed to examine the grounds of Mr. Arnott's indignation, and to defend, if defence is possible, the article which has stung him into such extravagant anger. Mr. Arnott's charges appear to be threefold. First, that Dr. Johnson, as Editor of the Medico-Chirurgical Review, has published a letter reflecting on Mr. Arnott, without previously consulting him, Mr. Arnott; or, to use his own peculiarly gentlemanly phraseology, "he will answer for it that there is not another editor of a medical journal, who would have published portions of a letter, containing *such* allegations against any individual, without making some inquiries as to their veracity." Mr. A. is Quixotic in answering thus for editors in the lump; he may have enough to do to answer, on all occasions, for himself. Secondly, he protests against expressions and sentiments employed by the reviewer, and attributed by him to the editor. "When the conductor of a Quarterly Journal (says he) descends to *personal attacks*, either to *gratify his own feelings or those of others*, the weekly medical press is *naturally* looked to for aid in promptly repelling those calumnious charges." Mr. Arnott would appear to consider the maxim, divide et impera, a good one, for he thinks it *natural* that the weekly press should be used as a means of defence or offence against the quarterly; I have just shewn that he will answer for all editors, including, I suppose, the monthly, against the unfortunate one selected by him for such signal chastisement. Thirdly, Mr. Arnott replies to the accusations of Dr. Wise.

I have already stated, and I repeat it, that Dr. Johnson was not the writer of the obnoxious article, nor was he concerned with it, directly or indirectly. The first and second counts of the indictment, then, are levelled against me, the third against Dr. Wise. I do not intend to act as umpire between these gentlemen; the quarrel is theirs—the stake, reputation—the natural judges are the public. But before that public I am now on my own defence, as a writer in a periodical, against the charges of Mr. Arnott, and I hope to shew that they are both ungenerous and unjust.

And first of the imputation, that "a letter was published containing *such* allegations, without inquiries being made as to their veracity." Mr. Arnott's ideas of the duties of editors must be a strange one, indeed, from the ready manner in which he *answers* for them, he seems to consider them an extraordinary race. If an editor should publish an anonymous slander (and such things have happened, incredulous as Mr. Arnott may be,) we would willingly join Mr. Arnott, or any honourable man, in denouncing the practice as treacherous and base. But when a statement is, to a certain extent, authenticated by the publicly-appended signature of a respectable man, it is too much to expect that any editor or writer should proceed to sift the evidence on both sides, and bandy letters from disputant to disputant, before he ventured to publish what he had received. If an accusation is openly preferred by one gentleman in the profession against another, I conceive that an editor is not only justified in inserting it, but that dishonour and disgrace should attach to him if he refused. And mark the parties in the present strife. One in a distant land, who can seldom have communication with Great Britain; the other a hospital surgeon in this metropolis, under the wing, I may say of his *natural* protectors, the weekly press. On which side the balance of interest is—on which side the preponderance of power lies, I need hardly stop to tell; but I know it must be evident that, in publishing the complaint of the surgeon in Bengal against the surgeon of the Middlesex Hospital, the editor of the quarterly review was exposing himself to the vituperation of the strongest.

The second charge advanced by Mr. Arnott applies to the manner in which the statements of Dr. Wise were introduced—to the expressions employed by the reviewer. I wish, Sir, I could intrude the whole of those expressions on your pages, that your readers, in fact, might judge of the caustic quality, not of sarcasms, but of compliments. I say, of compliments, for such they are, and Mr. Arnott must be thin-skinned, indeed, to feel the prick of the rose so sensitively as he has done. I appeal to all persons, unprejudiced by an excess of sensibility, to read those comments *throughout*, and say that they breathe aught but a tone of respect towards Mr. Arnott, tempered by a feeling of regret that he should fall under a public *accusation* of plagiarism. In the accusation itself I was not concerned, nor am I now; I stated, and I state it again, "we are no parties in the quarrel—we espouse neither side: but as this is a heavy accusation, it is imperative on Mr. Arnott to meet it and repel it."* I added, what think you, Sir! the sting of the libel! it was this. "In order that he (Mr. Arnott) may see with what he is charged, we shall insert the statement of Dr. Wise *as it is transmitted to us*, and we entertain no doubt, from the *high character which Mr. Arnott bears*, that he can satisfactorily remove the impression which such a statement, uncontradicted, would be calculated to produce."† Such are the calumnies of which Mr. Arnott complains, such his reasons for accusing Dr. Johnson of assurance, simplicity, and descending to personal attacks to gratify his own feelings. For shame, Mr. Arnott, for shame.

I have done all I proposed to do, defended myself, as reviewer, from Mr. Arnott's charges against me. I shall not examine his answer to Dr. Wise, for the latter gentleman will, in all probability, see that answer, and must vindicate himself, if vindication should seem to be necessary. Before I part from Mr. Arnott, let me give him a word of advice; less testiness will expose him, hereafter, to fewer mortifications, and he is much deceived if he thinks that noisy vapouring will either intimidate or convince. Any future notice of this affair from me will depend solely upon Mr. Arnott; but, however unwilling I may be to contend with one for whom I formerly entertained, and for whom I hope to continue to entertain a high respect, I owe it to myself, and to the Journal to which I have been a humble contributor, to repel argument by argument, and, disagreeable as it may be, to treat personalities as they deserve. I would, on all accounts, shun a contest with Mr. Arnott; but if he is determined to engage in one, I have

* Med.-Chir. Review, No. XXXIII. p. 221.

† Ibid.

sufficient firmness not to be daunted by his real merits or his idle wrath.

THE REVIEWER IN THE MEDICO-CHIRURGICAL REVIEW.

*July* 16*th*, 1832

---

XXXVI.

MR. CRAMPTON ON INJURIES OF THE HEAD.*

OUR contemporary of the silent city, though silence is certainly not the present attribute of the Green Isle, is likely to prove a valuable addition to periodical medical literature. We cannot but suspect that something like a feeling of shame exists among the professional magnates of Ireland, at the imputation of sloth which has not been unfrequently pointed against them. The heaving and stirring of their brethren in London, and the steady activity of Scotland, have stimulated them at intervals into efforts of exertion and deeds of emprise. But these have been far from constant, and though the flame would flare brightly for a season, it sank anon into its socket. We have had some admirable papers from the Dublin surgeons and physicians—they have given us a volume or two of excellent memoirs—but the genius of Ireland speedily waved her emerald wand, and the restless spirits sank exhausted and contented with their transient energy.

Even now they have been unable to produce a purely medical journal, and surgery has been fused in the alembic of chemistry, before it was found sufficiently malleable to receive the impress of the Dublin doctors. But we do not despair. In the present age of reform and revolution, when ancient structures are discovered to be crazy, and the temple of Dulness offers an insecure sanctuary to her votaries, men who have been placed by fortune or by merit in high situations, begin to feel that they must either use their hands or tumble. This truth having become manifest in most civilized countries, we need hardly feel surprized that Ireland should begin to display symptoms of its operation.

Wide and more wide it spread o'er all the realm !

Seriously, we cannot but express our approbation of this effort on the part of our Irish brothers. Those who are acquainted with the state of medicine and surgery in Dublin entertain too favourable an opinion of it, not to wish that its treasures should be rendered more available and useful to the world. High as Edinburgh stands in the opinions of Europe, we sincerely think that nothing is wanting on the part of Dublin but exertion and perseverance, to enable her to challenge the world. Returning to the consideration of the Dublin Journal, we must again express our approbation of the manner in which it is conducted, and our belief that, if continued with the same amount of zeal, and an increase of experience, it will be the means of advancing the best interests of the profession, and enhancing the reputation of Irish medicine. The paper to which we would call particular attention, is one from Mr. Crampton. The sentiments which it contains, and which we shall certainly transcribe, are honourable to his feelings, and creditable to his intelligence.

"It has often been objected to medical men, who are extensively engaged in practice, that they seldom communicate the result of their experience in such a way as to make it as available as it might be to the junior part of the profession. That they occasionally put forth large and valuable works on particular departments of the healing art, and communicate through the periodical press, the particulars of some remarkable case or brilliant operation, is not to be denied ; but this is not precisely the kind of information, or I should rather say, it is not the *only* information which the younger part of the profession are most anxious to obtain from them. They would, above all things, desire to learn in the shortest, simplest, and least expensive way, how a professional man of experience would manage

* Dublin Journal, No. IV.

certain cases which are of almost daily occurrence, but with respect to which the greatest diversity of opinion and practice prevails among the highest authorities.

They read, for example, that in France amputation is performed while the gangrene (from external injury) is rapidly extending up the limb; in Great Britain, on the other hand, this practice is reprobated by the *generality* of teachers and of systematic writers.* Again, amputation of the thigh is performed in one manner in London, in another in Edinburgh, and in a third in Dublin; the same may be said of lithotomy. In England I believe it is the general practice to open large chronic abscesses by a small valvular puncture, and to draw off the fluid in portions from time to time. In Paris and in Berlin, on the contrary, the abscess is opened by an extensive incision, so as to give issue to the whole of the contained matter at once. Again, in the treatment of compound fracture of the skull, the trephine is much more generally employed in London than in Dublin. But without multiplying examples, I am warranted in stating broadly, that the modes of practice in London, Dublin, Edinburgh, Paris, and Berlin, are on some of the most important points of surgery, not only different from, but often absolutely opposite to each other. How useful then must it be to the practitioner to be enabled to examine and compare the facts, and the reasonings on which this diversity of opinion and practice is founded, and how noble a task for an enlightened criticism, uninfluenced by prejudices either national or personal, to enable the enquirer after truth, to view those various modes of practice by the clear light of science and experience. It may at the first view seem strange, that at a time when a greater intercourse exists between the different capitals of Europe, than a few years since existed between different parts of the same kingdom, and when by means of a weekly periodical press, the opinions and practice of one country are scarcely announced, when they are known in every other; that there should be not merely a national, but almost a provincial surgery. It would be foreign from my present purpose to investigate the causes of this diversity, although the inquiry might not be devoid of interest or even of utility, but I may observe in passing, that the causes lie deep among the most immutable principles of human nature. Uniformity of practice in an art which mainly rests on human opinion is plainly impossible, and if possible would not be desirable, unless the art were brought to the utmost degree of perfection of which it is susceptible; but as this can never happen, so long as the human mind is progressive in improvement, all that can be done for the advancement of knowledge, is to engage the greatest possible number of intelligent, active, and independent spirits in the pursuit, and to bring the result of their separate labours into juxta-position, in order that they may be examined and compared, and their relative merits experimentally ascertained. In this way there will be a gradual and therefore safe approximation to uniformity of practice, and a pro tanto advance towards the perfection of the art. That the publication of clinical lectures, and of (what is perhaps of still more value) the incidental clinical remarks of the hospital surgeon while 'going his rounds,' has done much towards the advancement of surgical knowledge, is not to be denied; but until the practice becomes more general than it is at present, I believe that hospital-surgeons cannot perform a better or more acceptable service to the profession and to the public than by becoming their own 'reporters,' and communicating from time to time, through the medium of the periodical press, the results of their experience, without waiting until they have time to give them the form of a a regular treatise, or what is still more

* "It would appear, however, that opinion on this point has undergone a considerable alteration within the last few years in England.—*See* SIR A. COOPER'S *Lectures*—GUTHRIE *on Gun-shot Wounds*, and HENNEN'S *Military Surgery*."

deplorable, to expand them into a book; and surely if the practice of adulterating valuable medicines by the addition of some noxious, or, at best, useless ingredient be a punishable offence, those authors should not escape with impunity, who overlay some useful practical observation with the rubbish of a library. It may be objected to the plan of self-reporting, that it affords too wide a field for the exercise of vanity or of self-interest, and that even if the public had a sufficient guarantee for the integrity of the reporter, the influence of previous opinion, and a variety of other motives might induce him to view *his own case* in a very different light from that in which it might appear to a mere looker on; and this to a certain extent is true; but it is equally true, that a perfect corrective is at hand, and should always be applied. Let no case be reported on the authority of any indivividual, however trustworthy. Let every possible security be given against error, as well as fraud; if the case occur in hospital practice, misrepresentation will scarcely be ventured upon, and error will easily be detected and exposed. If in private practice, let some medical practitioner, who is acquainted with all the circumstances of the case, be referred to by name; as a further security, the name and residence of the patients (whenever it can be done so with propriety) should be inserted in the case; for want of this attention, surgical cases in particular, (however faithfully recorded,) are deprived of the greater part of their value; for it is not enough that an operation should be well and successfully performed, the great point to be ascertained is, how far it has been *permanently useful*, and this may be known by a re-examination of the patient after the lapse of a considerable time. We have read of the cutting out, '*with success*,' of large portions of the ribs, when affected by cancer, and the cutting off one-half of the face, when affected by the same disease; of removing a tumour of such dimensions, that it was difficult to say whether the tumour was cut away from the man, or the man from the tumour. But how far the subjects of these proceedings were compensated for the immediate pain and danger attendant on the operation, by a prolongation of life, or an abatement of suffering, can be learned only by following them from the hospital to their homes, if, indeed, they ever reach them after such 'successful operations.'

A Periodical Medical Journal, then, if conducted on the plan to which I have alluded, would faithfully reflect the state of medical opinion and practice in the country in which it was published, it would be, in a manner, the auto-biography of the healing art, the only kind of biography which, perhaps, has a claim to implicit credit; for it has been well remarked by a great writer, himself the best of biographers, that whatever desire the auto-biographer may entertain of appearing in a different character from his own, the man, as he is, will appear through every disguise he may assume. And so it is with medicine, the clinical cases, lectures, and remarks, which are published in the Weekly Medical Journals of London and Paris, have given us not only a professional, but almost a personal acquaintance with the distinguished men whose modes of practice, and even whose modes of thinking and of speaking, they so graphically describe.

The peculiarities which distinguish British from French surgery, are now as well known to the students in the Dublin hospitals, as to those who group round Mr. Brodie, Mr. Lawrence, M. Dupuytren, or M. Roux, in St. George's, St. Bartholomew's, the Hôtel Dieu, or the Charité. They can clearly perceive, that while the distinguishing character of the one is simplicity of practice, that of the other is complexity; and *perhaps* they may be disposed to think that, on some occasions, a *medium* might, with advantage, be found between 'the dose of house-physic and keeping quiet,' and the saignée, and sangsues, the boissons adoucissantes and delayentes, the potion calmante, the lavements and the demi-lavements, every third or fourth hour, and a variety of other remedies, which can leave the subject of so much skill and attention but little time for needful repose."

We have had a better opportunity of observing the practical working of the system of hospital reporting than Mr. Crampton. We do not hesitate to say that with much of good there is much of evil, and we own with regret that we see little likelihood of immediate improvement. In London there are three kinds of hospital reports, those furnished by pupils who conceal to the utmost both their names and occupation—those written by young men who are more or less known to the medical officers—and those supplied clandestinely, or at least without express acknowledgment, by the medical officers themselves. Of the first we can only say, that, so far as we have had an opportunity of comparison, and we have had such, they have been for the most part ridiculously incorrect, nor can we conceive the possibility of much good arising from them, at least under existing circumstances. The two last are always, or almost always genuine and authentic, but then they are liable to bias, and much of that *piquance*, which confers a flavour on the first description of report must necessarily be absent. On the whole we believe that the best, and fairest reports would emanate from intelligent and advanced pupils, known to the officers of the hospital, and yet not so closely connected with them as to be swayed by personal interest or private feeling. Perhaps it would be advisable that the reporter's name should be affixed to his report, and thus there would be every guarantee for authenticity, and the least objectionable safeguard against partiality. Some persons may object to this, that failures would not be published. They certainly would not be dramatized, nor is it right that they should be so. Would a private practitioner like his errors to be dragged before the public, blackened by private malignity, or caricatured by pert and vulgar ridicule. If a brother practitioner or haply an apprentice were to expose his little failings or even his great ones, and gibbet him to his circle and the world as no better than a malefactor, or no worse than a fool, would he not exclaim, and exclaim most justly, against the iniquitous brutality of the scoundrel. Yet what each and all would denounce if practised on himself, appears to be considered as tolerably just when employed against obnoxious hospital surgeons. Justice is justice, and what is criminal in the case of a private individual, is equally criminal, however the guilt may be veiled by casuistry or passion, in the instance of a public functionary.

We take it that the great object of hospital reports is professional utility—the diffusion of knowledge which would otherwise be buried in cloistered walls, or narrow circles. False knowledge is worse than none, in fact it can hardly be considered as knowledge at all. In our profession the basis of all reasoning is facts—our theories and our practice hinge entirely upon them. It is therefore our interest to take every possible precaution that our facts be true. Are accredited, educated, intelligent men—or unknown, imperfectly educated and irresponsible persons, the better calculated for purveyors of such facts ? We think that every rational, every unprejudiced person can form but one opinion, can make but one reply. Unfortunately, however, the mass of the profession is not unprejudiced, and unfortunately also the state of the medical constitution is such, that until very great alterations be made in it, prejudice and party feeling would turn the honey of Hybla to gall. Such are the demoralizing effects of an effete, and insulting, and pernicious system of medical monopoly, that its supporters, many of them possibly unwilling ones, are slandered and traduced without pity or compassion from the profession in general. Even honourable men have scarcely regretted that the advocates of a system so rotten and repulsive, should pay a sort of penalty for the public offence. We shall return to the subject of hospital reports and reporters at an early opportunity, for we do conceive that both might be rendered more useful to the profession, more calculated for the advancement and improvement of our science than they are at present.

Before we pass to the consideration of Injuries of the Head, we must advert to another point touched on by Mr. Crampton. It is the damnable system of blazoning as glorious and successful, frightful operations, which, in point of fact, end only in sorrow and disgrace. Those who are behind the scenes, who witness the practice of a large hospital, know that these bloody dramas are tragedies at last, and read with such disgust as Mr. Crampton has properly expressed, the lying chronicles of cures that abuse the credulous public. The well informed indeed are not deceived by this Fama mendax, but the mass of the profession are; for those engaged in country and in private practice, who have not the opportunity of witnessing such cruel displays, and cannot therefore contradict by experience and observation the delusive statements that meet their eye, are naturally and necessarily imposed upon. If hospital reports extensively adopted and faithfully executed had no other good result than that of disabusing the professional public of their admiration of bloody operations, they would not be unproductive of essential benefit.

But there is a medical as well as a surgical knight-errantry, if such a term be applicable to the case. The materia-medica becomes the physician's knife, with which he also aims at theatrical display. Those engaged in good private practice, and those who witness the results of public practice, soon find how false are many of the powers ascribed to new drugs and old ones. They learn to smile, or, if more sarcastic, to sneer at the miracles effected by iodine, or carbonate of iron, or by any other of the many medicines which enthusiasm, ignorance, impudence, or wit have invested with powers they do not possess. Here again is a field for the hospital reporter; a noble, because an useful opportunity of exposing charlatanerie, sobering enthusiasm, and detecting sophistry. We make no apology for having gone a little to one side in making these remarks; we sincerely believe that were they more attended to by the periodical press, it would execute its mission better than it does. That mission is Truth, and though we are not sufficiently Utopian to imagine that men will forget party, and prejudice, and interest to worship her, yet we believe that in all ranks of our profession there is a fund of good feeling, which, if properly directed by the press, cannot fail to redound to our honour and interest, and place us where we should be, in the van of civilization and intelligence.

Our readers are aware that Sir Astley Cooper and Mr. Brodie are inclined to advocate the application of the trephine in cases of compound fracture of the skull, whether symptoms of compression or irritation of the brain be present or not. It might naturally be imagined that a practice recommended by such authorities, should be very widely adopted in their own country, and, in fact, it is evidently becoming general. In Dublin, however, the lessons of Mr. Dease are still fresh, and continue to influence the opinions and direct the practice of his successors.

"I intimated at the beginning of this paper, that the modes of practice in certain severe injuries of the head, were somewhat different in London and in Dublin. The chief difference appears to consist in this, that in fracture of the skull with depression of the bone, the trephine is less frequently employed in Dublin than in London. In Dublin we conform in general with the rule of practice as originally laid down by Mr. Dease, (who preceded Desault by many years,*) namely, 'in fractures of the skull with depressed bone, *whether complicated with wound of the scalp or otherwise*, no attempt should be made to raise the depressed bone, *unless very decided symptoms be present of compressed or irritated brain.*' In London, on the contrary, the rule of

* "Mr. Abernethy is not correct in placing Desault before Dease. Mr. Dease's work on wounds of the head was published in 1778: Desault's thesis on his becoming a member of the College of Surgeons, is dated in 1776, and he was not appointed a surgeon to the Hôtel Dieu, until the year 1788.—*Eloge de Desault, par Bichat.*"

practice seems to be, 'that if the depressed bone be exposed in consequence of a wound of the scalp, the trephine is to be applied to elevate the depressed bone, *whether symptoms of injury of the brain exist or not*;'* either practice may be supported by the most specious arguments, and each has the sanction of the highest authority. It is plain, however, that in this case the question cannot be decided either by argument or authority, but solely by comparative experiments and observations conducted on a very extensive scale. I doubt, however, whether any individual, no matter how extensive his experience may have been, has instituted a sufficient number of experiments, or observed a sufficient number of facts, to establish definitively either of the opposite doctrines. In surgery, as in every thing else, extreme opinions, or opinions which are not qualified by exceptions and limitations, are seldom safe. I own, therefore, that the opinion of Mr. Dease,† qualified as it is by the exception in respect to those cases in which there are symptoms of compressed or irritated brain, has ever exercised a strong influence over my mind, and I may add, over the minds of all those who, like myself, received their earliest surgical impressions from the lessons and practice of Mr. Dease. I am quite aware that early impressions in favour of any particular mode of practice, are extremely apt to influence our opinions in after life, and that 'to seek the testimonies of truth,' (as Cicero beautifully expresses it,) '*ex animis consuetudine imbutis*,' is neither philosophical or safe;—but on the other hand, those who received their first impressions from the lessons and practice of Mr. Pott, are liable to a similar imputation of undue influence, operating in an opposite direction; we can only, therefore, approach to a solution of the problem, by placing in juxta-position, the *practice* of those who in different countries cultivate the same field of inquiry on a great scale, although by different means. The advantage of this method is well illustrated by what has happened within these very few years, with respect to the treatment of syphilis."

We agree with Mr. Crampton in considering the subject as yet undecided, and that not only do we require more facts, but that, from the difficulties surrounding the investigation, a length of time must elapse ere such facts can be properly collected, and accurately analysed. The question can only be settled by experience, careful observation, and rigid induction. By the way, Mr. Crampton mentions an incident illustrative of the barbarism of surgery, even at so late a period as fifty or sixty years ago. At the commencement of Mr. Dease's professional life, it was the duty of the senior apprentices to go to the hospital at an early hour on every Monday morning, and have all the persons who had suffered fractures of the skull during the festivities of the preceding Sunday evening, scalped, and ready for trephining at the visiting hour. Had such a story been told us by Sir Jonah Barrington, we should not have believed it. We proceed to the consideration of the cases related by Mr. Crampton.

CASE 1. *Compound Fracture of the Skull.*—James Fagan, æt. 23, tall and powerful, admitted into Steven's Hospital, at 12 P. M. March 17, 1832, under the care of Mr. Cusack. Half an hour before admission he had received a wound from a dragoon's sword, convex on its cutting edge, over the right parietal bone. When struck, he fell forwards, and lay insensible for five minutes, then leaped up and made a rush at the door, but fell again. He had been drinking previously. On admission he was violent and

* "See Sir Astley Cooper's Surgical Lectures in the Lancet. Mr. Brodie's Paper on injuries of the head.—*Med. Chirurg. Transactions*, vol. xiv. p. 412."

† 'The more eligible and rational method of treatment in all those cases, (fracture with depressed bone,) I believe will be, that of not being precipitate in applying the trepan, as long as the patient remains free from symptoms that would indicate the necessity of it, &c.'

incoherent—vomited twice—skin warm —pulse 92, and weak. The sword had cut through the bone and membranes of the brain, some cerebral substance appearing on the lips of the wound.

On the 18th, he lay as if asleep, moaning and answering peevishly, but often intelligibly when roused and questioned —when much roused he could tell where but not how he received the injury—said he had a pain in his head, and pointed to the wound with his hand—looked like one oppressed with drink and sleep. Surface warmer than natural—pulse 88, small, variable, quickened by any motion—tongue moist, brown in centre—pupils contractile. He had some cough which seemed to distress him, and had vomited four or five times during the night. On changing the dressing much cerebral substance, mixed with blood escaped.

*V.S. ad* ℥xvj.—*Cal. c jalap. D. febril.*

19th. Vomited after bolus, which has not operated—urine was drawn off last evening, but has been passed this morning—got some sleep. Is more sensible, and reluctantly answers questions—pulse 75, soft, regular—tongue brown in centre. *Enema purgans.*

20th. More torpid—moans frequently, and complains of pain in the head—pupils natural—pulse 55 when quiet, when disturbed 80—no stool. Wound glued together by lymph and pus.

*V.S. ad* ℥xx. *Cal. c. colocynth. Enema purgans.*

21st. Bowels well opened—less stupor —pulse 68—wound looks clean.

*Hirud.* xxiv. *capiti. Enema purgans. Cals.* gr. ij. *3tiis horis.*

"He continued much in the same state, but gradually becoming more sensible, until the 24th, when he appeared more torpid, and at 4 o'clock P. M. he had a violent convulsive attack, four men could scarcely keep him in the bed; three hours afterwards he had another fit equally severe, which was followed by great stupor. *As there were now evident symptoms of irritated, and probably compressed brain,* it was determined, in consultation, by Mr. Crampton, Mr. Peile, Mr. Collis, Mr. Wilmott, and Mr. Cusack, to lay bare the wound in the skull freely, and then to act according to circumstances respecting the removal of a portion of the bone. As a considerable quantity of brain had escaped from the wound in the first instance, it was obvious that both tables of the skull, the membranes of the brain, and the brain itself, had been divided by the sword, and it was probable that the inner table of the skull had been separated from the outer, and driven in upon the brain: and such, upon a careful examination with the probe, seemed to be the case. A slip of bone about ¼ of an inch broad, and three inches long was removed from the upper edge of the divided bone, by means of a *straight saw*, about four inches in length; the operation was performed by Mr. Cusack, and was effected in a very few minutes, and with great ease. On removing the slip of bone, it was ascertained that the inner table was detached, so as to form an acute angle with the outer table. Some softened cerebral substance escaped from the wound; he had one slight convulsive attack after the operation, but he soon became much more sensible, and now (four hours after the operation) he lies tranquilly with his eyes open."

At 10 P. M. he had another convulsive attack, but next morning was free from stupor. He complained of pain in the head—pulse 76, small and weak. On the 27th (10 days from the accident) a small red pulsating tumor, the size of a pea, appeared in the centre of the wound. He was ordered bark. On the 28th the fungus was doubled in size, of light purple colour, pulsating slightly; the wound was granulating, the general symptoms favourable. No material change occurred till the 13th of April, when the fungus began to enlarge considerably and to pulsate strongly, he was more torpid, and complained of pain in the head. About the 16th the fungus began to decline rapidly in size, the general symptoms likewise becoming more favourable. On the 20th the fungus had disappeared, and on the 1st May the wound was healed. On the 15th Fagan was discharged, and was able to resume his work as a pipe-maker. His memory was impaired, and when seen on the 20th July it continued so. He had forgotten *words*, not *things*, and told Mr. Crampton that "he knew every thing as well as ever he did, but could not put a name on any thing." Mr. Crampton shewing him a button, he laughed, and said "I know what it is very well, it is a ba, ba, ba,—Och! I can't say it, but there it is," pointing to a button on his own coat.

Mr. Crampton thinks it probable that, had the operation been performed earlier, the result would have been unfortunate, and that had it been performed later, it would have but aggravated the mischief, by irritating parts already in a state of incipient inflammation. This, however, is speculation, and the fact, as it was, is more valuable than Mr. Crampton's belief of what, had it not been as it was, it might have been. The following remark is more to the purpose.

"The convulsions which are caused by *irritation* of the brain, and which, not unfrequently appear shortly after a severe injury of the head, are of a very different character from those which are caused by the disorganization of the brain, consequent on *inflammation*. The first appear to be an epileptic character, and are probably connected with a disordered state of the functions, rather than of the structure of the brain, and are *comparatively* attended with but little danger.

The second usually appear in connexion with strabismus and coma at the termination of the train of symptoms called 'secondary,' from their occurring several days, or even weeks, after the injury has been inflicted, and it is scarcely necessary to add, that they are almost invariably the fore-runners of death."

The fact is that the subject of convulsion after injuries of the head has not been sufficiently investigated. For some valuable remarks on this symptom we may refer to Mr. Brodie's excellent paper. It seems probable that convulsion, as such, is not a symptom of much pressure or even disorganization of the brain. We see it after slight injuries, after depletion without injury at all, and as Mr. Crampton justly observes, it sometimes accompanies other and more deadly symptoms. If Mr. Crampton implies that the convulsion in the latter case differs in kind from the convulsion in the former, we doubt the correctness of his opinion. We have seen no decided difference in the character of the convulsions—the real difference lies in the character of the attendant phenomena. Yet surely this proves, both in logic and in common sense, that convulsion, of itself, is not to be looked upon as a deadly or even a very alarming symptom. We repeat, however, that the subject requires investigation.

Mr. Crampton dwells upon the fact, that an operation may be resorted to with advantage, even after the appearance of symptoms, which, if not relieved, invariably proceed to a fatal termination. In proof of this he cites some cases reported by Mr. O'Halloran, in the fourth volume of the Transactions of the Royal Irish Academy, "a work not usually found in medical libraries." The cases are too short to be further condensed.

CASE 1. "Pat. Kelly received repeated blows on the left parietal bone, which produced a very extensive fracture, with a contused wound of the integuments. He went on tolerably well for about ten days: he then became heavy and drowsy; the complaints increased, and when I was sent for, he was comatose, and so much oppressed, that I apprehended an operation would be useless, and had some thoughts of immediately returning; but on reflecting on the great resources of nature, I removed the integuments, applied the trephine, and elevated the depressed bone. Immediately after the operation, he opened his eyes, knew me, and spoke. Eight days afterwards I removed a loose piece of bone of considerable size. The next day he had paralysis of the opposite side: he laboured under this partial paralysis for fifteen days and then gradually recovered.

CASE 3. I visited Pat. Hayes, who 13 *days before* had received a blow which made a profound depression on the posterior superior part of the right parietal bone; the pulse was slow and weak, but regular; he was quite comatose, and could not articulate. The bone was crushed into bits, and was driven in upon the brain, wounding the dura mater in some places. By means of the probe, forceps, and elevator, (without the trephine,) I relieved the dura mater of all incumbrance. In some time the dura mater rose to its natural height; the diseased parts sloughed off, and he recovered."

To shew that extraneous matters may frequently be left in the brain for some time without injurious consequences, Mr. Crampton relates an interesting case, that of Mr. Henry Brougham, nephewof the present distinguished Chancellor. Mr. Brougham, then 18 years of age, received the contents of a fowling-piece obliquely in his forehead. He was bled by a country surgeon, and soon afterwards seen by Mr. Crampton. He was perfectly sensible, and had a faint recollection of the manner in which the accident had happened. The eyes were closed by the swelling of the eyelids and cheeks—the principal wound occupied the centre of the forehead; it was circular, above two inches in diameter, and nearly an inch in depth, the bottom pulsating, and covered with a mixture of coagulum, disorganized brain, fragments of bone, and some of the wadding of the gun. Mr. Crampton attempted to clear the wound of the extraneous matters lodged in it, but scarely had he touched with the forceps a large fragment of bone, when the whole body was convulsed and he moaned deeply. Mr. Crampton then merely attempted to cleanse the wound with tepid water. The patient instantly cried out that the sensation was dreadful. Mr. C. then gave up all attempt at even washing the wound, and merely covered it with a piece of soft lint. The treatment consisted in leeches daily to the forehead and temples—iced water to the head—attention to the bowels—starvation. The local treatment was equally simple. The discharge contained, for several days, much softened brain, mixed with blood, but the wound itself was not washed for twenty-two days, and then nothing more was done than to lift off the small fragments of bone as they became detached by the natural process. The wound was healed in two months, but small spiculæ of bone were occasionally discharged for a year afterwards. The cicatrix was deeply depressed, and pulsated. Mr. Brougham resumed his collegiate studies, graduated, and became a valuable clergyman. We are informed that Mr. Crampton's reports are to be continued. We hope that they will be so; and we beg to hint to Mr. C., that the profession will expect from him some of the many interesting *facts* that must have fallen under his observation.

---

XXXVII.

Clinical Reports of the Surgical Practice of the Glasgow Royal Infirmary. By John Macfarlane, M D. senior Surgeon to the Royal Infirmary of Glasgow, &c. Octavo, pp. 314. Glasgow, 1832.

Clinical reports, in one shape or other, are pouring in upon us. We hail this as a good sign—good for the science—good for the profession. Having made some remarks upon the subject in another article, we shall not go over the ground again. Dr. Macfarlane's short preface will shew why he has been led to publish these clinical reports, and will shew, at the same time, why other surgeons or physicians, with equal or greater opportunities, should follow his example.

"Having ceased to act as one of the attending Surgeons of the Glasgow Royal Infirmary, it occurred to me that a plain and candid Report of the unsuccessful as well as of the successful cases, which had been under my care in that Institution, might be useful to the numerous classes of students who attended me in my daily visits, and, probably, not unacceptable to some of the junior members of the profession. The short time that has elapsed since this resolution was formed, the pressure of other important avocations, and my anxiety to have the work completed as soon after the termination of my attendance as possible, are the only apologies I have to offer for so hurried and imperfect a performance. I have endeavoured, however, to give a faithful detail of all the cases of importance, appending to them such observations as they naturally suggested : and, at the same time, I have made an unreserved disclosure of all the adverse occurrences which I have met with in Hospital, and, when the subject admitted of it, even in private practice. All the operations which proved fatal are candidly recorded, and the causes of failure pointed out, from a conviction that by such details science is more likely to be benefited, and the surgical student improved and instructed, than by merely disclosing the '*cures*,' however numerous or brilliant.

These Reports embrace but a very limited period ; they include, however, the whole time of my attendance as junior and senior surgeon, viz. from 1st May to 1st August, 1826, when only one surgeon officiated, and the attendance was limited to three months; from 1st November, 1826, to 1st May, 1827, when the period was extended to six months, and two surgeons acted ; and from 1st May, 1831, to 1st May, 1832, when the time of attendance was increased to one year, which arrangement is at present followed.

It cannot be denied, that the public at large, by whom our Hospitals are so munificently supported, and especially the medical world, are entitled to look to the Physicians and Surgeons of such establishments for the results of their experience, whether fortunate or adverse, in the shape of a periodical publication. Were such documents regularly furnished by the attendants of British hospitals, as is occasionally done upon the Continent, and thus made available to the profession, a mass of invaluable materials would be soon accumulated, which would far exceed in value and importance all the isolated papers and cases with which the journals of the day abound. It is not, however, in the power of the surgeons to the Glasgow Infirmary to furnish any thing like an extensive Report, the period of their attendance being so limited as to prevent any single individual from obtaining that ample experience, or making that extended series of observations, which close and assiduous practice in an hospital for years can alone render important. Nevertheless, it is their duty to communicate the results of their experience, limited though it be ; and should they not be able to give to the public much that is interesting or valuable, they may at least show that they have not been inattentive to the duties of the important office to which they have been appointed."

The volume before us is rich in facts, and those of the kind which we require. We have failures presented as well as successes, and the profession can judge of surgery as it is, and not as vanity would wish that it should be. It is such men as Dr. Macfarlane who are calculated to serve the real interests of truth, and to raise the moral character of the profession. How noble a contrast does his plain and manly conduct present to that of the shuffling advocates of a special remedy. The present Number of this Journal will afford discerning men the means of comparison. We shall lay this volume under an embargo, in order that we may rifle it of its valuable contents. Dr. Macfarlane will be pleased to see his cases made far more extensively useful, than the limited circulation of a medical monograph could possibly permit. As the book is a mass of arranged cases, we are not tied down to an analysis or a review, but may suck, at our convenience or caprice, the honey from its numerous and varied flowers.

### I. LUPUS.

"This disease is met with in two different forms. The first and most common variety commences in a slight papillary enlargement of one or more of the sebaceous glands and follicles of the nose, cheek, or lip, which parts soon assume a dark red or livid colour. They are at first indolent, but gradually become itchy and painful, ulcerate on the surface, and discharge a thin ichorous fluid, which, on exposure to the air, becomes concrete; thus forming, over the whole affected surface, crusts or scabs of a yellowish or dark brown colour. These soon separate, and new ones are reproduced; and if the disease is not checked, it gradually extends, and more complete ulceration ensues, by which the original characters of the affection are modified, and in some measure obscured. The ulceration, however, is generally superficial, being rarely deeper seated than the skin, or accompanied with much swelling of the surrounding soft parts; and it seldom produces the ravages or deformity of the second species, which I shall now shortly describe. The sebaceous glands and follicles are here also the seat of the disease; but in general there is only one tubercle, which enlarges slowly, assumes a purple or violet colour, has a broad, firm, and indolent base, and presents many of the characters of a small carbuncle. This lupoid tubercle is slow in its progress, and may remain for a considerable time discoloured and indolent before ulceration takes place. In one case that came under my notice, the tumour, which was the size of a walnut, resisted every kind of treatment, for fourteen months previous to the occurrence of ulceration. When this process does commence, however, it spreads deeply, and produces great destruction of the soft parts, as well as of the cartilaginous and bony structures with which it comes in contact. I have seen the nose, the upper and lower lips, the cheek and the right eye, completely destroyed by it, with the nasal, and a portion of the superior maxillary bones."

Case 1.—*Lupus affecting the Nose, &c. cured by Arsenic.*

C. M'K., æt. 9, was admitted into the infirmary on Jan. 4, 1831, under Dr. Perry. On the 1st May she became the patient of our author. The upper lip, and apex and alæ nasi were covered with yellowish scabs, under which the ulcerated surface had a florid papillary appearance, and discharged a thin matter; a part of the septum and alæ nasi was destroyed.

There was some difficulty of swallowing from great swelling and irregularity of both tonsils, which were much thickened, superficially ulcerated, and tuberculated; the uvula also enlarged, adhered firmly to the left. The ointment of the iodide of mercury had been used for several weeks without advantage. A solution of the white oxide of arsenic in water, grs. vj. to the oz., was ordered to be applied to the ulcerated surface twice daily, was continued for several weeks, together with small doses of Fowler's solution of arsenic internally. The parts cicatrized several times, but in a few days the ulceration returned. At length two applications of the actual cautery, followed by the arsenical lotion, produced a more permanently healthy action, and she left the infirmary cured on the 17th September.

"I have great faith in arsenic as a local application in lupus, and am in the habit of employing it for two different purposes. When used as a stimulant, it must be applied in solution, but when an escharotic effect requires to be produced, the white oxide mixed with an ointment, or made up in the form of paste, is laid over the affected surface. Dupuytren has frequently observed, that when the arsenical paste is applied to lupous ulcerations, extensive erysipelas of the surrounding integuments is not unfrequently produced, and he therefore prefers applying the proto-chloride of mercury in powder, which he says is free from this objection, and gradually but steadily changes the morbid state of the parts, acting more as a specific than a caustic. Although I have never seen the arsenical paste give rise to erysipelas, yet in two cases in which the ulcerated surface was extensive, its application was followed by severe inflammation of the gastro-enteritic mucous membrane, apparently in consequence of its absorption into the system; and in another case, the slough which it produced penetrated so deeply as to expose the cartilage of the nose, a portion of which exfoliated. When, however, the diseased surface is small, and the lupus exists in the form of tubercle, the arsenical paste may be employed, not only with safety, but also with marked advantage, for it destroys the ulcerated surface and the surrounding induration more completely than can possibly be effected by applying it in solution. But in every other state of the disease, the latter formula is the safest and most efficient,—its strength being so regulated as to produce either a stimulant or escharotic effect. I have employed it for the latter purpose in several cases, and have never seen the most concentrated solution, even when applied to irritable and extensive surfaces, produce local mischief or constitutional disturbance.

The other stimulating applications which I have employed with most advantage, are the actual cautery, and a solution of mercury in strong nitric acid. The red-hot iron has a powerful effect in changing the morbid action, producing healthy granulations and speedy cicatrization. I have employed it in several cases of lupus with decided benefit, and am in the habit of doing so with similar good effects in other forms of ulcer, both indolent and irritable. I have only once employed the '*nitrate acide de mercure*,' which Richerand and Cloquet have found so frequently useful when other applications had failed. It produced a slough, which was three times removed, when a cure was accomplished. In this case the right nostril was nearly shut up by an enlarged, ulcerated, and granular state of its mucous membrane. This diseased state was removed, and occlusion of the external aperture prevented by the introduction of sponge."

In the following case the obstruction of both nostrils was complete, and the deformity required removal by an operation.

CASE 2.—*Lupus affecting the Nose, &c. and producing obliteration of the Nostrils—removed by an operation.*

W. M., æt. 18, was admitted March 21, 1826, under Dr. Young. The disease had commenced 18 months previously on the nose, cheek, and centre of the upper lip, by dark redness, tumefaction, and itchiness, slowly succeeded by ulceration; the parts then becoming covered with thick yellowish-green or dark brown scabs, extending along the septum nasi, and accompanied with a purulent discharge from the nostrils. Three applications of the actual cautery and the use of resinous ointment seemed to effect a cure; but on the 1st of May, when Dr. Macfarlane took charge of the case, the diseased parts were covered by a delicate, smooth, and shining cuticle, and were elevated, irregular, and apparently unsound. In a few days ulceration recommenced, scabs forming, dropping off, and being speedily renewed. Under the arsenical lotion and Fowler's solution internally a gradual improvement took place, and the patient was dismissed cured on the 26th June. On the 24th August the disease re-appeared after the reception of a smart blow on the nose. On Feb. 16, 1827, he re-applied at the infirmary. The external parts were healed, but the opening into both nostrils was completely obliterated by the firm union of the alæ with the septum. The adhesions, which were firm and extensive were divided by a narrow bistoury, and sponge tents were afterwards introduced. When these were discontinued the parts healed under the application of the nitrate of silver.

"Although lupus may be purely local in its origin, yet it sometimes happens that its continuance is prolonged, and the cure materially impeded by constitutional causes. I have seen, both in the Infirmary and in private practice, several children labouring under this disease, who had been subject for years to psoriasis, tinea capitis, or other cutaneous complaints. It has been also observed to prevail most obstinately among those of a scrofulous diathesis, especially while labouring under enlargement of the lymphatic glands, affections of the bones, chronic ophthalmia, tabes mesenterica, &c. According to M. Rayer, it has been at times particularly prevalent among the poor in some parts of France, from scanty and unwholesome nourishment; and in this city, during the years 1818 and 1819, when the working classes were exceedingly ill fed, I had occasion to see a greater number of cases among the poor in the district which was then under my care than at any former or subsequent period. These facts are sufficient to shew the necessity of combining constitutional with local treatment. With this view I generally order Fowler's solution of arsenic in small doses; but I have sometimes found it necessary to prescribe the muriate of mercury, dissolved in a bitter tincture, such as that of cinchona, colomba, or gentian, so as to produce a mildly-alterative effect."

Case 3.—*Tubercular Lupus mistaken for Cancer, cured.*

W. P., æt. 44, was sent from the Highlands to undergo an operation for cancer. On the 14th June, 1826, the following was his condition. Two inches of the central portion of the upper lip were destroyed by ulceration—the sore looked angry—its edges were thin and ragged—its surface irregular, and covered by a greyish secretion through which small purple granulations were visible—the discharge was bloody and fetid—and the surrounding integuments were livid, indurated, and the seat of darting pains. The nose was covered with a number of small, prominent tubercles, to the apices of which were attached hard yellowish scabs. The disease had existed for nearly two years, ulceration having occurred eight months after the first appearance of the tubercle in the lip, during which time several of the lymphatic glands on both sides of the neck became enlarged and painful. The arsenical lotion was used by Dr. Macfarlane, at first as an escharotic and afterwards as a stimulant, whilst small doses of the hyd. mur. were given so as to affect the gums. By these means the morbid action of the parts was arrested, and in less than two months a complete cure was effected. Though much of the lip was destroyed, yet, as granulations formed, the parts were gradually approximated by means of sticking plaster and a double-headed roller, and ultimately re-union took place with but little deformity.

"Sometimes the progress of a lupoid tubercle may be arrested, and its ulceration prevented, by the application of leeches around its base, especially when the tumour is painful and covered by inflamed integuments. This followed by evaporating lotions, and alterative doses of calomel, will not unfrequently subdue the inflammation upon which the progress of the disease depends, and reduce it to that indolent and chronic state, in which friction, with an ointment containing the ioduret of zinc or mercury, may be beneficially employed to promote its absorption."

We have given all the cases of lupus related by Dr. M. as well as the practical remarks accompanying them, because we know how important it is to understand the treatment of this most distressing complaint. We might offer some observations of our own, but as we are anxious to place before our readers as much as possible of the valuable matter contained in the present volume, we will not stop to indulge in any. We will merely say this, that in lupus, as in other maladies, the practitioner must not neglect the general principles which regulate all treatment, even the most specific. He must see what is wrong in the general health, and endeavour to set it right, and he must recollect that the state of the parts in lupus is liable to variation. Occasionally we find them inflamed—occasionally indolent. When scabs form it is better to apply a light bread poultice until they are separated, and then resort to the applications determined on.

### II. Lumbar Abscess.

Dr. Macfarlane remarks that lumbar abscess has become a more manageable disease since the general adoption of Mr. Abernethy's practice—that of opening the tumor frequently by a small puncture, healing the wounds, and allowing the sac to contract. This perhaps is not so certain as Dr. Macfarlane imagines, indeed we suspect that at present Mr. Abernethy's method is rather declining in general estimation. At St. George's Hospital, for instance, where free openings, and punctures, as recommended by Mr. Abernethy, have been tried extensively and compared together, the latter are nearly abandoned. Mr. Brodie, whose opportunities have of course been great, and who has paid no inconsiderable degree of attention to the subject, prefers free openings and the subsequent application of a poultice, in order that the matter of the abscess may drain out. Mr. Brodie has been led to give a preference to this practice from having observed that, after closing a small wound, the contents of the sac became putrid under the admission of a little air, when sulphuretted hydrogen gas was generated, and the usual remarkable symptoms of a putrid abscess ensued. We remember, ourselves, having witnessed a case of this description. A patient had psoas abscess, which presented in the thigh. It was opened, but at this monent we do not remember how, and after the puncture the wound closed. On the next or ensuing day peculiar symptoms of prostration with fever of a typhoid character set in. The wound was immediately broken open, when coagula of blood that had been locked in and putrid gas escaped. We believe that the patient died. But we return to Dr. Macfarlane.

Dr. M. observes that before puncturing the cyst, which, it will be recollected, he does in the Abernethian manner, he has derived much advantage from establishing an issue over the lumbar spine; he has also irritated the depending part of a lumbar abscess by the moxa, when, from non-adhesion of the puncture, there was reason to fear that inflammation of the cyst would supervene, and this has been attended in several cases with decided advantage.

CASE 1.—*Lumbar Abscess successfully treated by Issue, Moxa, and repeated puncturing.*

W. J. æt. 18, admitted July 14, 1826. The abscess presented below Poupart's ligament on the left side. He complained of pain in the left iliac and lumbar regions, but no tenderness, nor alteration of the spinal column could be detected. He was of strumous aspect—his strength reduced—there were frequent dry cough, hurried breathing, and night sweats—he could not stand erect, or walk without keeping the trunk bent forward at a considerable angle—pulse 120—pyrexia. The tumour had been first observed at the upper part of the thigh five weeks previously, but for several months he had been subject to pain in the back, and weakness of the limbs.

"A small caustic issue was formed on the left side of the spinous process of the second lumbar vertebra; and after the eschar had separated, and the discharge was established by the insertion of peas, the abscess was punctured, and about eight ounces of well-matured pus (being apparently about two-thirds of its contents) were evacuated. The edges of the wound were brought accurately together, and adhesion effected. He was allowed a milk diet, with a small quantity of wine, and six grains of quina daily; his strength and appetite gradually returned, his pulse fell to eighty in the minute, and the diarrhœa and perspirations ceased. The sac was punctured again in ten days, and the wound closed; but, on repeating this operation for the third time, about a fortnight after, adhesion did not take place; there was, therefore, a daily discharge of pus from the opening for about three weeks, when it completely closed. On the third day after the last puncture was made, he had a rigor, which was followed by pain in the lower part of the abscess, and by a fetid and brownish-coloured discharge. The application of a moxa over the most depending part of the cyst, and of another over Poupart's ligament, checked these symptoms, and restored the purulent secretion to a more natural appearance."

The following case is one of not very unusual occurrence.

CASE 2.—*Lumbar Abscess—Diseased Spine not discovered till after death.*

A. S. æt. 22, admitted Dec. 13, 1826. A large abscess presented under Poupart's ligament on the right side. No disease of the spine could be detected. The patient was feeble, with little pyrexia. The tumor had been first observed seven weeks previously, but he had been out of health for nearly three years, when his left leg was amputated for scrofulous disease of the knee-joint. Three moxas were burnt over the lumbar spine, after which a pound and a half of curdy pus was evacuated by puncture. The wound was closed before more than two thirds of the contents of the sac had escaped. In two days he had a rigor, followed by pyrexia, and pain in the tumor. On the 19th the bandage was removed, and a pound of flaky pus escaped from the opening. Before the tumor was more than half emptied the wound was again closed, and compress and bandage applied. On the 21st three pounds of pus were discharged; he had now acute pain in the left side of the chest with dyspnœa, and rapid feeble pulse. The pain was removed by local bleeding and fomentations, but vomiting, diarrhœa, and aphthæ came on, the discharge became brownish coloured, fetid, and mixed with air, and he died on Jan. 7.

*Sectio Cadaveris.* The cavity of the abscess extended from the right side of the spine close to the diaphragm, along the psoas, to the middle of the thigh. The sac was thickened and of chocolate colour, its surface in some places granular, and covered with lymph; through its posterior wall was a small opening, opposite the upper edge of the sacrum, and extending to the two inferior lumbar vertebræ, which, together with the articulating surface of the sacrum, were carious; a considerable portion of the intervertebral cartilage was destroyed.

It is not an uncommon circumstance for the spine, or the bones of the pelvis, to be extensively affected, without any symptom to excite suspicion during the life-time of the patient. The fact is, that a large abscess in the loins, lower part of the belly, or upper part of the thigh should, of itself, lead a surgeon to *suspect* diseased bone. We have formerly related a case of extensive disease of the thoracic vertebræ, in which the patient complained only of pain in one hip-joint. In this case there were carious ribs, and two ulcerated openings in front of the chest led to the affected bones. This patient died of inflammation of the pleura. We lately saw another instance of extensive disease of bone, unaccompanied with prominent symptoms. The patient was an old man, of sallow, unhealthy aspect, and presented himself with chronic abscess of the upper and posterior parts of the thigh, much below Poupart's ligament, and apparently unconnected with it. The tumor was punctured twice, and after the second opening symptoms of inflammation of the interior of the cyst, with secondary hæmorrhage, ensued. He died. On examination, the cavity of the abscess was found to be enormously large, and extended up under Poupart's ligament to the cup of the ilium. Here the whole surface of the bone was bare and rough, and there was also disease of the sacrum and lower lumbar vertebræ. The abscess had only been observed for two months, and up to the patient's first application he had walked about.

Diseased bone, in these cases of large abscess, is frequently not only not suspected during life, but even overlooked after death. Mr. Brodie has remarked, that a small opening occasionally leads down to exposed or carious vertebræ, which opening may be, indeed has been, overlooked. Surgeons, in their examinations, should be careful to attend to this hint of Mr. Brodie's, or they may unwittingly deceive themselves and others. At the same time, there can be no doubt that large abscesses do occasionally form in the neighbourhood of the psoas, the lumbar, the glutæal, and the abdominal, as well as other muscles, independent of any affection of bone. The cellular membrane is, we know, extremely liable to suppuration, and there is no good reason why this tissue should be exempt in the situations alluded to. Every practical surgeon will agree with us, in believing that an accurate knowledge of the various abscesses that present about the groin and inguinal region is of great importance. Before we pass to the relation of another case, we must express our surprise at the rapid manner in which some of these enormous abscesses are cured, or seem to be so. We lately saw a patient with a very large abscess in one natis; it was punctured, and nearly 40 ozs. of pus discharged; filled again, and was again opened, no precautions being taken to close the wound. The patient who was reduced to death's door, acquired flesh and strength under generous diet and bark, and the opening, which at first seemed inclined to be fistulous, healed. No exposed bone was at any time discovered. Dr. Macfarlane gives a fatal case of lumbar abscess, which is interesting, as it shews the kind of symptoms that occasionally follow the operation of puncturing.

CASE 3. *Lumbar Abscess—fatal Irritative Fever and Effusion on the Brain after Puncture.*

W. D. æt. 12, admitted Dec. 28th, 1826. There was a lumbar abscess projecting from the inner and upper part of the left thigh, and nearly as large as a child's head. There was fixed pain in the lumbar spine, increased on pressure and motion, especially at the lower part, where two spinous processes projected, and the integuments were thickened and painful. Several inguinal and cervical glands were enlarged. The tumour had appeared five weeks before admission, but he had suffered under pain and weakness of the back for some years.

A caustic issue was applied, and, on the 2d Jan. a puncture was made. The wound did not heal; and, on the 4th, the bandage was removed, and 12 ozs. of matter escaped. In the evening, there was a slight rigor, followed by pyrexia and acute pain in the lumbar spine. For several days, the pulse was from 120 to 140—the skin hot and dry, with vomiting and hiccup—flushed face—hurried breathing—typhoid sordes on the tongue and teeth. There was no pain in the abscess, the discharge from which was diminished, and more like whey than pus. The pain in the spine became so excruciating that he could not bear the slightest motion—he was occasionally delirious, and died comatose on the 19th.

"On inspection, the cyst was found to be considerably larger and more extensive in the abdomen than exterior to it, and to extend from above the origin of the psoas muscle to the middle of the thigh. It was free from inflammation, and covered by curdy matter, which adhered firmly to its inner surface. The lumbar vertebræ were curved to the left side, and the three inferior bones carious, and surrounded by pus. The intervertebral cartilages were ulcerated, and an incipient psoas abscess, about the size of an orange, was discovered on the right side of the spine. That portion of the spinal marrow enclosed in the affected bones was softened, the brain was in a state of congestion, there was effusion under the arachnoid membrane, on the surface of the hemispheres, and each lateral ventricle contained about half an ounce of limpid serum."

We shall return to the rich harvest of facts presented in this volume, and we hope to glean many instructive cases from its pages.

---

### XXXVIII.

### AFFECTIONS OF THE HEAD PRODUCED BY QUININE.

QUININE was found to produce some remarkable affection of the head, in almost every case in which it was administered at Jubbulpore, in 1829. In one officer it caused transient deafness; in another vertigo; in a third intolerance of light, to such degree, that the medical men were alarmed lest effusion on the brain should take place. A fourth European was subject, for a short time, to much confusion of ideas, and all sorts of chimeras after taking Quinine. A Portuguese was affected with tinnitus aurium, and deafness. In all these cases, the Quinine acted favourably on the fever, for which it was administered. In the same note, favourable mention is made of the efficacy of Rohena Bark (Swietina Febrifuga) in fever cases; but large doses of that remedy were also found to produce vertigo. — *Calcutta Transactions, Vol.* V.

---

### XXXIX.

### Reported Diuretic Properties of Lichen Vulgaris.

The Lichen Vulgaris, is found in abundance at Bangalore and Trichinopoly; its Tamul name is Kull-pashie; and its decoction in milk is used by the natives in that part of India, as a diuretic; but they are not acquainted with its efficacy when applied as a poultice over the loins. Dr. Stevenson recommends the Lichen to be boiled in water, and bruised in a mortar; it is then to be applied over the region of the kidneys, as a poultice, and renewed twice a day: using at the same time only laxative medicines, when requisite. Dr. S. mentions the case of a man, who had suffered for many months from dropsy, his abdomen being very tumid and undulating; urine scanty; extremities œdematous; countenance bloated, and skin harsh and dry. A poultice of Lichen Vulgaris was applied over each kidney, and produced its diuretic effects so freely, that a quart of urine was voided every second hour, to the interruption of rest. In ten days, the circumference of the man's belly had decreased a foot: the dropsy was cured, and the man's health restored. The remedy was used in July, 1829, and the patient remained well when last heard from, 16 months after. The 2d case was a Dragoon, who had Anasarca, the symptoms of which were speedily removed, but the disease returned, and the man died; when the dropsical symptoms were found to be connected with disease of the heart. Dr. S. knows of the Lichen being used in a third case, with benefit; but the patient was not under his care, and used other medicines at the same time; therefore, the effects of the remedy might be doubtful in that case. The Lichen Vulgaris is commonly found in the Deckan, growing on rocks, and the lower branches and denuded roots of the larger trees.—*Ibid.*

---

### XL.

### Cases of Abscess of the Liver.

The following three cases of abscess of the liver are to be found in the Transactions, from which the two preceding articles are taken. They were communicated by the Medical Board of Bengal, and are not devoid of interest.

*Case* 1. The patient was an European, 21 years of age; a man of steady, good conduct, and respectable connexions. He had appeared for a long time in delicate health, and depressed in spirits, but had never been in Hospital for any affection of his liver; nor was he known to have made any complaint of pain in the side, until within three days of his admission into Hospital, on the 14th September 1830. At that time he suffered from Pyrexia, and acute pain across the stomach, increased by pressure. The pulse was strong and rapid, but subdued by a bleeding of only 1 ℔ after which 12 leeches were applied. He was further treated by active purgatives, calomel, and antimonials, and had a blister. The patient had a severe rigor on the 15th September, which, by his account, was only the 5th day after he first felt the pain at the Epigastre. On the 19th September fluctuation was evident between the ensiform Cartilage and Umbilicus; and on the 20th, in the afternoon, a large quantity of matter, coagulated blood, and sloughs, were passed by stool. The pulse before this had hardly ever exceeded 100; it now became very frequent and weak, and the patient sunk rapidly. The fluctuation at the Epigastre was so very evident on the 20th, that the Surgeon was induced to make an opening into the tumor with a lancet, by which some pus escaped; the patient experienced no relief from this operation; but sunk gradually, and died on the morning of the 22d. On dissection, the liver was found much enlarged; the right lobe gorged with blood, and softened; the left lobe is described as one large abscess, from which an opening had taken place into the colon: and the liver was adherent to that intestine.

The reporters observe that matter had probably formed before the patient's admission into hospital on the 14th. But it must be remembered that the first rigor was on the 15th, and the patient had only been ill for five days. The case certainly shews the intractable nature of acute hepatitis, unless early and actively treated.

*Case* 2. This patient, also an European, aged 26 years, had an attack of fever on the 14th May, 1830, brought on by, or at least preceded by drunkenness; the fever continued for a week, with slight occasional exacerbations; the head chiefly affected: he was purged by calomel and salts, and kept on low diet. On the 21st May he had pain in right side, and "quick pulse," but cool skin; was bled to 1½℔ with very little benefit. On the 22d, symptoms being unabated, he was again bled to 20 ozs. and in the afternoon 30 leeches were applied to the right side, whereby the pain was moderated, but not removed. Purgatives, with calomel and antimonials, were employed daily, and the mouth affected by the mercury. On the 24th, pain of right shoulder is mentioned, and the pain of side was gradually increasing, until 27th, when 30 leeches were again applied to the side, and repeated on the 28th, by which his strength was reduced, and he is stated to have "fallen down in a fit;" however, the pain of side was not removed. Purgatives were used daily, and on the 29th he was ordered tartar emetic, ¼ of a grain, every 2d hour. On the 31st May, oppression at chest was felt; and he had a caustic issue to the side. On the 2d June, pulse was observed to be 112. And on the 4th, the side is reported just the same, in respect to pain. One drachm of nitric acid was now prescribed in drink, daily; and the patient was purged with extract of jalap. On the 9th and 10th the pain of side somewhat alleviated, and he took aperients of rhubarb and ginger; or of salts and infusion of Cheraytta, for some days. On the 10th June there was an increase of pain in the side, affecting the respiration, and a small quick pulse; the skin cool. He was bled to 18 ozs. with some relief, and purged by extract of jalap and senna mixture. After this, purgatives, with tartar emetic, were used almost daily; and occasionally a small dose of blue pill, the pain in side and shoulder never being entirely removed, and often becoming severe. On the 27th July, at night a sense of suffocation came on, and a copious expectoration of "phlegm;" the pulse becoming rapid, and respiration hurried; the gums were still tender, and remained so for several days; by this time the patient was much debilitated, but the pain of side moderated. On the 1st August, the Sputa are described as matter tinged with blood. There was great pain in right side of chest on the 5th August, not alleviated by V. S. to 14 ozs. Colliquative Diarrhœa took place on the 15th September, under which, and an extensive suppuration of the liver and lung, the patient sunk, and died on the 30th. On dissection, a large abscess of the right lobe of the liver was found, with an opening into the lung.

The reporters observe that the inutility of bleeding at long intervals, and of salivation as a preventive of abscess of the liver is evident in this case. There was no rigor; the communication of the hepatic abscess with the lung was sufficiently distinct.

*Case* 3. This occurred in an European soldier, and terminated in abscess; the patient's age, and period of residence in India, are not mentioned. He was a man of intractable disposition, who obstinately resisted a sufficient course of depletion by V. S. in the early stage of the disease, and was detected afterwards in using an unauthorised quantity of animal food; nevertheless, the progress of the disease was in many respects interesting, and illustrative of those points to which practitioners have to direct attention in the treatment of acute hepatic disease. The patient was 76 days in hospital, being admitted on the 14th of August, with Pyrexia, and pain in the right side, and at top of right shoulder: he was bled to 24 ozs. on that day; 30 leeches were applied on the 16th; V. S. was repeated to 20

ozs. on the 18th ; and again to 20 ozs. on the 19th: on the 20th 30 more leeches were applied. The medicines consisted of a saline purgative on admission; after which calomel, gr. v. with pulv. antimon. gr. ij. were given three times a day. On the 21st, these medicines were repeated every three hours, and severe salivation occurred on the 22d : the sore mouth was kept up afterwards for a long time. Still the symptoms indicating the progress of hepatic abscess slowly advanced, proving how little can be trusted to salivation, as a means of arresting the progress of acute liver-disease. The loss of life in this instance, seems to have been owing to the patient not permitting the repetition of blood-letting sufficiently *often, and at short intervals, at the commencement of his illness.* The frequent repetition of blisters, at a very early stage of the disease, is evidently shewn to be of no avail, in making up for the deficiency in depletion by blood-letting, during the acute stage of hepatitis; for this man was blistered on the 16th, 20th, and 22d August, without benefit. Three weeks before death, the ribs on the right side were observed to be heaved up, and symptoms of the encroachment of the tumid liver on the chest became more evident; issues were then put in the side, but without benefit; irritation and debility increased for 14 days, and were followed, when the abscess opened into the cavity of the chest, by oppressed and hurried respiration, rapid pulse, hectic fever, obscure rigors; and ultimately by diarrhœa; under which the patient sunk, without having expectorated any matter. Latterly, the treatment consisted of mineral acids, quinine, mild aperients, and anodynes.

The most constant symptoms during the early part of this man's illness, were frequent pulse, morbid heat of skin, and pain in the right side; afterwards a sense of weight in the side; subsequently attended with faintness, and pain in front of shoulder, with numbness of the arm and thigh.

On dissection, an enormous abscess of the liver was found to have made its way through the diaphragm, into the right cavity of the chest.

These cases certainly deserve to be recorded.

---

### XLI.

### Case of Bite from the Karrait Snake.

This case was communicated by Brigadier Wilson, who watched and treated it with much care and some dexterity. As the narrative is brief and very interesting we shall use the narrator's own words. The details exemplify well the action of an animal poison extremely virulent, yet not sufficiently intense to destroy a man.

"June 10th, 1829.—Reached home from the lines of the 12th Regt. at about five minutes past 10, p. m. The bearers were carrying away the empty palkee, when one of them who had taken off his shoes trod on a Karrait* snake, which bit him just above the great toe of the right foot. On the man's calling out that a snake had bitten him, I took up a candle and a stick, and went out and killed the snake; it was, I think, a full grown Karrait, though I have seen larger ones; it was about two feet three inches in length, and was much more active than those I have generally seen. As the snake was within thirty yards of the house, it did not take me three minutes to kill him. The man who was bitten, did not seem very much alarmed at first, but in less than five minutes he was very much so; he became quite insensible in less than ten minutes after he was bitten; he sunk down, was unable to move, and appeared like a man quite drunk, except that he made little noise: his pulse was rather faint and irregular, and he breathed hard. I should have said, that as soon as he was brought to the house, which was in ten minutes

* Supposed to be the Karetta, plate 26, page 32 of Russell's work on Indian serpents.—W. T. *Secretary Med. Soc.*"

after he was bitten, I gave him a tea-spoonful (about sixty drops) of Eau-de-luce, in a glass of water; he easily drank it, and I also rubbed some of the same on the bite. I continued to apply hartshorn, till about half-past 11, or quarter to 12. I had nearly emptied my Eau-de-luce bottle in the morning on another man, my surwan, and had to send to the Sudder for more, upwards of a mile off, in a dark night; and I feared the man might die for want of medicine: I therefore gave him about two tea spoons-full of hartshorn, of which I had plenty. I think that the hartshorn did as much good as the Eau-de-luce. The man had no difficulty in swallowing, and was perfectly in his senses at 11 o'clock, though he complained of great pain in his foot, and seemed very uneasy. At half-past 11 the part about the wound was very raw, all the skin having come off, (from rubbing, I suppose;) he could sit up and complained much; the leg was quite cold, and mottled black here and there, some way above the ancle: he was very sleepy; his pulse was regular, but rather slow, and weak. About midnight I had his foot put in water, as warm as he could bear it; and hot towels were put on his leg, which was well rubbed for two hours. I also gave him about 12 grains of opium, dissolved in Eau-de-luce, and put into a glass of brandy to rub to the part. The man was pretty easy at 2 o'clock, and at 3 I allowed him to go to sleep, which he seemed much to require. The thin blood continued running from the wounds, particularly those caused by the snake's two upper teeth, which seemed to have done all the mischief; they were much larger than the others. Up to 3 o'clock there was no symptom of swelling in any part of the foot or leg; which continued quite cold, and mottled with black. The man, a stout young Cahar, was much better the next morning, at 8 o'clock, when he got up; but his leg was terribly swollen up to the hip; there was pain all the way up, but not very severe; blood (very thin) ran out of his nose and foot all day, and a great many blisters arose below the knee and all over the foot: as these grew worse, and burst, they turned into bad sores. I had two poultices put on every day, and the pain and swelling came gradually down in about three weeks: the sore is now nearly well; all the blister-sores, except one above the fourth toe, are healed. I gave the man plenty of meat (flesh), and a glass of brandy and water, under the name of physic every day. Perhaps the effects of the bite would have been less sudden, had not the man's blood been in rapid circulation at the moment he was bitten by the snake. He must have been heated, as I had come upwards of a mile in my palanquin, and he was under the pole when I arrived at home. It is now upwards of a month since the above circumstance took place, and I state from recollection the steps that I took to cure the sufferer. Dr. Eckford, of the 12th Regt. was so good as to tell me how to treat the man subsequent to the bathing of his leg in hot water, at 12 o'clock on the night of the accident."

---

### XLII.

### Native Lithotomists in India.

The Celsian operation, "by the gripe," would seem to be the rudiment of a scientific and successful lithotomy. In India the native operators employ it, and, were we to credit the stories they tell us, with very great success. Our experience, however, is sufficient to prevent our being deluded by such narrations. Mr. King, of Patna, gives an interesting account of a native operator and his operation.

"At Poonerai, a village 3 coss from Patna, resides an Hujam, of the name of Chukkun; who says that he has performed the operation of lithotomy, on 35 persons, one only of whom died: in that case the stone was of a very large size, and broke into several pieces; the patient died on the 3d day after the operation. He shews 20 calculi, some of which are of considerable bulk. The instrument he uses, is something like a clumsy-made pen-knife, and he describes his mode of operating, as follows.

The patient being put in a convenient posture, and held firmly by several persons; an assistant presses the lower part of the abdomen, in the direction of the pelvis. The operator introduces his fore and middle fingers into the rectum, and crooking them so as to obtain a hold of the stone, he brings it towards the perinæum, till it becomes prominent there. He then makes an incision over the stone, (on one side of the raphé, and about an inch from the anus,) and extracts it. The urine passes through the wound for some days; and the cure is generally completed in from twelve to twenty days. Chukkun acquired his art from his father, and has now taught his son to perform the operation. As this man keeps no register of the cases, his statement may be liable to errors, respecting the numbers of persons on whom he has operated; and probably a liberal deduction should be made from his account of success, which it is of course his interest to magnify. In fact, it is almost an invariable custom with native operators, to assert that their operations are attended with certain success; and that cures in their hands are infallible. At the same time we must acknowledge, that they evince much cunning and dexterity in assigning reasons for not operating on unfavourable cases. They operate with a view to *cure*, and would not hazard their reputation for the prospect of alleviating continued suffering, when they did not expect ultimate recovery."

---

### XLIII.

### ST. GEORGE'S HOSPITAL.

We purpose in each succeeding number of this Journal to give a lengthened, and, we trust an interesting report of surgical and medical cases treated in this institution. On the utility of Hospital Reports we need say nothing, as the general demand for them proves that the profession attach to their acquisition some value, real or imaginary. It is true that there are inconveniences attendant on the practice of relating cases from public hospitals, and it is also true that, with all the publicity which those cases possess, there is yet much room for unintentional error, and intentional misrepresentation. Yet still the advantages predominate, and more especially this one, that the profession becomes acquainted with the results of treatment on a large scale, where accidental circumstances are less likely than in insulated and single experiments to interfere with the results. When particular remedies are in vogue it is scarcely possible to ascertain their actual merits in private practice, at least it requires the experience of a certain length of time to do so. But in hospitals diseases are usually presented in the mass, and accordingly we frequently find that true notions of the value of particular plans of treatment, and even an acquaintance with the phenomena of particular diseases, are entertained in hospitals long before they become generally diffused among medical men. The object of the hospital reporter should be to facilitate and expedite this diffusion of information.

We fear that clinical instruction will never be carried to the same perfection in this country, which it has attained upon the Continent. For this there are reasons on which we need not touch. But the records of our public institutions might, with little difficulty, be made more extensively and permanently useful than they are. In few hospitals, are there any lasting and easily available histories of the many valuable facts which are witnessed within their walls. Some may pass to the note book of the surgeon and physician, but they then are private property, and their utility is narrowed, perhaps destroyed. A more public, more systematic, more comprehensive, and more durable record should be established.

But we must take things as they are, and under existing circumstances we know of no better means of conveying abroad the knowledge which circulates, and is apt to be confined in a hospital, than by publishing a fair and, as far as possible, an accurate account of the most important cases which occur in it. Such cases may be reported in two ways, in groups, or as individuals. Both methods are serviceable and we shall adopt which appears most convenient at the time. In the present instance, we shall give the details of some cases which appear to deserve commemoration, without reference to any particuler subject.

1. OPERATION OF LITHOTOMY FOR THE EXTRACTION OF A PORTION OF CATHETER FROM THE BLADDER.

A STOUT athletic looking man who had lost one leg at Waterloo was brought into the operating theatre, in order to have a portion of gum catheter extracted from his bladder, by the ordinary lateral operation of lithotomy.

It appeared from the man's statement, that many years ago he had been in the Hospital under Sir Everard Home, with stricture of the urethra. For eight years after leaving the hospital he had continued tolerably well, but subsequently to that period he had been subject to occasional attacks of retention of urine, for which he was in the habit of introducing for himself a gum catheter of moderate dimensions. The catheter last in use was so antique that he had been obliged to cut off the rounded extremity, in consequence of flaws and cracks at the eye. On the 1st of the present month he had the misfortune to let the instrument slip entirely into the urethra. After some ineffectual attempts to remove it by his own efforts, he went to a medical gentleman in the Vauxhall-road, who seized the catheter with a pair of forceps but could not withdraw it; it broke in the urethra, a large portion remaining in the bladder and vesical end of the canal. The patient describes the piece of catheter as forming a sort of projection in the perineum, and no doubt it might have been cut down on in that situation and extracted, if it could not have been removed by other means. The patient went his way and sought no further advice till the 3rd inst., when he repaired to another surgeon at Camberwell, who seems to have handled him rather roughly. This gentleman, whose incredulity appears to have been fully equal to that of the Lazarites, told the man he was a "bloody rogue," and summoned the new police. This sort of gend'armerie surgery did not satisfy the patient, who went to another doctor, and another, and ended at last by coming to the hospital where he had formerly received benefit. This was on the 4th inst., but the operation was not performed until the 8th. In the interim he suffered much. He had constant desire to make water; passed little at a time and that with pain; had violent pain in the right lumbar region, but not in the glans penis; had alkaline urine with deposition of mucus, and as we believe, the phosphates; and although some narcotics were administered he spent but a miserable time of it.

On the 8th, as we have stated, the operation was performed by Mr. Brodie. It was the usual lateral operation, but the incisions were rather smaller than are made for the extraction of a calculus. Mr. Brodie employed his beaked knife, and the prostate was divided, the forceps introduced, and the gum-catheter extracted with Mr. Brodie's general precision, facility, and dispatch. The catheter was moderately curved, five or six inches in length, cracked in several places, and incrusted with a deposition of the phosphate of lime. The patient bore the operation without a murmur, and on the 15th, when we saw him, the wound was nearly healed, and the urine almost natural.

Mr. Brodie made some valuable remarks upon the case to the gentlemen assembled to witness the operation. He said that he had never known a similar accident occur before,* although he had seen several instances of other foreign bodies slipping into the bladder and forming the nucleus of stone. One of these cases was rather curious. A gardener labouring under stricture conceived the ingenious but unsafe idea, of employing the bougies which Nature

* Much such a case occurred in Paris two or three years ago. The patient applied at La Charité, and M. Roux performed the lateral operation with the bistoire caché, for the extraction of the catheter. The patient died. The case was related in this Journal.

placed in his way. When labouring under more than ordinary difficulty in making water, he introduced a flower-stalk into the urethra and dilated the passage. But flower-stalks are brittle, and unfortunately one broke at last in the urethra, and a portion slipping into the bladder formed the nucleus of a stone which was afterwards removed by operation. Mr. Brodie pointed out the mortar-like incrustation on the catheter which he had just extracted. Now I know, said he, without resorting to any chemical analysis that this incrustation is the phosphate of lime, because in such cases it always is so. The foreign body in the bladder becomes a source of irritation and inflammation—the inflamed mucous membrane of the bladder secretes mucus—and from that mucus is deposited the phosphate of lime. It is true that if the case went on other depositions would occur. The irritation, which had its primary seat in the bladder, would be propagated to the kidney, to the system. The *secretion of urine* would then become depraved, the triple phosphate would be formed in the kidney, and this, mingling with the phosphate of lime in the bladder, would form the fusible calculus.

Mr. Brodie explained why he had not attempted to remove the catheter by Sir Astley Cooper's urethra forceps, or by a pair of his own, which he shewed, and which exhibits several improvements on the former. He considered that the foreign body had been in the bladder for some time, that it was described as being of considerable size, that he might have failed in its extraction and would have irritated further a part much irritated already. These and other considerations determined Mr. Brodie to cut into the bladder without any previous attempts with forceps, and the event has confirmed his judgment.

### II. Diffused Hydrocele of the Cord.

We have lately seen two specimens of hydrocele of the cord, which can hardly be considered examples of that which is usually called encysted. The instances of encysted hydrocele of the cord, which we have seen, have been small, more or less globular in form, and circumscribed. But the swelling in the cases we are about to describe had not these characters.

We were struck with the appearance of the scrotum of a physician's patient in the dead-house of the hospital. There was a swelling, which, at first sight, looked like that of common hydrocele. It was large, and extended from near the ring to near the bottom of the scrotum—pyriform with the fundus below—immoveable, fluctuating, and *very* transparent. The testis was at its bottom, and rather to one side, but was firmly attached to the swelling. On dissecting the tumor, the cord was found spread out, its veins, in particular, being scattered irregularly over the sides of the swelling, The cyst was very thin, and occupied the cord from just below the ring to the testis; it appeared to be rather formed from the interstitial cellular tissue of the cord, than from that general cellular envelope or fascia which surrounds it. It was firmly attached below to the epidydimis, the direction of which was nearly horizontal, instead of obliquely from above downwards. The testis was below instead of in front, and its malposition gave it a dislocated appearance; it was healthy, and the cavity of the tunica vaginalis, though large, was free from fluid. The fluid of the cyst was very pellucid.

This may be considered as merely a large encysted hydrocele of the cord. However that may be, it is well for practitioners to know that the ordinary descriptions of encysted hydrocele will not apply to it, and that its characters so nearly resemble those of common hydrocele, as to make it likely to be mistaken for it. The points in which it differs from common hydrocele are, the position of the testis below rather than behind, and its very pellucid appearance between the eye and the light. Such a cyst, it must be recollected, is not strictly a serous membrane, nor so prone to inflammation and obliteration of its cavity by adhesions. It is well known that injections will not cure encysted hydrocele of the cord as they will hydrocele of the vaginal tunic, and it is probably from the operation of this law, that they will not do so. On looking at one of these cysts, we usually observe that it is extremely thin, and possesses but little vascularity; its growth is slow, its inflammation weak. In the encysted hydrocele of the cord, a seton will sometimes effect a cure—incision, and the introduction of lint into the cyst, is more certain. The treatment, which, when applied to the fibro-serous tunica vaginalis, excites very violent inflammation, causes barely sufficient in the pellucid cysts of the cord. It is on this account that we have drawn attention to this form of hydrocele, that practitioners may not deceive themselves and disappoint their patients, from imagining that they have a common hydrocele to deal with, when one of the kind we have been describing presents itself. Surgeons in large practice are probably well acquainted with the subject; but all surgeons are not in large practice, and things familiar to a Cooper or a Brodie are far from familiar to them.

### III. Fatal Case of Constipation.

John Clements, æt. 35, admitted Nov. 23, 1831, under the care of Dr. Seymour.

Ten days previously he had been attacked with severe colicky pain in the right colic region, for which he was leeched, and we believe, bled. A day or two after that he had a motion, since which he had had none. He stated that previously to the attack of pain he had passed motions of the natural calibre, and that his bowels had not been unusually constipated.

On admission, the belly was enormously distended, hard, tympanitic, tender to the touch, particularly in the umbilical and cæcal regions. He felt great inconvenience and uneasiness—passed no flatus per anum—had stercoraceous vomiting. Features pinched—tongue thickly coated—pulse 110, rather feeble. The treatment adopted may be set down in the following series: bleeding, calomel and warm water injections—croton oil and enemata of castor oil—one oz. of quicksilver, afterwards repeated with enemata—assafœtida with turpentine enemata, wine, arrow-root, and beef tea—injections and a blister to the belly. All this was accomplished by dint of indefatigable exertions on the parts of physician, apothecary, and house-surgeon, in the short space of time that intervened between the 23d, when the patient entered the hospital, and the 27th when he died. At one time, under the assafœtida and turpentine administration, the halo of nasty effluvia round the bed, from the combined effects of these not inodorous substances and the stercoraceous vomit, was such as can be more easily imagined than described.

On examining the body the convolutions of the small intestines were found almost inextricably intertwined and bound together by coagulable lymph. The stomach was contracted, the small intestines distended with air, the cæcum of great size, the transverse and descending arches of the colon contracted and empty.

The cæcum, we have said, was of great size, dark coloured, thin, bursting under the slight touches of the house-surgeon who examined it, generally adherent to the abdominal parietes. Its mucous membrane was in part destroyed, and there was much extravasation of blood between its coats. The ileo-cæcal valve was large and tumid. The cæcum was filled with apparently a mixture of fluid fæces and the injections which had been thrown up. In the small intestines were some solid fæces. The peritoneal inflammation seemed to be chiefly of some days' standing. If a stricture existed in the twisted small intestines it was not made out. It was reported that the patient had previously had an attack of peritoneal inflammation.

Sundry conjectures were hazarded on the actual cause of the constipation. The facts are more valuable than were the theories.

### IV. Fatal Results from Amputation of Fingers.

*Case* 1. A man aged 44, got the ring finger of his left hand entangled in an iron chain employed in raising a very heavy weight; it was torn off at its second phalanx near its joint with the third. He applied at the hospital, and the house-surgeon found it necessary to amputate the ragged end of finger, which was done about the centre of the second phalanx. Nothing particular occurred until the sixth day after the accident, when he was attacked with rigors and pain in the hand. On the next day he was admitted into the hospital under Mr. Brodie.

The hand, especially its dorsum, was a good deal swollen, with diffuse redness and pain—some redness extending in streaks up the fore-arm, with enlarged glands in the axilla. There was pyrexia and rather an anxious countenance.

Numerous leeches were applied to the fore-arm, and calomel purgatives with fomentations, and antiphlogistic measures resorted to. On the next day the swelling was greater, and the pyrexia continued. On the 8th day, more pain—much swelling of the palm and dorsum of the hand—diffuse redness, tension, and tenderness of the latter. Leeches were again applied, and repeated on the following day. On the 10th day the hand was much the same, saving that the redness had in some degree subsided—pyrexia, with irritability, and some depression. This night he had a rigor followed by a slight hot stage, but much sweating. He looked anxious, low, and ill. He complained now of stiffness in the *right* arm, on the outside of which was tenderness, fulness, and a slight circumscribed erythematous blush; in short, a purulent depôt was here forming in the cellular membrane. There was a similar appearance commencing on the outside of the right fore-arm. Mr. Brodie determined to try the effects of the oxymuriate of mercury, which he ordered in doses of one-eighth of a grain three times daily.

On the 11th day he said he was much better, which patients labouring under the depôts most commonly do; his pinched and anxious countenance gave his tongue the lie. The belly was now tympanitic, the symptoms merging into symptomatic typhus; there was also slight oppression at the chest.

After this rigors took place at intervals—there was generally a disposition to perspiration—he had rambling delirium, yet if asked how he felt himself would say he was well—he passed his stools under him. On the 12th day a depôt was observed in the right thigh. On the 13th, diarrhœa having set in the oxymuriate was discontinued; it had obviously been productive of no benefit. A puncture was made in the hand first affected, and a good deal of pus let out. A puncture was also made in the depôt in the thigh, and pus discharged. On the 16th day, the diarrhœa being checked, the oxymuriate was resumed. He now became rapidly lower, the breathing grew frequent and oppressed, and he sank early in the following morning. For the last two or three days there had been no rigors.

There was no affection of the thoracic or abdominal viscera. The cranium was not examined, neither were the limbs, in consequence of the great repugnance of the friends.

Case 2. — *Amputation of Finger — Erysipelas—Death.*

William Green, æt. 19, applied at the hospital as an out-patient, at the latter end of December, 1830, with disease in the joint between the 2d and 3d phalanges of the right fore-finger. The cartilages were ulcerated, there was great thickening of the soft parts, and several sinuses led down to the diseased joint. The disease had been the result of a slight accident. He went in the first instance to the Middlesex Hospital, but left it to apply to this.

Splints, &c. were employed, but the finger not becoming much better, and the lad being extremely desirous of its removal, it was amputated on January 15th, nearly midway between the metacarpo-phalangal and first phalangal joint. On the following morning the lad complained of feeling ill, and was rather feverish; he was therefore admitted into the house.

In the afternoon he had a rigor, and in the evening pyrexia; there was some fulness about the stump. *Ordered some calomel and James's powder, house physic, and salines.* On the 17th the pulse was more frequent, with headache; there were pain and starting in the stump, and a little diffuse redness about the dorsum and palm of the hand. On the 19th he was extremely irritable, and vomited several times. Some diffuse inflammation of the skin and cellular membrane, and also of the absorbents, appeared on the back of the hand. On the 20th the absorbents were inflamed as high as the axilla, where an enlarged and painful gland could be felt. The nitrate of silver was applied round the arm, above the inflamed absorbents and gland; the salines were continued. On the 21st there was little alteration. The nitrate of silver was applied over the inflamed surfaces on the hand and outside of the elbow. In the night he became quite delirious and unmanageable. On the 22nd, the face had a dull flush on it—skin dry—pulse 130, feeble—tongue red at the tip, white on the dorsum—bowels not open. He was extremely irritable and rather incoherent. Erysipelas of dull colour and without much vesication had appeared round the parts to which the nitrate of silver had been applied, this having produced vesication. The streaks produced by inflamed absorbents of the arm had nearly disappeared, but the axillary gland was still painful. The inflammation had never passed beyond the upper boundary of nitrate of silver. In the evening he had a furious paroxysm of maniacal delirium—the erysipelatous redness of the hand and elbow had spread, with deep dull colour and indistinct margin. He obtained no sleep till 5 A. M. of the 23d. On that day he was very low, with pulse 140, dry tongue, sordes about the teeth. The urine was drawn off; there was scarcely any in the bladder. He lay in a stupid state, scarcely answering any question. He was ordered some bark and ammonia, and a saline injection was administered.

24th. Passed a pretty quiet night. The stupor has now all the characters of that produced by effusion on the brain. He takes little notice of what is going on around him, but every now and then he suddenly utters some unconnected and irrelevant cries. Pupils dilated and sluggish—pulse 130, feeble—teeth incrusted with sordes—tongue dry, reddish brown—skin cool. The erysipelas has, for the last two days, been spreading on the fore-arm and hand, but little so upon the arm; it is of the same characters as before.

*Emp. canth. nuchæ. Radatur caput et infr. capito raso ung. hyd. necnon etiam cruribus. P. c. h. cinchonæ.*

On the 25th he was a shade better, calling on the house-surgeon by name, and appearing in all respects more sensible. Erysipelas had left the hand, and was spreading up the arm. Next day the gums appeared somewhat mercurialized. He continued a little better—pulse 110—no heat of skin. Camphor liniment was applied to the head instead of the mercurial ointment. On the 27th there was little alteration. The erysipelas had left the fore-arm and was creeping up over the shoulder, with little tumefaction, a tolerably good tint, and pretty distinct margin. The gums were decidedly affected—small pustules had appeared about the head. He was ordered a little porter. On the 28th there was little alteration. The blister on the neck, which had previously been dressed with mercurial, was now treated with savin ointment. *Ordered some infusion of cinchona and cusparia.*

On the 30th, the erysipelas had left the arm, but was spreading a little on the back—he would not take his food or medicine so well as before.

*Emp. canth. capiti raso. Hyd. sub.* gr. ij. *Ext. Lactucæ*, gr. v. 8*vis. horis. Adde haustui Amm. carb.* gr. v.

FEB. 2. There has been little alteration in the symptoms since the last report, save that the patient has been gradually sinking. There has been no further return of intelligence, and he has scarcely spoken for the last two or three days; now and then, when disturbed, he will mutter a few unintelligible exclamations. The erysipelas has extended a little on the back, but it is trifling both in character and in extent. Emaciation has taken place to a remarkable degree. On the following morning he died.

Sectio Cadaveris. *Cranium.*—No traces of inflammation of the membranes or substance of the brain. No serous effusion between the membranes. Ventricles large, filled with clear watery fluid; there was about half an ounce in each.

*Thorax.* Old adhesions on both sides of the chest. In the upper lobe of the left lung some common tubercles, in stages of maturation and softening. Some had formed small vomicæ about the size of nuts. The other lobe of the left lung, and the whole of the right were free from tuberculous formations.

*Abdomen.* Partial congestions and vascular injection of the mucous membrane of the lower part of the ileum. Here and there, small ulcerations in the mucous membrane, apparently not follicular, as in fever; there was no attempt at reparation.

We should mention that the stump had healed early, and without trouble.

Another instance of death from amputation of a finger, occurred in the practice of Mr. Babington. We have not preserved any notes of the case, but the following is an abstract of the particulars.

The patient, an old woman, was admitted with much disease of the thumb of the left hand. It was considerably enlarged, the integuments discoloured, and several sinuses led down to denuded bone in the phalangal articulation. Mr Babington removed the thumb below this joint. In a day or two inflammation attacked the cellular membrane and skin of the hand, and spread in an erysipelatous form up the fore-arm and arm. The swelling was not very considerable, the tint of the skin was a dull red, the margin tolerably distinct. Vesications occurred, but the cutis rapidly became mottled from extravasation into its texture, and then gradually died, exposing lymph and serum in the subcutaneous cellular tissue. The affection was by no means of that rapidly destructive character which traumatic gangrene assumes; it was in fact a mixture of erysipelas and diffuse cellular inflammation, occurring in a very debilitated and aged person. At the end of seven or eight days she died, her constitutional powers being evidently incapable of sustaining, or even originating the reparative process.

A case of a similar kind, though the patient ultimately recovered, occurred to Mr. Brodie. A finger was removed for disease of the first phalangal articulation. Erysipelas succeeded and passed up the fore-arm to the arm, where it stopped. Some incisions were necessary in the hand, and convalescence was slowly established.

### V. Cases of Laceration of the Urethra.

Case 1. *Fractured Pelvis—lacerated Urethra.*

George Plummer, a carter, æt. 49, admitted June 18, 1830, under the care of Mr. Keate.

Whilst conducting his horses and cart at 9 a. m. this morning, he had occasion to jump off the shaft, and was severely crushed between the wheel and a post. He sank down and has since been incapable of using his lower extremities. The accident happened at no great distance from the hospital, and at 11 a. m. he was admitted.

There was evidently fracture of the pelvis in more than one place. A projecting edge of bone could be felt on the left ilium, about three inches behind its anterior superior spinous process. There was little ecchymosis about the pelvis, a good deal on the outside of the left thigh. Some blood had issued from the orifice of the urethra; he had made water about two or three hours previous to the accident.

*Laid on back with pillows under knees.*

At half-past one p. m. the catheter was introduced—an obstruction occurred about the membranous part, but was surmounted. The urine was bloody, but the greater quantity of blood issued at first.

*Patient laid on Earle's bedstead, with a broad belt round the hips.*

*Vesp.* Troublesome cough—some heat of skin. Urine drawn off, dark and bloody, without coagula.

*H. salin. c. Tinct. hyos.* ♏xx. *Sp. æth. nit.* ʒss. *Oxymel. scillæ,* ʒj. *Vin. ipec.* ♏xx. 6*tis hor.*

19th. Urine thick and muddy-looking—pain in the pelvis on coughing. Pulse frequent, rather wiry—aspect anxious—no motion from the bowels.

Next day there was more pyrexia, and he complained of feeling "pain all over him"—the cough was troublesome, and expectoration difficult—the bowels open. The urine was now pretty clear. Gum catheter had been retained in bladder.

*Linctus in tussim. H. sal. c. Vin. ant. t.* ♏xx. *Tr. camph. c.* ʒss. 6*tis hor. Omr. alia.*

On the 21st he was better, and on the 22d the catheter was removed. The urine continued clear, but was high-coloured. At 1, a.m. of the 23d he had a rigor, succeeded by heat, which soon passed away. Was this rigor occasioned by his making water without the catheter? He had passed urine at 9, p.m. the preceding evening. At 11, p.m. he had another rigor, without apparent cause. There were neither local nor general symptoms of any consequence.

No further report is necessary. Finding some little difficulty in making water, he introduced the gum catheter for himself occasionally. On the 20th July he was removed from Earle's bedstead, and, on the 10th August, began to walk about with crutches. At the end of the month he was discharged cured, and said that his bones felt stronger than before. We saw the man lately, and he continued well; he had very slight lameness.

CASE 2.—*Injury of the Pelvis—Lacerated Urethra.*

George Frost, æt. 11, admitted, Oct. 11th, 1830, under the care of Mr. Hawkins. At 8 o'clock this morning the lad was run over, at Putney, by a horse and cart heavily laden. The wheels appeared to pass over the pelvis, and there is reason to think that he was lying on his side or belly. Soon afterwards some blood issued from the orifice of the urethra.

He was admitted at 5, p.m. and, in the interval, he had either made no water at all, or very little. No fracture of the pelvis could be distinguished—there were contusions and effusion of blood, chiefly above and about the right natis, with some over the left hip. He complained of pain on moving the pelvis, and said he could not make water.

The catheter could not be introduced, the instrument appearing to hitch in some laceration on the left side of the urethra, whence bleeding readily occurred.

*Tinct opii,* ♏xx. *Fotus abdomini. Knees and feet bound together—double-inclined plane.*

On the following morning the house-surgeon passed the catheter, and drew off some bloody urine. At 1, p.m. it was again found impossible to introduce it. On the 27th, the catheter was re-introduced, with less difficulty. The urine was less bloody, but porter-coloured, and apparently mixed with much lithate of ammonia. The constitutional symptoms were those of febrile excitement, and a good deal of irritability. It has been mentioned that the boy was unable to make water by his own efforts. The treatment consisted in perfect repose, starvation, and occasional purgatives.

30th. Has continued much in the same state. Can rarely make water without the assistance of the catheter, and then it issues but sparingly, and on pressure above the pubes; it is mixed with the lithate of ammonia and some blood. No fever. *Ordered beef-tea.*

Next day he was placed on ordinary diet. On Nov. 2d, we find that he had made water without the assistance of the instrument for the preceding 24 hours. He was allowed to get up. On Nov. 6th there were no unpleasant symptoms, and he could make water well enough, without any appearance of blood in it. Dismissed.

In the two preceding cases, the exact amount and situation of the injury are, of course, problematical. It is not unlikely, from the nature of the injury, the bloody urine, and the difficulty experienced in both cases in the introduction of the catheter, that there was some laceration of the urethra. At the same time, it is reasonable to imagine that the laceration was not very extensive, because the urine soon ceased to be bloody, and no symptoms of its effusion into the cellular membrane ensued. Perhaps, in such cases, the mucous membrane, or even the spongy part of the urethra, may be lacerated, without any injury of the external fibrous tunic. This is conjectural; the facts themselves are practically interesting.

Case 3.—*Ruptured Urethra—Purulent Depôts.*

We made the following memoranda of this case. It was treated last year in the hospital.

A middle-aged, healthy, labouring man fell astride a chair, and struck his perinæum. He was admitted into the hospital soon afterwards. He had made water with great pain, and there was much ecchymosis about the perinæum and scrotum. An attempt was made, without success, to pass a large silver catheter; it seemed to pass between the bladder and the rectum, and blood only flowed. A smaller gum catheter was introduced on the first trial; it was left in the bladder. The catheter slipped out now and then, and some difficulties occurred.

In a few days the man had a rigor, then another, and another. An incision was made in the perinæum, and a large opening into the urethra was found, with a sloughy state of the cellular membrane. He seemed to be relieved, but soon became very low; he was ordered a little support. Rigors again came on; abscesses formed about the right groin and upper part of the thigh—then swelling and acute pain in the calf of the left leg. He died with the usual symptoms of the purulent depôts. He had complained, before these symptoms commenced, of pain in the side of the chest and in the epigastrium; it seemed to be sympathetic.

On dissection, a large sloughy depôt was found between the deep and superficial layer of muscles in the calf of the left leg; some smaller deposites were also found in the cellular membrane elsewhere. There was much destruction of the lower wall of the urethra, opposite the incision in the perinæum, and also in another part, about an inch nearer to the bladder. The cellular membrane in the neighbourhood was dark and sloughy.

Several years ago, Mr. Brodie had a patient in the hospital with ruptured urethra. The accident occurred in a manner not very dissimilar to that in the last case. This patient died with the purulent depôts, but we do not exactly remember the particulars, and we do not possess any notes of the case. If our memory does not deceive us, there was a deposite of pus in the corpus spongiosum urethræ.

An instance of ruptured urethra, in a boy, occurred lately under Mr. Babington. It was necessary to make an incision in the perinæum, and the urine subsequently passed through the opening. When we saw the lad last, the perinæal wound was closing, and the discharge of urine by the natural channel was becoming daily more established.

### VI. Fractured Pelvis.

In a former number of this Journal we reported two interesting cases of fractured pelvis, in one of which the bladder was ruptured, in the other the superior wall of the membranous part of the urethra, internal to the ligament of Camper. Both patients died with the symptoms and consequences of effusion of urine into the pelvic cellular membrane. The following case shews that fracture of the pelvis, unaccompanied with lesion of important organs or parts, is not necessarily fatal, even under unfavourable circumstances as to age and constitution.

*Case.* Mary Wilson, æt. 61, admitted Nov. 19th, 1830, under Mr. Hawkins.

She was received late in the evening of the 19th, having fallen from a ladder 12 feet high, immediately prior to her admission.

She was in a state of insensibility—arterial blood escaped from the right ear and from the nose—much ecchymosis over the os innominatum—right lower extremity nearly motionless, and toes everted—crepitus distinct in the right os innominatum on flexion or rotation of the limb—on applying the hand to the bone itself a sensation of the existence of several fragments communicated to the fingers.

In the night she was so low as to require stimulants, but next morning some reaction had occurred. She was partially insensible, and made irrelevant answers to questions. The pupils were contracted and sluggish—the urine was not bloody.

*Feet and knees bound together, and feet secured to the bed—pillows under the knees and shoulders—stimulants omitted—beef-tea and arrow-root.*

21st. Face slightly flushed—pulse fuller. Regained her sensibility last night, and answers questions rationally—tongue dry, rough, furred.

*H. salin. ex. Ammon. carb. 6tis hor.*

22d. Bowels confined—tongue not so dry—lies in a half-dozing state. *Ol. ric.*

On the 24th she was ordered some porter. On the 27th, the report states that there had been little alteration for the last few days. She was inclined to that low delirium which is frequently seen in old people after injuries. She was sick several times on that morning.

*Mist. camph.* ℥iss. *Ammon. carb.* gr. v. *Sp. æth. nit.* ʒj. *6tis hor.*

28th. During the last day or two there has been some cutaneous and cellular inflammation near the right olecranon, apparently the consequence of injury received in her fall. An incision was made yesterday, and some sloughy cellular membrane exposed. To-day there is a good deal of induration of the cellular membrane about the upper part of the forearm and bend of the arm, with dull, diffused redness of the skin. Pulse rather lower.

*Incision made—a poultice.*

On the 29th there was rather an extension of the redness down the forearm. On the 30th it was diminished, and there was less feverishness. On the latter day she was placed on ordinary diet. On Dec. 3d the incisions were enlarged, and matter which did not escape with freedom previously was discharged; after this she was relieved. On the 9th, cellular inflammation having re-appeared, incisions were made over the back of the elbow. There was still much ecchymosis about the pelvis, but no crepitus could now be felt.

*Vin. rub.* Oss. *quotid.*

We need not pursue the details farther. Sufficient to say that, under proper dressings and bandaging, the arm gradually got well, and the health improved in the same gradual but steady manner. On the 11th Jan. she could sit up very well, and soon afterwards she was wheeled about the ward in a chair. She was discharged at the latter end of the month, being then able to assist herself a little with crutches. We heard many months afterwards that she had continued to gain strength, but was not able to walk unsupported by crutches.

We may make an observation, which is applicable not only to this case but to many others. Placing pillows under the hams, and gently binding the knees and feet together, insures a considerable degree of quiet on the part of the patient. But frequently this position becomes so irksome that patients, especially the old and the young, cannot be persuaded to maintain it. Under such circumstances the best plan is either to put them on Earle's bedstead, or allow them to lie in a common bed in what position is most easy for them. It was necessary to do this in the last case.

### VII. FRACTURED STERNUM FROM FALLING ON THE HEAD.

In the following case the sternum was fractured in rather an extraordinary manner. The reader will believe, or not, the correctness of the patient's statement.

*Case.* A stout and healthy labourer from the country, æt. 22, applied at the hospital May 26th, 1831. He walked into the out-patient's room with his head bent forwards, and was incapable of either flexing, extending, or rotating it further. He had all the appearance of having partially dislocated one of the cervical vertebræ, but no displacement could be discovered. On further examination there was found about three inches from the top of the sternum anteriorly a transverse projection, but the swelling was too considerable to allow of an accurate knowledge of its nature. The man stated that he had just fallen from the top of a hay-cart, and that he had pitched upon his head. He denied having struck the breast in any manner.

He was sent to bed, and in the evening ten leeches were applied over the sternum. There was no cough, nor indeed any other symptom of visceral injury. Some pyrexia succeeded, and salines were requisite. On the 30th, medicine was discontinued, and he was placed on ordinary diet.

June 1. All swelling has disappeared. The case is clearly one of fractured sternum, the upper portion being depressed behind the lower, and the superior margin of the latter causes the transvere projection already mentioned. The fracture is situate about opposite the cartilage of the third rib. On the head being thrown far backwards, the bones slip into apposition with an audible snap, but when the unnatural position of the head is discontinued, the displacement recurs with a snap also. It is no doubt through the agency of the sterno-cleido mastoid muscles, that the upper portion is drawn up on throwing the head back.

The means adopted were these:—keeping the chin as high and the head as far backwards as possible—a figure of 8 bandage to brace back the shoulders—a pad over the projecting portion of bone, and a circular roller on the chest to make pressure upon it, and prevent motion.

These measures did not remove the displacement, but they certainly diminished it. On the 17th the bandages were removed. There was now little riding of the bone, and no inconvenience produced by it. A large abscess was discovered in the right axilla, unattended with any constitutional disturbance. It was opened, the cavity gradually filled up, and the patient was dismissed cured.

There can be little doubt that this abscess was owing to the pressure of the figure of 8 bandage. We have seen the same result on two other occasions, and, what is singular, in no instance was much pain complained of prior to the discovery of the collection of matter. Another cause of suppuration, especially in the neighbourhood of fractures, is much or coarse examination. We think we have seen some very bad consequences from over-manipulation, simple fractures converted into compound, and compound fractures extremely aggravated. It is a question also whether leeches applied to fractured limbs, in which there is much swelling, do not give a tendency to diffuse inflammation and suppuration in the subcutaneous cellular membrane. We have certainly seen this commence around the leech-bites, but whether the circumstance is a post hoc or a propter hoc we cannot pretend to affirm.

In some cases of fractured scapula it was found necessary to adopt very different plans. In one, for instance, the hand of the injured side was placed on and secured to the opposite shoulder, in another this position was reversed, and the elbow was carried backward. We should say that it is impossible to lay down any other rule than this, in the management of fractures of the scapula below its spine—to see what position of the arm brings the broken portions best into apposition, and, having found that position, to maintain it.

In our next report we hope to give a more complete collection of cases on particular subjects in surgery.

---

### XLIV

### Nitric Acid for Toothache.

We have received the following note in answer to Dr. Ryan. Whilst we agree to its insertion we cannot but regret that a professional question should breed any acrimonious feeling between two honourable men. We trust that the discussion will not be resumed, and that each gentleman will be satisfied that his character, both medical and private, stands well in the estimation of his brethren. We thus act as peace-maker, because we are truly sorry to observe any bickering or recrimination between persons whom we respect.

*To the Editor of the Medico-Chirurgical Review.*

Sir—I cannot allow the forthcoming number of your journal to go to press without acknowledging the *condescension* of Dr. Ryan, in informing you, and through you the medical public at large, by whom your excellent periodical is so generally perused, that *he* places *credence* in *my* statement respecting the use of nitric acid in some species of tooth ache—"'Tis an honor I dream'd not of" and "I must be more or less than mortal," as the noviciate says when returning thanks in his maiden speech, for having been toasted at a vestry or election dinner, "if I did not feel as I ought to do on the *important* occasion."

However singular it may appear to the Doctor, that I did not earlier publish the benefits resulting from the use of the acid, yet it is no less true than strange that I did for a period of *twelve* years (viz. from 1820 till 1832) and not *eight* as he erroneously calculates, apply it, and that too with pretty general success. From my brother practitioners in the neighbourhood I did not withhold the information, and from publishing it in some of the periodicals, I again assert I was deterred only till the "test of experience" should bear me out in doing so. Perhaps the Doctor will *deign* to admit the veracity of *this* part of my statement also, and then my design will be accomplished, and my claim to the priority of discovery based upon firmer foundations than even that of Mr. Myers, with whom alone I have to combat—I say with whom alone I have to combat, as the Doctor in my opinion has no pretensions whatever to the discovery (although he says my claims are no better than his own), for in the first place, Mr. Myers informed him of the particulars, and he now again confesses "several of his friends assure him they have known the acid applied to carious teeth many years ago." It is not from him then I wish to wrest the claim, but from Mr. Myers; and whether he or I, or any other person was the first to hit upon the remedy, it is sufficient that it is now before the public.

Trusting you will find a spare corner for this *billet*, and that the Doctor will join me when I say palmam qui meruit ferat,

I remain Sir,
Your obedient Servant,
W. THOMAS, *Surgeon.*

*Pembroke Dock.*
15*th August,* 1832.

---

### XLV.

### SEVERE INJURY OF THE HEAD—DISCHARGE OF BRAIN, AND, SUBSEQUENTLY, PUS FROM THE EAR.

THE following case has been transmitted to us by Mr. Elliot, a very intelligent surgeon of Lancashire. It is so interesting in its details, and was treated so judiciously, and successfully, that we feel great pleasure in giving it publicity in our pages.

*Case.*—At half-past 2 o'clock, p.m. the 28th of June, 1831, Edward Broadbent, a stout labourer, fell from a scaffold 30 feet in height to the ground, and pitched upon his head; he was removed senseless to the next house. At 3, p.m. the symptoms were these, viz. cold extremities—face pale—pulse scarcely perceptible—pupils dilated, and insensible to the impression of light—breathing tranquil—scalp apparently uninjured—rolling about the bed groaning, and putting his hands to his head—the knees drawn up towards the breast—a total suspension of the intellectual and mental powers—a discharge of brain from the ear, an ounce of which escaped during the afternoon—no vomiting. Ordered, bottles filled with warm water to the extremities, and two table-spoonsful of wine immediately.

℞. *Hydrarg. submur.* gr. x. *Pulv. jalap.* ℈j. *M. statim sumend.*

8, p.m. Reaction has taken place: pulse 115 and strong—heat restored—

furious delirium, three men having to hold him in bed—bowels unmoved. Venesectio ad ℥xxx. To have the whole head shaved, and cold evaporating lotions constantly applied; 20 leeches to the temples, and a purgative enema.

℞. *Infus. sennæ*, ℥vj. *Magnes. sulphat.* ℥j. *M. capt. cochl.* ij. *ampla tertia quaque horâ.*

June 29th, 8, a.m. Has been very noisy during the night—talks incoherently—bowels have not been moved, as the medicine could not be got into him: the delirium comes on by paroxysms, in the intervals he is comatose. Pulse 116 and full—great heat of the scalp—pupils still dilated—tongue furred, dry, and brown: the discharge of brain from the ear has ceased.

*Habeat Ol. croton. tiglii*, gtt. ij. *to be dropped into his mouth while sleeping, and repeated pro re nata. Venesectio ad* ℥xvj. *Empl. canthar. magn. nuchæ. Enema aloeticum. P. c. lot.*

Half-past 7, p.m. The medicine operated upon the bowels briskly; only one dose of the croton oil was taken—pulse 110, rather softer—pupils dilated—face flushed: complains of great pain of the head—rolls about the bed, his knees almost at his chin—great thirst and dry tongue.

*Hirudines*, No. xviij. *temporibus admovend. Lotiones continuantur.*

℞. *Hydrarg. submur.* gr. x. *Pulv. antim.* gr. iij. *Pulv. opii*, gr. jss. *M. h. s. sumend.*

June 30th, 9, a.m. Has had a very restless night—talks very much and incoherently—passes his urine and fæces involuntarily—pulse 108, full and hard—thirst urgent—great heat of the scalp—pupils continue dilated.

*Hirudines*, No. xx. *ponè aures. Venesectio ad syncopen. Emplast. cantharid. toti capiti applicand. Lotiones continuantur.*

6, p.m. Begins to be more sensible, and complains of great pain in the head—pupils rather less dilated—pulse 120, small and feeble—has slept three hours, and taken a pint of milk. The cold applications to the head to be continued.

July 1st, 9, a.m. There is little alteration to be observed in the condition of the patient—has had a very restless, sleepless night, tossing about the bed, swearing, singing, and whistling alternately. The head has been well blistered; pulse 106, and strong—passes the excretions involuntarily—a little less pain of the head—scalp continues very hot—the breathing is rather stertorous.

℞. *Hydrarg. submur.* gr. iv. *Pulv. opii*, gr. ijss. *M. h. s. sumend. Sectio arteriæ temporalis, et detrahantur sanguinis unciæ decem.*

July 2d. Has slept four hours—pupils less dilated—headache better—pulse 106, and softer—no evacuation from the bowels—tongue furred—complains of thirst—continues for the most part delirious. *Lotiones continuantur.*

℞. *Hydrarg. submur.* gr. v. *Antim. tart.* gr. 1-3d, *statim.*

July 3d, *mane.* The early part of the night he has been tranquil—slept three hours, but awoke very delirious—face flushed—pulse 120, and strong—skin and scalp hot and dry—tongue furred and brown—pupils dilated—bowels unmoved—falls into a comatose state if undisturbed.

*Habeat Ol. croton. tiglii*, gtt. ij. *statim. Rept. V. Sectio ad* ℥xvj.

℞. *Ant. tart.* gr. jss. *Tinct. digitalis.* ʒjss. *Liq. ammon. acet.* ℥iv. *Aquæ, q. s. ft.* ʒvj. *M. ex quâ Cochl.* ij. *ampla tertia quaque hora capienda.*

*Vespere.* Continues very delirious. Pulse 125—bowels freely moved—skin moist—pupils still continue dilated: has taken a pint of milk. The remedies to be continued, and a blister applied behind each ear.

July 4th. Has passed a better night—bowels continue free—skin moist, and less hot—blisters operating well—pupils less dilated—gums tender—can answer questions when roused, but falls again into a comatose state.

*Lotiones et mistura continuantur.*

July 5th. No untoward symptoms have appeared since yesterday—bowels have been twice moved—pupils less dilated—has slept a few hours—continues rather delirious, and complains of pain in the head.

*Lotiones et mistura continuantur.*

℞. *Hydrarg. submur.* gr. x. *Pulv. jalap.* gr. xvj. *statim sumend.*

July 6th. Has slept little—bowels have been moved twice—skin moist—pulse 118—can give no distinct answer to questions—complains of pain in the head—tongue dry, and mouth parched—thirst urgent—gums tender—very restless.

*Hirudines,* No. xij. *capiti. Contin. mistura et lotiones.*

℞. *Hydr. submur.* gr. x. *Pulv. antimonialis,* gr. iij. *Opii,* gr. ij. *M. h. s. sumend.*

July 7th. Slept four hours—appears more sensible—pulse 98—pupils a little dilated—bowels moved once—has taken a little milk and porridge—answers questions more distinctly—less headache—the action of the voluntary and involuntary muscles more perfect.

*Cont. mistura et lotiones. Rep. Calomel et Opium hora somni.*

July 8th. Has slept five hours, and says he feels better—pulse 100, and soft—skin moist—can answer questions more distinctly when roused—is rather noisy at times—bowels have been moved twice during the night.

My patient being a pauper, belonging to the parish of Ashton-under-Lyne, was this afternoon removed to the parish hospital, a distance of six miles, and placed under the care of my friend, Dr. Campbell, of that town, by whom I am informed, that he found it necessary to continue the antiphlogistic treatment for three weeks before any very decided amendment was perceptible, and that a considerable discharge of pus took place from the ear. The man is now following his usual occupation, and enjoying good health, without any diminution of his mental faculties.

JAMES ELLIOTT, Surgeon.
*Lees, near Oldham, Lancashire,*
*Nov. 24th,* 1831.

---

XLVI.

### EXTENSIVE FRACTURE OF THE PELVIS, AND RUPTURE OF THE BLADDER.

THE following case has also been transmitted to us, and is interesting as shewing the quantum of injury that may be borne. Rupture of the bladder is always a formidable, generally a fatal injury, and in the present case it was unusually severe.

*Norfolk Island, 12th Dec.* 1831.

Thomas Asmond, prisoner of the Crown, labourer, æt. 21, of robust make, and sanguine temperament, was brought to the hospital this afternoon, 5, p. m., in consequence of a bank of earth at which he was working having fallen upon him. He complained of extreme pain in the hypogastric and iliac regions. Said he felt "something give way at the time of the accident in his belly, and as if his guts had fallen down into his purse." He also complained of great pain in his right hip which he said "was out." Face pale and covered with a cold clammy perspiration—lips livid—pulse scarcely perceptible—a small quantity of blood issued from the penis—could pass no urine; the catheter was introduced, but only a little blood was passed. Thirty minims of tincture of opium were administered with a little wine. Fotus abdomini.

Half-past 9. Much the same—still low—wine and water from time to time. Inj. enema com.

13th. Passed a very uneasy and restless night; had only one motion during the night, and that, partly of the injection mixed with blood. Scrotum greatly distended with blood and serum, and half of the penis injected also. Two incisions were made in the scrotum, from which a quantity of bloody serum escaped. Cont. fomentatio. Pulv. purgant. ʒss.

Evening. No effect from the powder, and an enema was thrown up, which produced no effect, and two drops of croton oil were given, from which he had three thin watery stools. Penis completely injected; abdomen extremely painful, tense, and tender to the touch. Pulse 124, small, but rather hard. V.S. ad levamen symptomatum—semicupium.

14th. Eighteen ounces of blood were only abstracted, which exhibited a firm coagulum, without any other marks of inflammatory action. Had a most uncomfortable night—several thin stools from a purging draught taken early this morning. Abdomen tense, but not so tender. Pulse weak and intermittent. Distention of scrotum and penis much complained of. Several punctures made in both, and a quantity of thin fluid mixed with blood escaped. Cont. fotus et potus vegetabilis.

Evening. Has been very restless and uneasy—frequent thin stools—occasional delirium—pulse intermittent—mouth dry and parched—in short, he appears to be rapidly sinking.

15th. No sleep—very uneasy, but suffering less pain. Had an inclination to make water, but could not. Catheter introduced, and a small quantity of urine mixed with blood was drawn off, which he thought afforded him considerable relief. Copious discharge of bloody serum from the scrotum. Cont. fomentatio—nec non potus vegetabilis.

16th. Symptoms most unfavourable —gradually sinking, and died at one o'clock, p.m.

*Sectio Cadaveris.* All the soft parts in the hypogastric and lumbar regions seemed to suffer from contusion, and the scrotum and penis greatly distended with blood, as well as the muscular parts about the perinæum. On opening the abdomen, the intestines were much distended with flatus, and a slight blush of inflammation was on the small intestines. My attention was principally directed to the hypogastric region, which was the seat of his injury. I found the bladder nearly empty, and on passing my hand into the pelvis, a rupture was discovered at its fundus, which the symptoms on admission into hospital warranted my suspecting to be the nature of the injury. On further examination, a second rupture was discovered at its neck close to the prostate. From the extensive injury, the quantity of blood effused into the surrounding structures would prove the laceration of numerous blood-vessels. As the man complained of great pain in his right hip, my attention was next directed to that part, and I found that the injury to the bones, composing the pelvis, was most extensive. A fracture of the horizontal ramus of the pubes with the ileum, passing through the thyroid foramen, and extending through the ischium at its tuberosity. The attachment of the bladder to these bones was completely torn through, and it was at this place where the rupture of the fundus was observed—other fractures of this bone were found, one extending backwards and downwards from the middle of the crista of the ileum to its connexion with the sacrum, and another from the posterior part of the crista to the same connectedly, both fractures meeting at the upper part of the junction of the two bones. The opposite bones of the pelvis were fractured nearly in a similar manner. All the other viscera were healthy.

*Remarks.* On the receipt of the injury this man was in rude health, and from the detailed statement of the severe injury he sustained, no plan of treatment could save him. The shock which his system had sustained was so severe, that I thought, on his being brought to the hospital, that his dissolution must be immediate. The stimuli which were called for produced a sufficient reaction to keep up the circulation throughout the night. As soon as any tenderness on pressing the abdomen appeared, the lancet was employed with other suitable remedies to check inflammation; but such was the shock to the nervous system, that no reaction of any importance was produced, and therefore it is to be presumed that death was hastened by the depression of nervous energy.

Alex. Gamack,
Colonial Assist. Surg.

---

### XLVII.

### Cases of Phlebitis—Advantages of Free Openings.

We have already noticed the Madras Reports published by the Medical Board in 1831, and kindly presented with the sanction of Government, to the Editor of this Journal. It unnecessary for us to say how much we approve of the practice, becoming daily more general, of publishing the Transactions of Societies, the interesting cases that occur in public institutions, and the documents in the possession of public boards. In such publications there are fewer inducements to distort facts, to magnify success, or conceal failures, and therefore we can place more reliance on what is offered us, than, unfortunately we can always venture to extend to more private statements. We therefore beg to offer our meed of approbation of the plan pursued in the present instance by the Madras Board.

We are tempted to notice some cases of phlebitis offered as illustrative of a mode of treatment by an operation, practised by the late surgeon Thomas Bond, of the Madras European Regiment.

CASE 1.—*Phlebitis—fatal.* J.R. private, æt. 39, 14 years resident in India, was admitted into hospital January 27, 1827, with symptoms of spasmodic asthma, &c., for which he was bled in the right cephalic vein, and took mercury to ptyalism. On Feb. 2, he complained of the arm being very painful—it was much inflamed round the joint, very tender to the touch, and a drop or two of matter exuded from the orifice on pressure. Thirty leeches and a poultice were applied, and next day it was still swelled, but without pain; he looked very anxious and sal- sallow. He was allowed some port wine. In the evening he had an opiate followed by some aperient medicine. He vomited after the medicine, and next morning the arm was much swelled and very painful, and his countenance was worse. He was ordered camphor, and the leeches were repeated. On the 5th the swelling was extending up the shoulder, the puncture closed. We may mention that the cellular membrane appeared more affected than the vein, and in the evening of the 6th the swelling and tension were greatest at the inner side of the arm and not in the course of the vein. The treatment consisted in mild aperients, opiates, and baths. On the 7th he was delirious, the pulse 86 and very weak, the countenance extremely anxious. In the evening he was more delirious, the pulse 130, the skin hot. On the 8th we find him with low muttering delirium, countenance deadly, pulse 100, tongue 'discoloured.' *Ordered quina and wine and water—a blister to the spine.* The arm was scarcely larger than the other. He sank and expired at 2 A. M. of the 11th.

*Sectio Cadaveris.* "Coagulable lymph was thrown out in the cellular membrane. The integuments around the wound adhering strongly together, and to the wound in the vein. The vein quite closed. The vein was laid open and found to be thickened, and full of pus, up to the insertion of the deltoid muscle, from which place it was empty and sound. The abdominal viscera, heart, and the lungs, were healthy; but the latter adhered to the pleura costalis, by old adhesion."

In perusing the foregoing case we cannot but be struck with the vacillating treatment pursued. The patient is leeched on the 2nd, ordered port wine on the 3d, camphor and leeches on the 4th, and so on. However, those who have seen many cases of phlebitis will probably be most charitable in judging of treatment.

CASE 2.—*Phlebitis after Venesection—Operation—recovery.*

Ensign H. æt. 19, resident in India one year, was bled to syncope (℔ijss) in the night of Feb. 14, 1827, for dysenteric symptoms. He used the arm much in the night, and the bandage came off. The night of the 15th was very restless, he vomited frequently, and had much heat of skin and headache, with furred tongue, pulse 120. The arm was painful, but not swelled, there was a little redness round the puncture, and on pressing around it with a sponge a small quantity of pus and blood was discharged. He had taken since the 14th a scruple of calomel, and two of blue pill. *Wound closed with sticking plaister**—*cold lo-*

* This we think decidedly bad practice. A light poultice should have been applied.—*Rev.*

*tion—and purgative medicine.* At 2 P. M. the arm was more painful—skin hot and dry—had vomited—more pus pressed from puncture. In the evening 18 leeches were applied and afterwards a compress was put on four inches above the puncture. *Calomel and opium—salines with laudanum and antimony—bath.* On the 17th the arm was more swollen below the compress. There was little alteration till noon of the 18th, when there was increased heat of skin, the arm was more painful and swollen towards the compress, and more matter was pressed from the wound by drawing the finger down the vein, from the compress to the puncture. At 3 P. M. the vein was swelled up to the shoulder, with great constitutional irritation. The following operation was performed.

"An incision was made with a scalpel through the integuments, and the median basilic vein exposed, a director was then introduced through the puncture, and, with a probe-pointed bistoury, the opening was extended above and below as far as there was any appearance of pus, which was carefully extracted by pressure, and the wound closed in the usual manner. The application of the compress was continued till the 22d in the morning."

About ʒij. or ʒiij. of pus were evacuated, and the wound was dressed with a resino-terebinthinate ointment. In the course of half an hour the constitutional irritation had remarkably subsided, and the countenance, previously extremely anxious, was more composed. The same plan of treatment was persevered in, the patient taking a calomel and opium pill at night, with a diaphoretic opiate, and aperients. On the 19th, he was ordered quina, arrow-root with wine, and camphor. On the 20th he was improving, the arm was dressed and better: it discharged freely. In the evening he was worse, the pulse being much quickened, with pain in the chest. *Calomel and opium and an anodyne.* After the increase of heat perspiration occurred, and in point of fact there was a paroxysm of fever, though without any perceptible rigor. Next day he was better, but had a slight cough. We need not pursue the details. On the 24th the arm was well, and some derangement of the hepatic function with a slight cough was his sole complaint. He soon perfectly recovered.

CASE 3.—*Phlebitis after Venesection—Operation—Recovery.*

John Palmer, private, æt. 24, resident in India two months, was admitted, March 16th, 1827, with acute hepatitis, for which ℔ij. of blood were taken from the right cephalic vein. The treatment for inflammation of the liver was continued; but, on the evening of the 18th, he complained of pain in the arm, which was swollen, and the vein was found inflamed—pulse 100—headache. On the next morning he was free from fever, but the arm was as before. A compress and bandage placed on the vein, above the puncture, and the whole arm laid on a soft poultice. In the evening the arm was more painful and swollen, the inflammation reaching as high as the compress, though no pus appeared at the puncture—pulse 100—headache—fever—anxious countenance. *Calomel c. Pulv. antim. et Op—Baln.* He passed a feverish night, but next day he was cool, with furred tongue and anxious countenance; a little pus could be pressed from the wound.

"An incision extending from one inch below, to one inch above, the puncture, was made into the vein; its coats were found to be much thickened, its cavity larger than natural (on account of the compress placed on it above), and containing from about two, to three, drachms of pus. The inflammation does not extend beyond the compress; but a little pus was pressed from the portion of vein next the hand. The wound was dressed with ungt. resin. flav. with a little oil of turpentine added to it. The arm again placed in a poultice."

He was ordered quinine, and under its exhibition, with occasional purgatives, no further unfavourable symptoms occurred. On the 23d, he is reported as getting rapidly well, and in a few days the arm healed.

CASE 4.—*Phlebitis after Venesection, fatal—Pus in the deeper Veins.*

T. H. private, æt. 22, in India five months, admitted June 27th, 1827, with feverishness and headache. The median basilic vein was opened, but, not bleeding freely, the cephalic in the same arm was cut, and ℔j. of blood extracted, with relief. He was leeched thrice, and appeared to be doing well, till the morning of the 30th, when the arm was painful; both punctures were inflamed, but the inflammation appeared to extend up the cephalic vein, and not up the basilic. A pad and bandage were applied about the centre of the arm, and cold lotion used. In the evening the arm was worse, with fever. The cephalic vein was laid open as in the former cases, and ʒij. of pus discharged from it, a little being pressed out of the median basilic, and a little from the puncture in the basilic. The arm was laid in a poultice; the treatment much as in the former cases. On July 1st, he seemed better, a little matter oozed out at the puncture in the median basilic, without apparent inflammation; the gums were tender. On the 3d, we find no pain or inflammation in the arm, the wound suppurating and looking well. Ordered quinine and wine in arrow-root. On the 5th he had pain across the pit of the stomach, and leeches were applied. In the evening, he had that all-but-fatal symptom in such cases, severe rigors and fever. Next day he looked ill, his appearance indicating mischief, though the arm shewed nothing amiss. The median basilic vein was laid open, and found to have a black appearance. In the evening of the 7th he had another attack of fever; and, on the 8th, he said he felt better, "though he did not look so," a very characteristic symptom. He gradually sank, and died at 7, p.m. of the 11th.

On dissection, the veins which had been operated on looked healthy, but the deep-seated veins, especially the brachial, were found full of pus. No mention is made of the state of the liver or lungs.

We have analysed these cases, because we think they inculcate an important point of practice, that of laying open freely an inflamed and suppurating vein. The secondary consequences of phlebitis, as of other injuries, are most frequently observed when matter is locked up or does not freely escape. It is on this account that diffuse inflammation of the cellular membrane, between muscles, is so much more frequently followed by visceral deposites, than diffuse inflammation of the subcutaneous cellular tissue. We do not place much confidence on the employment of pressure by a pad; on the contrary we are tempted to believe that it is injurious, by the mechanical obstruction offered to the circulation of the limb. Every surgeon is aware how much any ligature, or compression on an inflamed limb, interferes with the natural processes of reparation. We suspect, from the manner in which the foregoing cases are drawn up, that the reporters are not aware of all that has been done in the investigation of the secondary inflammations. Thus, in the last case, we are informed, after the occurrence of rigors, that "the arm does not seem to be at all connected with his ill-health, but the disease to be in the liver, in the bowels." No surgeon who had seen many cases of the purulent depôts would make such a remark. However, we again beg to draw particular attention to the practice employed in some of these cases, and to advocate most warmly the free opening of inflamed veins, when the surgeon has reason to believe that they contain pus, which does not escape with the utmost freedom and facility.

---

### XLVIII.

### CHOLERA.

WE have lately dedicated so much of our space to cholera, that both ourselves and our readers must be somewhat sick of it. If we say little at present, it is because there is really little to say—little, at least, that is new. It is true that every day brings a fresh specific; yet, strange to tell, the lying cholera-list shews no alteration in the relative proportion of recoveries and deaths. There must surely be something rotten in the state of Denmark, when, out of the multitude of certain, or nearly certain remedies, from the tincture of the Russian Merchant on Cornhill to the salts of Dr. Stevens, none have yet effected, what all pretend to effect, a diminution in the gross mortality. Amidst the variety of remedies presented to our notice, we feel like a hungry guest with a splendid bill of fare—each article tempts, but which shall he prefer? One gentleman cures cholera with cold water—another removes it with hot—a third puts it to flight with calomel and opium—a fourth exclaims that calomel and opium are poison, and drenches with salt water—a fifth declaims against the absurdity of salines by the mouth, and throws them into the veins—a sixth—but why should we go on? It is enough that all are sanguine, all successful, all full of cases confirmatory of their statements; yet, notwithstanding, the cholera kills as many as if all the projectors, and all the schemes, were still in the wombs of the journals.

With our boasted civilization and intellect, we walk the same mill-horse round we have walked before, and that others have walked before us. When cholera appeared in Hindostan, the papers so teemed with specifics and cures, that the Government put a stop to their further publication, on account of the mortality they caused. Our Government has not the power to do this; and we may thank our forefathers that, in this happy country, any one may cure himself to death as he pleases. Gentlemen are at liberty to publish their miracles, and the faithful are equally at liberty to believe them. For ourselves, what shall we say? Alas! we must own that we are gloomy, heartless sceptics, without so much as a grain of faith, or one single, saving particle of belief. Would that it were otherwise—would that we could only so much as imagine that cholera has been, is, or will be cured by the thousand and one plans, of happy memory, already published, publishing, or to be published. Carbonari that we are, we believe not a word of it.

In sober sadness, when we look at the official reports, and when we pay regard to our own experience, we cannot attach the slightest importance to the statements of cures in the weekly and other journals. In point of fact, we know no better mode of treating cholera now than when it first appeared in the island, and the really severe cases are just as fatal as they ever have been. The only solid benefit that has hitherto been derived from the investigation of the malady in this country, is the attention directed to the premonitory symptoms, especially the diarrhœa.

We have frequently been asked if our opinions on the question of contagion have been modified by longer experience. We say most unhesitatingly, no. We were contingent contagionists, and contingent contagionists we remain. Under favourable circumstances we have believed and do believe in the propagation of the disease by contagion, but under ordinary circumstances we deny it. Who sees it spread in a family where ventilation is procurable and cleanliness observed—how many have been attacked in the household of Mrs. Smith, of Lord Holland, of the Bishop of London, nay, even of Majesty? But we are not about to rip up this discussion, for we think it acknowledged on all hands, that few but the most bigoted and most blind imagine that cholera is the virulent contagion which many would have us believe it.

The Medical Gazette has lately related two circumstances not undeserving of attention. The first is the appearance of cholera in Canada. It is stated, (we cannot lay our hands on the number,) that the disease appeared soon after the arrival of an emigrant ship from an Irish port where the cholera prevailed, and that in the first instance it attacked the emigrants, and from them spread to the inhabitants of Quebec. Such statements should hardly be received on vague authority, not even on the word of that omnipresent personage, "a correspondent on whom we can rely." It will be time to pay attention to such facts when they come in a questionable shape. Our contemporary, indeed, appears to have a fancy for such props on which to lean his editorial elbows. A little while ago he assured us on the representation of "an estimable physician," or some such satisfactory testimony, that contagion was proved to demonstration in Edinburgh. Ah! Dr. Craigie what are you about? proved to be roaming and snubbed in your own Athens!

We beg pardon for troubling the Gazette, but one word more and we have done. It cites with an air of something not very unlike triumph the recantation of M. Velpeau. This gentleman signed the famous anti-contagious *compte-rendu* of the Parisian faculty, on the outbreak of cholera in that metropolis. He has since found reason to change his opinion, and published the facts which have led him to do so. We find no fault with M. Velpeau for this, nor do we complain of the Gazette for noticing it. It slily chuckles at the Frenchman's reformation. We protest we quite envy it that saintly and celestial joy which it must feel over *one* repenting sinner. We almost wish we were contagionists ourselves, to feel the fragrance of a delicious sensation that can hardly be conceived on this side the grave.

The Board of Health has published a new circular, in some respects unlike the last. We have often thought that such instructions, issued by Boards of Health, not only in this Country but in others, are either useless or injurious. They can scarcely be fitted at once for the public and the profession, and yet, if they are separately addressed to one, they are apt to be neglected by the other. Regulations must necessarily be, in some degree, issued *ex cathedrâ*, yet it is not to be supposed that professional men will recognize, in Boards, the right divine of deciding on points which occupy their own attention, and are yet in abeyance. If a Board, for instance, meddles with the question of contagion, it must necessarily displease one party, and, differing from it in opinion, must be exposed to all the consequences of free party discussion. These are difficulties that must surround all Boards in the present day; and we cannot be surprised that the present should be sensibly affected by them.

To point them out more clearly, let us enter a little into detail.

In the paper before us, which is somewhat lengthy, and contains 41 paragraphs or sections, we find twelve prescriptions, two for pills, three for powders, three for draughts, one for mixture, and three for external applications. Now it is to be presumed, that each is adapted for some particular feature of the complaint, some situation in which the unhappy individual may find himself suddenly and inopportunely placed—it must be presumed, in short, that the prescriptions are neither too numerous nor too few. Then he is to get them all, but when got how is he to use them? We suppose that the code of instructions to which these prescriptions are appended, will instruct him. Let us look at these instructions—we will just take 39 and 40.

"39. It often happens, however, notwithstanding all our Care, that the Re-establishment of the Pulse and Heat are closely followed by Symptoms of Fever, by some Degree of Stupor, by great Oppression of breathing, by Distension and Tenderness of the Belly; all of which indicate Danger.

40. The moment such Symptoms appear, bleed from the Arm, or from the Part most affected, by Leeches or Cupping, to 10, 12, or 16 Ounces, according to the Effect produced by the Bleeding. Reduce the Temperature of the Patient's Room, give cool Drinks, and apply cold wet Clothes or pounded Ice in Bladders to the head; and give the Powders, No. 5, as already ordered."

It is evident that this is not directed to the patient but to the medical attendant. So the directions become of little or no use to non-professional persons, and are intended for the guidance of the profession. But if promulgated with such a view, why enter into trifling details, why omit all mention of such agents as saline injections, &c. In fine, turn it over how we may, we see very many inconsistencies—the document is too particular for patients, too general for the profession. The former cannot comprehend it, the latter will not respect it. Besides it is ridiculous to observe the dogmatical tone which such directions breathe. Like the centurion, Boards are somewhat positive; they say to this prescription come, and it cometh; and to that symptom go, and it goeth. The whole affair is, as Nebuchednezzar's image, one foot iron and the other clay. The only chance which a circular has of being serviceable against cholera, is by being applied in removing an usual and disagreeable effect of the extreme alvine irritability attending it.

We had just penned these reflections when we received the Lancet for Sept. 8th. The leading article is devoted to the proposal of a travelling cholera commission of three, to be "placed under the orders of the Board of Health." The triumvirs are to be men of "indisputable integrity, acknowledged ability, and ample experience of disease in general, and this disease in particular." Where these black swans are to be found we are not told, but doubtless there is *somewhere* a stock, like the one pound notes that saved the Bank, from which the nation may be furnished in its need.

"An inquiry of this kind should be instituted at the expense of the *nation*, and conducted by medical men of indisputable integrity, acknowledged ability, and ample experience of disease in general, and this disease in particular. The *clinical investigations* should be pursued in the localities where the epidemic presents itself in its worst forms.

To meet these objects, a travelling commission, consisting *at least* of three medical gentlemen, should be formed, and placed under the orders of the Government Board of Health. Their business should be to repair to the worst scenes of the epidemic at a moment's notice, and there put in practice any method of treatment directed by the Board. To afford a fair field for their investigations, the expense of the temporary experimental hospital should be defrayed from the *national* treasury.

In giving directions to the moveable commission for the institution of these clinical trials, the Government Board should not be altogether invested with a discretionary power. We must explain.—No proposal of treatment, for example, should be carried into extensive trial at the public expense until some evidence of its probable value is previously obtained. Thus the Board should not be permitted to institute a method never before tried, and merely proposed on uncertain theoretical data. Sufficient evidence should be first laid before them of the promising nature of the proposal.

The moveable commission should be in every respect the agents of the Board, and be totally deprived of any discretionary power in the treatment of the cases, ample and minute directions being in every instance given by the Board. As the proposer of a practice would probably desire to see his intentions exactly carried into effect, he should be permitted, or rather invited, to inspect the treatment of the cases, and see that everything was accurately done. The expenses of the proposer should of course be defrayed from the public treasury, should a favorable report be made on his method by the moveable commission.

As the peculiar service of the commission would expose them to constant and intense danger, provision should be made for their families in the event of their falling victims to their public labours.

In the management of any system of treatment, the cases should be visited *at least every hour*, and minute reports forthwith entered in a book, and signed by the officer in attendance. Each method of treatment should be carried into trial on from ten to fifty patients. A report should be made on the termination of each general experiment, and such report published in the medical and other public journals."

When such propositions are seriously made, there is no use mincing the matter. The profession would place no confidence in any commission of the kind, for who would pretend to select three men, who could fairly be considered as fit for such an office, or really the delegates and representatives of the profession. So far from seeing any benefit in such commissions, we should apprehend nothing but an interminable system of jobs, without any advantage to the public equivalent to the expense, any satisfaction to the profession, or any benefit to science. The Lancet, indeed, is sanguine, nay it even imagines that a specific may already exist, locked up perhaps, like Solomon's seal, in a portmanteau. "It is *not impossible*," says he, "that a *specific* for cholera has already been discovered." Nay the idea has so laid hold of the imagination of our contemporary, that he even pictures to himself the discoverer of the secret, in the new commission, becoming the possessor of a title! Baron Cholera, of Cholera Hall, would certainly sound lordly and well.

"Even supposing the contingency of wealth and title to be the reward of the discovery of some proved specific, does there exist the wretch who would grudge the outlay for such deserts?"

We are sure we would not.

But we pass to another subject, having more of substantial and melancholy reality around it. We allude to the recent riot at Manchester, of which the particulars have been given in the newspapers. We cannot but agree with the editors of the Herald and Times, in lamenting that an excuse should have been presented to a misguided and ignorant mob, for allowing their passions to lead them to cruelty, outrage, and destruction. The folly of decapitating a boy dead in a cholera hospital, reckless or ignorant of what consequences might arise, deserves the most severe reprehension. But this was only the immediate cause of the tumult in question; the real and efficient one lies much more deeply. It is to be found in our police regulations, in our quarantine, in short in the contagious doctrines and regulations formerly promulgated by authority. Most medical men have remarked the strong sense in medical matters frequently displayed by the editor of the Times. On the present occasion he has shewn his usual sagacity and tact. The article is short and we insert it.

"It is distressing to witness among the sensible people of this country a scene like that which occurred at Manchester on Sunday last, (a description of which will be found in another column), and it is still more distressing to reflect that any cause should have been given for such a popular tumult by a previous outrage on popular feeling. The poor and uninstructed, though sensible to unequivocal kindness, are but too apt to be suspicious of ill-usage, where the dearest ties of life are concerned, and where, in their view, distinctions are made between their own relatives and those of their more powerful neighbours. Hence in epidemics, where police regulations are adopted to prevent alleged contagion, or particular modes of medical treatment are authoritatively enforced to dispel disease, they are prone to believe that they are hardly dealt with, merely because they are poor,—that the doctor is their enemy, if not their future assassin,—that the wealthy classes desire only to consult their own security at the expense of their indigent brethren,—that the hospital is intended as a preparatory depôt for the grave, and that the period of interment is hastened, or the mode of interment is altered, to conceal the violence perpetrated on the dead or to inflict insult on the best sympathies of the living. Hence the riots and the reports of poisoning which occurred when the cholera reached Paris; hence the rebellion against the magistrates and the faculty in Hungary; and hence the general aversion of the common people in all countries to those regulations which remove their relatives from their personal care, or order them to be buried at a shorter interval after the fatal termination of the malady.

In the recent case of Manchester there was, unhappily, a new circumstance of suspicion to warrant a more violent explosion of the common feeling. The child had most improperly been converted into a subject for dissection, and his corpse had been mutilated before interment, though nobody but the most ignorant and thoughtless participator in the tumult could have imagined that he was murdered in the hospital. We regret this circumstance the more, as, connected with the new anatomy bill, it will create alarm in the minds of those whose circumstances may require hospital relief, that if they die, like this poor child, under medical treatment, their bodies may be refused to their relatives, as his was to his grandfather, and reserved for anatomical dissection.

But this is not the purpose for which we have alluded to the Manchester tumult or its cause. Our object is to suggest the policy or necessity of *changing that system* with respect to this epidemic which has given so great, and in our opinion, such just cause of offence to the poorer classes who have lost their relatives, or are themselves threatened by its attacks. *Why is a particular spot selected for the interment of persons who die of the cholera?* Why are their relatives compelled to bury them when they have a suspicion in their minds that they may still be alive? No precaution has yet prevented the spread of the malady, and if it be contagious, it is the most capricious contagion which was ever known. Physicians who attend the hospitals are more likely to spread it than the undertaker, although he were allowed to execute his work in the regular way, and after the regular time. At any rate, it may be supposed to be buried in the grave which contains its victim, and is not likely to rise to attack the present generation. *We are convinced that more evil than good has been caused by these regulations to which the common people have shewn such aversion, and which have prevented them from deriving all the advantages which they might* from the means of relief or cure placed within their reach."

Every syllable of this is true. Why what a mummery it is to select particular spots for the burial of the dead; what a disgusting exhibition of humbug and of folly. Are the quiet tenants of the grave to be protected from the contact of the pestilential corpse? do their buried ancestors refuse commune with the cholera dead? or are the burials so numerous as to taint the air of the ordinary church-yard? Common sense would whisper that, if infection reside about the bodies, it would surely be more efficient when collected and condensed into a focus. But common sense has nought to do with some of the regulations upon cholera.

And then again the cere-cloths, and the wrappings, and the speedy burials. Against whom are these *enforced*? Against the rich? Oh no! The policeman enters not the titled hall, and the pestilent corpse of the fashionable lady is not borne off by watermen paid five shillings per head, for their damnable profanation of the feelings of surviving parent or child.* Had another, and a conciliatory course been adopted, had the feelings of the people not been scarified, their evil passions roused, and their fears exasperated, by the regulations which have at former times been promulgated, the cholera might not have been rendered less fatal, but there would at all events have been more good will, more community of feeling between the rich and the poor, and greater resignation in the latter to the evils inseparable from their lot. We take this opportunity of observing, that the Board in their last circular have certainly disavowed all wish or attempt at coercion, and allowed that, except under particular circumstances, the attendants of the sick need feel no alarm, nor apprehend any danger from infection.

We have thought it might be useful to throw together the substance of the many communications on cholera that appear in the weekly journals. Our readers may possibly be reminded of the facetious gentleman who kept a basin on his knee at a dinner party, and privately deposited in it whatever he was asked to eat and drink. When the cloth was about to be removed he exhibited his basin with its contents, and asked

---

* A magistrate directed twenty watermen to be procured, to carry off and bury by force the corpse of a cholera patient in Seven Dials. Even the police had refused the abominable office. And yet we wonder that the lower orders are excited.

the company what his stomach must be composed of to digest such a heterogeneous and contradictory mixture. Our readers may apply this story as they please.

### Excerpta Choleralogica.

### I. Saline Injections into the Veins.

The well-known fact that, in cholera, the serous and saline parts of the blood are rapidly thrown out upon the mucous surface of the bowels, naturally enough suggested the idea of supplying the loss by direct injection of a sero-saline kind into the veins. To throw such a fluid into the stomach or bowels, where it was already in superabundance, and at a time when all powers of absorption are suspended, appears the wildest and most absurd scheme that ever was invented, as the result has shewn. Contrary to expectation, the saline injections were not only borne with apparent safety, but, in almost every instance, produced a speedy, and as it were, a resuscitating effect on the whole system, restoring the pulse, the temperature, and the plumpness of the body. The prospect was too flattering to be realized. Such a gigantic power over disease—it might be said over death, was more than Nature would permit, and accordingly we find two important draw-backs on the new remedy. Either the fluids run off by the bowels with more rapidity than ever—or the head or lungs become oppressed, and death takes place in a different manner than by cholera. Of both these events we have seen the most unequivocal and incontestible proofs. While therefore we bear testimony to the astonishing—the almost miraculous physiological effects of the saline injections, we are reluctantly compelled to bear witness to the pathological phenomena which succeed. We are grieved to say that, in our opinion, a remedy is yet to be sought for the collapse of the epidemic cholera. Till this be found, our only resource is vigilant attention to the first stage or diarrhœa, with which the disease almost always commences, when a milder form of the flux does not precede. We are now to notice some communications from practitioners on the subject of saline injections.

Dr. Lawrie, of Glasgow, whose able work on cholera we have already noticed in this Journal, has stated the results of his observations in the Medical Gazette for June 7th, 1832. Following the directions of Dr. Latta, he injected from 70 to 150 ozs. within a few hours —and all his patients died, viz. six in number. In all of these temporary benefit was produced; "but in several the fatal result seemed accelerated." In one case he then tried the addition of albumen to the saline injection—in another the serum of bullock's blood—in a third, human serum—in a fourth, human blood—in two cases, small quantities of whiskey—in two, a few drops of laudanum—still *all* died. Dr. L. then began to suspect that the quantities injected were too large, and they were limited to 30 ounces at a time, thrown in very slowly, and carefully watching the effects. Under these precautions, four have recovered; "but so many have died in despite of all precautions, so evidently injured by the practice, that I have now almost entirely laid it aside, as not only useless but frequently hazardous." Dr. L. injected 26 cases, 22 of which died! Dr L. witnessed the phenomenon to which we have alluded—the drain of fluids from the bowels as fast as the injections were thrown into the veins. One patient made this remark—"as fast as you put water in my veins, it runs out by my stomach."

---

II. Mr. Buchanan, of Hull, in the same journal, has communicated some remarkable instances of cholera cured by somewhat unusual means. He premises his cases by stating it as his conviction that "this disease arises from malaria, sui generis, combined with a certain state of the atmosphere which acts with fatal rapidity on the human frame." The following recipe is that which has been so successful in the hands of Mr. B.

℞. Ol. oliv. ℥j.

Tinct. acet. opii, gt. xc.

Ol. cinnam. gt. xx.

Tinct. iodinæ, gt. x.

Sp. vini rectif. ℥j.

Spir. æth. sulph. ʒj.

Tinct. acaroid (?) ʒij. Misce ft. mistura.

One-half of the above mixture, (to which was sometimes added tinct. assafœtid.) was given at once, and the remainder in half an hour. In very urgent cases he administered the whole at once, using external heat and the usual means at the same time. In 24 cases there were only three deaths. Either the remedy is peculiarly efficacious, or the cases were of a milder description than we have been accustomed to see. But the fact is, that if cholera cases are seen early, very trifling remedies will be often successful; whereas, if the collapse has commenced, the most vigorous means will fail in nine cases out of ten.

### III. Bandaging the Abdomen in Cholera.

This is among the newest proposals for the intractable epidemic. It is founded on a mechanical rather than a physiological idea. In *one* case, the only one in which it was tried, Mr. Bowden, of Sloane Street, "found a compress and tight bandage to the whole abdominal surface effectually restrain the *serous hæmorrhage* from the bowels; and, as a farther and more remote consequence, but an important one, perhaps, restore the secretion of urine." Calomel and opium, with the saline medicines, were used at the same time. Whatever efficacy the tight bandage may possess, we conceive the expression "serous hæmorrhage," one of the best that has yet been applied to the disease, even although it does express an inconsistency in denominating a flow of blood a flow of serum.

### IV. Anodyne Paste for Cholera.

Dr. Williams, President of the Ipswich Central Board of Health, has published a small pamphlet, with the view of recommending an opiate paste, in the form of suppository, in the treatment of cholera. Its effects have been "so strikingly salutary, so immediately remedial, and eventually so successful, that he dares venture to congratulate the suffering community on its discovery." It consists of "one drachm of the best soft crude opium not dried or powdered, and seven drachms of hard soap, which being intimately blended and well pounded in a mortar, forms a solid paste, of which the quantity introduced into the rectum on each application, is about 2½ grains of soft opium and 17½ grains of soap. The paste should be conical in shape, like a suppository. If the bowels be severely excited, it will be desirable to delay the application of the paste till some pause or relief in such excitement take place, or till after such exhaustion or weakness be felt." Several cases illustrative of the efficacy of this suppository are related by Dr. Williams.

### V. Cold Water or Nothing in Cholera.

It will have been seen by the two or three last memoranda that Dr. Lawrie cannot cure cholera by saline injections—that Mr. Buchanan cures it by stimulants and iodine—Mr. Bowden by bandaging the abdomen—Dr. Williams by an anodyne suppository. The next gentleman who appears upon our list, Mr. French, surgeon to the St. James's Cholera Hospital, whose pamphlet is now before us, hints that all such is *improper* treatment. "The patients to whom he gave internal stimulants died, and the application of heat to the whole surface he has found to increase the collapse!" Here we are brought to a pretty pass. One by one all our remedies have been cut away from under our feet, and even warmth to the surface and frictions, the hencoops to which we had clung in the sea of uncertainty into which we have been tossed, are found to bring destruction rather than safety! Mr. French protests against astringents and against calomel, because they have "not altered the mechanism of the disease." The means which Mr. French, for he is not very explicit, appears to recommend, are large draughts of cold water, or nothing at all. In the most severe case which he relates, at least the most severe of those in which recovery took place, the patient obstinately refused all medicine, and even drink, from the fear of its containing medicine. We know not whether this is meant as a sly hit at the numerous cures of cholera that appear in every journal, but whether it be so meant or not, it may certainly be so applied. Mr. French would suggest, that "the treatment be of that simple kind, which is not likely to interfere with the processes which he has assumed to be resorted to by Nature for the recovery of the patient." By all accounts, the French mode of treatment, we really intend no pun, is simple and expectante enough, yet we are not struck with any marvellous diminution of mortality thereby. However, Mr. French recommends cold water; and we shall feel delighted if cold water will do what its advocates anticipate. In order that Mr. French's practice may be clearly seen, we shall mention his treatment of two cases detailed by him. The first patient was a young girl. She was ordered mustard poultices to the spine and scrobiculus cordis—4 grains of common salt, 4 of carbonate of soda, and 2½ of nitre every hour, and 2 grains of calomel every hour also, with cold water ad libitum. Another case was treated by sinapisms to the spine and epigastrium, hot-water bottles to the feet, and cold water ad libitum.

---

### VI. NARCOTIC GLYSTERS IN CHOLERA.

These are recommended in the Medical Gazette, for July 21st, by Mr. Hallett, of Devonport. He proposes gtt. x. to xx. of tinct. opii, in ℥ij., ℥iij., or ℥iv. of water; or from one to two grains of crude opium in watery solution, thrown up by means of a small brass syringe, with an ivory pipe.

---

### VII. SUCCESS OF MURIATE OF SODA IN CHOLERA.

It becomes quite a pleasing task to peruse papers on cholera. The disease is stripped of its terrors, and every communication breathes nought but success. We wonder at the perversity of the Central Board, in publishing those infamously-incorrect lists of deaths and recoveries which meet our eyes in the newspapers. In the Times of Sept. 14th, we see, cured, 330—dead, 143. How is it possible that the proportion of deaths can be as high as stated, when almost every medical man has a successful method of his own. There is reprehensible error somewhere. However, when these excepta choleralogica are published, and gentlemen see how many successful plans of treatment there are, we do hope that the lists will exhibit a perceptible difference.

Dr. Pidduck's plan is to give the muriate of soda. We give his directions in his own words.

"After the ordinary preparations are made for favouring the restoration of heat to the extremities, and promoting a profuse perspiration, by putting the patient to bed in a flannel shirt next his skin, wrapping him up in hot blankets, covering him with plenty of bed-clothes, and applying large stone-ware bottles, filled with hot water, to his feet and sides, one ounce, or two tablespoonfuls of common salt, dissolved in eight ounces of warm water, is to be administered. The patient's head is then to be covered with the bed-clothes, and on *no account* is he to be permitted to rise. Half an ounce of salt, dissolved in four ounces of cold water, is to be given every hour till a copious vomiting and purging of bile is produced, and a profuse sweat breaks out. It has rarely been found necessary to repeat the salt more than three times. After the free evacuation of bile by vomiting and purging, the blueness of the skin gradually vanishes, and when the perspiration subsides, the tongue loses its cold, moist, livid character, and assumes a dry yellow, or brown appearance, with a red margin, and the patient *suffers from intense thirst*. He also frequently complains of acute pain in the ears, and of tenderness in the epigastric region.

For the relief of these symptoms, one or two drachms of oleum ricini, repeated every six hours, to carry off the vast accumulation of bile, the citrate of potass, with a few grains of carbonate of soda, in a state of effervescence, repeated every three hours, and the application of a few leeches behind the ears, or to the epigastrium, are generally sufficient. To allay the thirst, let him drink moderately of thin gruel, soda-water, or even cold water, which, instead of retarding, seems to promote free perspiration, and the abundant discharge of bile, upon which his safety depends, and the retention of which appears to be the cause of all the distressing and dangerous symptoms."

It will be observed that Dr. Pidduck successfully employs what Mr. French has found to increase the collapse; nay, what must be Mr. French's horror, at observing that Dr. Pidduck terms the means of "promoting a profuse perspiration *ordinary* preparations." We are only chroniclers of facts.

### VIII. Successful Prohibition of any Drink in Cholera.

Mr. Wilson, of New Street, Bishopsgate, is not a patron of cold water, nor of any water, nor of any fluid of any description whatever. In the Med. Gaz. for Aug. 11th, he relates four cases of cholera in which the patients were totally prohibited from drinking water, or any other fluid. Their suffering from thirst, he tells us, was dreadful, and it was grievous to hear one cry out feebly for *a drop of water—a drop of water.* Mr. W. is satisfied, "that had she been indulged in her wish, she would have died." Of this we are rather sceptical, and when we take a glance at our excerpta, we cannot but suspect that it signifies not much whether patients drink cold water or let it alone, saving and excepting the gratification or endurance of a tormenting thirst. This, however, is a speculation. As we have mentioned what Mr. Wilson will not let his patients have, we ought to mention what he will. In the first case, which was successful, his treatment consisted of all possible external stimulants—opiates in the first instance, with solutions of liquor potassæ in water—and subsequently mild aperients. The treatment appears, so far, to have been judiciously based on general principles, in the absence of any specific remedy.

### IX. Mortality from Cholera in London.

Dr. George Gregory is an exact man, and likes to see things stated arithmetically. We would venture, now, that he possesses the organ of numbers, albeit we know that he shews no love for the phrenological craft. Whether this be or be not true, certain it is that Dr. Gregory is a regular investigator of the Bills of Mortality, though we hope he does not contribute towards them. Dr. G. is not like the surgeon in Gil Blas, who, after running out at the back-door to stab a man, sallied forth from the front to offer his professional assistance. The result of Dr. Gregory's last inquiry, is contained in a short note to the Editor of the Medical Gazette.

"In the sixth volume of the London Medical Gazette (page 691), I submitted to your readers an analysis of the London Bills of Mortality for 1829; wherein it appeared that there died in the metropolis that year, of what are commonly called *bowel complaints* only forty-one persons—viz:

| | |
|---|---|
| Of Diarrhœa | 31 |
| Of Dysentery | 6 |
| Of Flux | 4 |
| Total in 1829 | 41 |

In the year 1830, the number of deaths by the same disorders are stated as follows:—

| | |
|---|---|
| By Diarrhœa | 19 |
| By Dysentery | 24 |
| By Flux | 10 |
| Total in 1830 | 53 |

The term *cholera morbus* appears in these bills, for the first time, on Tuesday, 21st December, 1830; and the first death by such a disease is recorded in the following week. The total number of deaths by bowel-complaints, in the year ending December 20th, 1831, is thus given:—

| | |
|---|---|
| By Cholera Morbus | 48 |
| By Diarrhœa | 33 |
| By Dysentery | 11 |
| Total in 1831 | 92 |

Of the total number of deaths by cholera morbus in the year 1831 (48), there took place,

| | | |
|---|---|---|
| Prior to August | | 12 |
| In the autumnal months, August | 10 | 33 |
| September | 13 | |
| October | 10 | |
| Subsequent to October | | 3 |
| Total in 1831 | | 48 |

The following table exhibits the deaths by cholera morbus in the first six months of the present year:—

| | |
|---|---|
| In January | 0 |
| February | 25 |
| March | 250 |
| April | 273 |
| May | 52 |
| June | 67 |
| Total in the first six months of 1832 | 667 |

The next table gives the deaths by cholera morbus in the month of July, as registered in the Bills of Mortality The result will probably prove as great a surprise to your readers as it has done to me.

| | |
|---|---|
| In the week ending July 3, 1832 | 55 |
| ——— 10 | 108 |
| ——— 17 | 158 |
| ——— 24 | 380 |
| ——— 31 | 305 |
| | 1006 |

The concluding table gives the total number of deaths by bowel complaints, in the first seven months of 1832:—

| | |
|---|---|
| By Cholera Morbus | 1673 |
| By Diarrhœa | 25 |
| By Dysentery | 12 |
| Total in the first seven months of 1832 | 1710 |

These statements afford, I think, a pretty satisfactory proof that the precautions of Government have not been unnecessary, and that the anxiety of the public mind has not overstepped the bounds of reason so far as some *philosophers* would induce us to think."

Dr. Gregory, like Mr. Cobbett, would seem to have a great dislike for the *feelosofers.* Why Dr. G. should be so hard on them we do not know, but the miserable men may exclaim, in the words of the song,

> "Perhaps it is right to dissemble your love
> But why should you—kick us down stairs?"

When Dr. Gregory thinks this a satisfactory proof that "the precautions of Government have not been unnecessary," we confess that we feel some misgivings as to *his* philosophy. According to his own shewing, there have been 1710 deaths from cholera in seven months, out of a population of at least a million and a half, and by far the greater part of those 1710, it must be remembered, taken from the poorest of the poor, and most debauched of the debauched. Precautionary measures, if they mean any thing, mean preventive measures, and it remains for Dr. Gregory to shew, that those preventive measures have effected any good, or made the mortality less than it would otherwise have been. The precautionary measures actually enforced were chiefly quarantine restrictions. Have *they* been of any service? have *they* kept the disease out? It laughed at them and their admirers. The precautionary measures, proved by 1710 deaths in London not to have been unnecessary, were totally inoperative, as all who have eyes in their heads must see, in preventing the rise or progress of cholera, but very operative indeed in inspiring panic, crushing commerce, and heaping additional poverty and distress on a pauperized population. It is true that, had the wishes of Dr. Gregory's party been carried into effect, *some other* precautionary measures would have been resorted to. Cordons of police and of soldiery would have been formed in this good city of London—parishes would have been declared infected, and cut off from communication with neighbouring parishes—a red cross would have been chalked upon our doors, and "Lord have mercy on us" might have been added, as in the time of the plague. All this, and more, would have been done by those who, perhaps properly, sneer at philosophers, and what would have been the consequence? Let the riot at Manchester tell.

In making these remarks we intend no personal affront to Dr. Gregory. He is too good-natured to care for a little sparring when he puts on the gloves. We should indeed be much to blame if we said any thing to hurt the feelings of a gentleman, whom his very enemies, if he has any, allow to bear a retort with the most perfect good humour. At the same time Dr. G. may suppose that nobody likes now-a-days to be called a *philosopher*.

---

### X. Salines not successful in Cholera.

In the same number of the Medical Gazette as that which contains Dr. Gregory's letter, we find a record of four cases from a public institution, (name not mentioned,) in which salines were exhibited without the least success. These were really severe cases, and, as we observed in our review of Dr. Stevens' book, the patients might as well have had so much ditch-water. The gentleman who relates the cases observes, " the failure of the above treatment in these cases caused us to relinquish the plan altogether"—a very sensible resolution. As we hate to read of unsuccessful treatment, we shall pass to some other plans.

---

### XI. Dr. Lindsey's Opinions on the Treatment of Cholera.

Really, so far as we have gone, we think Dr. Lindsey's brief observations on the treatment of cholera by far the most practical and sensible of any we have had occasion to notice. To clear our way and shew what he does not recommend, we must observe that " the saline treatment has been fully tried at his cholera hospital, and found unsuccessful"—that saline injections into the veins have been tried in two cases, and failed—that hot water has been tried and failed. Dr. Lindsey does not seem to have tried cold water, he would probably have placed it in the same category. Most of the cases sent to the hospital are in a state of collapse. Dr. Lindsey employs stimulant emetics in the first instance, with sinapisms to the epigastrium, and hot salt to the feet and back, followed by the *cautious* use of the lancet, and afterwards calomel in moderate doses. This treatment is adapted to cases in their commencement, that is, in the commencement of grave symptoms.

" In the cases of collapse the indication seems to be to give an impulse to the arterial system, which I conceive is best effected by producing energetic vomiting. With this view the mustard emetic is given in all cases where re-action has not been already established; and at the same time the external stimulants mentioned above are employed. After the operation of the emetic, the carbonate of ammonia in solution is given in doses of a scruple, at the intervals of a quarter or half an hour, or an hour, according to the intensity of the collapse. Calomel is now used in ten grain doses, the *first* being *sometimes* combined with a grain, or a grain and a half, of opium. This plan is followed up by three grain doses of calomel every second hour, which are persevered in until biliary evacuations are established. Should the ammonia not be rejected, it is continued until re-action takes place, and then omitted. Great circumspection is required in the use of this stimulant, as, if too long persevered in, it will induce cerebral congestion."

When re-action is violent, general and local bleeding should be used according to circumstances—for great irritability of the stomach, leeches to the epigastrium, warm fomentations, a blister, effervescing draughts with opium, or mustard emetic. Opium is not used in large quantities from its tendency to increase determination to the head. The drink of the patient *should be regulated by his feelings*, and may be bottled ale, cider, soda water, wine, brandy and water, or spring water. Dr. L. speaks highly of ale.

---

### XII. Treatment of Cholera at Leeds.

This is described in a letter from Mr. Birtwhistle in the Lancet for Aug. 25. Mr. B. states that saline injections have been tried, found to fail, and abandoned. Mr. Morley, resident surgeon of the Hospital in Marsh-lane, Leeds, used the salines, after Stevens' fashion, but had not seen enough to determine their value. "This was prior to the exposure of the cases in Cold-bath-fields prison." The treatment actually pursued was nearly the same in Leeds and York.

"When a patient was admitted in the collapsed stage, tins containing hot water, and suitably shaped, were applied to the abdomen, back, and feet, the body being previously surrounded with a blanket. These retain the heat for a long time, and had been found to answer better than any kind of bath in restoring the natural temperature. Should the prostration of strength be extreme, reaction was assisted by the internal administration of strong stimulants, consisting chiefly of ammonia and camphor. A scruple of calomel, uncombined with any other medicine, was then given every hour, until the severity of the symptoms had abated. The quantity was then lessened, and the interval between each dose increased. Six or eight doses had been generally sufficient to arrest the vomiting and purging, and to excite the biliary secretion. It had rarely been necessary to push the mercury to salivation, although I think it would be advisable gently to touch the mouth, with the view of lessening the consecutive fever."

At the York cholera hospital re-action was promoted by the enema of spirits of turpentine, in a mucilaginous mixture, six ounces being thrown up in extreme cases. We have seen one patient recover from the stage of collapse who took turpentine per os et per anum. Drs. Goldie and Anderson speak favourably of the medicine. Mr. Teale avoids much opium on account of the secondary affection of the head. The majority of the medical men in Leeds and York are non-contagionists.

### XIII. Cholera cured by Calomel and Opium.

Mr. Dobson, a very intelligent surgeon of Pimlico, has related a case of this description. The patient was almost in extremis when he saw her. Heat was applied to the surface and the whole body rubbed constantly with warm turpentine. Large quantities of brandy were given, and cal. ℈i. with opii, gr. ij. every half hour. Besides this an enema of starch and tinct opii, ℥ss. was administered. As the patient rallied the doses of calomel and opium were diminished. She recovered perfectly.

---

### XIV. A Logical Deduction.

Dr. Weatherill, of Liverpool, injected into the veins of a man labouring under cholera, four hundred and eighty ounces of saline fluid. Strange to say, the man recovered. However, Dr. Weatherill declares that his own opinion of saline injections, *from this single case*, "is exalted, and he should not think he did his duty if ever again he *suffered* a patient to die of cholera, without using it." He sums up thus;—"any medical man who does not (use injections) will excuse me for passing severe judgment on his conduct." This is modest with a vengeance. So every medical man is to prostrate his judgement, his reason, his experience before Dr. Weatherill, or excuse him for censuring him severely, if he does not. It matters not that others may reason differently, or have a larger experience in this mode of treatment; they must employ injections at the pain of Dr. Weatherill's displeasure.

---

### XV. Cold Water in Cholera.

This, at the time we are writing, Sept. 10th, is the last plan recommended for the treatment of cholera. It is contained in a communication from Dr. Hardwicke Shute to the Board of Health, and has been transmitted by the Board to the Lancet and Medical Gazette. The quantity of cold water taken "in the most marked cases of recovery," was as much as some gallons in a few hours. Dr. Shute adds to this the abstraction of all kinds of stimulus external and internal, even to the exclusion of friction or of the application of heat in any form. Dr. Shute does not shuffle—he says broadly and boldly that the treatment is to apply to the second and particularly to the third stage of cholera, " when the pulse at the wrist has ceased, or become nearly imperceptible." This is manly and honourable. Dr. Shute does not say, like Dr. Stevens, that the premonitory diarrhœa is not curable by ordinary means, and then laud his method for curing it. All is fair and above-board with Dr. Shute ;—" if my remedy does not succeed in the really bad cases, reject it." Dr. Shute not only applies cold internally but to a certain degree externally also. The following is this able and benevolent physician's practice.

" The windows of the apartments, at the Cholera Hospital in Gloucester, are large and numerous in proportion to the size of the room, and the door which opens immediately into the garden, is seldom shut. The windows are open day and night, so that the patient may be considered as living in the open air, and the fire is kept so low as not to influence the temperature of the room. The covering of the patient is confined to a light blanket or rug ; and it seldom happens that some part of the patient, particularly the breast and shoulders, is not constantly exposed. Under these circumstances, a pint of cold water is offered to the patient, and very frequently two-thirds of this are taken at a draught. In what I consider the most favourable cases, vomiting is almost immediately produced, and the patient in two or three minutes again calls for and eagerly drinks the same quantity, with the same results. This is often continued for hours, until gallons of water have been taken, and the greatest proportion, but I conceive not all, is rejected. In other cases the patient is too insensible to ask for water ; and under these circumstances, it is offered every ten minutes or quarter of an hour, and most commonly drunk with avidity. If gruel or tea be offered, the patient most frequently refuses it ; and, generally speaking, no kind of nutriment is taken in any form until the period of convalescence. In the first six or eight hours no amendment can be observed. In the next six or eight hours there is some diminution of intensity in the purple hue of the extremities. In the next six or eight hours there is a manifest improvement in the countenance of the patient, and increased disposition to sleep. In some cases the pulse has not been perceptible for twenty-four or thirty-six hours. From this period the pulse, the animal heat, and the secretions, are very gradually restored ; and at the end of forty-eight hours, on the third day from the commencement of the plan of treatment proposed, the patient is convalescent ; and in all cases without consecutive fever. I mention these circumstances particularly, in order that the practitioner may not be impatient ; he should make not the least alteration in the plan laid down, as long as the patient is merely not getting worse."

Dr. Shute observes that he has tried this plan in twelve consecutive cases with success. It is not consistent with our plan to indulge in elaborate criticisms in these excerpta. Our object is rather to collect facts. When we look back at the various methods that have been proposed, considered by their proposers successful, and on trial abandoned, can our readers blame us at expressing scepticism ; will they not become sceptics themselves ? When we see the most clashing theories and most opposite plans of treatment equally enthusiastically advocated, we cannot but suspect, that all are equally or nearly equally inefficacious. Amidst all this glitter of success the general lists shew no diminution of relative mortality, on the contrary they exhibit an increase. When the epidemic first appeared in this country, one method of treament was pretty generally pursued. Latterly we have had experiment on experiment hot water and cold water—bleeding and —stimulants—calomel and opium and salines — frictions and no frictions—drink ad libitum, and no drink at all.

Here is confusion worse confounded, and we scarcely hesitate to say, that in point of principles of treatment and practice, we are worse off now than we were twelve months ago. The cold water treatment is the last, and sanguine persons may believe that it is the best: unfortunately we are not sanguine.

In a disease like the present we would not discountenance rational and cautious experiments, far from it. But we would discountenance and reprobate most strongly premature declarations of success, and dogmatic abuse of all who do not yield implicit faith to what is written or said. Look, for instance, at the manner in which many of the advocates of the saline treatment have attacked and are attacking those who have opposed or have not assented to their views. Can any thing be more disgraceful? It is certain that truth will in the end prevail, and fallacy be as signally exposed. Dr. Shute behaves like a man of science and candour; all he expects his statements to lead to, is "a more extensive trial of the plan proposed." This is as it should be.

---

### XVI. M.M. FOVILLE AND PARCHAPPE ON THE CHOLERA AT ROUEN.

These gentlemen have written a sensible and interesting pamphlet. We cannot of course give even an abstract of it, but may mention one or two facts related by the authors. They had the charge of the cholera hospital at Rouen. The epidemic broke out in Rouen early in April, 1832, but owing to popular prejudices, no patients were received in the establishment until the 13th. This establishment appears to have been an asylum for the insane, situate on the left bank of the Seine in an apparently healthy situation. The total number of cholera patients was 78, of whom 38 died, 37 recovered, and three left the house before the issue was determined. There is another fact deserving of great attention, but too often lost sight of by those who treat, and those who weigh the value of the treatment of, cholera. The disease lasted 32 days in the asylum. In the first eight days there were 36 patients, and 23 deaths—in the succeeding 16 days there were 31 patients and 15 deaths—and in the last eight days 11 patients and no death. Here we see the rate of mortality progressively diminishing, from the first quarter of the epidemic's duration. Had Dr. Stevens arrived with his salines during the last quarter, when 11 cases occurred without a death, what a buz would have been made! The French reporters, however, are too candid and too sagacious not to see that the diminution of deaths was rather owing to the laws of the disease itself, than to the influence of treatment.

The reporters draw attention to another subject, the propagation of the disease within the asylum. The cholera wards were on the ground floor of the centre of the building; the upper stories being inhabited by the insane. Of the latter, 26 were attacked with cholera, 25 of whom inhabited the part of the building appropriated to the cholera patients. It was also found that most were seized in the dormitories nearest the cholera-wards, and, with one exception, the farther these were removed from each other the more rare were the attacks. Amongst the employés of the asylum only one was attacked — the cook. He lived in the centre of the building. Of thirty-three *religieuses* who attended the sick, ten were attacked and three died.

From these considerations the reporters think they are justified in pronouncing, that "under the influence of the epidemic constitution that gives rise to cholera, persons inhabiting the vicinity of a collection of cholera patients are more likely to contract the disease than those removed from it."

We think this a fair expression of the facts of the individual case, and were the same thing generally noticed, it would also be a fair enunciation of a general law. But this has not been generally observed, at least there are very many recorded instances in which it was not; a circumstance which will tend to diminish the general applicability of the principle. At the same time we think that facts so well authenticated and so candidly stated should make men hesitate before they subscribed to the doctrine of absolute non-contagion.

In their resumé the reporters observe that the cause of cholera is yet a problem—that its seat is essentially in the digestive apparatus—that certain organic alterations constantly exist, and that their seat is in the same apparatus —that these alterations consist, first of a considerable defluxion towards the mucous membrane, and development of the glands of Brunner and Peyer, with extreme secretion of a particular fluid, followed by various degrees of inflammation of the membrane and of the follicles—that these alterations and the symptoms proceed pari passu—that in the absence of a specific, hitherto undiscovered, we must be guided by general principles and by the facts already mentioned—and that the true method of treatment should therefore be antiphlogistic, though not actively so.

We might proceed farther with these Excerpta, we might advert to contagion and to other topics, but we will desist. All parties should be anxious to apply the most successful treatment. We have therefore received with much pleasure the following circulars addressed by the Board of Health to all medical men who have the charge of patients affected with cholera.

(No. 1.) *Council Office, Whitehall, 3d Sept.* 1832.

Sir,—The Central Board of Health being anxious to obtain, from authentic practical Sources, short Outlines of the different Plans of Treatment in Cholera which may have been considered most successful; I am directed to request that you will have the kindness to submit the enclosed (No. 2.) to the Medical Members of your Board, and to any other Medical Gentlemen in the Neighbourhood who may have had extensive Practice in the Disease.

You will also request the Medical Gentlemen in charge of Cholera Hospital to fill up, and forward through you, a Return of the following Form, for each of these Establishments within the District under the Superintendence of your Board.

I am, Sir,
Your most obedient Servant,

To the Secretary of the
Board of Health of

Return of Patients admitted into the Cholera Hospital at ________
From ________ to ________ 1832.

| Total Numbers. | | | |
|---|---|---|---|
| Admitted. | Died. | Recov. | Remarks. |
| | | | |

(Signed)

(No. 2.) *Council Office, 3d Sept.* 1832.

Medical Gentlemen, who have had Experience in the Treatment of Cholera, and are of opinion that they have been successful in their Practice, are requested to forward to the "Secretary of the Central Board of Health," under Cover to "The Clerk of the Council in waiting, Whitehall," a short Account of their respective Methods of Treatment of the Epidemic:

1st. When in the Form of Bilious Diarrhœa.

2d. In that of Rice-water Evacuations.

3d. In the Stage of Collapse.

We are convinced that if the Board follow up this plan—if they carefully procure and make known to the profession the results of treatment—if they issue short and simple directions to the public, especially the poor, as to what they should and what they should not avoid—if they calm terror and distrust by ceasing even to hint at restrictive measures which, if carried into effect, could do no good, but which really cannot be carried into effect at all—if they religiously abstain from violating the ties of kindred and feelings of affection by cruelly sanctioning the dragging away of the bodies of the dead, or by encouraging the mummery of separate burial places—if they rather invite than compel the transportation of the sick to cholera hospitals—if they treat with urbanity the representation of those who differ from them in opinion, and do not dogmatise when they fail to convince—if they do this, we say, they will deserve and gain a popularity which they have not yet enjoyed, and will contribute to establish a kindlier feeling between the parties who have differed so widely and even so bitterly on cholera.

---

### XLIX.

### MENSTRUATION IN DIFFERENT COUNTRIES.

IN Part X. of "The Principles and Practice of Obstetric Medicine," lately published, Dr. Davis considers the anatomy of the uterus and its appendages, and enters on the subject of menstruation. From the remarks on this function we are tempted to select those on the differences observed in different nations and climates. It is generally known that the period of puberty is earlier attained in the warm regions of the South, and in "the lands of the sun," than in the chilling North, or even than in temperate climes. But Dr. Davis has collected some facts and illustrations, not quite so familiar as the general doctrine.

"In the more fervid climates of Asia and Africa, the women arrive at puberty at ten years of age, and not unfrequently at nine. Some years ago an English gentleman resident at Malta, who there occupied a distinguished post under the Government, favoured the author with the fact, that the native girls of that island sometimes marry before they attain even the latter age, and generally within a year or two afterwards. It is a matter of history, that the celebrated prophet of the Moslem faith consummated his marriage with one of his wives 'when she was full eight years old.' 'And now Khadija his wife being dead, after she had lived twenty-two years with him, to strengthen himself the more, he took two other wives in her stead, Ayesha the daughter of Abu Beker, and Sewda the daughter of Zama.'——'Ayesha was then not six years old, and therefore he did not take her into his bed till two years after, when she was full eight years old. For it is usual in those hot countries as it is all India over, which is in the same clime with Arabia, for women to be ripe for marriage at that age, and also to bear children the year following.' Prideaux's Life of Mahomet, p. 30, 1718. We are on the other hand informed that in Sweden, Norway, and a great part of Russia, menstruation does not often take place till the more stayed ages of seventeen or eighteen. In reference to this latter fact it might seem open at least to a presumption that so much absence of alacrity to engage in a function so essential to reproduction, as in the sequel we shall find menstruation to be, might have an unfavourable effect upon the population of the countries of the north. The facts of history are, however, directly opposed to such a conclusion; and the explanation seems to be, that in those countries, the duration of the function comprehends a more extended series of years; that the women are strong and well constituted; and that they therefore are competent to menstruate more regularly and during a longer portion of their lives than the women of the south: whence it results that in the end they are found more prolific, and that under favourable circumstances as to the means of living, they become the parents of a healthier and more vigorous offspring. We are accordingly informed by Rudbeck and other writers, that the Swedes have usually families of ten or twelve children, AND NOT VERY RARELY a progeny equal to more than twice those numbers. It should not therefore seem surprising that the population of those countries, in other respects so unproductive, was become so superabundant during the Goth and Vandal times as to have been competent to furnish entire armies of adventurers, first to conquer and finally to colonise distant and more favoured regions. But let us reverse the picture. M. Virey, in his article on climate, in le Dict. des Sc. Medic. observes that the burning climates of the more southern countries of Europe are well known to produce 'strongly-marked nervous temperaments and great precociousness of puberty. Hence,' he adds, 'the women of those countries have scarcely grown out of their infancy before they become mothers. But, mark the consequence: just like those perishing flowers of a day which the ardour of a summer's sun causes to open in the morning, and in the evening to wither and die, they soon lose their fecundity, and pass rapidly from the morning of their lives towards its decline and close.' This fact is well understood by the English inhabitants of India, who all send their children to England, in order to avoid the known effects of the climate of India in the production of precocious developments; but developments too premature to be permanently compatible with vigorous health, and almost certainly inductive of an early death.'

Again the quantity of the menstrual secretion is, as might perhaps be anticipated, materially affected by the climate and habits of life.

"With respect to the influence of climate in this matter, we meet with no great discrepancies of opinion amongst practical writers; it being something more perhaps than a very general presumption that women menstruate less abundantly in hot than in more temperate regions, and somewhat more abundantly in the latter than in the coldest countries of the north. This difference is attributed to the balancing influence of the cutaneous function of perspiration. 'In hot climates,' says Buffon, 'where the TRANSPIRATION is greater than in cold countries, the catamenial secretion is less.' Hist. Nat. de l'Homme, tom. iv. p. 491. 'In general,' Maigrier observes, Dict. Medicale, tom. xxxii. p. 386, 'the women of the south have their periods less abundantly than those of the north; but we may remark that those who live under the equator, as well as those who inhabit the most northern countries of the globe, have scarcely any traces of them. With respect to the former, their fluids are so volatilized by the excessive heat of their climate as to become insufficient to furnish the means of a menstrual secretion; and as for the latter, the rigour of their cold is such as, by constringing all their natural filters, to produce similar results.' The author is very doubtful whether M. Maigrier, in respect to the total absence of the menstrual function in the extreme northern latitudes, has not considerably overstated his case. Women of nervous and irritable temperaments, otherwise sometimes called bilious and melancholic, menstruate abundantly, sometimes profusely; whilst, on the contrary, with those of robust and well-steadied constitutions the secretion usually appears in sparing quantity, as also in the cases of persons of feeble and cachectic health who begin to menstruate late, and who perform the function difficultly and painfully. Females who live in towns, and who have frequent opportunities of engaging in the public pleasures of gay and fashionable society, exposed to all the temptations incident to the possession or accessibleness of whatever means may be calculated to exalt the imagination, to inflame the passions, and, by excessive or too frequent indulgences, to abuse the appetites, are in most cases the subjects of precocious, profuse, and morbidly irregular menstrual discharges. On the rear of the same class of females, and therefore subject to the same evil results, are the idle, the unfortunates, and the dissipated in all ranks of society. 'All the arts,' says Gardien, 'such as music and painting, including that of design, excite vividly the imagination. Music especially, cultivated too exclusively and at too early a period, develops an extreme sensibility. It was to an imprudence of this kind that we have heard attributed the death, on the approach of puberty, of both the daughters of Grétry, the celebrated musician.' Women of naturally strong passions, all other things being equal, are said to menstruate more abundantly than those of colder temperament, and such are indifferent to the privileges of connubial life. The evacuation is moreover less abundant with the average of women who live in the country than with a large proportion, as we have already seen, of such as inhabit great cities and towns, not only because the atmosphere of the country is more uncontaminated and salubrious, but because also its inhabitants are usually more regularly and usefully employed, and exempt from the profligate vices of great societies. The produce of the catamenial function becomes ordinarily less abundant with advancing life; and the same thing is true, but on a smaller proportional scale, in the instance of some mothers of numerous offsprings. A first gestation has sometimes improved the character and habits of the function; but there is reason to fear that such an advantage is not generally to be calculated upon. On the contrary, it may be admitted as an axiom founded on extensive observation of the phenomena of the function, that if it be performed imperfectly, painfully, or otherwise irregularly, during the first few years of the menstruating part of a woman's life, it will continue to be so performed throughout the remainder of it."

We have already, and on more than one occasion, expressed our approbation of this work of Dr. Davis's, and have taken the liberty of pointing out what we consider to be faults. Let Dr. Davis endeavour to compress much matter into little space, not to swell little matter into monstrous bulk—let him make his style, what it is not, simple, clear, and concise—and for Heaven's sake let him curtail those prolix, though valuable cases, that, in some former fasciculi, have swallowed, like Aaron's rod, all rival matter. If he follows this advice, he will render his work creditable to himself, and really useful to the profession.

---

### L.

### CHOLERA.

THE Editor of this Journal, in a tour through England and Scotland, has had his attention a good deal directed to the prevailing epidemic. He was very much struck with the influence of locality in the production or extension of this mysterious malady. This influence, indeed, is so obvious, that any person, in the least conversant with medical topography, would almost immediately form a tolerably correct estimate of the extent of cholera in any town or village through which he passed. Thus, let us look at Edinburgh: The locality is very unfavourable to the production of malaria, and accordingly we find that, in the new town, there has scarcely been a case, and in the old town, the narrow wynds, where a local malaria is constantly generated, furnished nine-tenths of the cases. Upon the whole, Edinburgh escaped much more fortunately than Glasgow or Paisley, whose topography is very remarkably calculated for the production of malaria. Thus, in sailing up the Clyde, we are struck with the low and swampy grounds on the left bank of the river. The whole plain, indeed, between the Clyde and Paisley, is a flat alluvial soil, beneath the level of the river at spring-tides, and reminds one very much of the Scheldt, the Ganges, and other rivers, on whose banks the mysterious miasms of fever and various diseases are generated in such profusion. Liverpool, on arriving from the pure air of Cumberland and Westmoreland, assails the olfactories of the stranger in a very forcible manner. This second city of England is almost as malodorous as Rome or Naples in the dog-days! The aspect of the inhabitants, especially of the lower orders, is singularly unhealthy—and, altogether, one would predicate that this town would suffer severely from any epidemic connected with malaria.

Oxford is situated in a locality that is far from being an eligible one in this respect. The town is built on a flat, much lower than the surrounding country, and so intersected with canals, some fluent, others stagnant, that one would conclude that a colony of Dutchmen had settled here, and introduced the topographical features of Walcheren or Beveland into Oxfordshire. The cholera has here carried off 80 or 100 people. These are merely specimens, which correspond very closely with the general descriptions of those places where cholera has shewn its greatest malignity.

Let us look to some localities of a different description. Edinburgh has been instanced. The towns of Cheltenham, Leamington, Malvern, Warwick, Buxton, Matlock, &c. have remained totally free from cholera, and no places could present a greater contrast in medical topography, to those places already enumerated as malarious, than these last-mentioned towns. Cholera was brought, indeed, to Malvern, but the disease died with those who brought it there. It will be very surprising if Malvern suffers to any considerable extent. It is the very reverse of Gloucester, where the malady has committed considerable ravages.

Although cholera has now assailed a certain number of the respectable classes of society, both in town and country, yet, upon minute enquiry, there has generally been some local cause, or some error in diet, to excite the disease into activity. But when we consider how powerful the miasm of cholera has been in some places, we cannot wonder that the malady has assailed men in all ranks of society.

The eccentric manner in which the epidemic has pursued its course, or rather started from time to time into existence, is as remarkable in this as in other countries. Thus lunatic asylums were supposed to exempt their unfortunate inmates from the contagion of cholera; but this has proved an error. At the Bethnal Green Lunatic Establishment, and also at the "White House," upwards of an hundred cases have occurred, and proved very satisfactorily the spontaneous origin of cholera under a general epidemic influence. The two establishments mentioned, though adjoining, are completely separate, as to officers, attendants, &c. The first case that occurred in the "Red House" was a woman who had been long confined within the walls, and where all trace of contagion was impossible to be discovered. The medical superintendant of the "White House" appears to have taken "prudent precautions," by cutting off communication with the *red* mansion, and even the windows that looked towards the infected district were blocked up! But yet the cholera got into the White House. None of the medical men, nurses, burier of the dead, however, contracted the disease. It is curious that, during severe attacks of the epidemic among the lunatics, reason was restored—at least so our contemporary, the Medical and Surgical Journal, avers.

We may state here, on the authority of Sir William Burnett, that cholera suddenly broke out in the lunatic wing of Haslar Hospital, without any source of contagion, and as suddenly stopped in the midst of its progress, without being communicated to nurses, attendants, or others in the hospital.

The result of our observations, both in England and Scotland, confirms the fact that diarrhœa is the precursor, or rather the first stage of the epidemic. In nineteen out of twenty cases this is the beginning and end of the disease—and where early attended to, the complaint is easily managed. When collapse is allowed to occur, the great majority will die under any mode of treatment, for the fluid parts of the blood are drained off, and organic lesions take place in the brain and nervous system, which render a fever indispensable, and too often fatal.

In the course of our observations and enquiries, we have found that cold drink given almost ad libitum, and small doses of calomel given at very short intervals, have been the most effectual treatment in arresting the vomiting and determining the blood to the surface. This is no new practice, either in this or in other countries. Aræteus and many of the ancients have recommended cold drink—and at Vienna, many months ago, the practice was carried to a considerable extent, and, it is said, with much success.—*See Dr. Gilkrest's Letter in the Times.*

Before concluding these hasty notices we may remark, that Dr. Mackintosh of Edinburgh, appears to have carried on his pathological investigations in the cholera hospital there with unprecedented zeal and talent. We have had an opportunity of inspecting that gentleman's pathological museum, and we hope Dr. M. will favour the profession with the results of his indefatigable labours. The changes of structure observed after death are most extraordinary. The par vagum, in particular, has been found to present remarkable lesions, its neurilema being injected, and its substance bulged out into ganglia. The brain, the spinal marrow, the heart, the arteries, the internal coats of the intestines, have all presented phenomena of the most marked and peculiar character, which are multiplied and illustrated by the most beautiful preparations in Dr. Mackintosh's museum. We will not attempt to anticipate the well-earned fruits of that gentleman's labours, as we have no doubt he will soon make them available to the world.

---

### LI.

### MR. SCHLOSS'S ANATOMICAL MODELS.

WE have, on former occasions, spoken highly of the wax models of healthy and of morbid structures imported by Mr. Schloss, the German bookseller in St. Martin's Lane. We are glad to perceive that Mr. S. meets with some encouragement, and we should feel pleased if he met with more. Such models are unavoidably too expensive for private individuals to purchase, but they certainly should form an integral portion of the cabinets of medical schools and of medical institutions. We have no hesitation in saying that they are indispensably requisite for both. Mr. Schloss has induced the Lords of the Treasury to remit the duty on such anatomical models as are imported for public establishments, and they may, consequently, be now obtained at a reduction of 25 per cent, an important consideration. We have just inspected some models recently arrived; they are as follows.

1. Fæminæ Hottentottæ partes obscenæ, cum membranaceo illo rimæ tegumento longè famoso. In museo Regis Kratislaviensi servantur et ab artifice diligentissimè cerâ expressæ sunt. Hujus speciminis descriptionem invenies in Wmi. Otto libro.

2. Ligamenta glottidis.

3. Nervi superficiales ac arteriæ faciei et cervicis.

Mr. Schloss also shortly expects models of fungus hæmatodes of the liver and testis, and of osteo-sarcoma of the lower jaw.

With respect to the models which we have seen, we can only say that they are beautifully and accurately executed; that of the nerves of the face is almost above praise. Those who have had any dealings with Mr. Schloss must feel that his politeness and attention furnish an additional claim on their consideration, and we must say that we think this enterprising foreigner worthy of British patronage.

---

### LII.

### DR. ARNOTT'S HYDROSTATIC BED.

WE had seen this bed in use in St. George's Hospital, and were curious to find a description of it, when we lighted on one in the Lancet, for Sept. 15th of the present year. The account is said to be abridged from the 5th edition of Dr. Arnott's Elements of Physics, now in the press. We hasten to present it abridged, or rather curtailed, still further to our readers. We need not inform them how difficult it is, in many cases of long illness, to prevent the formation of sloughs on the hips or nates, in consequence of the pressure to which they are subjected, nor that, when such sloughs are formed, they too frequently contribute to the patient's destruction. In such a case, where Earle's bed and other means had failed in preventing, and, when established, in checking such sores, Dr. Arnott conceived the ingenious idea of applying water to the purposes of a bed.

"Under these circumstances, the idea of the hydrostatic bed occurred to me. Even the pressure of an air pillow had killed her flesh, and it was evident that persons in such a condition could not be saved unless they could be supported without sensible inequality of pressure. I then reflected, that the support of water to a floating body is so uniformly diffused, that every thousandth of an inch of the inferior surface has, as it were, its own separate liquid pillar, and no one part bears the load of its neighbour, and that the patient might be laid upon the surface of a bath, over which a large sheet of the waterproof India-rubber cloth was previously thrown, she being rendered sufficiently buoyant by a soft mattress placed beneath her. Such a bed was immediately made. A long trough, a foot deep, was lined with metal, to make it watertight; it was about half-filled with water, and over it was thrown a sheet of the India-rubber cloth. Of this sheet the edges (touched with varnish, to prevent the water creeping round by capillary attraction) were afterwards secured in a water-tight manner all round to the top of the trough, the only entrance left being through an opening at one corner, which could be perfectly closed. Upon this dry sheet a suitable mattress was laid, constituting a bed ready to receive its pillow and bedclothes, and not distinguishable from a common bed but by its surpassing softness. The patient was laid upon it; she was instantly relieved; sweet sleep came to her; she awoke refreshed; she passed the next night much better than usual; and on the following day all the sores had assumed a healthy appearance; the healing from that time went on rapidly, and no new sloughs were formed.

The down pillows were needed no more.

In choosing the mattress, if unusual positions are required, by having different thicknesses in different parts, or by placing a bulk of folded blanket or of pillow over or under the mattress in certain situations, any desired position of the body may be easily obtained. If the water be about six inches deep, which in general will suffice, the person standing upon any part of the bed, or sitting with the knees raised, will cause the part of the mattress on which he rests, gently to touch the bottom. On lying down, he as completely floats as if the Atlantic were under him.

This bed is a warm bed, owing to water being nearly an absolute non-conductor of heat from above downwards, and owing to its allowing no passage of cold air from below. From this last-mentioned fact, however, less of the perspiration, sensible and insensible, will be carried off by the air than in a common bed, and unless the patient can rise or be lifted daily, to allow the bed to be aired like a common bed, there will be a necessity for using some such means as the following, to prevent the condensation of perspiration on the water sheet below—an oiled silk laid over the mattress, or a blanket, to be occasionally changed, laid under it, or a set of flexible tubes of spiral wire laid under it, with their ends open to the atmosphere, to ensure a constant ventilation of the mattress; or, similarly placed, and producing the same effect, a layer of cork cut into square pieces, with spaces left between them, to serve as conduits of air. This bed in itself is as dry as a bed can be, for the India-rubber cloth (of which bottles can be made) is quite impermeable to water, and the maker is now preparing cloth expressly for this purpose. Unlike any other bed, it allows the patient to change his position almost like a person swimming, and so affording the kind of relief which, in constrained positions, is obtained by occasional stretching. It exceedingly facilitates turning for the purpose of dressing wounds, for by raising one side of the mattress, or depressing the other, or merely by the patient's extending a limb to one side, he is gently rolled over, nearly as if he were simply suspended in water; and it is possible even to dress wounds, apply poultices, or place vessels under any part of the body, without moving the body at all; for there are some inches of yielding water under the body, and the elastic mattress may at any part be pushed down, leaving a vacant space there, without the support being lessened for the other parts. It may be made so cheaply, that even in hospitals, where economy must prevail, it may at once be adopted for many of the bedridden. The bed has been introduced into St. Bartholomew's and St. George's Hospitals, and elsewhere. With it sloughing need never occur again, and by alleviating distress through the earlier stages of disease, it may prevent many cases from even reaching a degree of danger. It is peculiarly applicable to cases of fractured bones, and other surgical injuries; to palsies, diseases of the hip-joint, and spine; and universally where persons are obliged to pass much time in bed. Also in all cases of curvature of the spine, existing or threatened. If used without the mattress, it becomes a warm or a cold bath, not allowing the body however to be touched by the water; and in India it might be made a cool bed for persons sick or sound, during the heats which there prevent sleep and endanger health."

Dr. Arnott has very properly commissioned no person in particular to manufacture it.

---

### LIII.

### THE ANATOMY ACT.

On the 1st of August the ACT FOR REGULATING SCHOOLS OF ANATOMY became the law of the land. The passing of such an Act is a sort of era in medicine, and one illustration, among many, of the growing intelligence of legislators. It is now apparent that what is just and reasonable in principle, will, if properly and patiently urged, receive the attention and ultimately the sanction of those who legislate in England. On these accounts we hail the Act as a boon, no, not a boon, but a tribute to the majesty of truth, a concession to the interests of science, a triumph to intellect and civilization. We look not to the wording of the Act, we look not to its details, but we look to its general tenor, and we find in those who framed it a sincere desire and an anxious attempt to promote the true interests of the public, and the advancement of the medical profession. This is due to Mr. Warburton, and we freely offer our tribute of praise to the gentleman by whose exertions the Bill was introduced, and to the Government by whose liberality it was carried.

But in making this acknowledgment, we do not surrender our private judgment, nor lose the right of expressing our individual opinions. We do think the Bill defective, and although we cannot pretend to foretell in what manner it will work, we confess that we entertain misgivings as to the result. We are willing to admit that great difficulties have presented themselves to the supporters of the measure, and it may be true, though we do not ourselves believe it, that a more efficient one could not have been carried. Before we make any farther remarks, we insert the whole of the Act, as contained in the weekly medical journals, together with the circulars issued by Dr. Somerville, who has been appointed to the office of inspector.

### AN ACT FOR REGULATING SCHOOLS OF ANATOMY.

[1*st August*, 1832.]

WHEREAS a knowledge of the causes and nature of sundry diseases which affect the body, and of the best methods of treating and curing such diseases, and of healing and repairing divers wounds and injuries to which the human frame is liable, cannot be acquired without the aid of anatomical examination: And whereas the legal supply of human bodies for such anatomical examination is insufficient fully to provide the means of such knowledge: And whereas, in order further to supply human bodies for such purposes, divers great and grievous crimes have been committed, and lately murder, for the single object of selling for such purposes the bodies of the persons so murdered: And whereas therefore it is highly expedient to give protection, under certain regulations, to the study and practice of anatomy, and to prevent, as far as may be, such great and grievous crimes and murder as aforesaid; be it therefore enacted, by the King's most Excellent Majesty, by and with the advice and consent of the Lords Spiritual and Temporal, and Commons, in this present Parliament assembled, and by the authority of the same, That it shall be lawful for his Majesty's Principal Secretary of State for the time being for the Home Department in that part of the United Kingdom called Great Britain, and for the Chief Secretary for Ireland in that part of the United Kingdom called Ireland, immediately on the passing of this Act, or so soon thereafter as may be required, to grant a licence to practise anatomy to any fellow or member of any college of physicians or surgeons, or to any graduate or licentiate in medicine, or to any person lawfully qualified to practice medicine in any part of the United Kingdom, or to any professor or teacher of anatomy, medicine, or surgery, or to any student attending any school of anatomy, on application from such party for such purposes, countersigned by two of his Majesty's justices of the peace acting for the county, city, borough, or place wherein such party resides, certifying that, to their knowledge or belief, such party so applying is about to carry on the practice of anatomy.

II. And be it enacted, That it shall be lawful for his Majesty's said Principal Secretary of State, or Chief Secretary, as the case may be, immediately on the passing of this Act, or as soon thereafter as may be necessary, to appoint respectively not fewer than three persons to be inspectors of places where anatomy is carried on, and at any time after such first appointment to appoint, if they shall see fit, one or more person or persons to be an inspector or inspectors as aforesaid; and every such inspector shall continue in office for one year, or until he be removed by the said Secretary of State or Chief Secretary, as the case may be, or until some other person shall be appointed in his place; and as often as any inspector appointed as aforesaid shall die, or shall be removed from his said office, or shall refuse or become unable to act, it shall be lawful for the said Secretary of State or Chief Secretary, as the case may be, to appoint another person to be inspector in his room.

III. And be it enacted, That it shall be lawful for the said Secretary of State or Chief Secretary, as the case may be, to direct what district of town or country, or of both, and what places where anatomy is carried on, situate within such district, every such inspector shall be appointed to superintend, and in what manner every such inspector shall transact the duties of his office.

IV. And be it enacted, That every inspector to be appointed by virtue of this Act shall make a quarterly return to the said Secretary of State or Chief Secretary, as the case may be, of every deceased person's body that during the preceding quarter has been removed for anatomical examination to every separate place in his district where anatomy is carried on, distinguishing the sex, and as far as is known at the time, the name and age of each person whose body was so removed as aforesaid.

V. And be it enacted, That it shall be lawful for every such inspector to visit and inspect, at any time, any place within his district, notice of which place has been given, as is herein-after directed, that it is intended there to practise anatomy.

VI. And be it enacted, That it shall be lawful for his Majesty to grant to every such inspector such an annual salary, not exceeding one hundred pounds, for his trouble, and to allow such a sum of money for the expenses of his office as may appear reasonable; such salaries and allowances to be charged on the Consolidated Fund of the United Kingdom, and to be payable quarterly; and that an annual return of all such salaries and allowances shall be made to Parliament.

VII. And be it enacted, That it shall be lawful for any executor or other party having lawful possession of the body of any deceased person, and not being an undertaker or other party intrusted with the body for the purpose only of interment, to permit the body of such deceased person to undergo anatomical examination, unless, to the knowledge of such executor or other party, such person shall have expressed his desire, either in writing at any time during his life, or verbally in the presence of two or more witnesses during the illness whereof he died, that his body after death might not undergo such examination, or unless the surviving husband or wife, or any known relative of the deceased person, shall require the body to be interred without such examination.

VIII. And be it enacted, That if any person, either in writing at any time during his life, or verbally in the presence of two or more witnesses during the illness whereof he died, shall direct that his body after death be examined anatomically, or shall nominate any party by this Act authorized to examine bodies anatomically to make such examination, and if, before the burial of the body of such person, such direction or nomination shall be made known to the party having lawful possession of the dead body, then such last-mentioned party shall direct such examination to be made, and, in case of any such nomination as aforesaid, shall request and permit any party so authorized and nominated as aforesaid to make such examination, unless the deceased person's surviving husband or wife, or nearest known relative, or any one or more of such person's nearest known relatives, being of kin in the same degree, shall require the body to be interred without such examination.

IX. Provided always, and be it enacted, That in no case shall the body of any person be removed for anatomical examination from any place where such person may have died, until after forty-eight hours from the time of such person's decease, or until after twenty-four hours notice, to be reckoned from the time of such decease, to the inspector of the district, of the intended removal of the body, or, if no such inspector have been appointed, to some physician, surgeon, or apothecary residing at or near the place of death, nor unless a certificate stating in what manner such person came by his death shall previously to the removal of the body have been signed by the physician, surgeon, or apothecary who attended such person during the illness whereof he died, or if no such medical man attended such person during such illness, then by some physician, surgeon, or apothecary who shall be called in after the death of such person to view his body, and who shall state the manner or cause of death according to the best of his knowledge and belief, but who shall not be concerned in examining the body after removal; and that in case of such removal, such certificate shall be delivered, together with the body, to the party receiving the same for anatomical examination.

X. And be it enacted, That it shall be lawful for any Member or Fellow of any College of Physicians or Surgeons, or any graduate or licentiate in medicine, or any person lawfully qualified to practise medicine in any part of the united kingdom, or any professor, teacher, or student of anatomy, medicine, or surgery, having a licence from his Majesty principal secretary of state or chief secretary as aforesaid, to receive or possess for anatomical examination, or to examine anatomically, the body of any person deceased, if permitted or directed so to do by a party who had at the time of giving such permission or direction lawful possession of the body, and who had power, in pursuance of the provisions of this Act, to permit or cause the body to be so examined, and provided such certificate as aforesaid were delivered by such party together with the body.

XI. And be it enacted, That every party so receiving a body for anatomical examination after removal shall demand and receive, together with the body, a certificate as aforesaid, and shall, within twenty-four hours next after such removal, transmit to the inspector of the district such certificate, and also a return stating at what day and hour and from whom the body was received, the date and place of death, the sex, and (as far as is known at the time) the christian and surname, age, and last place of abode of such person, or, if no such inspector have been appointed, to some physician, surgeon, or apothecary residing at or near the place to which the body is removed, and shall enter or cause to be entered the aforesaid particulars relating thereto, and a copy of the certificate he received therewith, in a book to be kept by him for that purpose, and shall produce such book whenever required so to do by any inspector so appointed as aforesaid.

XII. And be it enacted, That it shall not be lawful for any party to carry on or teach anatomy at any place, or at any place to receive or possess for anatomical examination, or examine anatomically, any deceased person's body after removal of the same, unless such party, or the owner or occupier of such place, or some party by this Act authorized to examine bodies anatomically, shall, at least one week before the first receipt or possession of a body for such purpose at such place, have given notice to the said secretary of state or chief secretary, as the case may be, of the place where it is intended to practise anatomy.

XIII. Provided always, and be it enacted, That every such body so removed as aforesaid for the purpose of examination shall, before such removal, be placed in a decent coffin or shell, and be removed therein; and that the party removing the same, or causing the same to be removed as aforesaid, shall make provision that such body, after undergoing anatomical examination, be decently interred in consecrated ground, or in some public burial ground in use for persons of that religious persuasion to which the person whose body was so removed belonged; and that a certificate of the interment of such body shall be transmitted to the inspector of the district within six weeks after the day on which such body was received as aforesaid.

XIV. And be it enacted, That no member or fellow of any college of physicians or surgeons, or any graduate or licentiate in medicine, nor any person lawfully qualified to practise medicine in any part of the united kingdom, nor any professor, teacher, or student of anatomy, medicine, or surgery, having a licence from his Majesty's principal secretary of state or chief secretary as aforesaid, shall be liable to any prosecution, penalty, forfeiture, or punishment for receiving or having in his possession for anatomical examination, or for examining anatomically, any dead human body, according to the provisions of this Act.

XV. And be it enacted, That nothing in this Act contained shall be construed to extend to or to prohibit any post-mortem examination of any human body required or directed to be made by any competent legal authority.

XVI. And whereas an Act was passed in the ninth year of the reign of his late Majesty, for consolidating and amending the statutes in England relative to offences against the person, by which latter Act it is enacted, that the body of every person convicted of murder shall, after execution, either be dissected or hung in chains, as to the court which tried the offender shall seem meet; and that the sentence to be pronounced by the court shall express that the body of the offender shall be dissected or hung in chains, whichsoever of the two the court shall order; be it enacted, That so much of the said last-recited Act as authorizes the court, if it shall see fit, to direct that the body of a person convicted of murder shall, after execution, be dissected, be and the same is hereby repealed; and that in every case of conviction of any prisoner for murder, the court before which such prisoner shall have been tried shall direct such prisoner either to be hung in chains, or to be buried within the precincts of the prison in which such prisoner shall have been confined after conviction, as to such court shall seem meet; and that the sentence to be pronounced by the court shall express that the body of such prisoner shall be hung in chains, or buried within the precincts of the prison, whichever of the two the court shall express that the body of such prisoner shall be hung in chains, or buried within the precincts of the prison, whichever of the two the court shall order.

XVII And be it enacted, That if any action or suit shall be commenced or brought against any person for any thing done in pursuance of this Act, the same shall be commenced within six calendar months next after the cause of action accrued; and the defendant in every action or suit may, at his election, plead the matter especially, or the general issue not guilty, and give this Act and the special matter in evidence at any trial to be had thereupon.

XVIII. And be it enacted, That any person offending against the provisions of this Act in England or Ireland shall be deemed and taken to be guilty of a misdemeanor, and, being duly convicted thereof, shall be punished by imprisonment for a term not exceeding three months, or by a fine not exceeding fifty pounds, at the discretion of the court before which he shall be tried; and any person offending against the provisions of this Act in Scotland shall, upon being duly convicted of such offence, be punished by imprisonment for a term not exceeding three months, or by a fine not exceeding fifty pounds, at the discretion of the court before which he shall be tried.

XIX. And in order to remove doubts as to the meaning of certain words in this Act, be it enacted, That the words "person and party" shall be respectively deemed to include any number of persons, or any society, whether by charter or otherwise; and that the meaning of the aforesaid words shall not be restricted although the same may be subsequently referred to in the singular number and masculine gender only.

XX. And be it enacted, That this Act shall commence and take effect from and after the first day of August in the present year.

XXI. And be it enacted, That this Act may be altered or amended during the present session of Parliament.

*Directions of the London Inspectors of Anatomy, under the New Anatomical Bill.*

SIR,—I am desirous, on being appointed inspector pursuant to the Act for regulating the schools of anatomy, to solicit your co-operation in carrying into execution the enactments of the legislature.

In order to facilitate this object, and to secure uniformity and regularity of proceeding, I beg leave to submit to you a general form of return, as required by the statute, together with an abstract of the duties which appear to me to be imposed by it upon teachers of anatomy. It is unnecessary for me to point out how important it is, for the satisfaction of the public mind, that the natural feelings of human nature should be consulted by a strict attention to the thirteenth section of the Act, which provides for the decent interment of bodies after they have undergone anatomical examination.

I have only to add that I shall be anxious to receive such advice and information as your knowledge and experience may suggest, and to assure you that any such communications, as well as the returns, shall be considered by me strictly secret and confidential, and shall only be reported to the public authorities as directed and required by the Act of Parliament. I am, Sir,

Your obedient servant,

JAS. C. SOMERVILLE, M.D.

Office of Inspector of Anatomy,
5, Saville Row.
30th Aug. 1832.

*Form of Application.*

The party applying for a licence to practise anatomy must make a written application to the secretary of state, signed by him with his christian and surname at length, in which application he must state, he applies for a licence to practise anatomy, in pursuance of the Act passed for regulating schools of anatomy. He must state also the place of his residence, and the place where he is about to carry on the practice of anatomy, and must further state that he is, according as the case may be, [a fellow or a member of a College of Physicians or Surgeons], *or* [a graduate or licentiate in medicine], *or* [a person lawfully qualified to practise medicine in England, Scotland, or Ireland], *or* [a professor or teacher of anatomy, medicine, or surgery], *or* [a student attending a school of anatomy.]

This application is to be countersigned by two justices of the peace acting for the county, city, or borough wherein the party applying resides, and so describing themseves, certifying the place of residence of the party, and also the place where they believe him to be about to carry on the practice of anatomy.

*Duties of Teachers of Anatomy, according to the Act for regulating Schools of Anatomy.*

To obtain a licence according to the provisions of the Act 2d and 3d Will. IV. c. 75.

Upon receiving a body for anatomical examination, to demand and receive with the body a certificate from the physician, surgeon, or apothecary who attended the person during the illness of which he died, or from some surgeon, physician, or apothecary who shall have been called in after the death of such person to view the body, stating the manner or cause of the death, and to transmit, within twenty-four hours next after the removal of the body, such certificate to the inspector of the district; and also a return stating at what day and hour and for whom the body was received, the date and place of death, the sex, and, as far as is known at the time, the christian and surname, age, and last place of abode of such person; or if no such inspector shall have been appointed, to some physician, surgeon, or apothecary residing at or near the place to which the body is removed; and to enter or cause to be entered the aforesaid particulars, and a copy of the certificate, in a book to be kept by him for that purpose, and to produce such book whenever required by the inspector.

To give notice to the secretary of state of every place where it is intended to practise anatomy at least one week before the first receipt or possession of a dead body for such purpose at such place.

To take care that every body removed for examination shall be removed in a decent and fitting manner, and to make provision that such body, after undergoing anatomical examination, be decently interred, according to the thirteenth clause of the said Act; and that a certificate of the interment of such body be transmitted to the inspector of the district within six weeks after the day of the reception of such body.

*Duties of the Inspector.*

He shall keep an account of all the schools of anatomy licensed by the secretary of state within the district for which he shall be appointed, with the names and residences of the teachers.

He shall make a quarterly return to the secretary of state of the body of every deceased person that during the preceding quarter has been removed for anatomical examination to every separate place in his district where anatomy is carried on; distinguishing the sex, and, as far as is known at the time, the name and age of each person whose body has been so removed as aforesaid.

He shall visit and inspect from time to time every place within his district, of which place notice has been given that it is intended there to practise anatomy; he shall take care that the provisions of the Act of Parliament are enforced, and shall report to the secretary of state any irregularities or offences against the Act which he shall observe.

He shall enter in a register kept for the purpose the returns from each school separately, and shall keep copies of all his returns and reports to the secretary of state, and shall enter all the correspondence relating to the duties of his office in a letter-book.—

CONFIDENTIAL RETURN *from the School of Anatomy at*_____________

As required by the 2nd and 3rd Will. IV. c. 75.

| The Christian Name and the Surname of the Deceased. | Age and Sex. | Last Place of Abode of deceased Person. | Date and Place of Death. | Time of Removal. | Day and Hour at which the Body was received, and from whom received. | Observations. |
|---|---|---|---|---|---|---|
| | | | | | | |

(Signed)_____________ Teacher."

It will be observed that this Act says nothing respecting the supply of dead bodies, and the only ostensible source is that indicated in section VIII., viz. the bequest by any one of his or her own body, provided none of the "nearest known relatives" object. This source we may readily imagine to be not quite sufficient for the wants of the profession, yet this is the only, or at least the chief, source clearly pointed out. So far, then, the Act might seem, to ordinary observers, to resemble the play of Hamlet, without the part of the Prince of Denmark. It would, indeed, appear to be an Act for Regulating Schools of Anatomy, but not for Supplying them.

But when we look more closely, we shall see that dead bodies are implied to be a property—a legal property. But with whom? With relatives, of course. Others have also a legal right to dispose of the bodies of the deceased, at least such a right is dimly hinted at in the 7th section, which we reprint.

"VII. And be it enacted, That it shall be lawful for any executor or other party having lawful possession of the body of any deceased person, and not being an undertaker or other party entrusted with the body for the purpose only of interment, to permit the body of such deceased person to undergo anatomical examination, unless, to the knowledge of such executor or other party, such person shall have expressed his desire, either in writing at any time during his life, or verbally in the presence of two or more witnesses during the illness whereof he died, that his body after death might not undergo such examination, or unless the surviving husband or wife, or any known relative of the deceased person, shall require the body to be interred without such examination."

Thus the parties having the disposal of dead bodies are declared to be "any executors or other party having lawful possession," "not being an undertaker, or other party entrusted with the body for the purpose only of interment." So an executor may dispose of the body, provided there be no relatives to object, and provided also the deceased has expressed no objection before death. To get a body, in short, from an executor there must be no repugnant near relations, no previous objection on the part of the patient, and finally the executor himself must be willing. We fancy we need not expect much from executors. The question then comes to be this, can the words "other party" apply to overseers, and will they be induced to consider them as applicable. It is quite apparent that they may or they may not, that in fact they have the option of even construing the Act as totally inapplicable to them. Here then we are left at the mercy of the individual overseer, and all that we obtain from him must be from motives of interest, or it must be an obligation. Those who are acquainted with overseers will best know what they may expect from their courtesy; for our own parts we are little versed in parish arcana. Self-interest may work in two ways. First, the parish may escape the pauper's burial; and secondly, they may even drive a bargain for his corpse. The latter we presume will not be attempted. The expense then taken from the parish must naturally fall on him who obtains the body, that is upon the pupil. The London Medical Journal asserts that funeral expenses and burial fees will amount, on the lowest calculation, to from three to five pounds in every instance, and that if this be added to the sum which must be paid to relatives the price will be considerably increased.

So much for the question of expense, but the great point after all is the adequacy of supply. In Massachusetts a simple method has been adopted—the unclaimed poor have been dedicated to the purposes of dissection. The supply is defined, liable to no misconception, and we believe that it is adequate. Here there is no obvious supply at all, but a dark and a quiet league is to be struck between the anatomist and the parish. In point of efficiency no one, we believe, admits that such a provision, or rather want of provision, is comparable for a moment to the plain, consistent, and straight forward declaration that those who die friendless in public institutions may be dissected. It is right, however, to state that the advocates of the Act declare that it will work well, that the supply will be sufficient, and the expenses moderate. A short trial will determine the justice or otherwise of this expectation.

But supposing all this to be true, there are other, and very important considerations behind. The aim of a legislator on anatomy, we conceive to be, to confer the greatest quantum of benefit on science, with the least amount of injury to private feelings and public morals. This is the problem to be solved. Now which is most likely to foster prejudice, and engender vice—the plain and open declaration that the bodies of the friendless will be dissected—or a dark, cogging, intriguing covenant between the surgeon and the parish, or the surgeon and the relations of the dead? For our own parts we do not hesitate for one instant to prefer the former, and to express our unqualified disapprobation of the latter. By the one piece of legislation all is clear and undisguised, and the pauper to be dissected knows that this is one of the many evils which his vices or his misfortunes have brought upon him. By the other law, our own law, a door is opened to intrigue, sordid feelings, and even to a base traffic in dead bodies, on the part of mercenary relatives. A parish may determine, if it please, to sell its dead paupers, or the father may turn a penny by the corpse of his child, the wife by the body of her husband. Thus the trade in anatomy will be kept alive, and the private companies of resurrectionists may be superseded by huge parish monopolies, or licensed retailing relations. It will be said that these are idle fears. They may be so, but we are sure that the principle of the Act admits of these mal-practices, and is far more calculated to destroy morality than to encourage it. The other law may be stern, may in some degree be cruel, may be looked on as offering the pauper to the Moloch of science, but at least it is inflexible, it says to him, "you are vicious or unfortunate and poor, and we anatomize you when dead for the benefit of your species; but be assured that we do not make you an instrument of private or of public barter, we do not bring your corpse to a secret and unholy market."

Our space is too limited to suffer us to proceed further in these observations but we have thought it right to record our deep-seated objections against the constitution of the Act, now that it is about to be brought into operation. None will feel greater pleasure than we shall, if those objections should prove to be unfounded, but we feel a conviction too deep to be easily shaken, that less prejudice would ultimately have been excited, and that a more efficient, as well as a more honourable supply of dead bodies would have been obtained, by declaring manfully and honestly that the unclaimed poor would be dissected.*

* It appears that one of those taxes upon knowledge in which this country is so rife, has been mixed up with the Act in its operation. The clerks of the Home-Office, have been the first to make something by the dead, and £2. 2s. 6d. is charged for the license. Lord Melbourne having been written to, replied, very justly, that "the fee is in accordance with the *invariable* usage of the office," and his Lordship might have added, of every other office in this country. It must indeed be a legislative caput mortuum, which can afford in its passage through executive presses, no extract in the shape of fees. The living and the dead are equally rife in precious ore to the searching fingers of these official anatomists. It is well remarked by our contemporary, the London Medical and Surgical Journal, that the medical profession is the only one expected to give certificates for nothing. Those who are directed by the Act to sign the certificate of the patient's decease, &c. would not find it the invariable usage of the Home-Office to remunerate them.

### LIV.

### Undercliffe—Isle of Wight.

We beg to solicit the attention of our brethren to the following points. Mr. Whiskard, a surgeon, who was afflicted with pulmonary consumption, resided for some time at Undercliffe, in the Isle of Wight, and was so much benefitted and pleased with the situation, that he purchased a house there, and received invalid boarders. There is no doubt that he would have succeeded as far as pecuniary matters were concerned; but, unfortunately, his complaint had too far advanced, and he has just departed this world, leaving a widow and four orphans totally unprovided for, unless the patronage of the profession enables her to carry on the establishment which had been completed by her husband. We can safely recommend the situation for invalids requiring a mild sea air, sheltered from the northern winds, and the accommodations, we believe, in Mrs. Whiskard's house, will be found both comfortable and reasonable—the terms being two guineas a week for board and lodging. We hope and trust that the humane among our brethren, who may happen to have an opportunity of recommending patients to this delightful and salubrious retreat will not forget the widow and orphans of Mr. Whiskard.

LV.

PROVINCIAL MEDICAL AND SURGICAL ASSOCIATION.

ON the 19th July, upwards of fifty medical men met in the board-room of the Worcester Infirmary, to found an association bearing the above title. Its object is to promote cordial and social feeling among its members, and, by the publication of Transactions, to benefit the community and advance science. The Association owes its origin to the talent and energy of Dr. Hastings, a man who does as much honour to his profession as it can confer on him. We need scarcely say that we approve of such an association most warmly, we need scarcely add, that we would wish to see the social principle it developes diffused yet more widely amongst the medical practitioners of these Isles. Such associations are productive of benefit in several ways—first, by promoting harmony in a profession which is said to want it—secondly, by encouraging the publication of public and private experience—thirdly, by contributing to raise the professional and intellectual character of provincial surgeons and physicians. In a few years, the libeller or satirist may search in vain for that "low-born, cell-bred, selfish, servile crew," that private practitioners have, in former times, been said to be. With increased advantages of education, and such associations as these, the character of the whole profession is elevated.

LVI.

**Obituary.**

DR. J. ALLSOP.

IT is with unfeigned sorrow we record the death of a young, but a most zealous and talented physician of Birmingham, Dr. Josias Allsop, who fell a victim to the fatal epidemic, on the 6th of August last, after a short illness. He had been at Tipton (a village about 9 miles from Birmingham) where cholera was raging violently, and attended the poor there, day and night, for a fortnight, scarcely allowing himself any time for repose. He returned home on Saturday night, the 4th of August, greatly jaded and fatigued, observing to his wife that he had not had more than three hours sleep during the preceding week. On Sunday morning he seemed drowsy, but he dressed and went out to see some patients—returned to dinner—and appeared to have regained his strength and spirits. At 9 o'clock in the evening he ate a hearty supper, and retired to rest about 10 o'clock, apparently in health. At four o'clock in the morning he was called out of bed to see some patients, and returned at six o'clock. His wife, who had been recently confined of her third child, was soon informed by the nurse that her husband was very ill, and she instantly arose and went to him. The unfortunate lady was horrified at beholding her husband sitting up in bed, more like a corpse than a living being, and taking down notes of his own case! He expressed himself glad at having an attack of the epidemic, and said he would now be able to understand and to treat the disease better than before! But he soon became extremely weak, and gave up his pen to his wife, while he dictated to her the symptoms which he felt. The violence of the cramps soon compelled him to give over this task also, and after severe sufferings for some hours, he fell into a state of insensibility, from which he never after recovered! Thus perished, at the early age of 27 years, a most accomplished and intelligent physician, who, we have no doubt, fell a victim to his zeal in the investigation of a mysterious epidemic. Our readers are aware that Dr. Allsop wrote several papers on cholera in our contemporary the Medical Gazette—and we may here state that he was the author of the article "*Progress of Cholera in England*," from page 645 to 674 in our last volume, bearing the signature A.—to which we can confidently refer for proof of the learning, talents, and judgment of its author.

Deeply do we regret to add that Dr. Allsop has left an amiable widow and three orphans to deplore the premature death of an affectionate parent, who, had life been spared, would have honourably distinguished himself in his profession, and provided amply for his family.

